# Development and Validation of a Robust Ferroptosis-Related Prognostic Signature in Lung Adenocarcinoma

**DOI:** 10.3389/fcell.2021.616271

**Published:** 2021-06-24

**Authors:** Anran Zhang, Jinpo Yang, Chao Ma, Feng Li, Huan Luo

**Affiliations:** ^1^Department of Oncology, Henan Provincial People’s Hospital, Zhengzhou University People’s Hospital, Henan University People’s Hospital, Zhengzhou, China; ^2^Department of Medical Oncology, The Affiliated Cancer Hospital of Zhengzhou University, Henan Cancer Hospital, Zhengzhou, China; ^3^Charité – Universitätsmedizin Berlin, Freie Universität Berlin, Humboldt-Universität zu Berlin, Berlin Institute of Health, Berlin, Germany; ^4^Berlin Institute of Health Center for Regenerative Therapies and Berlin-Brandenburg Center for Regenerative Therapies (BCRT), Charité – Universitätsmedizin Berlin, Berlin, Germany; ^5^Department of Surgery, Competence Center of Thoracic Surgery, Charité University Hospital Berlin, Berlin, Germany

**Keywords:** biomarkers, tumor immunity, prognosis, risk score, gene signature, ferroptosis, lung adenocarcinoma

## Abstract

**Background:**

Lung adenocarcinoma (LUAD) is the most common subtype of non-small cell lung cancer. Ferroptosis is a newly recognized process of cell death, which is different from other forms of cell death in morphology, biochemistry, and genetics, and has played a vital role in cancer biology. This study aimed to identify a ferroptosis-related gene signature associated with LUAD prognosis.

**Methods:**

Dataset TCGA-LUAD which came from the TCGA portal was taken as the training cohort. GSE72094 and GSE68465 from the GEO database were treated as validation cohorts. Two hundred fifty-nine ferroptosis-related genes were retrieved from the FerrDb database. In the training cohort, Kaplan–Meier and univariate Cox analyses were conducted for preliminary screening of ferroptosis-related genes with potential prognostic capacity. These genes then entered into the LASSO Cox regression model, constructing a gene signature. The latter was then evaluated in the training and validation cohorts *via* Kaplan–Meier, Cox, and ROC analyses. In addition, the correlations between risk score and autophagy were examined by Pearson correlation coefficient. The analyses of GSEA and immune infiltrating were performed for better studying the function annotation of the gene signature and the character of each kind of immune cells played in the tumor microenvironment.

**Results:**

A 15-gene signature was found from the training cohort and validated by Kaplan–Meier and Cox regression analyses, revealing its independent prognosis value in LUAD. Moreover, the ROC analysis was conducted, confirming a strong predictive ability that this signature owned for LUAD prognosis. One hundred fifty-one of 222 (68.01%) autophagy-related genes were discovered significantly correlated with risk scores. Analyses of GSEA and immune infiltration exhibited in detail the specific pathways that associate with the 15-gene signature and identified the crucial roles of resting mast cells and resting dendritic cells owned in the prognosis of the 15-gene signature.

**Conclusion:**

In this present study, a novel ferroptosis-related 15-gene signature (RELA, ACSL3, YWHAE, EIF2S1, CISD1, DDIT4, RRM2, PANX1, TLR4, ARNTL, LPIN1, HERPUD1, NCOA4, PEBP1, and GLS2) was built. It could accurately predict the prognosis of LUAD and was related to resting mast cells and resting dendritic cells, which provide potential for the personalized outcome prediction and the development of new therapies in LUAD population.

## Introduction

Lung cancer is the leading cause of death from cancer. In the United States, there will be approximately 228,820 newly diagnosed cases and 135,720 deaths in 2020 (Siegel et al., 2020). Lung cancer mainly consists of two subtypes: non-small cell lung cancer (NSCLC) and small cell lung cancer (SCLC). NSCLC accounts for almost 80% of lung cancer cases and is made up of two major types, lung adenocarcinoma (LUAD) and lung squamous cell carcinoma (Herbst et al., 2018). LUAD is the predominant histology, and the rates are still increasing (Siegel et al., 2020). In recent years, several treatment advances have been made, especially the advancement of targeted therapy and the emergence of immunotherapy (Li et al., 2018; Low et al., 2019). However, these two methods can only benefit a limited number of subtypes, and the overall survival rate of LUAD is still very low (Wang et al., 2020). Therefore, further studies on therapeutic monitoring and prognostic assessment of LUAD are crucial for clinicians and patients.

Ferroptosis is a novel type of cell death that was discovered in recent years and is usually accompanied by a large amount of iron accumulation and lipid peroxidation during the cell death process (Li et al., 2020). It is characterized by increased mitochondrial membrane density and cell volume contraction, which is different from other morphological, biochemical, and genetically regulated cell deaths (Li et al., 2020; Tao et al., 2020). Recent research has shown that ferroptosis is closely associated with the pathophysiological process of many diseases, such as tumors, neurological disorders, ischemia–reperfusion injury, kidney injury, and blood diseases (Li et al., 2020). For the past few years, the induction of ferroptosis has emerged as a promising therapeutic alternative to trigger cancer cell death, especially for malignancies that are resistant to traditional treatments (Hassannia et al., 2019; Liang et al., 2019; Bebber et al., 2020). Apart from ferroptosis-inducing agents, numerous genes have also been identified as modulators or markers of ferroptosis (Stockwell et al., 2017; Bersuker et al., 2019; Doll et al., 2019; Hassannia et al., 2019; Liu et al., 2020). The fast-growing studies of ferroptosis in cancer have boosted a perspective for its usage in cancer therapeutics (Mou et al., 2019). The expression of FSP1 is correlated with the ferroptosis resistance of lung cancer cell lines, indicating that the upregulation of FSP1 is a strategy of ferroptosis escape in lung cancer (Doll et al., 2019). Additionally, in non-small-cell lung cancer (NSCLC) cell lines, it was shown that the level of MAPK pathway activity correlates with sensitivity to ferroptosis induced by cystine deprivation (Poursaitidis et al., 2017). Interestingly, LSH inhibits ferroptosis and promotes lung tumorigenesis by affecting metabolic genes through chromatin modification (Jiang et al., 2017). RNA sequencing in NSCLC cells showed that SLC7A11, a key gene associated with ferroptosis through its role in controlling iron concentrations, can be downregulated by XAV939 (an inhibitor of NSCLC), as the target genes of lncRNAs, and suppress the development of NSCLC *via* ferroptosis-mediated pathways (Yu et al., 2019). LSH promotes the expression of LINC00336 by upregulating ELAVL1 through the p53 signaling pathway in lung cancer (Wang M. et al., 2019; Wu et al., 2020). LINC00336 acts as a crucial inhibitor of ferroptosis in carcinogenesis by decreasing intracellular levels of iron and lipid ROS through interacting with ELAVL1 (ELAV-like RNA-binding protein 1), which has been recognized as a novel regulator of ferroptosis (Wang M. et al., 2019; Wu et al., 2020). The above evidence has highlighted the importance of ferroptosis in lung cancer therapeutics, but the roles of ferroptosis in tumorigenesis and development remain unclear.

Autophagy is the natural, regulated mechanism of the cell that removes unnecessary or dysfunctional components. It allows the orderly degradation and recycling of cellular components (Mizushima and Komatsu, 2011). The original study shows that ferroptosis is morphologically, biochemically, and genetically distinct from autophagy and other types of cell death (Kang and Tang, 2017). However, recent studies demonstrate that activation of ferroptosis is indeed dependent on the induction of autophagy (Kang and Tang, 2017). In addition, accumulating studies have revealed cross talk between autophagy and ferroptosis at the molecular level (Zhou et al., 2019).

Currently, several studies were mining prognostic gene signatures related to ferroptosis in tumors from public databases (Liang et al., 2020; Liu et al., 2020). Liu confirmed that the ferroptosis-related 19-gene signature could predict glioma patient survival (Liu et al., 2020). Liang et al. discovered a novel ferroptosis-related prognostic gene signature for hepatocellular carcinoma (Liang et al., 2020). However, there is still no study to declare whether there is a ferroptosis-related prognostic gene signature able to predict LUAD outcomes. In order to fill this blank and widen the potential of diagnosis and therapy of LUAD, the present study performed comprehensive analyses utilizing TCGA and GEO, along with ferroptosis-related genes identified in previous studies to determine and validate the minimum number of potentially robust prognostic genes of LUAD. In addition, we determined the correlations between the signature and autophagy and studied the characteristics of the gene signature in the tumor microenvironment through GSEA and immune infiltration analysis.

## Materials and Methods

### Mining From TCGA and GEO Databases

We selected the TCGA-LUAD dataset as the training cohort and only included cases that meet the following criteria: (1) gene expression data is available; (2) survival data is available; (3) and follow-up time is greater than 0 days. Finally, 500 LUADs were included, and their gene expression profile, survival data, and clinical characteristics were downloaded from GDC Xena Hub^1^. Besides, datasets of GSE72094 (*n* = 442) and GSE68465 (*n* = 443) were downloaded from the GEO database and taken as validation cohorts to examine the gene signature we trained.

### Ferroptosis-Related Genes

FerrDb^2^ is the world’s first manually curated database for regulators and markers of ferroptosis and ferroptosis–disease associations (Zhou and Bao, 2020). In this database, genes of drivers, suppressors, and markers of ferroptosis were listed based on the knowledge of previous studies (Supplementary Table 1). The unique 259 genes (Supplementary Table 2) were identified as the ferroptosis-related genes and entered to the next procedure.

### Construction and Validation of the Prognostic Ferroptosis-Related Gene Signature in LUAD

Kaplan–Meier and univariate Cox analyses were applied in the training cohort to determine the association between the gene expression and patients’ overall survival for potential prognostic genes, and *P*-value < 0.05 was considered to be statistically significant. The genes in the overlapped part of potential prognostic genes and ferroptosis-related genes were identified as potential prognostic ferroptosis-related genes, which then entered into an overall survival-based LASSO Cox regression model with penalty parameter tuning conducted by 10−fold cross-validation to detect the best penalty parameter lambda (Tibshirani, 1997; Sauerbrei et al., 2007; Friedman et al., 2010; Goeman, 2010). Based on the best lambda value, a list of prognostic genes with coefficients was harvested. As shown in the below formula, the risk score of each LUAD case could be obtained based on the expression level of each prognostic gene and its corresponding coefficient. In the formula, n, Expi, and βi indicate the number of hub genes, gene expression level, and regression coefficient value, respectively.

$$R\,i\,s\,k\,s\,c\,o\,r\,e=\sum_{i}^{n}E\,x\,p\,i*\beta \,i$$

In the training cohort, patients were divided into low- and high-risk groups by using the median risk score as a cutoff point, and the survival difference of the two groups was measured by Kaplan–Meier analysis. Also, Cox and ROC analyses were conducted for further assessing of the gene signature prognostic ability. Moreover, in the validation cohorts, the same formula and statistical methods was adopted to validate the prognostic capacity of the gene signature.

### Relationships Between Gene Signature and Autophagy in LUAD

To explore the relationship between autophagy and our gene signature, we identified 222 autophagy-associated genes from the Human Autophagy Database (HADb^3^), which contains an exhaustive, up-to-date list of human autophagy-related genes (Abdul Rahim et al., 2017) (Supplementary Table 3). The Pearson correlation coefficient was applied to assess the correlation between autophagy and gene signature risk score. In addition, the GEPIA2 online tool^4^ was applied to plot survival heatmaps of top 10 correlated genes (Tang et al., 2019). *P*-value < 0.05 was considered statistically significant.

### Gene-Set Enrichment Analysis (GSEA)

Gene-set enrichment analysis was performed based on Hallmark (v7.1^5^) gene-set collections using GSEA software (v4.1.0^6^) in the training cohort to uncover the functions and pathways in the differentially expressed genes (DEGs) between high- and low-risk groups. Gene sets with | NES | > 1, NOM *p*-value < 0.05, and FDR *q*-value < 0.25 were considered significant.

### Identification of the Relationship Between Gene Signature and 22 Tumor-Infiltrating Immune Cells (TIC)

The relative proportion of 22 TICs in the training cohort was calculated using the CIBERSORT algorithm (Thorsson et al., 2018; Newman et al., 2019). After the quality filtering (*P*-value < 0.05), 391 LUADs were selected for the next analysis. The correlations between 22 kinds of TICs were examined by the Pearson coefficient.

For identifying the relationship between the 22-TIC proportion and risk score, an integrated analysis of consisting of the Spearman coefficient and Wilcoxon rank-sum was applied. Based on the 22-TIC proportion and survival data, Cox and Kaplan–Meier analysis were deployed to screen 22 TICs with prognostic meaning. *P*-value < 0.05 was a statistically significant threshold.

### Statistical Analysis

The LASSO analysis was conducted applying the “glmnet” R package. Kaplan–Meier analysis was conducted using “survival” and “survminer” R packages. Univariate and multivariate Cox proportional hazard regression analyses were conducted using “survival” R package. The ROC analysis was applied using “survivalROC” R package. *P*-value < 0.05 was a statistically significant threshold.

## Results

### Cohort Characteristics

**FIGURE 1:**
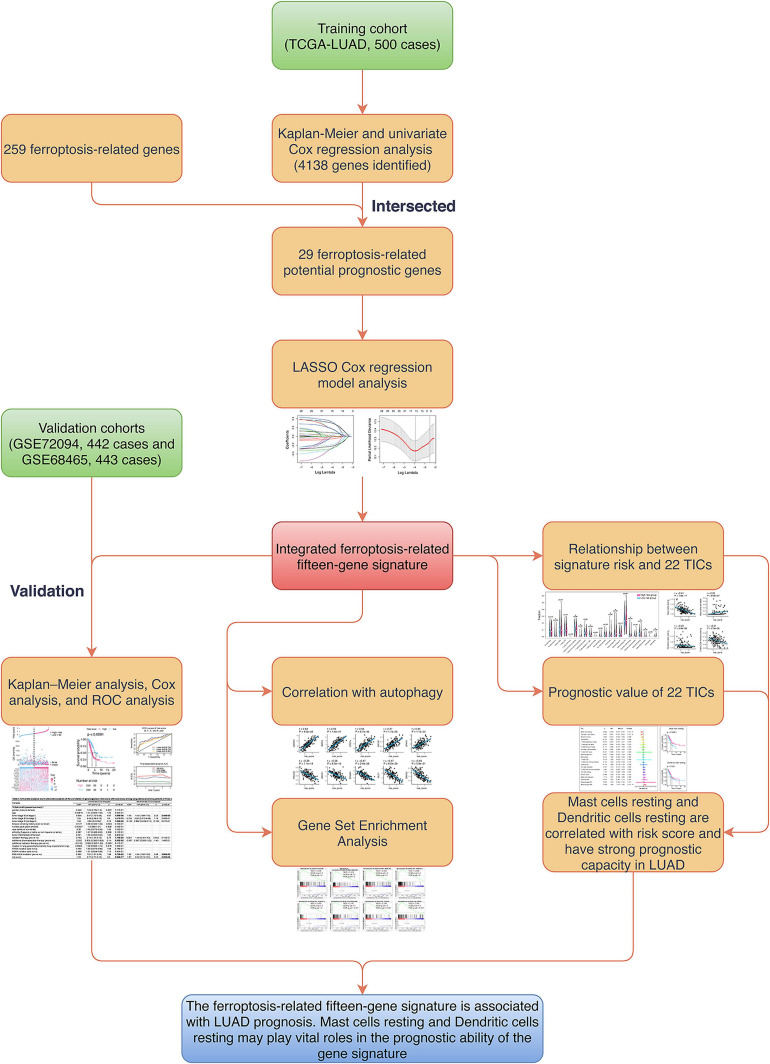
Flowchart of the study. LASSO, the least absolute shrinkage and selection operator Cox regression model; ROC, receiver operating characteristic; UM, uveal melanoma; TICs, tumor-infiltrating immune cells.

The flowchart of the present research is shown in Figure 1. Five hundred LUAD cases that came from the TCGA-LUAD cohort were taken as the training cohort. The datasets GSE72094 and GSE68465, containing 442 and 443 LUAD cases, respectively, were selected as the validation cohorts. The detailed clinical characteristics of cohorts included in this study are summarized in Table 1.

**TABLE 1:** Clinical characteristics of patients involved in the study.

Characteristics		Training cohort (TCGA-LUAD, 500 cases)	Validation cohort (GSE72094, 442 cases)	Validation cohort (GSE68465, 443 cases)
**Age**				
	< 65	219 (43.8%)	115 (26.02%)	214 (48.31%)
	> = 65	271 (54.2%)	306 (69.23%)	229 (51.69%)
	Unknown	10 (2%)	21 (4.75%)	0
**Gender**				
	Female	270 (54%)	240 (54.3%)	220 (49.66%)
	Male	230 (46%)	202 (45.7%)	223 (50.34%)
**T classification**				
	T1	167 (33.4%)	NA	150 (33.86%)
	T2	267 (53.4%)	NA	251 (56.66%)
	T3	45 (9%)	NA	28 (6.32%)
	T4	18 (3.6%)	NA	12 (2.71%)
	Unknown	3 (0.6%)	NA	2 (0.45%)
**N classification**				
	N0	324 (64.8%)	NA	299 (67.49%)
	N1	94 (18.8%)	NA	88 (19.86%)
	N2	69 (13.8%)	NA	53 (11.96%)
	N3	2 (0.4%)	NA	0
	Unknown	11 (2.2%)	NA	3 (0.68%)
**M classification**				
	M0	332 (66.4%)	NA	NA
	M1	24 (4.8%)	NA	NA
	Unknown	144 (28.8%)	NA	NA
**Tumor stage**				
	Stage I	268 (53.6%)	265 (59.95%)	NA
	Stage II	119 (23.8%)	69 (15.61%)	NA
	Stage III	80 (16%)	63 (14.25%)	NA
	Stage IV	25 (5%)	17 (3.85%)	NA
	Unknown	8 (1.6%)	28 (6.33%)	NA
**Race**				
	White	386 (77.2%)	399 (90.27%)	295 (66.59%)
	Black or African American	52 (10.4%)	13 (2.94%)	12 (2.71%)
	American Indian or Alaska native	1 (0.2%)	0	1 (0.23%)
	Asian	7 (1.4%)	3 (0.68%)	6 (1.35%)
	Unknown	54 (10.8%)	27 (6.11%)	129 (29.12%)
**Ethnicity**				
	Hispanic or Latino	7 (1.4%)	10 (2.26%)	NA
	Not Hispanic or Latino	381 (76.2%)	402 (90.95%)	NA
	Unknown	112 (22.4%)	30 (6.79%)	NA
**Tobacco smoking history**				
	Ever	415 (83%)	335 (75.79%)	300 (67.72%)
	Never	71 (14.2%)	33 (7.47%)	49 (11.06%)
	Unknown	14 (2.8%)	74 (16.74%)	94 (21.22%)
**Number pack years smoked**				
	< 30	115 (23%)	NA	NA
	> = 30	227 (45.4%)	NA	NA
	Unknown	158 (31.6%)	NA	NA
**Radiation therapy**				
	Yes	58 (11.6%)	NA	65 (14.67%)
	No	361 (72.2%)	NA	364 (82.17%)
	Unknown	81 (16.2%)	NA	14 (3.16%)
**Chemotherapy**				
	Yes	NA	NA	89 (20.09%)
	No	NA	NA	341 (76.98%)
	Unknown	NA	NA	13 (2.93%)
**Additional pharmaceutical therapy**				
	Yes	61 (12.2%)	NA	NA
	No	68 (13.6%)	NA	NA
	Unknown	371 (74.2%)	NA	NA
**Additional radiation therapy**				
	Yes	61 (12.2%)	NA	NA
	No	71 (14.2%)	NA	NA
	Unknown	368 (73.6%)	NA	NA
**EGFR mutation**				
	Yes	80 (16%)	47 (10.63%)	NA
	No	191 (38.2%)	395 (89.37%)	NA
	Unknown	229 (45.8%)	0	NA
**KRAS mutation**				
	Yes	23 (4.6%)	154 (34.84%)	NA
	No	39 (7.8%)	288 (65.16%)	NA
	Unknown	438 (87.6%)	0	NA
**STK11 mutation**				
	Yes	NA	68 (15.38%)	NA
	No	NA	374 (84.62%)	NA
**TP53 mutation**				
	Yes	NA	111 (25.11%)	NA
	No	NA	331 (74.89%)	NA
**EML4-ALK mutation**				
	Yes	34 (6.8%)	NA	NA
	No	207 (41.4%)	NA	NA
	Unknown	259 (51.8%)	NA	NA
**Tumor intermediate dimension**				
	< 1	287 (57.4%)	NA	NA
	> = 1	88 (17.6%)	NA	NA
	Unknown	125 (25%)	NA	NA
**Location in lung parenchyma**				
	Central lung	62 (12.4%)	NA	NA
	Peripheral lung	118 (23.6%)	NA	NA
	Unknown	320 (64%)	NA	NA
**Person neoplasm cancer status**				
	With tumor	134 (26.8%)	NA	NA
	Tumor free	243 (48.6%)	NA	NA
	Unknown	123 (24.6%)	NA	NA
**Vital status**				
	Alive	318 (63.6%)	298 (67.42%)	207 (46.73%)
	Dead	182 (36.4%)	122 (27.6%)	236 (53.27%)
	Unknown	0	22 (4.98%)	0

### Identification of Prognostic Ferroptosis-Related Gene Signature From the Training Cohort

**FIGURE 2:**
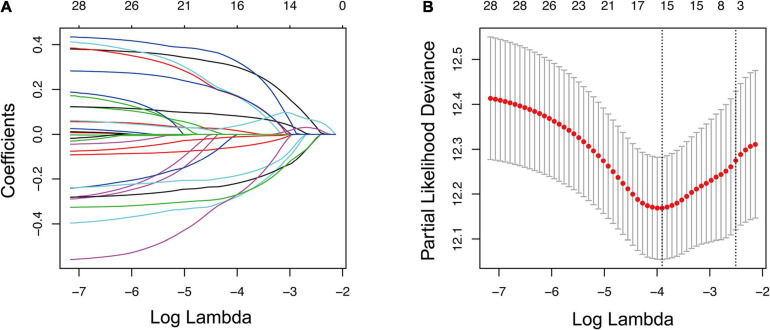
Construction of prognostic gene signature using LASSO regression analysis. **(A)** LASSO coefficient profiles of 29 ferroptosis-related potential prognostic genes. Each curve corresponds to a gene. **(B)** Selection of the optimal parameter in LASSO regression with 10-fold cross-validation. LASSO, the least absolute shrinkage and selection operator Cox regression model.

Kaplan–Meier and univariate Cox analyses were conducted on 500 LUAD cases in the training cohort to examine the prognostic relationship between each gene and overall survival. Only the gene showing *P*-values < 0.05 in both Kaplan–Meier and univariate Cox analyses was considered having potential prognostic capacity. Finally, 4,138 genes were identified as potential prognostic genes (Supplementary Table 4). The 4,138 potential prognostic genes and 259 ferroptosis-related genes were intersected to obtain a list containing 29 ferroptosis-related potential prognostic genes (Table 2). An overall survival-based LASSO Cox regression model was built using the 29 ferroptosis-related potential prognostic genes (Figure 2A). When 15 genes were included, the model achieved the best performance (Figure 2B). The regression coefficient of each gene was calculated and shown in Table 3.

**TABLE 2:** Twenty-nine ferroptosis-related potential prognostic genes generated from the training cohort.

Gene symbol	Description	Category	Kaplan–Meier analysis (*P*-value)	Univariate Cox regression analysis			
				HR	HR_95L	HR_95H	*P*-value
ACSL3	Acyl-CoA synthetase long-chain family member 3	Protein coding	1.82E-02	1.435	1.157	1.780	1.00E-03
ARNTL	Aryl hydrocarbon receptor nuclear translocator like	Protein coding	1.14E-02	0.676	0.492	0.929	1.59E-02
AURKA	Aurora kinase A	Protein coding	1.97E-02	1.268	1.103	1.458	8.61E-04
BACH1	BTB domain and CNC homolog 1	Protein coding	9.67E-03	1.310	1.012	1.696	4.05E-02
CDO1	Cysteine dioxygenase type 1	Protein coding	4.11E-02	0.713	0.522	0.973	3.30E-02
CISD1	CDGSH iron sulfur domain 1	Protein coding	2.41E-03	1.403	1.041	1.891	2.59E-02
CISD2	CDGSH iron sulfur domain 2	Protein coding	1.98E-03	1.372	1.005	1.872	4.64E-02
DDIT4	DNA damage inducible transcript 4	Protein coding	8.47E-03	1.214	1.072	1.374	2.22E-03
EIF2S1	Eukaryotic translation initiation factor 2 subunit alpha	Protein coding	6.52E-03	1.655	1.219	2.248	1.24E-03
FANCD2	FA complementation group D2	Protein coding	4.87E-03	1.428	1.095	1.861	8.48E-03
FLT3	Fms-related receptor tyrosine kinase 3	Protein coding	2.24E-02	0.452	0.247	0.829	1.02E-02
GLS2	Glutaminase 2	Protein coding	5.27E-03	0.356	0.177	0.718	3.91E-03
HELLS	Helicase, lymphoid specific	Protein coding	3.45E-03	1.282	1.025	1.604	2.96E-02
HERPUD1	Homocysteine-inducible ER protein with ubiquitin-like domain 1	Protein coding	8.21E-05	0.656	0.523	0.823	2.67E-04
IL33	Interleukin 33	Protein coding	3.77E-02	0.841	0.740	0.956	8.11E-03
KLHL24	Kelch-like family member 24	Protein coding	4.06E-02	0.761	0.580	0.998	4.81E-02
LINC00336	Long intergenic non-protein-coding RNA 336	RNA Gene	2.25E-02	0.339	0.120	0.952	4.01E-02
LPIN1	Lipin 1	Protein coding	4.63E-02	0.788	0.623	0.997	4.67E-02
NCOA4	Nuclear receptor coactivator 4	Protein coding	1.33E-02	0.776	0.603	0.999	4.89E-02
NRAS	NRAS proto-oncogene, GTPase	Protein coding	3.23E-02	1.360	1.077	1.717	9.68E-03
PANX1	Pannexin 1	Protein coding	3.25E-04	1.489	1.147	1.932	2.75E-03
PEBP1	Phosphatidylethanolamine-binding protein 1	Protein coding	6.95E-04	0.604	0.465	0.784	1.53E-04
RELA	RELA proto-oncogene, NF-KB subunit	Protein coding	4.45E-02	1.745	1.129	2.698	1.23E-02
RRM2	Ribonucleotide reductase regulatory subunit M2	Protein coding	5.69E-04	1.323	1.164	1.503	1.73E-05
SLC2A1	Solute carrier family 2 member 1	Protein coding	4.18E-05	1.261	1.134	1.401	1.71E-05
SLC3A2	Solute carrier family 3 member 2	Protein coding	2.93E-02	1.345	1.067	1.695	1.21E-02
TLR4	Toll-like receptor 4	Protein coding	4.18E-02	0.834	0.699	0.996	4.47E-02
VDAC2	Voltage-dependent anion channel 2	Protein coding	7.90E-03	1.592	1.213	2.090	7.99E-04
YWHAE	Tyrosine 3-monooxygenase/tryptophan 5-monooxygenase activation Protein Epsilon	Protein coding	4.52E-02	1.435	1.046	1.969	2.51E-02

**TABLE 3:** Fifteen ferroptosis-related prognostic genes obtained from LASSO Cox regression model.

Gene symbol	Description	Annotation*	Risk coefficient
ACSL3	Acyl-CoA synthetase long-chain family member 3	Ferroptosis suppressor	0.292079905
ARNTL	Aryl hydrocarbon receptor nuclear translocator-like	Ferroptosis suppressor	−0.052913519
CISD1	CDGSH iron sulfur domain 1	Ferroptosis suppressor	0.156274215
DDIT4	DNA damage-inducible transcript 4	Ferroptosis marker	0.079830296
EIF2S1	Eukaryotic translation initiation factor 2 subunit alpha	Ferroptosis marker	0.159470859
GLS2	Glutaminase 2	Ferroptosis driver	−0.267566406
HERPUD1	Homocysteine-inducible ER protein with ubiquitin-like domain 1	Ferroptosis marker	−0.213560347
LPIN1	Lipin 1	Ferroptosis driver	−0.179354454
NCOA4	Nuclear receptor coactivator 4	Ferroptosis driver	−0.253508649
PANX1	Pannexin 1	Ferroptosis driver	0.013029525
PEBP1	Phosphatidylethanolamine-binding protein 1	Ferroptosis driver	−0.262091825
RELA	RELA proto-oncogene, NF-KB subunit	Ferroptosis marker	0.31121564
RRM2	Ribonucleotide reductase regulatory subunit M2	Ferroptosis marker	0.043449128
TLR4	Toll-like receptor 4	Ferroptosis driver	−0.010188709
YWHAE	Tyrosine 3-monooxygenase/tryptophan 5-monooxygenase activation protein epsilon	Ferroptosis marker	0.160823922

*Drivers are genes that promote ferroptosis; suppressors are genes that prevent ferroptosis; markers are genes that indicate the occurrence of ferroptosis.

### Prognostic Value of the 15-Gene Signature in the Training and Validation Cohorts

**FIGURE 3:**
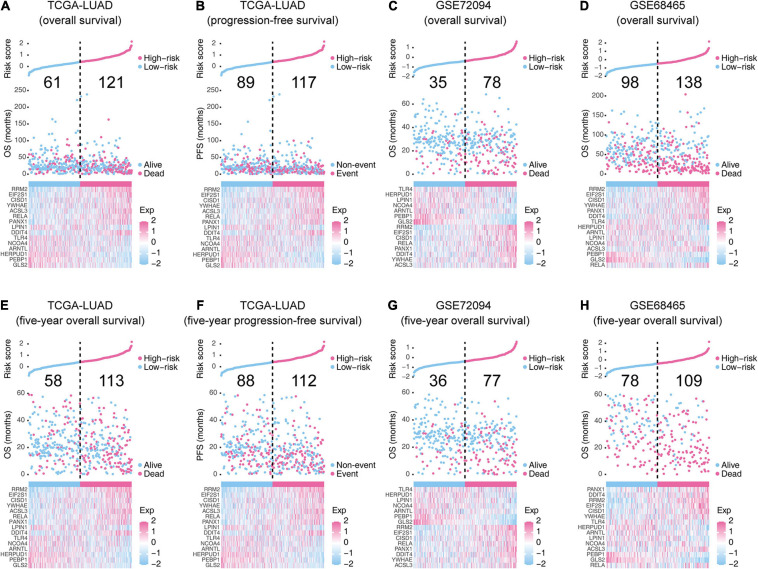
The overall performance of the 15-gene signature in the training and validation cohorts. **(A,C–E,G,H)** The upper parts indicate the distributions of risk score, and the middle parts indicate the patients’ survival status and survival time. **(B,F)** The upper parts indicate the distributions of risk score, and the middle parts indicate the patients’ event status and event time. The bold numbers in the middle-upper part of each graph represent the death/event counts in the low-risk and high-risk groups. The bottom part of each graph shows the heatmap of 15 gene expression profiles. Exp, expression level.

The risk score of each LUAD case was a linear combination of each 15-gene signature expression level and its risk coefficient. Patients were sorted to high- and low-risk groups based on their median. The distribution of risk scores, outcome status, and gene profiles of the 15-gene signature in training and validation cohorts are shown in Figure 3. As demonstrated in the graph (Figures 3A,D), more deaths or events happened in the high-risk groups than those in their corresponding low-risk groups. We checked the performance of this 15-gene signature in 5-year survival (Figures 3E,H) and found consistent patterns. The boxplots in Supplementary Figure 1 show the 15-gene expression distributions in the high- and low-risk groups. The analysis in the training cohort witnesses that ACSL3, YWHAE, DDIT4, PANX1, RELA, CISD1, EIF2S1, and RRM2 were overexpressed, while GLS2, PEBP1, ARNTL, NCOA4, LPIN1, HERPUD1, and TLR4 were downregulated in high-risk groups.

**FIGURE 4:**
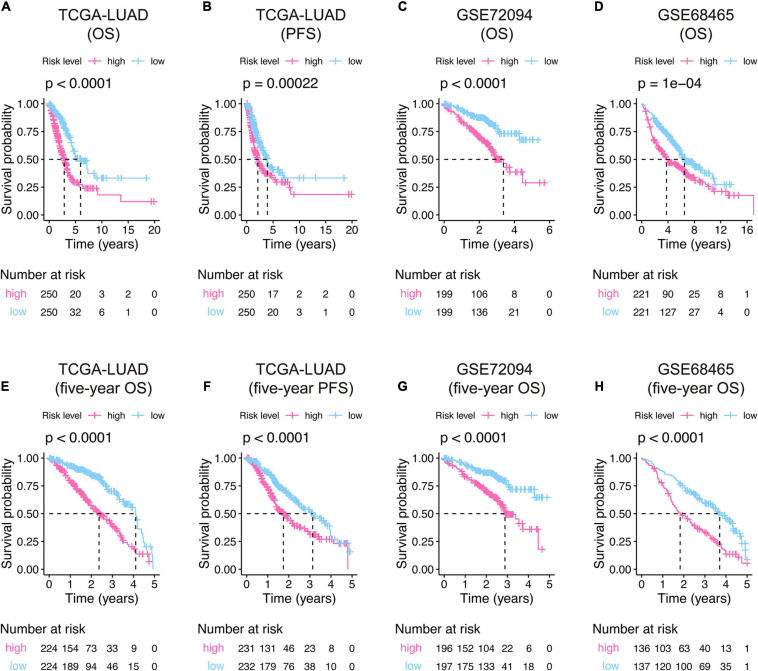
Kaplan–Meier curves of the 15-gene signature risk score in the training **(A,B,E,F)** and validation **(C,D,G,H)** cohorts. The middle part of each graph indicates the number of patients at risk. The differences between the high- and low-risk groups were measured by the two-side log-rank test with a *P*-value < 0.05. OS, overall survival; PFS, progression-free survival.

Kaplan–Meier curves displayed that the high-risk groups have poor overall survival in TCGA-LUAD (*P*-value < 0.0001, Figure 4A), GSE72094 (*P*-value < 0.0001, Figure 4C), and GSE68465 (*P*-value = 0.0001, Figure 4D), and progression-free survival in TCGA-LUAD (*P*-value = 0.00022, Figure 4B) compared to specific low-risk patients. The Kaplan–Meier curves of 5-year survival showed the same pattern that high-risk score patients owned significant unfavorable outcomes than their corresponding low-risk groups (Figures 4E–H).

Univariate and multivariate Cox analyses were applied in the training and two validation cohorts based on overall survival or progression-free survival, using the available co-variables including risk score, gender, age, race, tumor stage, tobacco smoking history, radiation therapy, KRAS mutation, and EML4-ALK mutation to validate the prognostic capacity and the independence of the 15-gene signature among other clinic-pathologic characteristics (Table 4). In the overall survival-based Cox regression model of the training cohort, both univariate and multivariate results suggested that the 15-gene signature was a powerful player (HR = 3.77, 95% CI = 2.77–5.14, *P*-value = 4.59E-17, and HR = 6.52, 95% CI = 2.74–15.5, *P*-value = 2.20E-05, respectively). Consistent with that in the training cohort, in either univariate or multivariate analysis, the 15-gene signature showed excellent ability in the other two independent validation cohorts in predicting overall survival (*P*-value < = 1.04E-04). Also, we utilized progression-free survival data in the training cohort to perform the Cox analysis and found that the 15-gene signature had the ability not only in univariate but also in multivariate models to predict the outcomes (*P*-value < = 2.84E-03). These evidences demonstrated that the 15-gene signature was to be an independent and strong variable of prognosis.

**TABLE 4:** Univariate analysis and multivariate analysis of the correlation of gene-signature risk score with outcomes among lung adenocarcinoma patients in three cohorts*.

Variable	Univariate Cox analysis				Multivariate Cox analysis			
	coef	HR (95% CI)	z	*P*-value	coef	HR (95% CI)	z	*P*-value
**TCGA-LUAD (overall survival)^**								
Gender (male vs. female)	0.048	1.05 (0.784–1.4)	0.322	7.47E-01				
Age	0.00816	1.01 (0.993–1.02)	1.05	2.94E-01				
Tumor stage II (vs. stage I)	0.905	2.47 (1.72–3.56)	4.87	**1.09E-06**	1.49	4.43 (1.69–11.6)	3.02	**2.49E-03**
Tumor stage III (vs. stage I)	1.25	3.49 (2.38–5.12)	6.4	**1.51E-10**	0.705	2.02 (0.613–6.68)	1.16	2.48E-01
Tumor stage IV (vs. stage I)	1.34	3.82 (2.2–6.62)	4.76	**1.92E-06**	-0.125	0.882 (0.248–3.14)	–0.193	8.47E-01
Tobacco smoking history (ever vs. never)	–0.127	0.88 (0.583–1.33)	–0.605	5.45E-01				
Number pack years smoked	0.00327	1 (0.996–1.01)	0.938	3.48E-01				
Race (white vs. non-white)	0.36	1.43 (0.875–2.34)	1.43	1.53E-01				
Ethnicity (Hispanic or Latino vs. non-Hispanic or Latino)	0.387	1.47 (0.466–4.65)	0.658	5.10E-01				
Tumor intermediate dimension	0.441	1.55 (0.922–2.62)	1.65	9.80E-02				
Radiation therapy (yes vs. no)	0.763	2.14 (1.44–3.19)	3.76	**1.73E-04**	0.397	1.49 (0.451–4.9)	0.653	5.14E-01
Additional pharmaceutical therapy (yes vs. no)	–0.502	0.605 (0.382–0.959)	–2.14	**3.26E-02**	-0.567	0.567 (0.263–1.22)	–1.45	1.48E-01
Additional radiation therapy (yes vs. no)	–0.0153	0.985 (0.625–1.55)	–0.0661	9.47E-01				
Location in lung parenchyma (central lung vs. peripheral lung)	0.0908	1.09 (0.684–1.75)	0.378	7.06E-01				
KRAS mutation (yes vs. no)	0.492	1.63 (0.672–3.98)	1.08	2.78E-01				
EGFR mutation (yes vs. no)	0.268	1.31 (0.828–2.06)	1.15	2.50E-01				
EML4-ALK mutation (yes vs. no)	0.592	1.81 (1.01–3.24)	1.98	**4.73E-02**	1.52	4.55 (1.62–12.8)	2.88	**3.98E-03**
Risk score	1.33	3.77 (2.77–5.14)	8.4	**4.59E-17**	1.87	6.52 (2.74–15.5)	4.24	**2.20E-05**
**TCGA-LUAD (progression-free survival) #**								
Gender (male vs. female)	0.07	1.07 (0.815–1.41)	0.499	6.17E-01				
Age	–0.000998	0.999 (0.985–1.01)	–0.141	8.88E-01				
Tumor stage II (vs. stage I)	0.798	2.22 (1.61–3.07)	4.85	**1.22E-06**	1.18	3.26 (1.88–5.66)	4.22	**2.44E-05**
Tumor stage III (vs. stage I)	0.709	2.03 (1.38–2.99)	3.61	**3.10E-04**	0.866	2.38 (1.2–4.69)	2.49	**1.26E-02**
Tumor stage IV (vs. stage I)	0.849	2.34 (1.32–4.13)	2.93	**3.40E-03**	0.697	2.01 (0.846–4.77)	1.58	1.14E-01
Tobacco smoking history (ever vs. never)	–0.0557	0.946 (0.637–1.4)	–0.276	7.82E-01				
Number pack years smoked	–0.000552	0.999 (0.993–1.01)	–0.165	8.69E-01				
Race (white vs. non-white)	0.0557	1.06 (0.703–1.59)	0.268	7.89E-01				
Ethnicity (Hispanic or Latino vs. non-Hispanic or Latino)	0.291	1.34 (0.495–3.62)	0.574	5.66E-01				
Tumor intermediate dimension	0.328	1.39 (0.84–2.29)	1.28	2.01E-01				
Radiation therapy (yes vs. no)	0.707	2.03 (1.42–2.89)	3.92	**9.01E-05**	0.43	1.54 (0.827–2.86)	1.36	1.74E-01
Additional pharmaceutical therapy (yes vs. no)	0.27	1.31 (0.914–1.88)	1.47	1.41E-01				
Additional radiation therapy (yes vs. no)	0.16	1.17 (0.825–1.67)	0.89	3.73E-01				
Location in lung parenchyma (central lung vs. peripheral lung)	–0.0709	0.932 (0.584–1.49)	–0.298	7.66E-01				
KRAS mutation (yes vs. no)	–0.275	0.759 (0.316–1.82)	–0.616	5.38E-01				
EGFR mutation (yes vs. no)	0.483	1.62 (1.06–2.49)	2.21	**2.71E-02**	0.319	1.38 (0.846–2.24)	1.29	1.98E-01
EML4-ALK mutation (yes vs. no)	0.509	1.66 (0.965–2.87)	1.83	6.72E-02				
Risk score	0.83	2.29 (1.72–3.06)	5.63	**1.79E-08**	0.756	2.13 (1.3–3.5)	2.98	**2.84E-03**
**GSE72094 (overall survival) &**								
Gender (male vs. female)	0.44	1.55 (1.07–2.25)	2.33	**1.98E-02**	0.444	1.56 (1.07–2.28)	2.29	**2.20E-02**
Age	0.00696	1.01 (0.988–1.03)	0.702	4.83E-01				
Race (white vs. non-white)	–0.0694	0.933 (0.38–2.29)	–0.151	8.80E-01				
Ethnicity (Hispanic or Latino vs. non-Hispanic or Latino)	–0.642	0.526 (0.0733–3.78)	–0.638	5.24E-01				
Tobacco smoking history (ever vs. never)	0.314	1.37 (0.597–3.14)	0.741	4.59E-01				
Tumor stage II (vs. stage I)	0.758	2.13 (1.32–3.44)	3.11	**1.85E-03**	0.69	1.99 (1.23–3.23)	2.81	**4.92E-03**
Tumor stage III (vs. stage I)	1.13	3.09 (1.93–4.97)	4.67	**3.00E-06**	1.15	3.14 (1.95–5.07)	4.69	**2.79E-06**
Tumor stage IV (vs. stage I)	1.21	3.35 (1.59–7.06)	3.18	**1.48E-03**	1.22	3.39 (1.6–7.15)	3.2	**1.37E-03**
KRAS mutation (yes vs. no)	0.376	1.46 (1–2.12)	1.97	**4.92E-02**	0.13	1.14 (0.774–1.68)	0.66	5.09E-01
EGFR mutation (yes vs. no)	–1.34	0.262 (0.0965–0.71)	–2.63	**8.49E-03**	-0.822	0.44 (0.158–1.22)	–1.57	1.16E-01
STK11 mutation (yes vs. no)	–0.0393	0.961 (0.58–1.59)	–0.153	8.79E-01				
TP53 mutation (yes vs. no)	0.211	1.23 (0.82–1.86)	1.01	3.13E-01				
Risk score	0.855	2.35 (1.76–3.14)	5.77	**8.07E-09**	0.734	2.08 (1.53–2.84)	4.64	**3.50E-06**
**GSE68465 (overall survival) $**								
Gender (male vs. female)	0.33	1.39 (1.07–1.8)	2.5	**1.25E-02**	0.271	1.31 (0.991–1.74)	1.9	5.78E-02
Age	0.0266	1.03 (1.01–1.04)	3.97	**7.16E-05**	0.035	1.04 (1.02–1.05)	4.84	**1.28E-06**
Race (white vs. non-white)	0.32	1.38 (0.702–2.71)	0.931	3.52E-01				
Chemotherapy (yes vs. no)	0.488	1.63 (1.22–2.18)	3.29	**9.99E-04**	0.222	1.25 (0.84–1.86)	1.1	2.72E-01
Radiation therapy (yes vs. no)	0.698	2.01 (1.47–2.75)	4.37	**1.26E-05**	0.274	1.31 (0.848–2.04)	1.22	2.21E-01
T classification (T2 vs. T1)	0.381	1.46 (1.09–1.97)	2.52	**1.18E-02**	0.145	1.16 (0.846–1.58)	0.912	3.62E-01
T classification (T3 vs. T1)	1.2	3.31 (2.06–5.34)	4.92	**8.78E-07**	0.758	2.13 (1.29–3.53)	2.95	**3.22E-03**
T classification (T4 vs. T1)	1.45	4.26 (2.17–8.34)	4.22	**2.41E-05**	1.04	2.82 (1.33–5.98)	2.7	**6.92E-03**
N classification (N1 vs. N0)	0.821	2.27 (1.67–3.09)	5.23	**1.74E-07**	0.773	2.17 (1.57–3)	4.67	**3.02E-06**
N classification (N2 vs. N0)	1.36	3.9 (2.77–5.48)	7.82	**5.47E-15**	1.23	3.41 (2.31–5.02)	6.19	**5.97E-10**
Tobacco smoking history (ever vs. never)	0.22	1.25 (0.79–1.96)	0.946	3.44E-01				
Risk score	0.452	1.57 (1.29–1.92)	4.43	**9.27E-06**	0.44	1.55 (1.24–1.94)	3.88	**1.04E-04**

*Only variables identified having prognostic capacity in the univariate Cox analysis were included in the multivariate Cox analysis;

*^Concordance = 0.762 (se = 0.045), likelihood ratio test = 32.74 on 7 df, *P* = 3e-05, Wald test = 29.51 on 7 df, *P* = 1e-04, score (log-rank) test = 32.24 on 7 df, *P* = 4e-05; #Concordance = 0.725 (se = 0.031), likelihood ratio test = 44.91 on 6 df, *P* = 5e-08, Wald test = 42.61 on 6 df, *P* = 1e-07, score (log-rank) test = 48.02 on 6 df, *P* = 1e-08; &Concordance = 0.727 (se = 0.024), likelihood ratio test = 65.11 on 7 df, *P* = 1e-11, Wald test = 65.31 on 7 df, *P* = 1e-11, score (log-rank) test = 69.38 on 7 df, *P* = 2e-12; $ Concordance = 0.717 (se = 0.018), Likelihood ratio test = 125 on 10 df, *P* = < 2e-16, Wald test = 130 on 10 df, *P* = < 2e-16, score (log-rank) test = 143.4 on 10 df, *P* = < 2e-16;*

**P*-value in bold indicates < 0.05.*

**FIGURE 5:**
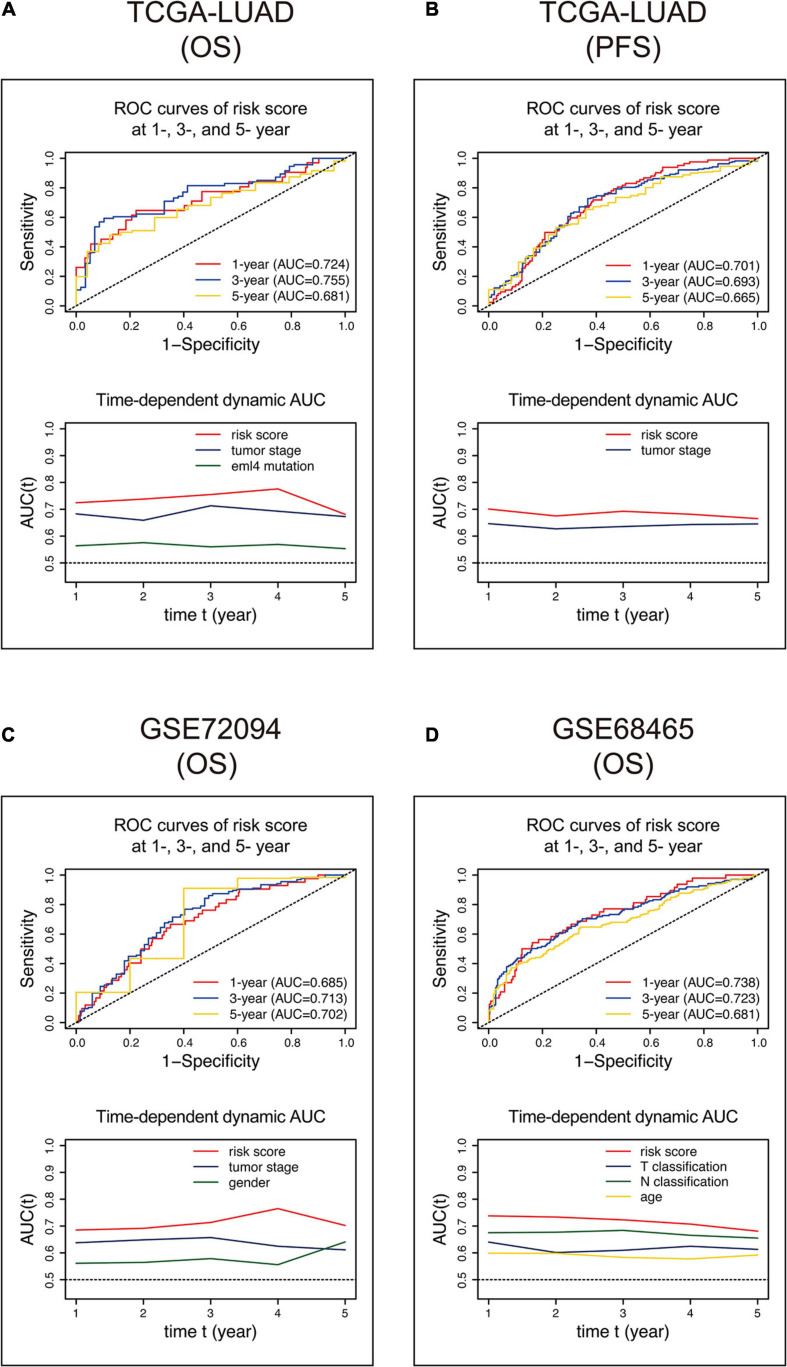
ROC and time-dependent dynamic AUC curves of the 15-gene signature risk score in the training **(A,B)** and validation **(C,D)** cohorts. The ROC curve shows prognostic abilities with risk scores at 1, 3, and 5 years. The time-dependent dynamic AUC curve shows a comparison between the risk score and other independent factors. ROC, receiver operating characteristic; AUC, area under the ROC curve.

We built ROC curves and time-dependent dynamic AUC comparisons with other variates that showed an independent prognostic value in the multivariate Cox analysis in Table 4 to evaluate how the 15-gene signature could behave in predicting outcome and whether risk score is superior to other variates. As shown in Figure 5A, the AUCs of the 15-gene risk score model performed in the training cohort were 0.724, 0.755, and 0.681 at 1, 3, and 5 years, respectively, and the time-dependent dynamic AUC plot exhibited that the risk score displayed the best performance among the independent variates at all time points within the 5-year period. Consistently, the AUCs of the risk score in the GSE72094 (Figure 5C) and GSE68465 (Figure 5D) cohorts were > = 0.681 at 1, 3, and 5 years and were greater than other vital variates at any time within 5 years. Besides, we used progression-free survival data in the training cohort to evaluate the predicting ability of risk score and found a similar pattern that in the other three cohorts, risk score had the best capacity (Figure 5B).

### Identification of the Autophagy Correlation With the Gene Signature Risk Score

**FIGURE 6:**
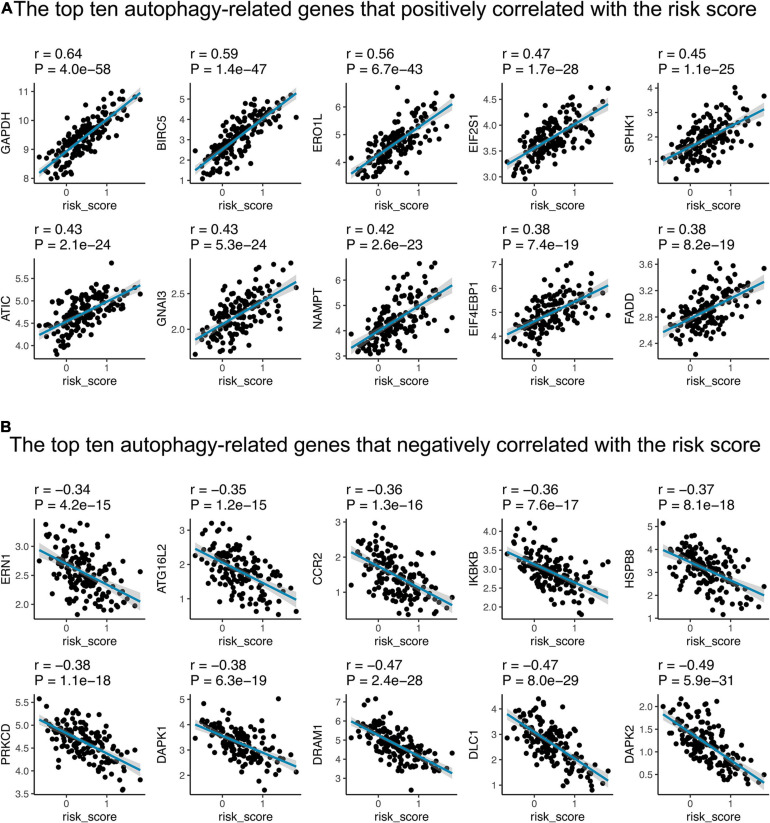
The correlations between the 15-gene signature and autophagy-related genes in the training cohort. The figure shows the top ten autophagy-related genes that positively **(A)** and negatively **(B)** correlated with the risk score, respectively. The blue line in each graph fits a linear model that indicates the proportional trend of expression level of each gene and the risk score. The shading around the blue line represents the 95% confidence interval. The Pearson coefficient was applied for correlation test.

Moreover, we conducted Pearson correlation to evaluate the relationship between autophagy-related genes and the 15-gene signature risk score. Of the 222 autophagy-related genes, 151 (68.01%) were significantly correlated with risk scores, of which 74 were positively correlated and 77 were negatively correlated (Supplementary Table 5). As shown in Figure 6, GAPDH, BIRC5, ERO1L, EIF2S1, SPHK1, ATIC, GNAI3, NAMPT, EIF4EBP1, and FADD are the top 10 autophagy-related genes that positively corrected with the risk score; 8/10 showed a significant elevated hazard ratio in LUAD (Supplementary Figure 2A). ERN1, ATG16L2, CCR2, IKBKB, HSPB8, PRKCD, DAPK1, DRAM1, DLC1, and DAPK2 are the leading 10 that have negative relationships with the 15-gene signature risk score; three of them exhibited a decreased hazard ratio (Supplementary Figure 2B).

### GSEA With the 15-Gene Signature

**FIGURE 7:**
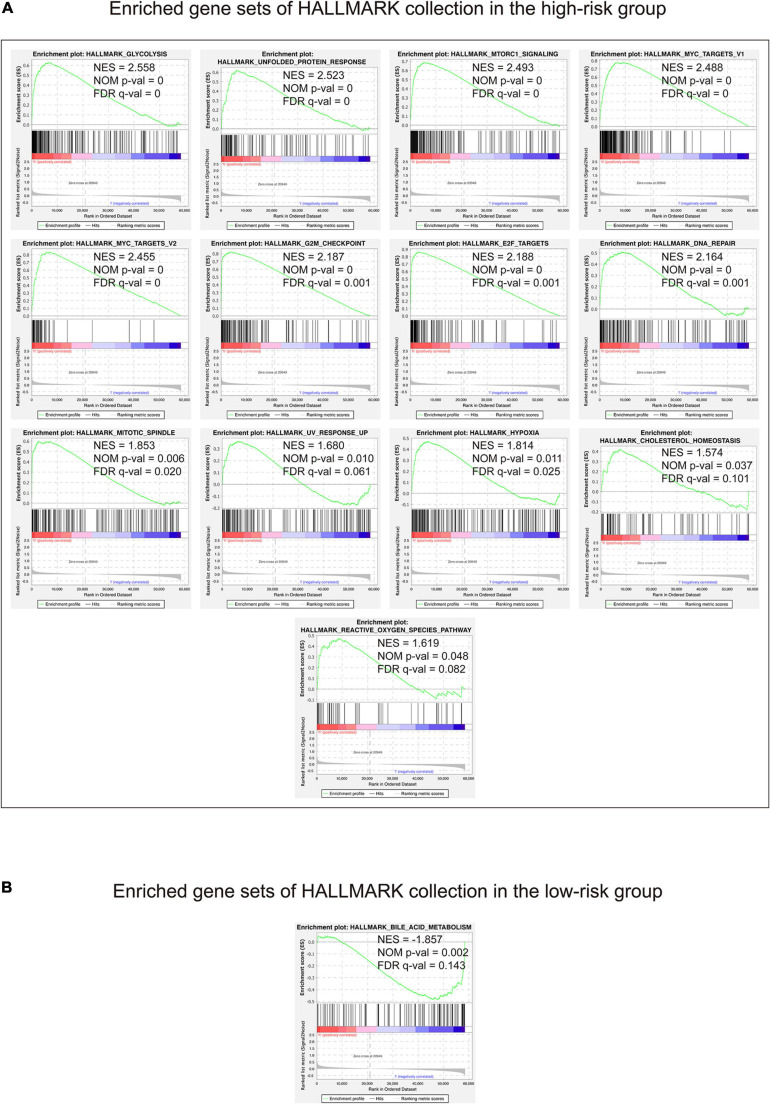
Gene set enrichment analysis performed using HALLMARK collection. The figure shows the enriched gene sets in the high-risk **(A)** and low-risk **(B)** groups, respectively. Gene sets with | NES | > 1, NOM *p*-value <0.05, and FDR *q*-value < 0.25 were considered significantly enriched. H, high-risk group; l, low-risk group.

In view of the negative correlation between the 15-gene signature risk score level and the LUAD outcomes, GSEA was performed between the high-risk and low-risk groups. As displayed in Figure 7A and Supplementary Table 6, enriched gene sets of HALLMARK collection in the high-risk group were mainly involved in pathways related to glycolysis, unfolded protein response, mTORC1, MYC, G2/M checkpoint, E2F, DNA repair, mitotic spindle assembly, ultraviolet radiation, hypoxia, cholesterol homeostasis, and reactive oxygen species, whereas the gene set concerned with metabolism of bile acids and salts was primary enriched in the low-risk group (Figure 7B and Supplementary Table 6).

### Identification of the Relationship Between the Fifteen-Gene Signature and 22 TICs

To better study how the 15-gene signature and the immune microenvironment interact, we used the CIBERSORT algorithm to evaluate the proportion of tumor-infiltrating immune subpopulations and made comprehensive comparisons with the risk score. The relative content distribution of 22 TICs in the TCGA-LUAD cohort and the correlation between 22 TICs are shown in Supplementary Figure 3.

**FIGURE 8:**
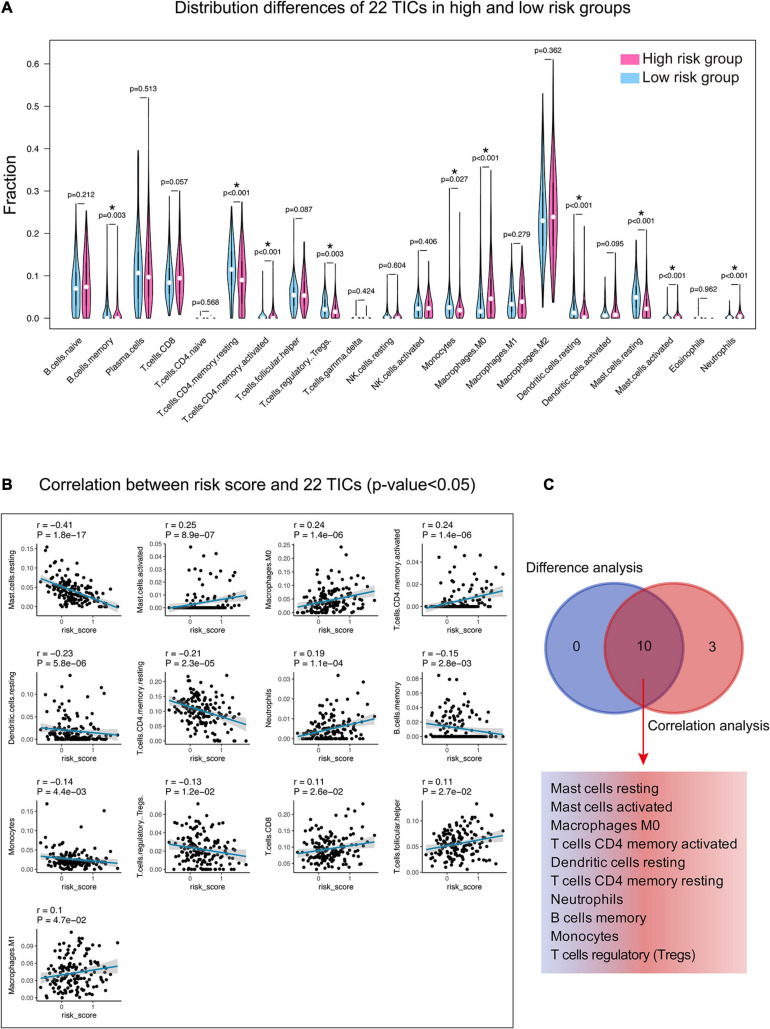
Relationship between TICs and 15-gene signature risk score. **(A)** The Violin plot shows the ratio differentiation of each of 22 TICs between the high- and low-risk groups. Wilcoxon rank sum was applied for the significance test. **(B)** The correlations between the TICs and 15-gene signature risk score (only correlations with significance were plotted). The blue line in each graph fits a linear model that indicates the proportional trend of the TICs and the risk score. The shading around the blue line represents the 95% confidence interval. The Spearman coefficient was applied for the correlation test. **(C)** The Venn diagram shows that 10 TICs have a pronounced correlation with the risk score, which is determined by the results of the violin and the scatter plot. *P*-value < 0.05 is the cutoff. TIC, tumor-infiltrating immune cell. *, *P*-value < 0.05.

Incorporating the results of difference analysis (Figure 8A) and correlation analysis (Figure 8B and Supplementary Table 7), 10 TICs, including resting mast cells, activated mast cells, M0 macrophages, activated CD4 memory T cells, resting dendritic cells, resting memory CD4 T cells, neutrophils, B cell memory, monocytes, and regulatory T cells (Tregs), were identified associating with the 15-gene signature risk score (Figure 8C). Among them, mast cells activated, macrophages M0, and T cells CD4 memory activated were positively correlated with risk score, the remaining negatively correlated.

**FIGURE 9:**
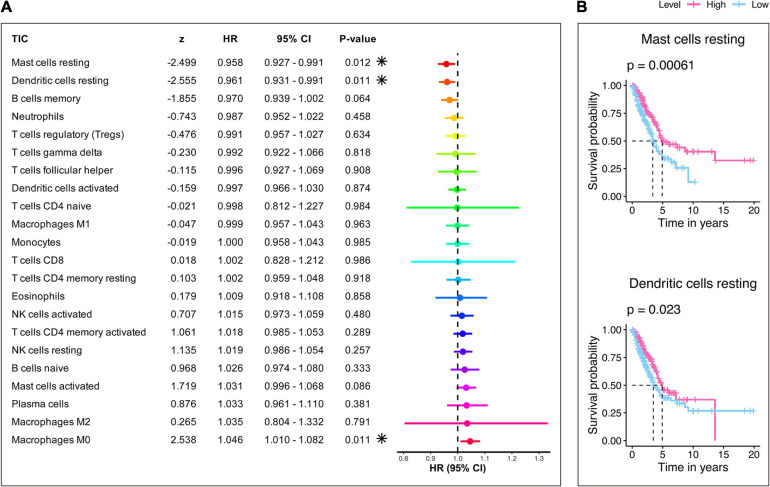
Evaluation of the prognostic ability of 22 TICs. **(A)** Univariate Cox regression model built for 22 TICs based on overall survival. Asterisk shown in the B plot indicates *P*-value < 0.05. **(B)** Kaplan–Meier survival curves. Only graphs with *P*-value < 0.05 in the log-rank test were plotted.

Furthermore, the prognostic capacity of each TIC was examined using univariate Cox and Kaplan–Meier analyses. As shown in Figure 9, the univariate Cox regression model (Figure 9A) highlighted that resting mast cells, resting dendritic cells, and M0 macrophages impacted the prognosis; besides, Kaplan–Meier analysis (Figure 9B and Supplementary Table 8) indicated that resting mast cells and resting dendritic cells can predict the survival of LUAD. It can be seen that resting mast cells and resting dendritic cells showed significance in both analyses and may have potential prognostic ability in LUAD. Also, resting mast cells and resting dendritic cells not only had prognostic value but also owned significant correlations with the risk score, as mentioned earlier. Therefore, the significant infiltration with resting mast cells and resting dendritic cells may play a vital role in contributing to the prognosis value of the 15-gene signature in LUAD.

## Discussion

In this study, we built a ferroptosis-related 15-gene signature for the prognosis of LUAD by comprehensively mining the TCGA and GEO databases. After discovering the potential ferroptosis-related prognosis genes using Kaplan–Meier and univariate Cox analyses in the TCGA-LUAD cohort, the LASSO Cox regression model was applied, and a 15-gene signature was generated which was related to outcome of LUAD. By applying the 15-gene signature in the training and validation cohorts, pronounced statistical differences were seen in Kaplan–Meier analysis, univariate and multivariate Cox regression models, and ROC curves, demonstrating the effectiveness and broadness of the gene signature in predicting LUAD prognosis. In the following correlation analysis, the 15-gene signature was found correlated with most autophagy-related genes. The GSEA and analysis of immune infiltration exhibited important pathways that relate to the 15-gene signature and vital roles that resting mast cells and resting dendritic cells may have played backing the 15-gene signature influencing the outcome of LUAD. Compared with previous studies discovering the prognostic gene signature in LUAD, we are the first to utilize ferroptosis-related genes for training and validation in two independent cohorts (more than 400 cases each). This work we have done aimed to present more hints in future LUAD research.

The ferroptosis-related 15-gene signature that we discovered showed strong prognostic prediction capabilities not only in the training cohort but also in the two independent validation cohorts after being examined by a variety of statistical methods. The signature was composed of 15 genes (Table 3), which were RELA, ACSL3, YWHAE, EIF2S1, CISD1, DDIT4, RRM2, PANX1, TLR4, ARNTL, LPIN1, HERPUD1, NCOA4, PEBP1, and GLS2, respectively. In our research, RELA, ACSL3, YWHAE, EIF2S1, CISD1, DDIT4, RRM2, and PANX1 show unfavorable impacts on LUAD prognosis, while other genes displayed protective effects on the outcome. Interestingly, in the gene signature, 83.3% (5/6) ferroptosis markers and 66.7% (2/3) ferroptosis suppressors predicted bad prognosis, while 83.3% (5/6) ferroptosis drivers indicated favor outcomes. RELA activation has been found to be correlated with cancer development, suggesting the potential of RELA as a cancer biomarker (Ali et al., 2017; Onishi et al., 2018; Ahmed et al., 2019; Vlahopoulos et al., 2019). RELA is implicated in tumor–stroma interactions and correlates strongly with the severity of tumor infiltration by inflammatory cells in NSCLC patients (Giopanou et al., 2015). ACSL3 activity was previously demonstrated specifically to promote a ferroptosis-resistant cell state (Magtanong et al., 2019). ACSL3 overexpression increased cell proliferation, migration, and invasion altering the metabolic properties of lung cancer cells and was associated with worse clinical outcomes in patients with high-grade NSCLC (Fernandez et al., 2020). YWHAE was upregulated in breast cancer (Yang et al., 2019), high-grade endometrial stromal sarcoma (Hemming et al., 2017), gastric cancer (Leal et al., 2016), and colorectal cancer (Bjeije et al., 2019) and associated with poor outcomes. However, the role of YWHAE in LUAD has not been adequately evaluated. The interaction between TIPRL and EIF2S1 leads to phosphorylation of EIF2S1 and activation of the EIF2S1-ATF4 pathway, thereby inducing autophagy and playing a role in affecting the prognosis of LUAD (Jeon et al., 2019). CISD1 inhibits ferroptosis by protection against mitochondrial lipid peroxidation (Yuan et al., 2016). A recent study revealed that the NEET protein CISD1 plays a critical role in promoting the proliferation of cancer cells, supporting tumor growth and metastasis (Mittler et al., 2019). The function of CISD1 in cancer cells was found to be dependent on the degree of lability of their 2Fe-2S clusters (Mittler et al., 2019), whereas the mechanism of CISD1 in LUAD remained unclear. In multiple malignancies, studies have shown that DDIT4 participates in tumorigenesis and impacts patient survival (Cheng et al., 2020). Mu et al. (2019) showed that inhibition of SIRT1/2 induces pro-survival autophagy *via* acetylation of HSPA5 and subsequent activation of ATF4 and DDIT4 to inhibit the mTOR signaling pathway in NSCLC cells. It is reported that RRM2 is involved in the progression of various cancers, including glioma, colorectal cancer, bladder cancer, and NSCLC (Huang et al., 2019). In a recent study, Su provided important evidence that PANX1 plays an important role in ferroptotic cell death (Su et al., 2019). In addition, Jalaleddine identified a role for PANX1 in breast cancer progression and that PANX1 mediates this tumor-promoting role by modification of the EMT pathway (Jalaleddine et al., 2019). However, the impact of PANX1 on LUAD and its prognosis remained unknown. The expression of TLR4 is highly observed in the cells of the immune system such as monocytes, lymphocytes, and splenocytes, but it is also expressed in lower levels in epithelial and endothelial cells as well as cancer cells (Shetab Boushehri and Lamprecht, 2018). TLR4 agonists have thus been, and are still being, wildly explored as highly potentially immunotherapeutic for the treatment of cancer (Shetab Boushehri and Lamprecht, 2018). Previous study has shown that ARNTL played a role in tumor suppression (Liu et al., 2019). Nevertheless, few studies have found that ARNTL has tumor promotion properties (Liu et al., 2019). LPIN1 plays an important role in cell proliferation and tumor development through the regulation of intracellular signaling pathways. However, the underlying molecular mechanisms and the specific signaling pathway of LPIN1 during tumor development remains to be elucidated (Kim et al., 2016). HERPUD1 protects against oxidative stress-induced apoptosis through downregulation of the inositol 1,4,5-trisphosphate receptor (Paredes et al., 2016). However, little is known about the links between HERPUD1 and cancers. Tang’s research shows that overexpression of NCOA4 increases ferritin degradation and promotes ferroptosis (Hou et al., 2016). However, how NCOA4 affects the occurrence and progression of tumors is still largely unknown (Hou et al., 2016). It has been well-established that PEBP1 suppresses the metastatic spread of tumor cells; moreover, the downregulated expression of PEBP1 is observed in a number of human cancers (Xu et al., 2010). According to one recent study, downregulation of PEBP1 is associated with poor prognosis of hepatocellular carcinoma, while the relationship between PEBP1 and LUAD is still unknown (Xu et al., 2010). The GLS2 gene is a transcriptional target of p53, and in glioblastoma and liver cancer GLS2 has been described as a tumor suppressor (Lukey et al., 2019). The role of GLS2 in LUAD needs to be studied.

Autophagy is the natural, regulated mechanism of the cell that removes unnecessary or dysfunctional components. It allows the orderly degradation and recycling of cellular components (Mizushima and Komatsu, 2011). The original study shows that ferroptosis is morphologically, biochemically, and genetically distinct from autophagy and other types of cell death (Kang and Tang, 2017). However, recent studies demonstrate that activation of ferroptosis is indeed dependent on the induction of autophagy (Kang and Tang, 2017). In addition, accumulating studies have revealed cross talk between autophagy and ferroptosis at the molecular level (Zhou et al., 2019). Autophagy has been shown to exert pleiotropy, exhibiting anti-carcinogenic, pro-survival, and pro-apoptotic effects in different stages of lung cancers (Ryter and Choi, 2015). Recent efforts to develop lung cancer treatment strategies have focused on understanding the role of autophagy as a tumor-suppressing or tumor-promoting mechanism (Jeon et al., 2019). In this study, we discovered that the risk score correlated with more than half of the autophagy-related genes (68.01%, 151/222) and found that the top 10 correlated genes hold the same effect predicting the LUAD outcomes, which elaborated further the relation between the ferroptosis-related 15-gene signature and LUAD and also provided more possibilities for autophagy-targeted strategies.

The GSEA in HALLMARK collection found that gene sets about glycolysis, unfolded protein response, and mTORC1 were top enriched. Changes in energy metabolism are the biochemical fingerprints of cancer cells and represent one of the “hallmarks of cancer.” This metabolic phenotype is characterized by preferentially relying on glycolysis to produce energy in an oxygen-independent manner (Ganapathy-Kanniappan and Geschwind, 2013; Liberti and Locasale, 2016). Tumor cells (including lung cancer cells) ingesting a large amount of glucose will hinder the supply of nutrients to adjacent normal cells (Sotgia et al., 2011; Chang et al., 2020). Glycolysis can also induce deoxyribonucleic acid (DNA) mutations and peroxide production, both of which are conducive to the proliferation and metastasis of tumor cells (Sotgia et al., 2011; Chang et al., 2020). The unfolded protein response is a cellular stress response related to the endoplasmic reticulum stress (Madden et al., 2019). The internal pressure in the tumor (such as oncogenic activation) and the external pressure exerted by the tumor environment can increase the level of misfolded proteins in the endoplasmic reticulum, which triggers the activation of the unfolded protein response pathway (Madden et al., 2019). The tumor environment is an environment of hypoxia, acidity, and nutritional deficiency. The three arms of unfolded protein response are highly active in many types of cancers (including breast, lung, liver, and colorectal and glioma) (Madden et al., 2019). mTOR is a pathway found to be dysregulated in many disease states including cancer (lung cancer) (Ekman et al., 2012; Kim et al., 2017). Studies have shown that mTOR signal inhibition blocks tumor cell progression, disrupts angiogenesis, and induces apoptosis and autophagy (Ekman et al., 2012; Kim et al., 2017). Growth factors, stress, amino acids, energy, and oxygen give inputs to mTOR which activate the mTORC1 (Vicary and Roman, 2016; Murugan, 2019). Activated mTORC1 regulates stress/DNA damage and enhances nucleotide synthesis, protein synthesis, and metabolism that promote cell proliferation, cell survival metastasis, etc. (Vicary and Roman, 2016; Murugan, 2019). Targeting of mTOR is thus an attractive strategy in the development of therapeutic agents against lung cancer (Ekman et al., 2012). These GSEA results described in detail the ways and methods that the 15-gene signature participates in the progress of LUAD, which can benefit future targeted therapy research.

Moreover, the CIBERSORT algorithm-based TIC analysis discovered that resting mast cells and resting dendritic cells have strong prognostic capacity in LUAD and a significant correlation with the ferroptosis-related 15-gene signature risk score, revealing that the infiltration of resting mast cells and resting dendritic cells may play key roles affecting the prognostic ability of the 15-gene signature. Mast cells can be used as an important innate immune sentinel and have the ability to enhance the immune response mediated by T cells but in other cases also show the ability to suppress the immune response (Dudeck et al., 2019; Kaesler et al., 2019). Consistent with their functional plasticity, the number of mast cells in TME is reported to be associated with cancer progression and improvement in patient survival (Kaesler et al., 2019). In NSCLC, mast cell infiltration of tumor islets confers a survival advantage independently of tumor stage (Varricchi et al., 2017). In another study, it was found that only in stage I NSCLC were increased peritumoral mast cells associated with a better prognosis (Varricchi et al., 2017). Dendritic cells represent a heterogeneous group of innate immune cells that infiltrate and process tumors and present tumor-derived antigens to naive T cells (Wylie et al., 2019). Dendritic cells play a key role in triggering antitumor T cell immunity and therefore represent the main therapeutic target of cancer immunotherapy (Wylie et al., 2019). Dendritic cells are a key factor in providing protective immunity against lung tumors (Wang J.B. et al., 2019). Clinical trials have proved that the DC function of lung cancer patients is reduced (Wang J.B. et al., 2019). Based on our research, resting mast cells and resting dendritic cells have the potential to target the 15-gene signature for the treatment means of LUAD, and good efforts should be carried out to investigate these immune cells further.

## Conclusion

Our study found a novel robust ferroptosis-related 15-gene signature for LUAD. The signature is strongly associated with the prognosis of LUAD and can precisely detect the LUAD risk level. Remarkably, we validated the reliability and applicability of this signature by applying two independent validation cohorts and identified the vital role resting mast cells and resting dendritic cells may interplay in the prognostic capacity of the gene signature, which could potentially advance the discovery of new treatments for LUAD.

## Data Availability Statement

The datasets presented in this study can be found in online repositories. The names of the repository/repositories and accession number (s) can be found below: https://portal.gdc.cancer.gov/, TCGA-LUAD; https://www.ncbi.nlm.nih.gov/geo/, GSE72094; https://www.ncbi.nlm.nih.gov/geo/, GSE68465.

## Author Contributions

AZ, JY, and CM organized and wrote the manuscript. FL and HL produced the figures and visualized the data. CM revised the manuscript. All authors reviewed the manuscript and approved the manuscript for publication.

## Conflict of Interest

The authors declare that the research was conducted in the absence of any commercial or financial relationships that could be construed as a potential conflict of interest.

## Footnotes

1 https://gdc.xenahubs.net

2 http://www.zhounan.org/ferrdb/index.html

3 http://www.autophagy.lu/index.html

4 http://gepia2.cancer-pku.cn

5 https://www.gsea-msigdb.org/gsea/downloads.jsp

6 https://www.gsea-msigdb.org/

## Supplementary Material

Supplementary Figure 1 Fifteen gene expression distributions in the high- and low-risk groups in the training **(A)** and validation **(B,C)** cohorts. The box plot shows the expression differentiation of each of the fifteen gene between the high- and low-risk groups. Wilcoxon rank-sum was applied for the significance test. *P*-value < 0.05 was considered statistically significant; ns: *P*-value > 0.05; ^∗^*P*-value < 0.05; ^∗∗^*P*-value < 0.01; ^∗∗∗^*P*-value < 0.001.

Supplementary Figure 2 Survival heatmaps of the top 10 autophagy-related genes positively **(A)** and negatively **(B)** correlated with risk score in LUAD (GEPIA2 web patrol, http://gepia2.cancer-pku.cn/). The survival heatmaps show the hazard ratios in the logarithmic scale (log10) for different genes. The red and blue blocks denote higher and lower risks, respectively. The rectangles with frames mean the significant unfavorable and favorable results in prognostic analyses. LUAD, lung adenocarcinoma.

Supplementary Figure 3 TIC distribution map and correlation analysis of tumor samples in the training cohort. **(A)** The bar graph shows the relative content distribution of 22 TICs in the LUAD samples in the training cohort. Each column represents a case. **(B)** The heatmap shows the correlation between 22 TICs. The color and number in each box indicate the coefficient between the two TICs. The coefficient of X-shaped coverage is not significant. The Pearson coefficient is used for correlation testing. *P*-value < 0.05 is the cutoff. LUAD, lung adenocarcinoma; TIC, tumor-infiltrating immune cell.

Supplementary Table 1 Genes of drivers, suppressors, and markers of ferroptosis.

Supplementary Table 2 259 genes were identified as the ferroptosis-related genes.

Supplementary Table 3 222 autophagy-associated genes identified from the Human Autophagy Database.

Supplementary Table 4 4138 genes were significantly predicting the prognosis of LUAD patients by both Kaplan-Meier and univariate Cox regression analyses (*P*-value < 0.05).

Supplementary Table 5 The correlations of 222 autophagy-related genes with the fifteen-gene signature risk score examined by the Pearson correlation coefficient.

Supplementary Table 6 Enriched gene sets in HALLMARK collection in high- and low-risk groups (| NES | > 1, NOM *P*-value < 0.05, and FDR *q*-value < 0.25).

Supplementary Table 7 Correlations of risk score with 22 kinds of TICs tested by Spearman coefficient.

Supplementary Table 8 Prognostic capacity of 22 TICs examined by Kaplan–Meier analysis.
