# Supplementary material for: Development and Validation of a Robust Ferroptosis-Related Prognostic Signature in Lung Adenocarcinoma
Source: Front Cell Dev Biol. 2021 Jun 24;9:616271. doi: 10.3389/fcell.2021.616271 (PMC8264775; doi:10.3389/fcell.2021.616271)
Supplement: Supplementary Figure 1 — Fifteen gene expression distributions in the high- and low-risk groups in the training (A) and validation (B,C) cohorts. The box plot shows the expression differentiation of each of the fifteen gene between the high- and low-risk groups. Wilcoxon rank-sum was applied for the significance test. P-value < 0.05 was considered statistically significant; ns: P-value > 0.05; ∗P-value < 0.05; ∗∗P-value < 0.01; ∗∗∗P-value < 0.001. [file Data_Sheet_1.PDF]

Figure S1

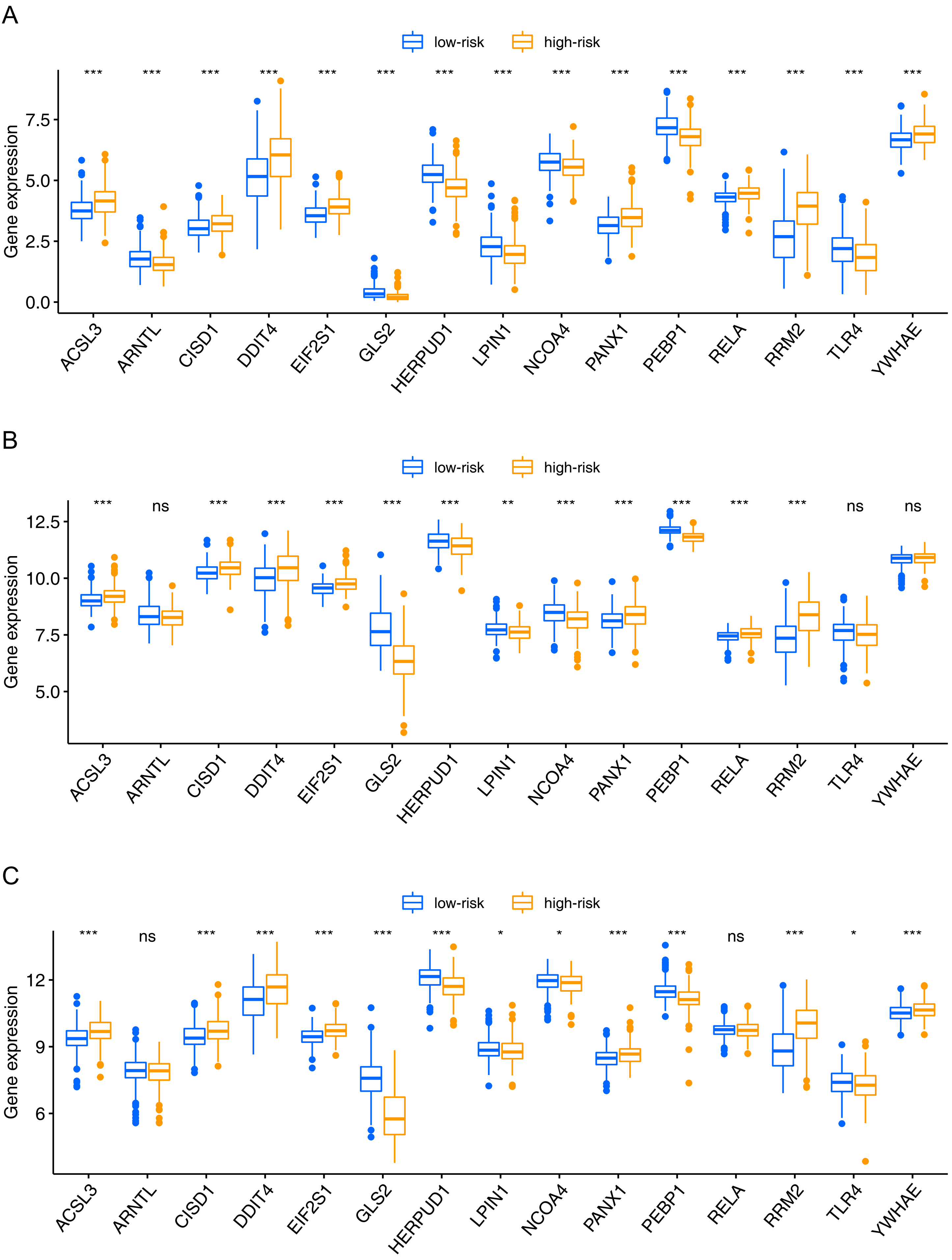
A
B
C
low-risk
high-risk
Gene expression
ACSL3
ARNTL
CISD1
DDIT4
EIF2S1
GLS2
HERPUD1
LPIN1
NCOA4
PANX1
PEBP1
RELA
RRM2
TLR4
YWHAE
***
**
*
ns
0.0
2.5
5.0
7.5
10.0
12.5
6
9
12

Figure S2

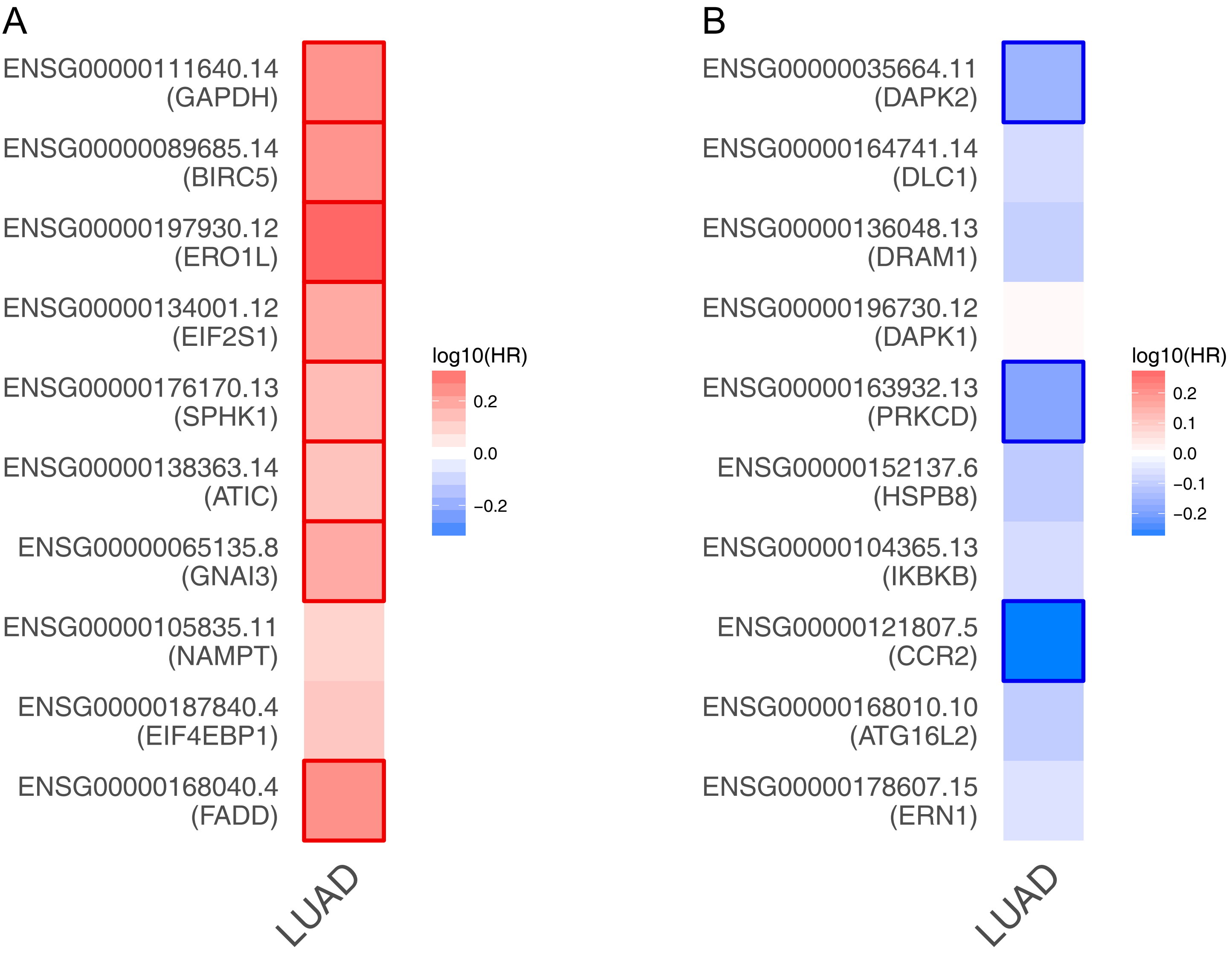
A
ENSG00000111640.14 (GAPDH)
ENSG00000089685.14 (BIRC5)
ENSG00000197930.12 (ERO1L)
ENSG00000134001.12 (EIF2S1)
ENSG00000176170.13 (SPHK1)
ENSG00000138363.14 (ATIC)
ENSG00000065135.8 (GNAI3)
ENSG00000105835.11 (NAMPT)
ENSG00000187840.4 (EIF4EBP1)
ENSG00000168040.4 (FADD)
LUAD
log10(HR)
0.2
0.0
−0.2
B
ENSG00000035664.11 (DAPK2)
ENSG00000164741.14 (DLC1)
ENSG00000136048.13 (DRAM1)
ENSG00000196730.12 (DAPK1)
ENSG00000163932.13 (PRKCD)
ENSG00000152137.6 (HSPB8)
ENSG00000104365.13 (IKBKB)
ENSG00000121807.5 (CCR2)
ENSG00000168010.10 (ATG16L2)
ENSG00000178607.15 (ERN1)
LUAD
log10(HR)
0.2
0.1
0.0
−0.1
−0.2

Figure S3

A

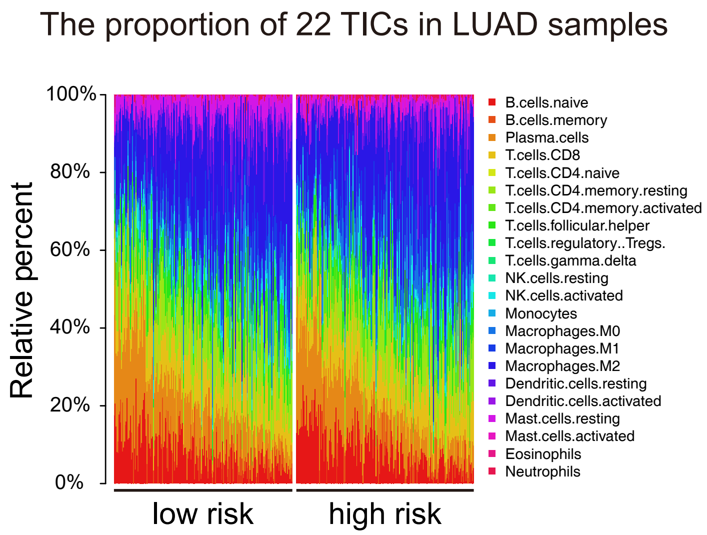

The proportion of 22 TICs in LUAD samples
Relative percent
100%
80%
60%
40%
20%
0%
low risk
high risk
B.cells.naive
B.cells.memory
Plasma.cells
T.cells.CD8
T.cells.CD4.naive
T.cells.CD4.memory.resting
T.cells.CD4.memory.activated
T.cells.follicular.helper
T.cells.regulatory..Tregs.
T.cells.gamma.delta
NK.cells.resting
NK.cells.activated
Monocytes
Macrophages.M0
Macrophages.M1
Macrophages.M2
Dendritic.cells.resting
Dendritic.cells.activated
Mast.cells.resting
Mast.cells.activated
Eosinophils
Neutrophils

B

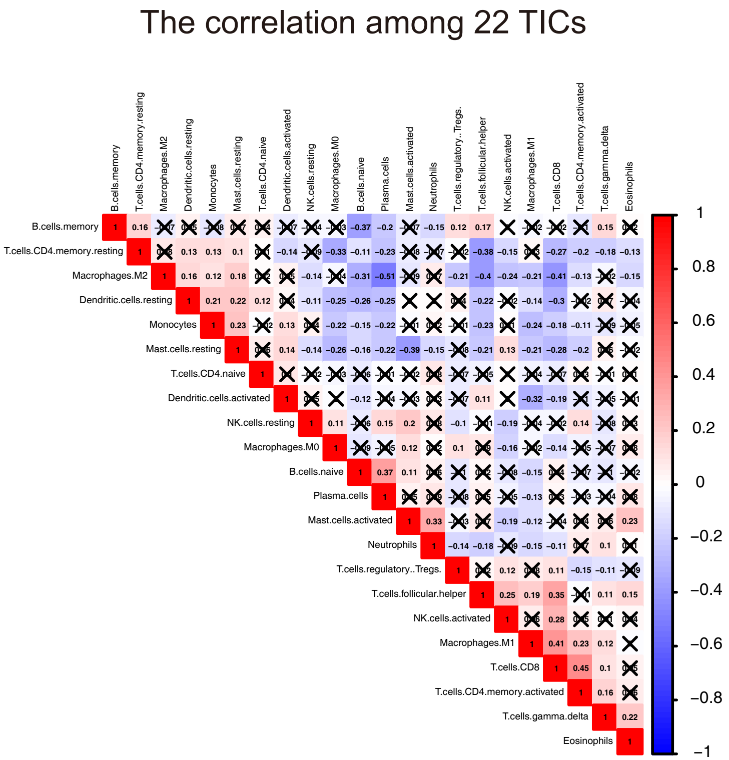

The correlation among 22 TICs
B.cells.memory
T.cells.CD4.memory.resting
Macrophages.M2
Dendritic.cells.resting
Monocytes
Mast.cells.resting
T.cells.CD4.naive
Dendritic.cells.activated
NK.cells.resting
Macrophages.M0
B.cells.naive
Plasma.cells
Mast.cells.activated
Neutrophils
T.cells.regulatory..Tregs.
T.cells.follicular.helper
NK.cells.activated
Macrophages.M1
T.cells.CD8
T.cells.CD4.memory.activated
T.cells.gamma.delta
Eosinophils
1
0.8
0.6
0.4
0.2
0
−0.2
−0.4
−0.6
−0.8
−1

Table S1. Genes of drivers, suppressors, and markers of ferroptosis.

Gene Symbol	Description	Evidence	Confidence	PMID
**Drivers which are genes that promote ferroptosis**				
RPL8	Ribosomal protein L8	Required in erastin-induced ferroptosis. Silencing of it conferred against erastin-induced ferroptosis.	Validated	22632970
IREB2	Iron response element binding protein 2	Required in erastin-induced ferroptosis. Silencing of it conferred against erastin-induced ferroptosis.	Validated	22632970
ATP5MC3	ATP synthase membrane subunit c locus 3	Required in erastin-induced ferroptosis. Silencing of it conferred against erastin-induced ferroptosis.	Validated	22632970
CS	Citrate synthase	Required in erastin-induced ferroptosis. Silencing of it conferred against erastin-induced ferroptosis.	Validated	22632970
EMC2	ER membrane protein complex subunit 2	Required in erastin-induced ferroptosis. Silencing of it conferred against erastin-induced ferroptosis.	Validated	22632970
ACSF2	Acyl-CoA synthetase family member 2	Required in erastin-induced ferroptosis. Silencing of it conferred against erastin-induced ferroptosis.	Validated	22632970
NOX1	Nicotinamide adenine dinucleotide phosphate (NADPH) oxidase (NOX) 1	Suppresses erastin-induced ferroptosis when inhibited by inhibitor.	Deduced	22632970
CYBB	Cytochrome b-245 beta chain	Suppresses erastin-induced ferroptosis when inhibited by inhibitor.	Deduced	22632970
NOX3	Nicotinamide adenine dinucleotide phosphate (NADPH) oxidase (NOX) 3	Suppresses erastin-induced ferroptosis when inhibited by inhibitor.	Deduced	22632970
NOX4	Nicotinamide adenine dinucleotide phosphate (NADPH) oxidase (NOX) 4	Suppresses erastin-induced ferroptosis when inhibited by inhibitor.	Deduced	22632970
NOX5	Nicotinamide adenine dinucleotide phosphate (NADPH) oxidase (NOX) 5	Suppresses erastin-induced ferroptosis when inhibited by inhibitor.	Deduced	22632970
DUOX1	Dual oxidase 1	Suppresses erastin-induced ferroptosis when inhibited by inhibitor.	Deduced	22632970
DUOX2	Dual oxidase 2	Suppresses erastin-induced ferroptosis when inhibited by inhibitor.	Deduced	22632970

G6PD	Glucose-6-phosphate dehydrogenase	Required in erastin-induced ferroptosis.	Validated	22632970
PGD	Phosphoglycerate dehydrogenase	Required in erastin-induced ferroptosis.	Validated	22632970
VDAC2	Valtage-dependent anion channels 2	Required in erastin-induced ferroptosis.	Validated	22632970
PIK3CA	Phosphatidylinositol-4,5-bisphosphate 3-kinase catalytic subunit alpha	Inhibitor blocked ferroptotic cell death.	Validated	24739485
FLT3	Fms related tyrosine kinase 3	Inhibitor blocked ferroptotic cell death.	Validated	24739485
SCP2	Sterol carrier protein 2	SCP-2 inhibitors showed anti-ferroptotic activity, which was substantiated by knocking down SCP-2 in Gpx4−/− cells.	Validated	25402683
TP53	Tumor protein p53	Inhibits cystine uptake and sensitizes cells to ferroptosis. Erastin induced high levels of cell death in p53+/+ MEFs.	Validated	25799988
ACSL4	Acyl-CoA synthetase long chain family member 4	Deletion of this gene likely suppress ferroptosis by limiting the membrane-resident pool of oxidation-sensitive fatty acids.	Predicted	25965523
LPCAT3	Lysophosphatidylcholine acyltransferase 3	Deletion of this gene likely suppress ferroptosis by limiting the membrane-resident pool of oxidation-sensitive fatty acids.	Predicted	25965523
NRAS	NRAS proto-oncogene, GTPase	NRAS12V mutant protects RMS13 cells from ferroptotic cell death.	Deduced	26157704
KRAS	KRAS proto-oncogene, GTPase	KRAS12V mutant protects RMS13 cells from ferroptotic cell death.	Deduced	26157704
HRAS	HRas proto-oncogene, GTPase	HRAS12V mutant protects RMS13 cells from ferroptotic cell death.	Deduced	26157704
TF	Transferrin	Essential for the induction of ferroptotic cell death. Transferrin can only interact with transferrin receptor and be transported into the cell when it is loaded with iron.	Validated	26166707
TFRC	Transferrin receptor	RNAi of transferrin receptor (TfR) inhibited ferroptosis.	Validated	26166707
TFR2	Transferrin receptor 2	RNAi of transferrin receptor (TfR) inhibited ferroptosis.	Validated	26166707

SLC38A1	Solute carrier family 38 member 1	RNAi knockdown markedly blocked ferroptosis.	Validated	26166707
SLC1A5	Solute carrier family 1 member 5	Pharmacological inhibition by L-g-glutamyl-p-nitroanilide or RNAi knockdown markedly blocked ferroptosis.	Validated	26166707
GLS2	Glutaminase 2	Both inhibitor inhibition and gene knockdown inhibit ferroptotic cell death.	Validated	26166707
GOT1	Glutamic-oxaloacetic transaminase 1	RNAi reduced ferroptosis.	Validated	26166707
CARS1	Cysteinyl-tRNA synthetase 1	Required for ferroptosis in diverse cell contexts. Knockdown of CARS inhibited erastin-induced death by preventing the induction of lipid reactive oxygen species, without altering iron homeostasis.	Validated	26184909
TP53	Tumor protein p53	Wild type p53 can induce ferroptosis upon reactive oxygen species (ROS)-induced stress.	Validated	26218928
ALOX5	Arachidonate 5-lipoxygenase	The 5-Lipoxygenase inhibitor zileuton protected HT22 neuronal cells from erastin-induced ferroptosis.	Validated	26235588
KEAP1	Kelch like ECH associated protein 1	Knockdown of Keap1 reversed loss of p62-increased degradation of NRF2 in ferroptosis. Keap1 knockdown led to resistance to erastin-induced and sorafenib-induced growth inhibition with decreased ferroptotic events in the absence or presence of p62 knockdown.	Validated	26403645
HMOX1	Heme oxygenase 1	Zinc protoporphyrin IX, a HO-1 inhibitor, prevented Erastin-triggered ferroptotic cancer cell death. Overexpression accelerates erastin-induced cell death.	Validated	26405158
TP53	Tumor protein p53	p53^3KR/3KR Xrcc4^-/- MEF cells are very sensitive to ferroptosis. Stabilized in the spleens of p53^3KR/3KR Xrcc4^-/- mice which enables to prevent the development of pro-B-cell lymphomas.	Validated	26943586
TP53	Tumor protein p53	Incubation with erastin led to profound cell death in wild-type MEFs.	Validated	27034505
GLS2	Glutaminase 2	Upregulated in erastin-treated wild-type MEFs, and silencing Gls2 exihibits cell death defect in wild-type MEFs treated with erastin.	Validated	27034505

ATG5	Autophagy related 5	Knockout or knockdown limited erastin-induced ferroptosis.	Validated	27245739
ATG7	Autophagy related 7	Knockout or knockdown limited erastin-induced ferroptosis.	Validated	27245739
NCOA4	Nuclear receptor coactivator 4	Inhibition suppressed ferroptosis, and overexpression promoted ferroptosis.	Validated	27245739
TF	Transferrin	Involved in siramesine and lapatinib-induced ferroptotic cell death. Increased following treatment with lapatinib alone or in combination with siramesine. Knocking down of transferrin resulted in decreased cell death and ROS after treatment.	Validated	27441659
ALOX5	Arachidonate 5-lipoxygenase	Silencing ALOX genes made cells resistant to ferroptosis.	Validated	27506793
ALOX12	Arachidonate 12-lipoxygenase, 12S type	Silencing ALOX genes made cells resistant to ferroptosis.	Validated	27506793
ALOX12B	Arachidonate 12-lipoxygenase, 12R type	Silencing ALOX genes made cells resistant to ferroptosis.	Validated	27506793
ALOX15	Arachidonate 15-lipoxygenase	Silencing ALOX genes made cells resistant to ferroptosis.	Validated	27506793
ALOX15B	Arachidonate 15-lipoxygenase type B	Silencing ALOX genes made cells resistant to ferroptosis. Erastin-induced cell death was rescued by silencing either ALOX15B or ALOXE3, which supported the hypothesis that lipoxygenases are required for ferroptosis.	Validated	27506793
ALOXE3	Arachidonate lipoxygenase 3	Silencing ALOX genes made cells resistant to ferroptosis. Erastin-induced cell death was rescued by silencing either ALOX15B or ALOXE3, which supported the hypothesis that lipoxygenases are required for ferroptosis.	Validated	27506793
PHKG2	Phosphorylase kinase catalytic subunit gamma 2	U-2-OS cells became resistant to erastin upon PHKG2 silencing. Erastin-treated HT-1080 cells were rescued by shPHKG2.	Validated	27506793
TFRC	Transferrin receptor	The gene targets of the enriched shRNAs are potential genes that positively regulate ferroptosis.	Screened	27514700
ACO1	Aconitase 1	The gene targets of the enriched shRNAs are potential	Screened	2751470

		genes that positively regulate ferroptosis.		0
IREB2	iron responsive element binding protein 2	The gene targets of the enriched shRNAs are potential genes that positively regulate ferroptosis.	Screened	2751470 0
SLC38A1	Solute carrier family 38 member 1	The gene targets of the enriched shRNAs are potential genes that positively regulate ferroptosis.	Screened	2751470 0
GLS2	Glutaminase 2	The gene targets of the enriched shRNAs are potential genes that positively regulate ferroptosis.	Screened	2751470 0
G6PDX	_NA_	The gene targets of the enriched shRNAs are potential genes that positively regulate ferroptosis.	Screened	2751470 0
ULK1	Unc-51 like autophagy activating kinase 1	Potential positive regulators of ferroptosis. Knockout of ULK1 led to significantly lower levels of erastin-induced ferroptosis in a dose- and time-dependent manner.	Validated	2751470 0
ATG3	Autophagy related 3	Potential positive regulators of ferroptosis. Knockout of ATG3 greatly reduced the sensitivity of MEFs to ferropotosis, and reconstituting ATG3 back to these cells restored the ferroptosis sensitivity.	Validated	2751470 0
ATG4D	Autophagy related 4D cysteine peptidase	Potential positive regulators of ferroptosis.	Screened	2751470 0
ATG5	Autophagy related 5	Potential positive regulators of ferroptosis. Knockout of ATG5 led to significantly lower levels of erastin-induced ferroptosis in a dose- and time-dependent manner.	Validated	2751470 0
BECN1	Beclin 1	Potential positive regulators of ferroptosis.	Screened	2751470 0
MAP1LC3A	Microtubule associated protein 1 light chain 3 alpha	Potential positive regulators of ferroptosis.	Screened	2751470 0
GABARAPL2	GABA type A receptor associated protein like 2	Potential positive regulators of ferroptosis.	Screened	2751470 0
GABARAPL1	GABA type A receptor associated protein like 1	Potential positive regulators of ferroptosis.	Screened	2751470 0
ATG16L1	Autophagy related 16 like 1	Potential positive regulators of ferroptosis.	Screened	2751470 0
WIPI1	WD repeat domain, phosphoinositide interacting 1	Potential positive regulators of ferroptosis.	Screened	2751470 0

WIPI2	WD repeat domain, phosphoinositide interacting 2	Potential positive regulators of ferroptosis.	Screened	27514700
SNX4	Sorting nexin 4	Potential positive regulators of ferroptosis.	Screened	27514700
ATG13	Autophagy related 13	Knockout of ATG13 greatly reduced the sensitivity of MEFs to ferropotosis, and reconstituting ATG13 back to these cells restored the ferroptosis sensitivity.	Validated	27514700
ULK2	Unc-51 like autophagy activating kinase 2	Knockout of ULK2 led to significantly lower levels of erastin-induced ferroptosis in a dose- and time-dependent manner.	Validated	27514700
NCOA4	Nuclear receptor coactivator 4	Elimination of NCOA4 expression by RNAi knockdown significantly block ferroptosis	Validated	27514700
ACSL4	Acyl-CoA synthetase long chain family member 4	Knockdown inhibited erastin-induced ferroptosis, whereas overexpression restored ferroptosis sensitization.	Validated	27565726
TP53	Tumor protein p53	p53 acetylation has a critical role in ferroptotic responses. Simultaneous loss of K98/117/161/162 acetylations is unable to induce ferroptosis, and its ability to thwart cancer growth is also abrogated.	Validated	27705786
SAT1	Spermidine/spermine N1-acetyltransferase 1	p53-mediated activation of SAT1 contributes to ferroptotic cell death in the presence of ROS stress. Knockdown of Sat1 partially rescued ROS-induced ferroptosis.	Validated	27698118
ALOX15	Arachidonate 15-lipoxygenase	SAT1- and ROS-induced ferroptosis was completely abrogated by PD146176, an ALOX15-specific inhibitor.	Validated	27698118
ACSL4	Acyl-CoA synthetase long chain family member 4	Inhibition of ACSL4 was effective in protecting against RSL3-induced cell death. Acsl4 KO cells are resistant to ferroptosis.	Validated	27842066
LPCAT3	Lysophosphatidylcholine acyltransferase 3	Knockdown of Lpcat3 increased resistance to ferroptosis triggered by RSL3.	Validated	27842066
ALOX15	Arachidonate 15-lipoxygenase	Liproxstatin-1 inhibited the 15-LOX enzymatic activity and suppressed ferroptosis.	Validated	27842066
ACSL4	Acyl-CoA synthetase long chain family member 4	An essential proferroptotic gene. Re-expression of Flag-tagged human wild-type (WT) ACSL4 (ACSL4-Flag) in Acsl4 KO Pfa1 cells restored full sensitivity to ferroptosis	Validated	27842070

		induction. Inhibition showed significantly prolonged survival compared to vehicle-treated mice.		
KEAP1	Kelch like ECH associated protein 1	Keap 1 silencing decreased ferroptosis.	Validated	28012440
EGFR	Epidermal growth factor receptor	Cell death in activated EGFR mutant cells occurs by ferroptosis. Inhibiting EGFR and MAPK signaling rescued cell viability following cystine withdrawal.	Validated	28297659
NOX4	NADPH oxidase 4	Inhibition of NADPH oxidase 4 (NOX4) blocked ferroptosis.	Validated	28297659
MAPK3	Mitogen-activated protein kinase 3	Inhibiting EGFR and MAPK signaling rescued cell viability following cystine withdrawal.	Validated	28297659
MAPK1	Mitogen-activated protein kinase 1	Inhibiting EGFR and MAPK signaling rescued cell viability following cystine withdrawal.	Validated	28297659
BID	BH3 interacting domain death agonist	BID deletion prevents erastin- and glutamate-induced cell death. BID inhibition inhibited erastin-induced ferroptosis.	Validated	28384611
ACSL4	Acyl-CoA synthetase long chain family member 4	Knockout of Acsl4 in ferroptosis-sensitive cells conferred protection from erastin- and RSL3-induced cell death.	Validated	28551825
ZEB1	Zinc finger E-box binding homeobox 1	Knockout of ZEB1 prevents cell death induced by GPX4 inhibition.	Validated	28678785
KEAP1	Kelch like ECH associated protein 1	Keap1 inhibition promotes resistance to ferroptosis.	Validated	28805788
DPP4	Dipeptidyl peptidase 4	Required for ferroptosis in TP53-deficient CRC cells.	Validated	28813679
ALOX15	Arachidonate 15-lipoxygenase	Suppression of ferroptosis following ALOX15 silencing was detected in cancer cells. Cells with exogenous expression of ALOX15 had an increased cell death rate following RSL3 treatment.	Validated	28837253
ALOX12	Arachidonate 12-lipoxygenase, 12S type	12-LOX inhibitors prevented cell death, whereas ALOX12 overexpression significantly enhanced cell death. ALOX12 expression was gradually elevated during the erastin or RSL3 treatments, and was stable in the late stage of ferroptosis.	Validated	28837253

CDKN2A	Cyclin dependent kinase inhibitor 2A	Combination of ARF induction and ROS treatment induced ferroptotic cell death. Knockdown of endogenous ARF protected cells from ROS-induced cell death.	Validated	28985506
PEBP1	Phosphatidylethanolamine binding protein 1	Elevated levels of PEBP1 resulted in increased sensitivity of HK2 cells to RSL3 whereas lowered contents of PEBP1 in HAEC and HT22 cells were associated with decreased sensitivity to ferroptosis.	Validated	29053969
SOCS1	Suppressor of cytokine signaling 1	Expression of SOCS1 sensitized cells to ferroptosis inducer. This effect of SOCS1 was efficiently blocked by ferroptosis inhibitor. Expression of SOCS1 reduced the levels of GSH, explaining in part its ability to sensitize cells to ferroptosis.	Validated	29081404
CDO1	Cysteine dioxygenase type 1	CDO1 suppression contributes to ferroptosis resistance.	Validated	29144989
MYB	MYB proto-oncogene, transcription factor	Erastin-induced ferroptosis was restrained when c-Myb was suppressed.	Validated	29144989
HMOX1	Heme oxygenase 1	Inhibiting HO-1 effectively attenuated BAY-induced ferroptotic cell death. Defective HO-1 expression significantly rescued cell survival suppressed by BAY.	Validated	29274359
MAPK8	Mitogen-activated protein kinase 8	JNK1/2 inhibitors inhibited t-BHP-induced ferroptosis. t-BHP treatment significantly increased the protein expression of p-JNK.	Validated	29330409
MAPK9	Mitogen-activated protein kinase 9	JNK1/2 inhibitors inhibited t-BHP-induced ferroptosis. t-BHP treatment significantly increased the protein expression of p-JNK.	Validated	29330409
MAPK1	Mitogen-activated protein kinase 1	ERK1/2 inhibitors inhibited t-BHP-induced ferroptosis. t-BHP treatment significantly increased the protein expression of p-ERK.	Validated	29330409
MAPK3	Mitogen-activated protein kinase 3	ERK1/2 inhibitors inhibited t-BHP-induced ferroptosis. t-BHP treatment significantly increased the protein expression of p-ERK.	Validated	29330409
SLC1A5	Solute carrier family 1 member 5	Overexpression of SLC1A5 restored miR-137-mediated ferroptosis suppression.	Validated	29348676

CHAC1	ChaC glutathione specific gamma-glutamylcyclotransferase 1	CHAC1 degradation of GSH might enhance cystine-starvation-induced cell death.	Validated	29383104
MAPK14	Mitogen-activated protein kinase 14	Ferroptosis was blocked by inhibiting p38 MAPK activation.	Validated	29436589
LINC00472	Long intergenic non-protein coding RNA 472	Increases erastin-induced growth inhibition, whereas depletion of P53RRA decreased erastin-induced growth inhibition.	Validated	29588351
NOX4	NADPH oxidase 4	Activated Nox4 contributes to PAB-induced ferroptotic cell death. knockdown made cells resistant to PAB-induced death.	Validated	29702192
GOT1	Glutamic-oxaloacetic transaminase 1	Overexpression of miR-9 suppressed GOT1, which subsequently reduced ferroptosis. Overexpression of GOT1 restored miR-9 mediated ferroptosis suppression.	Validated	30035324
BECN1	Beclin 1	Knockdown inhibits ferroptosis. Overexpression increases ferroptotic cancer cell death.	Validated	30057310
PRKAA2	Protein kinase AMP-activated catalytic subunit alpha 2	Inhibition of PRKAA/AMPKalpha diminishes ferroptosis.	Validated	30057310
PRKAA1	Protein kinase AMP-activated catalytic subunit alpha 1	Inhibition of PRKAA/AMPKalpha diminishes ferroptosis.	Validated	30057310
ELAVL1	ELAV like RNA binding protein 1	ELAVL1 siRNA led to ferroptosis resistance, whereas ELAVL1 plasmid contributed to classical ferroptotic events.	Validated	30081711
BAP1	BRCA1 associated protein 1	Suppresses SLC7A11-mediated cystine uptake and promotes ferroptosis. BAP1 mutants lose their abilities to repress SLC7A11 and to promote ferroptosis.	Validated	30202049
TP53	Tumor protein p53	Facilitates ART-induced ferroptosis. Conversely, knockdown of P53 blocked ART-induced ferroptosis.	Validated	30321484
ABCC1	ATP binding cassette subfamily C member 1	Accelerates ferroptosis. Disruption of MRP1 inhibited ferroptosis potently.	Validated	30726737
ACSL4	Acyl-CoA synthetase long chain family member 4	Inhibition suppresses ferroptosis.	Validated	30737476
MIR6852	microRNA 6852	Promotes ferroptosis. Binds to LINC0033 and serves as a	Validated	3078739

		negative upstream regulator of CBS-mediated ferroptosis inhibition.		2
ACVR1B	Activin A receptor type 1B	Inhibition attenuated erastin-induced ferroptosis.	Validated	30804470
TGFBR1	Transforming growth factor beta receptor 1	Inhibition attenuated erastin-induced ferroptosis.	Validated	30804470
BAP1	BRCA1 associated protein 1	Promotes ferroptosis induced by class I ferroptosis inducer.	Validated	30907299
EPAS1	Endothelial PAS domain protein 1	A driver of ferroptosis susceptibility. Ablation reduced susceptibility to ferroptosis.	Validated	30962421
HILPDA	Hypoxia inducible lipid droplet associated	Promotes ferroptosis sensitivity downstream of HIF-2alpha.	Validated	30962421
HIF1A	Hypoxia inducible factor 1 subunit alpha	Re-sensitized HIF-2alpha-null cells to ferroptosis. Induce ferroptosis sensitivity in cancer cells.	Validated	30962421
ALOX12	Arachidonate 12-lipoxygenase, 12S type	An essential factor of p53-dependent ferroptosis. Loss of one Alox12 allele is sufficient to abrogate p53-mediated ferroptosis.	Validated	30962574
ACSL4	Acyl-CoA synthetase long chain family member 4	Required for ferroptosis induced by erastin. ACSL4-null cells are resistant to ferroptosis induced by either erastin.	Validated	30962574
HMOX1	Heme oxygenase 1	Enhances the ferroptotic process in PRDX6-silenced cells by promoting cellular accumulation of ferrous ions. Overexpression increases both erastin and RSL-3-induced lipid ROS.	Validated	31036877
IFNG	Interferon gamma	Interferon gamma released from CD8+ T cells downregulates the expression of SLC3A2 and SLC7A11, and as a consequence, promotes tumour cell lipid peroxidation and ferroptosis.	Validated	31043744
ANO6	Anoctamin 6	Essential for ferroptosis. Inhibition blocked ferroptotic cell death induced by RSL3/erastin.	Validated	31060306
LPIN1	Lipin 1	Overexpression of adipose lipin-1 in mice facilitated the onset of hepatic ferroptosis.	Validated	31061954
HMGB1	High mobility group box 1	Required for erastin-induced ferroptosis. Knockdown of	Validated	3110599

		HMGB1 decreased erastin-induced cell death.		9
TNFAIP3	TNF alpha induced protein 3	Overexpression increased ROS generation and enhanced erastin-induced ferroptosis, whereas knockdown inhibited erastin-induced ferroptosis.	Validated	31160087
TLR4	Toll like receptor 4	Knockdown inhibited ferroptosis.	Validated	31196626
NOX4	NADPH oxidase 4	Knockdown inhibited ferroptosis.	Validated	31196626
ATF3	Activating transcription factor 3	Promotes ferroptosis induced by erastin.	Validated	31273299
ATM	ATM serine/threonine kinase	Essential for ferroptosis. Genetic knockdown and chemical inhibition of ATM both suppress ferroptotic cell death.	Validated	31320750
YY1AP1	YY1 associated protein 1	Makes cells more sensitive to ferroptosis. Cells lacking YAP were no longer sensitised to ferroptosis.	Validated	31341276
EGLN2	Egl-9 family hypoxia inducible factor 2	Inhibiting EGLN2 activation diminished ferroptotic tumor cell death.	Validated	31355331
MIOX	Myo-inositol oxygenase	Overexpression exacerbates cell death, knockdown inhibits ferroptosis.	Validated	31437128
TAZ	Tafazzin	TAZ removal confers ferroptosis resistance, whereas overexpression of TAZS89A sensitizes cells to ferroptosis.	Validated	31484063
MTDH	Metadherin	Can enhance sensitivity to inducers of ferroptosis. Enhances the vulnerability of cancer cells to ferroptosis.	Validated	31527591
IDH1	Isocitrate dehydrogenase (NADP(+)) 1	Deletion of the mutant IDH1 allele or pharmacological inhibition of mutant IDH1 confers resistance to erastin-induced ferroptosis. Ectopic expression of mutant IDH1 promotes ferroptosis.	Validated	31591388
SIRT1	Sirtuin 1	Knockout partially mitigates ferroptosis.	Predicted	31610175
TAZ	Tafazzin	TAZ removal confers ferroptosis resistance, while TAZS89A overexpression sensitizes cells to ferroptosis.	Validated	31641008
BECN1	Beclin 1	Overexpression aggravated isoflurane-induced cell damage by upregulating ferroptosis. This phenomenon was significantly attenuated by silencing of Beclin1.	Validated	31650158

FBXW7	F-box and WD repeat domain containing 7	FBXW7 plasmid induces ferroptosis.	Deduced	31679460
PANX1	Pannexin 1	Deletion protects against ferroptotic cell death. Silenced Panx1 expression significantly attenuated ferroptotic lipid peroxidation and iron accumulation induced by the ferroptosis inducer erastin.	Validated	31694915
DNAJB6	DnaJ heat shock protein family (Hsp40) member B6	Promotes ferroptosis in esophageal squamous cell carcinoma.	Predicted	31701262
BACH1	BTB domain and CNC homolog 1	Promotes ferroptosis by repressing the transcription of a subset of the erastin-induced protective genes.	Validated	31740582
ACSL4	Acyl-CoA synthetase long chain family member 4	Overexpression induced ferroptosis. The opposite results were observed when ACSL4 was silenced.	Validated	31789401
LONP1	Lon peptidase 1, mitochondrial	Inhibition of LONP1 negatively regulates erastin-induced cell death.	Validated	31822343
**Suppressors which are genes that promote ferroptosis**				
SLC7A11	Solute carrier family 7 member 11	Silencing of SLC7A11 sensitized HT-1080 cells to erastin-induced death, whereas transfection of HT-1080 cells with a plasmid encoding SLC7A11 conferred protection from erastin- and sulfasalazine-induced death.	Validated	22632970
GPX4	Glutathione peroxidase 4	RNAi-mediated GPX4 knockdown induces ferroptosis.	Validated	24439385
AKR1C1	Aldo-keto reductase family 1 member C1	Up-regulated in DU-145 erastin-resistant clones. Participate in the detoxification of toxic lipid metabolites. May confer partial resistance to erastin by enhancing the detoxification of reactive aldehydes generated downstream of the oxidative destruction of the plasma membrane during ferroptosis.	Validated	24844246
AKR1C2	Aldo-keto reductase family 1 member C2	Up-regulated in DU-145 erastin-resistant clones. Participate in the detoxification of toxic lipid metabolites. May confer partial resistance to erastin by enhancing the detoxification of reactive aldehydes generated downstream of the oxidative destruction of the plasma membrane during ferroptosis.	Validated	24844246

AKR1C3	Aldo-keto reductase family 1 member C3	Up-regulated in DU-145 erastin-resistant clones. Participate in the detoxification of toxic lipid metabolites. May confer partial resistance to erastin by enhancing the detoxification of reactive aldehydes generated downstream of the oxidative destruction of the plasma membrane during ferroptosis.	Validated	24844246
GPX4	Glutathione peroxidase 4	Knockout of glutathione peroxidase 4 (Gpx4) causes cell death in a pathologically relevant form of ferroptosis. Knockdown renders cells more sensitive to ferroptosis-inducing agents.	Validated	25402683
RB1	RB transcriptional corepressor 1	Rb knock-down cells exposed to sorafenib encounter ferroptosis. Lack of Rb sensitized HCC cells to the induction of ferroptosis.	Validated	25444922
HSPB1	Heat shock protein family B (small) member 1	Knockdown of HSF1 and HSPB1 enhances erastin-induced ferroptosis, whereas heat shock pretreatment and overexpression of HSPB1 inhibits erastin-induced ferroptosis.	Validated	25728673
HSF1	Heat shock transcription factor 1	Knockdown of HSF1 and HSPB1 enhances erastin-induced ferroptosis, whereas heat shock pretreatment and overexpression of HSPB1 inhibits erastin-induced ferroptosis.	Validated	25728673
SLC7A11	Solute carrier family 7 member 11	Overexpressed in human cancer specimens. Overexpression inhibits ROS-induced ferroptosis.	Validated	25799988
GPX4	Glutathione peroxidase 4	Ex vivo, Gpx4-deficient T cells rapidly accumulated membrane lipid peroxides and concomitantly underwent cell death driven by ferroptosis.	Validated	25824823
GCLC	Glutamate-cysteine ligase catalytic subunit	RNAi knockdown sensitized cell death induced by cystine starvation.	Validated	26166707
SLC7A11	Solute carrier family 7 member 11	Overexpression of SLC7A11 considerably abrogated ferroptosis.	Validated	26218928
NFE2L2	Nuclear factor, erythroid 2 like 2	NRF2 plays a central role in protecting hepatocellular carcinoma (HCC) cells against ferroptosis	Validated	26403645
SQSTM1	Sequestosome 1	The interaction between p62 and Keap1 increased	Validated	2640364

		following erastin and sorafenib treatment. Knockdown of p62 suppressed NRF2 expression and promoted growth inhibition with increased ferroptotic events including GSH depletion, lipid ROS production, and an increase of iron levels.		5
NQO1	NAD(P)H quinone dehydrogenase 1	Knockdown of p62, quinone oxidoreductase-1, heme oxygenase-1, and ferritin heavy chain-1 by RNA interference in HCC cells promoted ferroptosis in response to erastin and sorafenib.	Validated	26403645
HMOX1	Heme oxygenase 1	Knockdown of p62, quinone oxidoreductase-1, heme oxygenase-1, and ferritin heavy chain-1 by RNA interference in HCC cells promoted ferroptosis in response to erastin and sorafenib.	Validated	26403645
FTH1	Ferritin heavy chain 1	Knockdown of p62, quinone oxidoreductase-1, heme oxygenase-1, and ferritin heavy chain-1 by RNA interference in HCC cells promoted ferroptosis in response to erastin and sorafenib.	Validated	26403645
MUC1	Mucin 1, cell surface associated	MUC1-C (C-terminal subunit) blocks erastin-induced ferroptosis and induces increases in GSH.	Validated	26930718
SLC3A2	Solute carrier family 3 member 2	Required for in vitro cell survival because of its role in protecting cells from ferroptosis.	Validated	26945935
MT1G	Metallothionein 1G	A negative regulator of ferroptosis in HCC cells. Knockdown of MT-1G by RNA interference increases glutathione depletion and lipid peroxidation, which contributes to sorafenib-induced ferroptosis.	Validated	27015352

NFE2L2	Nuclear factor, erythroid 2 like 2	Required for sorafenib-induced expression of MT-1G which is a ferroptosis suppressor.	Deduced	27015352
SLC40A1	Solute carrier family 40 member 1	Involved in siramesine and lapatinib-induced ferroptotic cell death. Its expression is decreased after treatment with siramesine alone or in combination with lapatinib. Overexpression FPN resulted in decreased ROS and cell death whereas knockdown of FPN increased cell death after siramesine and lapatinib treatment.	Validated	27441659
SLC7A11	Solute carrier family 7 member 11	Knockdown increased cell death.	Validated	27441659
GPX4	Glutathione peroxidase 4	Knockdown increased cell death.	Validated	27441659
SLC7A11	Solute carrier family 7 member 11	Inhibition induces ferroptosis. Silencing of the SLC7A11 gene increases the cisplatin sensitivity of resistant HNC cells.	Validated	27477897
CISD1	CDGSH iron sulfur domain 1	Genetic inhibition of CISD1 contributes to erastin-induced ferroptosis. Stabilization of the iron sulfur cluster of CISD1 inhibits ferroptosis.	Validated	27510639
SLC7A11	Solute carrier family 7 member 11	Elevated levels of expression are resistant to erastin-induced ferroptosis. Repression of SLC7A11 expression by p53 sensitized cells to undergo erastin-induced ferroptosis.	Validated	27705786
FANCD2	FA complementation group D2	Inhibits erastin-induced ferroptosis. Plays a novel role in the negative regulation of ferroptosis.	Validated	27773819
GPX4	Glutathione peroxidase 4	Protects lipid peroxidation. Cell damage induced by GPx4 ablation is involved in ferroptosis.	Validated	27964880
NFE2L2	Nuclear factor, erythroid 2 like 2	Nrf2 activation contributes to the resistance of HNCs to artesunate-induced ferroptosis. Nrf2 inhibition sensitizes head and neck cancer cells to artesunate-induced ferroptosis.	Validated	28012440
FTMT	Ferritin mitochondrial	Overexpression significantly inhibited erastin-induced ferroptosis.	Validated	28066232

HSPA5	Heat shock protein family A (Hsp70) member 5	Negatively regulates ferroptosis. Suppression of HSPA5 expression increased erastin-induced death. Overexpressed HSPA5 inhibited erastin-induced ferroptotic cell death.	Validated	28130223
ATF4	Activating transcription factor 4	Inhibition of ATF4 expression increased erastin-induced cell death. ATF4 results in the induction of HSPA5, which in turn protects against GPX4 protein degradation and subsequent ferroptosis.	Validated	28130223
SLC7A11	Solute carrier family 7 member 11	Slc7a11 deletion increases susceptibility to iron overload-induced ferroptosis.	Validated	28195347
GPX4	Glutathione peroxidase 4	Ferroptosis drives neurodegeneration in Gpx4BIKO mice.	Validated	28212525
GPX4	Glutathione peroxidase 4	Downregulation conferred increased sensitivity to ferroptosis following cystine deprivation.	Validated	28297659
HMOX1	Heme oxygenase 1	Demonsrates antiferroptotic role. HO-1 deficiency promotes erastin-induced ferroptosis.	Validated	28515173
ATF4	Activating transcription factor 4	ATF4 expression induces acquired cell death resistance. ATF4 knockdown renders cells susceptible for ferroptosis.	Validated	28553953
NFE2L2	Nuclear factor, erythroid 2 like 2	Nrf2 over expression promotes resistance to ferroptosis.	Validated	28805788
TP53	Tumor protein p53	Inhibits ferroptosis in human colorectal cancer (CRC) cells. Loss of TP53 restored erastin sensitivity. Inhibits cell death induction by erastin in human CRC cells.	Validated	28813679
SLC7A11	Solute carrier family 7 member 11	Knockdown sensitized cells to erastin.	Validated	28813679
HELLS	Helicase, lymphoid specific	LSH inhibits ferroptosis by decreasing the intracellular levels of iron and lipid ROS.	Validated	28900510
SCD	Stearoyl-CoA desaturase	Depletion of the SCD1 and FADS2 metabolic genes induces ferroptosis.	Validated	28900510
FADS2	Fatty acid desaturase 2	Depletion of the SCD1 and FADS2 metabolic genes induces ferroptosis.	Validated	28900510
SRC	SRC proto-oncogene, non-receptor tyrosine kinase	Src-STAT3 activation renders the cell unable to undergo to ferroptosis. Src inhibition decreased cell viability	Validated	28972104

		significantly, and that loss of viability was rescued by ferroptosis inhibitors.		
STAT3	Signal transducer and activator of transcription 3	Src-STAT3 activation renders the cell unable to undergo to ferroptosis.	Validated	28972104
NFE2L2	Nuclear factor, erythroid 2 like 2	ARF-mediated ferroptosis was largely abrogated by co-expression of NRF2.	Validated	28985506
PML	Promyelocytic leukemia	PML expression turned cells highly resistant to ferroptosis.	Validated	29081404
MTOR	Mechanistic target of rapamycin kinase	Necessary and sufficient to protect cardiomyocyte cells against ferroptotic cell death. mTOR overexpression suppressed ferroptotic cell death, whereas mTOR deletion exaggerated cell death.	Validated	29127238
NFS1	NFS1 cysteine desulfurase	Suppression of NFS1 cooperates with inhibition of cysteine transport to trigger ferroptosis in vitro and slow tumour growth. Suppression of NFS1 predisposes cancer cells to ferroptosis.	Validated	29168506
TP63	Tumor protein p63	Delta Np63 alpha can inhibit ferroptosis independent of p53. Overexpression protects cells from ferroptosis-inducing agents.	Validated	29212036
SLC7A11	Solute carrier family 7 member 11	Overexeperession of SLC7A11 attenuated BAY-inhibited cell viability by ferroptosis.	Validated	29274359
TP53	Tumor protein p53	p53 stabilization suppresses ferroptosis. p53 suppresses metabolic stress-induced ferroptosis.	Validated	29346757
CDKN1A	Cyclin dependent kinase inhibitor 1A	Required to to suppress ferroptosis.	Validated	29346757
MIR137	microRNA 137	Suppresses ferroptosis both in vitro and in vivo.	Validated	29348676
SLC40A1	Solute carrier family 40 member 1	Overexpression of Fpn inhibited ferroptosis.	Validated	29436589
GPX4	Glutathione peroxidase 4	Activation blocked ferroptosis.	Validated	29436589
GPX4	Glutathione peroxidase 4	GPX4-overexpressing cells were resistant to reactive oxygen species-induced cell death. Conversely,	Deduced	29463878

		GPX4-knockdown cells were sensitive to reactive oxygen species-induced cell death.		
ENPP2	Ectonucleotide pyrophosphatase/phosphodiesterase 2	Overexpression modestly promotes migration and proliferation and significantly inhibits erastin-induced ferroptosis.	Validated	29551679
VDAC2	Voltage dependent anion channel 2	Overexpression could partially protect cells from ferroptosis.	Validated	29569437
FH	Fumarate hydratase	FH inactivation (FH-/-) proves synthetic lethal with inducers of ferroptosis. FH-/- sensitizes cells to multiple ferroptosis inducers.	Validated	29917289
CISD2	CDGSH iron sulfur domain 2	Overexpression conferred resistance to ferroptosis. Inhibition blocked resistance to ferroptotic cell death.	Validated	29928961
SLC40A1	Solute carrier family 40 member 1	A negative regulator of ferroptosis by reducing intracellular iron concentration. Knockdown accelerates erastin-induced ferroptosis.	Validated	29949159
MIR9-1	microRNA 9-1	Overexpression of miR-9 suppressed GOT1, which subsequently reduced ferroptosis. Suppression of miR-9 increased the sensitivity of melanoma cells to ferroptosis inducers.	Validated	30035324
MIR9-2	microRNA 9-2	Overexpression of miR-9 suppressed GOT1, which subsequently reduced ferroptosis. Suppression of miR-9 increased the sensitivity of melanoma cells to ferroptosis inducers.	Validated	30035324
MIR9-3	microRNA 9-3	Overexpression of miR-9 suppressed GOT1, which subsequently reduced ferroptosis. Suppression of miR-9 increased the sensitivity of melanoma cells to ferroptosis inducers.	Validated	30035324

CBS	Cystathionine beta-synthase	Inhibition triggers ferroptosis in hepatocellular carcinoma.	Validated	30258181
NFE2L2	Nuclear factor, erythroid 2 like 2	Associated with resistance to ferroptosis. Inhibition of Nrf2 sensitized cells to ferroptosis.	Validated	30339884
SQSTM1	Sequestosome 1	Inhibition of the p62 gene significantly reduced cell viability and increased cellular lipid ROS levels in HN3R cells; this was reversed by treatment with ferrostatin-1, a ferroptosis inducer.	Validated	30339884
GPX4	Glutathione peroxidase 4	Overexpression GPX4 resulted in decreased cell death after RSL3 treatment. Moreover, this effect was able to be reversed by overexpression of GPX4.	Validated	30524291
ISCU	Iron-sulfur cluster assembly enzyme	Over expression significantly attenuated DHA induced ferroptosis.	Validated	30557609
FTH1	Ferritin heavy chain 1	FTH reconstituted cells exhibited the reduced lipid peroxides content and restored the DHA-induced ferroptosis.	Validated	30557609
ACSL3	Acyl-CoA synthetase long chain family member 3	Required for exogenous monounsaturated fatty acids to protect cells against ferroptosis. Negatively correlates with ferroptosis sensitivity.	Validated	30686757
OTUB1	OTU deubiquitinase, ubiquitin aldehyde binding 1	Inactivation promotes ferroptosis by down-regulating SLC7A11 levels. Overexpression is critical for tumor growth.	Validated	30709928
CD44	CD44 molecule (Indian blood group)	Knockdown sensitizes cells to ferroptosis.	Validated	30709928
LINC00336	Long intergenic non-protein coding RNA 336	Overexpression inhibits ferroptosis. Knockdown promotes ferroptosis.	Validated	30787392
STAT3	Signal transducer and activator of transcription 3	Upregulated in ferroptosis resistant cells. Inhibition increases ferroptosis.	Validated	30811078
BRD4	Bromodomain containing 4	Inhibition induces ferroptosis.	Validated	30988278
PRDX6	Peroxiredoxin 6	A negative regulator of ferroptotic cell death.	Validated	31036877
MIR17	microRNA 17	Protects endothelial HUVEC cells from erastin-induced	Validated	3116008

		ferroptosis. Overexpression significantly reduced erastin-induced growth inhibition and ROS generation of HUVEC cells.		7
SCD	Stearoyl-CoA desaturase	Inhibition of SCD1 induces ferroptotic cell death. Expression of SCD1 protects cells from ferroptosis.	Validated	31270077
SESN2	Sestrin 2	Has cytoprotective effect against ferroptosis. In cells expressing Sesn2, erastin-induced cell death, ROS formation, and glutathione depletion were almost completely inhibited compared to that in control cells.	Validated	31323261
NF2	Neurofibromin 2	Genetic inactivation of NF2 rendered cancer cells more sensitive to ferroptosis. Mediates cell density-dependent inhibition of ferroptosis.	Validated	31341276
ARNTL	Aryl hydrocarbon receptor nuclear translocator like	Degration of the protein is critical for ferroptosis. Blocking ARNTL degradation diminished ferroptotic tumor cell death.	Validated	31355331
HIF1A	Hypoxia inducible factor 1 subunit alpha	Destabilizing HIF1A facilitated ferroptotic tumor cell death.	Validated	31355331
JUN	Jun proto-oncogene, AP-1 transcription factor subunit	O-GlcNAcylated c-Jun represents an obstructive factor to ferroptosis.	Deduced	31394193
CA9	Carbonic anhydrase 9	Inhibition induces ferroptosis.	Validated	31442913
HSPA5	Heat shock protein family A (Hsp70) member 5	Knockdown of GRP78 enhanced artesunate-induced ferroptosis of pancreatic cancer cells.	Validated	31456633
TMBIM4	Transmembrane BAX inhibitor motif containing 4	Protects against ferroptosis in HCC cells. Inhibition increased ferroptotic cel death.	Validated	31507082
HSPA5	Heat shock protein family A (Hsp70) member 5	Serves as a negative regulator of DHA-induced ferroptosis.	Predicted	31519193
PLIN2	Perilipin 2	An indispensable gene and protein in the suppression of ferroptosis caused by abnormal lipometabolism in gastric carcinoma.	Screened	31520166
MIR212	microRNA 212	Overexpression of miR-212-5p attenuated ferroptosis while downregulation of miR-212-5p promoted ferroptotic cell death.	Validated	31533781

Fer1HCH	Ferritin 1 Heavy Chain Homolog	Reduced heavy chain levels caused severe mitochondrial defects and ferroptosis.	Predicted	31568497
AIFM2	Apoptosis inducing factor mitochondria associated 2	A glutathione-independent ferroptosis suppressor. Pharmacological targeting of FSP1 strongly synergizes with GPX4 inhibitors to trigger ferroptosis.	Validated	31634899
AIFM2	Apoptosis inducing factor mitochondria associated 2	A potent ferroptosis-resistance factor. Positively correlates with ferroptosis resistance.	Validated	31634900
LAMP2	Lysosomal associated membrane protein 2	Knockdown promoted ferroptosis.	Validated	31672277
ZFP36	ZFP36 ring finger protein	ZFP36 plasmid impaired FBXW7 plasmid-induced HSC ferroptosis. Overexpression of Zfp36 impaired erastin- or sorafenib-induced ferroptosis.	Validated	31679460
GPX4	Glutathione peroxidase 4	Depletion or inhibition resulted in cell death by ferroptosis.	Validated	31685805
PROM2	Prominin 2	Induced by ferroptotic stress and promotes resistance to ferroptotic cell death. Facilitates ferroptosis resistance in mammary epithelial and breast carcinoma cells.	Validated	31735663
CHMP5	Charged multivesicular body protein 5	Ferroptosis activators increase ESCRT-III subunits (e.g., CHMP5 and CHMP6). Knockdown of CHMP5 or CHMP6 sensitizes human cancer cells to ferroptosis.	Validated	31761326
CHMP6	Charged multivesicular body protein 6	Ferroptosis activators increase ESCRT-III subunits (e.g., CHMP5 and CHMP6). Knockdown of CHMP5 or CHMP6 sensitizes human cancer cells to ferroptosis.	Validated	31761326
AKR1C1	Aldo-keto reductase family 1 member C1	Inhibition completely resensitizes resistant melanoma cells to ferroptosis execution.	Validated	31780644
AKR1C2	Aldo-keto reductase family 1 member C2	Inhibition completely resensitizes resistant melanoma cells to ferroptosis execution.	Validated	31780644
AKR1C3	Aldo-keto reductase family 1 member C3	Inhibition completely resensitizes resistant melanoma cells to ferroptosis execution.	Validated	31780644
CBS	Cystathionine beta-synthase	Knockdown in erastin-resistant cells caused ferroptotic cell death, while overexpression conferred ferroptosis resistance.	Validated	31819185
NFE2L2	Nuclear factor, erythroid 2 like 2	Genetically repression of NRF2 enhanced ferroptosis	Validated	3181918

		susceptibility.		5
CAV1	Caveolin 1	Cav-1 deficiency aggravated ferroptosis. Short hairpin RNA of Cav-1 promoted ferroptosis, which was ameliorated by Cav-1 overexpression.	Validated	3187735 7
GCH1	GTP cyclohydrolase 1	Gch1 overexpression and its downstream metabolites BH4/BH2 rescue from ferroptosis. Inhibition of GCH1 activity can sensitize resistant cancer cells to ferroptosis induction.	Validated	3198902 5
**Markers which are genes that promote ferroptosis**				
PTGS2	Prostaglandin-endoperoxide synthase 2	Simply a downstream marker of ferroptosis. The most upregulated gene in BJeLR cells upon treatment with either erastin or (1S, 3R)-RSL3, but ferroptotic cell death was not affected by inhibition of the enzyme.	Validated	2443938 5
DUSP1	Dual specificity phosphatase 1	Expression was upregulated during ferroptosis induced by erastin or RSL3.	Deduced	2443938 5
NOS2	Nitric oxide synthase 2	Expression was upregulated during ferroptosis induced by erastin or RSL3.	Deduced	2443938 5
NCF2	Neutrophil cytosolic factor 2	Expression was upregulated during ferroptosis induced by erastin or RSL3.	Deduced	2443938 5
MT3	Metallothionein 3	Expression was upregulated during ferroptosis induced by erastin or RSL3.	Deduced	2443938 5
UBC	Ubiquitin C	Expression was upregulated during ferroptosis induced by erastin or RSL3.	Deduced	2443938 5
ALB	Albumin	Expression was upregulated during ferroptosis induced by erastin or RSL3.	Deduced	2443938 5
TXNRD1	Thioredoxin reductase 1	Expression was upregulated during ferroptosis induced by erastin or RSL3.	Deduced	2443938 5
SRXN1	Sulfiredoxin 1	Expression was upregulated during ferroptosis induced by erastin or RSL3.	Deduced	2443938 5
GPX2	Glutathione peroxidase 2	Expression was upregulated during ferroptosis induced by erastin or RSL3.	Deduced	2443938 5
BNIP3	BCL2 interacting protein 3	Expression was upregulated during ferroptosis induced by erastin or RSL3.	Deduced	2443938 5

OXSR1	Oxidative stress responsive kinase 1	Expression was upregulated during ferroptosis induced by erastin or RSL3.	Deduced	24439385
SELENOS	Selenoprotein S	Expression was upregulated during ferroptosis induced by erastin or RSL3.	Deduced	24439385
ANGPTL7	Angiopoietin like 7	Expression was downregulated during ferroptosis induced by erastin or RSL3.	Deduced	24439385
CHAC1	ChaC glutathione specific gamma-glutamylcyclotransferase 1	Up-regulated in erastin-treated samples. A useful pharmacodynamic marker of system Xc- inhibition.	Validated	24844246
SLC7A11	Solute carrier family 7 member 11	Similar to erastin treatment, silencing of this gene inhibits glutamate release. Erastin specifically inhibits SLC7A11-dependent system Xc- function.	Deduced	24844246
DDIT4	DNA damage inducible transcript 4	Up-regulated (>= 2 fold) in erastin-treated samples.	Screened	24844246
LOC284561	_NA_	Up-regulated (>= 2 fold) in erastin-treated samples.	Screened	24844246
ASNS	Asparagine synthetase (glutamine-hydrolyzing)	Up-regulated (>= 2 fold) in erastin-treated samples.	Screened	24844246
TSC22D3	TSC22 domain family member 3	Up-regulated (>= 2 fold) in erastin-treated samples.	Screened	24844246
DDIT3	DNA damage inducible transcript 3	Up-regulated (>= 2 fold) in erastin-treated samples.	Screened	24844246
JDP2	Jun dimerization protein 2	Up-regulated (>= 2 fold) in erastin-treated samples.	Screened	24844246
SESN2	Sestrin 2	Up-regulated (>= 2 fold) in erastin-treated samples.	Screened	24844246
SLC1A4	Solute carrier family 1 member 4	Up-regulated (>= 2 fold) in erastin-treated samples.	Screened	24844246
PCK2	Phosphoenolpyruvate carboxykinase 2, mitochondrial	Up-regulated (>= 2 fold) in erastin-treated samples.	Screened	24844246
TXNIP	Thioredoxin interacting protein	Up-regulated (>= 2 fold) in erastin-treated samples.	Screened	24844246
VLDLR	Very low density lipoprotein receptor	Up-regulated (>= 2 fold) in erastin-treated samples.	Screened	24844246

GPT2	Glutamic--pyruvic transaminase 2	Up-regulated (>= 2 fold) in erastin-treated samples.	Screened	24844246
PSAT1	Phosphoserine aminotransferase 1	Up-regulated (>= 2 fold) in erastin-treated samples.	Screened	24844246
LURAP1L	Leucine rich adaptor protein 1 like	Up-regulated (>= 2 fold) in erastin-treated samples.	Screened	24844246
SLC7A5	Solute carrier family 7 member 5	Up-regulated (>= 2 fold) in erastin-treated samples.	Screened	24844246
HERPUD1	Homocysteine inducible ER protein with ubiquitin like domain 1	Up-regulated (>= 2 fold) in erastin-treated samples.	Screened	24844246
XBP1	X-box binding protein 1	Up-regulated (>= 2 fold) in erastin-treated samples.	Screened	24844246
ATF3	Activating transcription factor 3	Up-regulated (>= 2 fold) in erastin-treated samples.	Screened	24844246
SLC3A2	Solute carrier family 3 member 2	Up-regulated (>= 2 fold) in erastin-treated samples.	Screened	24844246
CBS	Cystathionine beta-synthase	Up-regulated (>= 2 fold) in erastin-treated samples.	Screened	24844246
ATF4	Activating transcription factor 4	Up-regulated (>= 2 fold) in erastin-treated samples.	Screened	24844246
ZNF419	Zinc finger protein 419	Up-regulated (>= 2 fold) in erastin-treated samples.	Screened	24844246
KLHL24	Kelch like family member 24	Up-regulated (>= 2 fold) in erastin-treated samples.	Screened	24844246
TRIB3	Tribbles pseudokinase 3	Up-regulated (>= 2 fold) in erastin-treated samples.	Screened	24844246
ZFP69B	ZFP69 zinc finger protein B	Up-regulated (>= 2 fold) in erastin-treated samples.	Screened	24844246
ATP6V1G2	ATPase H+ transporting V1 subunit G2	Up-regulated (>= 2 fold) in erastin-treated samples.	Screened	24844246
VEGFA	Vascular endothelial growth factor A	Up-regulated (>= 2 fold) in erastin-treated samples.	Screened	24844246
GDF15	Growth differentiation factor 15	Up-regulated (>= 2 fold) in erastin-treated samples.	Screened	2484424

				6
TUBE1	Tubulin epsilon 1	Up-regulated (>= 2 fold) in erastin-treated samples.	Screened	24844246
ARRDC3	Arrestin domain containing 3	Up-regulated (>= 2 fold) in erastin-treated samples.	Screened	24844246
CEBPG	CCAAT enhancer binding protein gamma	Up-regulated (>= 2 fold) in erastin-treated samples.	Screened	24844246
SNORA16A	Small nucleolar RNA, H/ACA box 16A	Down-regulated (>= 2 fold) in erastin-treated samples.	Screened	24844246
RGS4	Regulator of G protein signaling 4	Down-regulated (>= 2 fold) in erastin-treated samples.	Screened	24844246
BLOC1S5-TXNDC5	BLOC1S5-TXNDC5 readthrough (NMD candidate)	Down-regulated (>= 2 fold) in erastin-treated samples.	Screened	24844246
LOC390705	_NA_	Down-regulated (>= 2 fold) in erastin-treated samples.	Screened	24844246
EIF2S1	Eukaryotic translation initiation factor 2 subunit 1	Phosphorylated in erastin-treated sample.	Deduced	24844246
KIM-1	Kidney injury molecule-1	Down-regulated upon Fer-1 appearance	Deduced	25385600
IL6	Interleukin 6	Down-regulated upon Fer-1 appearance	Deduced	25385600
CXCL2	C-X-C motif chemokine ligand 2	Down-regulated upon Fer-1 appearance	Deduced	25385600
RELA	RELA proto-oncogene, NF-kB subunit	Down-regulated upon Fer-1 appearance	Deduced	25385600
HSD17B11	Hydroxysteroid 17-beta dehydrogenase 11	Enriched in RSL3-resistant cells.	Screened	25965523
AGPAT3	1-acylglycerol-3-phosphate O-acyltransferase 3	Enriched in RSL3-resistant cells.	Screened	25965523
SETD1B	SET domain containing 1B, histone lysine methyltransferase	Enriched in GPX4 inhibitor ML162-resistant cells.	Screened	25965523
HMOX1	Heme oxygenase 1	Its expression increased in response to artesunate-induced ferroptosis, indicating activation of	Deduced	26097885

		ROS-mediated signaling pathways.		
TF	Transferrin	Its expression is decreased in patients.	Deduced	26097885
FTL	Ferritin light chain	Its expression is decreased in patients.	Deduced	26097885
RPL8	Ribosomal protein L8	Significantly reduced in tumor tissues	Deduced	26097885
ATP5MC3	ATP synthase membrane subunit c locus 3	Significantly reduced in tumor tissues	Deduced	26097885
TFRC	Transferrin receptor	Expression of this gene is increased in patients.	Deduced	26097885
MAFG	MAF bZIP transcription factor G	The interaction between NRF2 and MafG was increased in response to erastin and sorafenib.	Deduced	26403645
IL33	Interleukin 33	IL-33 upregulation is a feature of ferroptosis. Ferrostatin-1, an inhibitor of ferroptosis, prevented the upregulation of IL-33.	Deduced	27352622
FTH1	Ferritin heavy chain 1	An increase of endogenous FTH1 level during ferroptosis. Degradation of FTH1 protein upon ferroptosis induction.	Validated	27514700
SLC40A1	Solute carrier family 40 member 1	Erastin-induced mRNA expression is upregulated in FANCD2-deficient cells.	Validated	27773819
TF	Transferrin	Erastin-induced mRNA expression is upregulated in FANCD2-deficient cells.	Validated	27773819
TFRC	Transferrin receptor	Erastin-induced mRNA expression is upregulated in FANCD2-deficient cells.	Validated	27773819
FTH1	Ferritin heavy chain 1	Erastin-induced mRNA expression is downregulated in FANCD2-deficient cells.	Validated	27773819
GPX4	Glutathione peroxidase 4	Erastin-induced mRNA expression is downregulated in FANCD2-deficient cells.	Validated	27773819
HAMP	Hepcidin antimicrobial peptide	Erastin-induced mRNA expression is downregulated in FANCD2-deficient cells.	Deduced	27773819
HSPB1	Heat shock protein family B (small) member 1	Erastin-induced mRNA expression is downregulated in FANCD2-deficient cells.	Validated	27773819
NFE2L2	Nuclear factor, erythroid 2 like 2	Erastin-induced mRNA expression is downregulated in	Validated	2777381

		FANCD2-deficient cells.		9
STEAP3	STEAP3 metalloreductase	Erastin-induced mRNA expression is downregulated in FANCD2-deficient cells.	Deduced	27773819
DRD5	Dopamine receptor D5	Ferroptotic erastin induces DRD5 gene expression in ferroptosis.	Deduced	27793671
GPX4	Glutathione peroxidase 4	Erastin promoted GPX4 degradation. Antiferroptotic dopamine increased the protein stability of glutathione peroxidase 4.	Deduced	27793671
DRD4	Dopamine receptor D4	Antiferroptotic dopamine suppressed dopamine receptor D4 protein degradation. Ferroptotic erastin promotes DRD4 protein degradation.	Deduced	27793671
MAP3K5	Mitogen-activated protein kinase kinase kinase 5	Cold stress evokes ferroptosis, and the ASK1-p38 pathway is activated downstream of lipid peroxide, leading to the cell death. ASK1-p38 axis is also activated in the erastin-induced ferroptosis model.	Deduced	28887319
MAPK14	Mitogen-activated protein kinase 14	Cold stress evokes ferroptosis, and the ASK1-p38 pathway is activated downstream of lipid peroxide, leading to the cell death. ASK1-p38 axis is also activated in the erastin-induced ferroptosis model.	Deduced	28887319
SLC2A1	Solute carrier family 2 member 1	Increased at LSH overexpression. Decreased at LSH knockdown. LSH can inhibit ferroptosis.	Deduced	28900510
SLC2A3	Solute carrier family 2 member 3	Decreased at LSH knockdown. LSH can inhibit ferroptosis.	Deduced	28900510
SLC2A6	Solute carrier family 2 member 6	Increased at LSH overexpression. Decreased at LSH knockdown. LSH can inhibit ferroptosis.	Deduced	28900511
SLC2A8	Solute carrier family 2 member 8	Decreased at LSH knockdown. LSH can inhibit ferroptosis.	Deduced	28900512
SLC2A12	Solute carrier family 2 member 12	Increased at LSH overexpression. Decreased at LSH knockdown. LSH can inhibit ferroptosis.	Deduced	28900513

GLUT13	_NA_	Increased at LSH overexpression. Decreased at LSH knockdown. LSH can inhibit ferroptosis.	Deduced	28900514
SLC2A14	Solute carrier family 2 member 14	Decreased at LSH knockdown. LSH can inhibit ferroptosis.	Deduced	28900515
EIF2AK4	Eukaryotic translation initiation factor 2 alpha kinase 4	CHAC1 degradation of GSH enhances cystine-starvation-induced ferroptosis through the activated GCN2-eIF2 alpha-ATF4 pathway.	Deduced	29383104
EIF2S1	Eukaryotic translation initiation factor 2 subunit alpha	CHAC1 degradation of GSH enhances cystine-starvation-induced ferroptosis through the activated GCN2-eIF2 alpha-ATF4 pathway.	Deduced	29383104
ATF4	Activating transcription factor 4	CHAC1 degradation of GSH enhances cystine-starvation-induced ferroptosis through the activated GCN2-eIF2 alpha-ATF4 pathway.	Deduced	29383104
ALOX5	Arachidonate 5-lipoxygenase	Overexpression sensitizes cells to ferroptosis.	Deduced	29632885
ALOX12	Arachidonate 12-lipoxygenase, 12S type	Overexpression sensitizes cells to ferroptosis.	Deduced	29632885
ALOX15	Arachidonate 15-lipoxygenase	Overexpression sensitizes cells to ferroptosis.	Deduced	29632885
ALOX5	Arachidonate 5-lipoxygenase	Necessary for hemin-induced ferroptosis in vitro.	Deduced	30294906
ACSF2	Acyl-CoA synthetase family member 2	Increased in ferroptotic events. Decreased in DFO-induced resistance to ferroptosis.	Deduced	30539824
IREB2	Iron responsive element binding protein 2	Increased in ferroptotic events. Decreased in DFO-induced resistance to ferroptosis.	Deduced	30539824
GPX4	Glutathione peroxidase 4	Upregulated in DFO-induced resistance to ferroptosis.	Validated	30539824
HMGB1	High mobility group box 1	Associated with ferroptotic cell death. Ferroptosis activators induce HMGB1 release.	Deduced	30686534
HMOX1	Heme oxygenase 1	Required for DOX-induced ferroptosis.	Deduced	30692261
NFE2L2	Nuclear factor, erythroid 2 like 2	DOX treatment induced ferroptosis. Protein and mRNA levels of Nrf2 were increased after DOX treatment.	Deduced	30692261

ELAVL1	ELAV like RNA binding protein 1	Binds to and increases the expression of the negative ferroptosis regulator LINC00336.	Deduced	3078739 2
SLC3A2	Solute carrier family 3 member 2	Strongly correlated with resistance to ferroptosis inducers.	Deduced	3104374 4
SLC7A11	Solute carrier family 7 member 11	Strongly correlated with resistance to ferroptosis inducers.	Deduced	3104374 4
TFAP2C	Transcription factor AP-2 gamma	Activated by ferroptosis inhibitor selenium.	Deduced	3105628 4
SP1	Sp1 transcription factor	Activated by ferroptosis inhibitor selenium.	Deduced	3105628 4
HBA1	Hemoglobin subunit alpha 1	Upregulated in cells treated with ferroptosis inducer erastin. Stimulates ferroptosis possibly in a GSH-dependent manner.	Deduced	3110846 0
NNMT	Nicotinamide N-methyltransferase	Upregulated in cells treated with ferroptosis inducer erastin. Stimulates ferroptosis possibly in a GSH-dependent manner.	Deduced	3110846 0
PLIN4	Perilipin 4	Upregulated in cells treated with ferroptosis inducer erastin. Stimulates ferroptosis possibly in a GSH-dependent manner.	Deduced	3110846 0
HIC1	HIC ZBTB transcriptional repressor 1	Essential for stimulation of pro-ferroptotic gene transcription upon ferroptosis induction.	Deduced	3110846 0
STMN1	Stathmin 1	Downregulated in cells treated with ferroptosis inducer erastin. Suppresses ferroptosis possibly in a GSH-dependent manner.	Deduced	3110846 0
RRM2	Ribonucleotide reductase regulatory subunit M2	Downregulated in cells treated with ferroptosis inducer erastin. Suppresses ferroptosis possibly in a GSH-dependent manner.	Deduced	3110846 0
CAPG	Capping actin protein, gelsolin like	Downregulated in cells treated with ferroptosis inducer erastin. Suppresses ferroptosis possibly in a GSH-dependent manner.	Deduced	3110846 0
HNF4A	Hepatocyte nuclear factor 4 alpha	Essential for stimulation of anti-ferroptotic gene transcription.	Deduced	3110846 0
NGB	Neuroglobin	Human neuroglobin (hNgb)-EGFP-expressing SH-SY5Y	Deduced	3140521

		cells to be significantly more resistant to ferroptosis induction.		3
YWHAE	Tyrosine 3-monooxygenase/tryptophan 5-monooxygenase activation protein epsilon	Required by RSL3 (a ferroptosis inducer) to inactivate GPX4 (a ferroptosis inhibitor).	Deduced	31581313
GABPB1	GA binding protein transcription factor subunit beta 1	Downregulated by lncRNA GABPB1-AS1 upon erastin treatment.	Deduced	31700067
AURKA	Aurora kinase A	Inhibition of AURKA or reconstitution of miR-4715-3p inhibited GPX4 and induced cell death, suggesting a link between AURKA and ferroptosis.	Predicted	31740746
MIR4715	microRNA 4715	Inhibition of AURKA or reconstitution of miR-4715-3p inhibited GPX4 and induced cell death, suggesting a link between AURKA and ferroptosis.	Predicted	31740746
RIPK1	Receptor interacting serine/threonine kinase 1	Reduced expression results in resistance to ferroptosis.	Deduced	31827280
PRDX1	Peroxiredoxin 1	Necessary to ferroptosis-related lipid peroxidation.	Deduced	31901729
MIR30B	microRNA 30b	Upregulation of miR-30b-5p in preeclampsia models plays a pivotal role in ferroptosis.	Deduced	31926626

Table S2. 259 genes were identified as the ferroptosis-related genes.

Gene Symbol	Description
ABCC1	ATP binding cassette subfamily C member 1
ACO1	Aconitase 1
ACSF2	Acyl-CoA synthetase family member 2
ACSL3	Acyl-CoA synthetase long chain family member 3
ACSL4	Acyl-CoA synthetase long chain family member 4
ACVR1B	Activin A receptor type 1B
AGPAT3	1-acylglycerol-3-phosphate O-acyltransferase 3
AIFM2	Apoptosis inducing factor mitochondria associated 2
AKR1C1	Aldo-keto reductase family 1 member C1
AKR1C2	Aldo-keto reductase family 1 member C2
AKR1C3	Aldo-keto reductase family 1 member C3
ALB	Albumin
ALOX12	Arachidonate 12-lipoxygenase, 12S type
ALOX12B	Arachidonate 12-lipoxygenase, 12R type
ALOX15	Arachidonate 15-lipoxygenase
ALOX15B	Arachidonate 15-lipoxygenase type B
ALOX5	Arachidonate 5-lipoxygenase
ALOXE3	Arachidonate lipoxygenase 3
ANGPTL7	Angiopoietin like 7
ANO6	Anoctamin 6
ARNTL	Aryl hydrocarbon receptor nuclear translocator like
ARRDC3	Arrestin domain containing 3
ASNS	Asparagine synthetase (glutamine-hydrolyzing)
ATF3	Activating transcription factor 3
ATF4	Activating transcription factor 4
ATG13	Autophagy related 13
ATG16L1	Autophagy related 16 like 1
ATG3	Autophagy related 3
ATG4D	Autophagy related 4D cysteine peptidase
ATG5	Autophagy related 5
ATG7	Autophagy related 7
ATM	ATM serine/threonine kinase
ATP5MC3	ATP synthase membrane subunit c locus 3
ATP6V1G2	ATPase H+ transporting V1 subunit G2
AURKA	Aurora kinase A
BACH1	BTB domain and CNC homolog 1
BAP1	BRCA1 associated protein 1
BECN1	Beclin 1
BID	BH3 interacting domain death agonist
BLOC1S5-TXNDC5	BLOC1S5-TXNDC5 readthrough (NMD candidate)
BNIP3	BCL2 interacting protein 3
BRD4	Bromodomain containing 4
CA9	Carbonic anhydrase 9
CAPG	Capping actin protein, gelsolin like
CARS1	Cysteinyl-tRNA synthetase 1

CAV1	Caveolin 1
CBS	Cystathionine beta-synthase
CD44	CD44 molecule (Indian blood group)
CDKN1A	Cyclin dependent kinase inhibitor 1A
CDKN2A	Cyclin dependent kinase inhibitor 2A
CDO1	Cysteine dioxygenase type 1
CEBPG	CCAAT enhancer binding protein gamma
CHAC1	ChaC glutathione specific gamma-glutamylcyclotransferase 1
CHMP5	Charged multivesicular body protein 5
CHMP6	Charged multivesicular body protein 6
CISD1	CDGSH iron sulfur domain 1
CISD2	CDGSH iron sulfur domain 2
CS	Citrate synthase
CXCL2	C-X-C motif chemokine ligand 2
CYBB	Cytochrome b-245 beta chain
DDIT3	DNA damage inducible transcript 3
DDIT4	DNA damage inducible transcript 4
DNAJB6	DnaJ heat shock protein family (Hsp40) member B6
DPP4	Dipeptidyl peptidase 4
DRD4	Dopamine receptor D4
DRD5	Dopamine receptor D5
DUOX1	Dual oxidase 1
DUOX2	Dual oxidase 2
DUSP1	Dual specificity phosphatase 1
EGFR	Epidermal growth factor receptor
EGLN2	Egl-9 family hypoxia inducible factor 2
EIF2AK4	Eukaryotic translation initiation factor 2 alpha kinase 4
EIF2S1	Eukaryotic translation initiation factor 2 subunit 1
ELAVL1	ELAV like RNA binding protein 1
EMC2	ER membrane protein complex subunit 2
ENPP2	Ectonucleotide pyrophosphatase/phosphodiesterase 2
EPAS1	Endothelial PAS domain protein 1
FADS2	Fatty acid desaturase 2
FANCD2	FA complementation group D2
FBXW7	F-box and WD repeat domain containing 7
Fer1HCH	Ferritin 1 Heavy Chain Homolog
FH	Fumarate hydratase
FLT3	Fms related tyrosine kinase 3
FTH1	Ferritin heavy chain 1
FTL	Ferritin light chain
FTMT	Ferritin mitochondrial
G6PD	Glucose-6-phosphate dehydrogenase
G6PDX	_NA_
GABARAPL1	GABA type A receptor associated protein like 1
GABARAPL2	GABA type A receptor associated protein like 2
GABPB1	GA binding protein transcription factor subunit beta 1
GCH1	GTP cyclohydrolase 1
GCLC	Glutamate-cysteine ligase catalytic subunit

GDF15	Growth differentiation factor 15
GLS2	Glutaminase 2
GLUT13	_NA_
GOT1	Glutamic-oxaloacetic transaminase 1
GPT2	Glutamic--pyruvic transaminase 2
GPX2	Glutathione peroxidase 2
GPX4	Glutathione peroxidase 4
HAMP	Hepcidin antimicrobial peptide
HBA1	Hemoglobin subunit alpha 1
HELLS	Helicase, lymphoid specific
HERPUD1	Homocysteine inducible ER protein with ubiquitin like domain 1
HIC1	HIC ZBTB transcriptional repressor 1
HIF1A	Hypoxia inducible factor 1 subunit alpha
HILPDA	Hypoxia inducible lipid droplet associated
HMGB1	High mobility group box 1
HMOX1	Heme oxygenase 1
HNF4A	Hepatocyte nuclear factor 4 alpha
HRAS	HRas proto-oncogene, GTPase
HSD17B11	Hydroxysteroid 17-beta dehydrogenase 11
HSF1	Heat shock transcription factor 1
HSPA5	Heat shock protein family A (Hsp70) member 5
HSPB1	Heat shock protein family B (small) member 1
IDH1	Isocitrate dehydrogenase (NADP(+)) 1
IFNG	Interferon gamma
IL33	Interleukin 33
IL6	Interleukin 6
IREB2	Iron response element binding protein 2
ISCU	Iron-sulfur cluster assembly enzyme
JDP2	Jun dimerization protein 2
JUN	Jun proto-oncogene, AP-1 transcription factor subunit
KEAP1	Kelch like ECH associated protein 1
KIM-1	Kidney injury molecule-1
KLHL24	Kelch like family member 24
KRAS	KRAS proto-oncogene, GTPase
LAMP2	Lysosomal associated membrane protein 2
LINC00336	Long intergenic non-protein coding RNA 336
LINC00472	Long intergenic non-protein coding RNA 472
LOC284561	_NA_
LOC390705	_NA_
LONP1	Lon peptidase 1, mitochondrial
LPCAT3	Lysophosphatidylcholine acyltransferase 3
LPIN1	Lipin 1
LURAP1L	Leucine rich adaptor protein 1 like
MAFG	MAF bZIP transcription factor G
MAP1LC3A	Microtubule associated protein 1 light chain 3 alpha
MAP3K5	Mitogen-activated protein kinase kinase kinase 5
MAPK1	Mitogen-activated protein kinase 1
MAPK14	Mitogen-activated protein kinase 14

MAPK3	Mitogen-activated protein kinase 3
MAPK8	Mitogen-activated protein kinase 8
MAPK9	Mitogen-activated protein kinase 9
MIOX	Myo-inositol oxygenase
MIR137	microRNA 137
MIR17	microRNA 17
MIR212	microRNA 212
MIR30B	microRNA 30b
MIR4715	microRNA 4715
MIR6852	microRNA 6852
MIR9-1	microRNA 9-1
MIR9-2	microRNA 9-2
MIR9-3	microRNA 9-3
MT1G	Metallothionein 1G
MT3	Metallothionein 3
MTDH	Metadherin
MTOR	Mechanistic target of rapamycin kinase
MUC1	Mucin 1, cell surface associated
MYB	MYB proto-oncogene, transcription factor
NCF2	Neutrophil cytosolic factor 2
NCOA4	Nuclear receptor coactivator 4
NF2	Neurofibromin 2
NFE2L2	Nuclear factor, erythroid 2 like 2
NFS1	NFS1 cysteine desulfurase
NGB	Neuroglobin
NNMT	Nicotinamide N-methyltransferase
NOS2	Nitric oxide synthase 2
NOX1	Nicotinamide adenine dinucleotide phosphate (NADPH) oxidase (NOX) 1
NOX3	Nicotinamide adenine dinucleotide phosphate (NADPH) oxidase (NOX) 3
NOX4	Nicotinamide adenine dinucleotide phosphate (NADPH) oxidase (NOX) 4
NOX5	Nicotinamide adenine dinucleotide phosphate (NADPH) oxidase (NOX) 5
NQO1	NAD(P)H quinone dehydrogenase 1
NRAS	NRAS proto-oncogene, GTPase
OTUB1	OTU deubiquitinase, ubiquitin aldehyde binding 1
OXSR1	Oxidative stress responsive kinase 1
PANX1	Pannexin 1
PCK2	Phosphoenolpyruvate carboxykinase 2, mitochondrial
PEBP1	Phosphatidylethanolamine binding protein 1
PGD	Phosphoglycerate dehydrogenase
PHKG2	Phosphorylase kinase catalytic subunit gamma 2
PIK3CA	Phosphatidylinositol-4,5-bisphosphate 3-kinase catalytic subunit alpha
PLIN2	Perilipin 2
PLIN4	Perilipin 4
PML	Promyelocytic leukemia
PRDX1	Peroxiredoxin 1
PRDX6	Peroxiredoxin 6
PRKAA1	Protein kinase AMP-activated catalytic subunit alpha 1
PRKAA2	Protein kinase AMP-activated catalytic subunit alpha 2

PROM2	Prominin 2
PSAT1	Phosphoserine aminotransferase 1
PTGS2	Prostaglandin-endoperoxide synthase 2
RB1	RB transcriptional corepressor 1
RELA	RELA proto-oncogene, NF-kB subunit
RGS4	Regulator of G protein signaling 4
RIPK1	Receptor interacting serine/threonine kinase 1
RPL8	Ribosomal protein L8
RRM2	Ribonucleotide reductase regulatory subunit M2
SAT1	Spermidine/spermine N1-acetyltransferase 1
SCD	Stearoyl-CoA desaturase
SCP2	Sterol carrier protein 2
SELENOS	Selenoprotein S
SESN2	Sestrin 2
SETD1B	SET domain containing 1B, histone lysine methyltransferase
SIRT1	Sirtuin 1
SLC1A4	Solute carrier family 1 member 4
SLC1A5	Solute carrier family 1 member 5
SLC2A1	Solute carrier family 2 member 1
SLC2A12	Solute carrier family 2 member 12
SLC2A14	Solute carrier family 2 member 14
SLC2A3	Solute carrier family 2 member 3
SLC2A6	Solute carrier family 2 member 6
SLC2A8	Solute carrier family 2 member 8
SLC38A1	Solute carrier family 38 member 1
SLC3A2	Solute carrier family 3 member 2
SLC40A1	Solute carrier family 40 member 1
SLC7A11	Solute carrier family 7 member 11
SLC7A5	Solute carrier family 7 member 5
SNORA16A	Small nucleolar RNA, H/ACA box 16A
SNX4	Sorting nexin 4
SOCS1	Suppressor of cytokine signaling 1
SP1	Sp1 transcription factor
SQSTM1	Sequestosome 1
SRC	SRC proto-oncogene, non-receptor tyrosine kinase
SRXN1	Sulfiredoxin 1
STAT3	Signal transducer and activator of transcription 3
STEAP3	STEAP3 metalloreductase
STMN1	Stathmin 1
TAZ	Tafazzin
TF	Transferrin
TFAP2C	Transcription factor AP-2 gamma
TFR2	Transferrin receptor 2
TFRC	Transferrin receptor
TGFBR1	Transforming growth factor beta receptor 1
TLR4	Toll like receptor 4
TMBIM4	Transmembrane BAX inhibitor motif containing 4
TNFAIP3	TNF alpha induced protein 3

TP53	Tumor protein p53
TP63	Tumor protein p63
TRIB3	Tribbles pseudokinase 3
TSC22D3	TSC22 domain family member 3
TUBE1	Tubulin epsilon 1
TXNIP	Thioredoxin interacting protein
TXNRD1	Thioredoxin reductase 1
UBC	Ubiquitin C
ULK1	Unc-51 like autophagy activating kinase 1
ULK2	Unc-51 like autophagy activating kinase 2
VDAC2	Valtage-dependent anion channels 2
VEGFA	Vascular endothelial growth factor A
VLDLR	Very low density lipoprotein receptor
WIPI1	WD repeat domain, phosphoinositide interacting 1
WIPI2	WD repeat domain, phosphoinositide interacting 2
XBP1	X-box binding protein 1
YWHAE	Tyrosine 3-monooxygenase/tryptophan 5-monooxygenase activation protein epsilon
YY1AP1	YY1 associated protein 1
ZEB1	Zinc finger E-box binding homeobox 1
ZFP36	ZFP36 ring finger protein
ZFP69B	ZFP69 zinc finger protein B
ZNF419	Zinc finger protein 419

Table S3. 222 autophagy-associated genes identified from the Human Autophagy Database.

Gene Symbol	Description	Entrez Gene ID
AMBRA1	autophagy/beclin-1 regulator 1	55626
APOL1	apolipoprotein L, 1	8542
ARNT	aryl hydrocarbon receptor nuclear translocator	405
ARSA	arylsulfatase A	410
ARSB	arylsulfatase B	411
ATF4	activating transcription factor 4 (tax-responsive enhancer element B67)	468
ATF6	activating transcription factor 6	22926
ATG10	ATG10 autophagy related 10 homolog (S. cerevisiae)	83734
ATG12	ATG12 autophagy related 12 homolog (S. cerevisiae)	9140
ATG16L1	ATG16 autophagy related 16-like 1 (S. cerevisiae)	55054
ATG16L2	ATG16 autophagy related 16-like 2 (S. cerevisiae)	89849
ATG2A	ATG2 autophagy related 2 homolog A (S. cerevisiae)	23130
ATG2B	ATG2 autophagy related 2 homolog B (S. cerevisiae)	55102
ATG3	ATG3 autophagy related 3 homolog (S. cerevisiae)	64422
ATG4A	ATG4 autophagy related 4 homolog A (S. cerevisiae)	115201
ATG4B	ATG4 autophagy related 4 homolog B (S. cerevisiae)	23192
ATG4C	ATG4 autophagy related 4 homolog C (S. cerevisiae)	84938
ATG4D	ATG4 autophagy related 4 homolog D (S. cerevisiae)	84971
ATG5	ATG5 autophagy related 5 homolog (S. cerevisiae)	9474
ATG7	ATG7 autophagy related 7 homolog (S. cerevisiae)	10533
ATG9A	ATG9 autophagy related 9 homolog A (S. cerevisiae)	79065
ATG9B	ATG9 autophagy related 9 homolog B (S. cerevisiae)	285973
ATIC	5-aminoimidazole-4-carboxamide ribonucleotide formyltransferase/IMP cyclohydrolase	471
BAG1	BCL2-associated athanogene	573
BAG3	BCL2-associated athanogene 3	9531
BAK1	BCL2-antagonist/killer 1	578
BAX	BCL2-associated X protein	581
BCL2	B-cell CLL/lymphoma 2	596
BCL2L1	BCL2-like 1	598
BECN1	beclin 1, autophagy related	8678

BID	BH3 interacting domain death agonist	637
BIRC5	baculoviral IAP repeat-containing 5	332
BIRC6	baculoviral IAP repeat-containing 6	57448
BNIP1	BCL2/adenovirus E1B 19kDa interacting protein 1	662
BNIP3	BCL2/adenovirus E1B 19kDa interacting protein 3	664
BNIP3L	BCL2/adenovirus E1B 19kDa interacting protein 3-like	665
C12orf44	chromosome 12 open reading frame 44	60673
C17orf88	chromosome 17 open reading frame 88	23591
CALCOCO2	calcium binding and coiled-coil domain 2	10241
CAMKK2	calcium/calmodulin-dependent protein kinase kinase 2, beta	10645
CANX	calnexin	821
CAPN1	calpain 1, (mu/I) large subunit	823
CAPN10	calpain 10	11132
CAPN2	calpain 2, (m/II) large subunit	824
CAPNS1	calpain, small subunit 1	826
CASP1	caspase 1, apoptosis-related cysteine peptidase (interleukin 1, beta, convertase)	834
CASP3	caspase 3, apoptosis-related cysteine peptidase	836
CASP4	caspase 4, apoptosis-related cysteine peptidase	837
CASP8	caspase 8, apoptosis-related cysteine peptidase	841
CCL2	chemokine (C-C motif) ligand 2	6347
CCR2	chemokine (C-C motif) receptor 2	729230
CD46	CD46 molecule, complement regulatory protein	4179
CDKN1A	cyclin-dependent kinase inhibitor 1A (p21, Cip1)	1026
CDKN1B	cyclin-dependent kinase inhibitor 1B (p27, Kip1)	1027
CDKN2A	cyclin-dependent kinase inhibitor 2A (melanoma, p16, inhibits CDK4)	1029
CFLAR	CASP8 and FADD-like apoptosis regulator	8837
CHMP2B	chromatin modifying protein 2B	25978
CHMP4B	chromatin modifying protein 4B	128866
CLN3	ceroid-lipofuscinosis, neuronal 3	1201
CTSB	cathepsin B	1508
CTSD	cathepsin D	1509
CTSL1	cathepsin L1	1514

CX3CL1	chemokine (C-X3-C motif) ligand 1	6376
CXCR4	chemokine (C-X-C motif) receptor 4	7852
DAPK1	death-associated protein kinase 1	1612
DAPK2	death-associated protein kinase 2	23604
DDIT3	DNA-damage-inducible transcript 3	1649
DIRAS3	DIRAS family, GTP-binding RAS-like 3	9077
DLC1	deleted in liver cancer 1	10395
DNAJB1	DnaJ (Hsp40) homolog, subfamily B, member 1	3337
DNAJB9	DnaJ (Hsp40) homolog, subfamily B, member 9	4189
DRAM1	DNA-damage regulated autophagy modulator 1	55332
EDEM1	ER degradation enhancer, mannosidase alpha-like 1	9695
EEF2	eukaryotic translation elongation factor 2	1938
EEF2K	eukaryotic elongation factor-2 kinase	29904
EGFR	epidermal growth factor receptor (erythroblastic leukemia viral (v-erb-b) oncogene homolog, avian)	1956
EIF2AK2	eukaryotic translation initiation factor 2-alpha kinase 2	5610
EIF2AK3	eukaryotic translation initiation factor 2-alpha kinase 3	9451
EIF2S1	eukaryotic translation initiation factor 2, subunit 1 alpha, 35kDa	1965
EIF4EBP1	eukaryotic translation initiation factor 4E binding protein 1	1978
EIF4G1	eukaryotic translation initiation factor 4 gamma, 1	1981
ERBB2	v-erb-b2 erythroblastic leukemia viral oncogene homolog 2, neuro/glioblastoma derived oncogene homolog (avian)	2064
ERN1	endoplasmic reticulum to nucleus signaling 1	2081
ERO1L	ERO1-like (S. cerevisiae)	30001
FADD	Fas (TNFRSF6)-associated via death domain	8772
FAM48A	family with sequence similarity 48, member A	55578
FAS	Fas (TNF receptor superfamily, member 6)	355
FKBP1A	FK506 binding protein 1A, 12kDa	2280
FKBP1B	FK506 binding protein 1B, 12.6 kDa	2281
FOS	FBJ murine osteosarcoma viral oncogene homolog	2353
FOXO1	forkhead box O1	2308
FOXO3	forkhead box O3	2309
GAA	glucosidase, alpha; acid	2548

GABARAP	GABA(A) receptor-associated protein	11337
GABARAPL1	GABA(A) receptor-associated protein like 1	23710
GABARAPL2	GABA(A) receptor-associated protein-like 2	11345
GAPDH	glyceraldehyde-3-phosphate dehydrogenase	2597
GNAI3	guanine nucleotide binding protein (G protein), alpha inhibiting activity polypeptide 3	2773
GNB2L1	guanine nucleotide binding protein (G protein), beta polypeptide 2-like 1	10399
GOPC	golgi-associated PDZ and coiled-coil motif containing	57120
GRID1	glutamate receptor, ionotropic, delta 1	2894
GRID2	glutamate receptor, ionotropic, delta 2	2895
HDAC1	histone deacetylase 1	3065
HDAC6	histone deacetylase 6	10013
HGS	hepatocyte growth factor-regulated tyrosine kinase substrate	9146
HIF1A	hypoxia inducible factor 1, alpha subunit (basic helix-loop-helix transcription factor)	3091
HSP90AB1	heat shock protein 90kDa alpha (cytosolic), class B member 1	3326
HSPA5	heat shock 70kDa protein 5 (glucose-regulated protein, 78kDa)	3309
HSPA8	heat shock 70kDa protein 8	3312
HSPB8	heat shock 22kDa protein 8	26353
IFNG	interferon, gamma	3458
IKBKB	inhibitor of kappa light polypeptide gene enhancer in B-cells, kinase beta	3551
IKBKE	inhibitor of kappa light polypeptide gene enhancer in B-cells, kinase epsilon	9641
IL24	interleukin 24	11009
IRGM	immunity-related GTPase family, M	345611
ITGA3	integrin, alpha 3 (antigen CD49C, alpha 3 subunit of VLA-3 receptor)	3675
ITGA6	integrin, alpha 6	3655
ITGB1	integrin, beta 1 (fibronectin receptor, beta polypeptide, antigen CD29 includes MDF2, MSK12)	3688
ITGB4	integrin, beta 4	3691
ITPR1	inositol 1,4,5-triphosphate receptor, type 1	3708
KIAA0226	KIAA0226	9711
KIAA0652	KIAA0652	9776
KIAA0831	KIAA0831	22863
KIF5B	kinesin family member 5B	3799
KLHL24	kelch-like 24 (Drosophila)	54800

LAMP1	lysosomal-associated membrane protein 1	3916
LAMP2	lysosomal-associated membrane protein 2	3920
MAP1LC3A	microtubule-associated protein 1 light chain 3 alpha	84557
MAP1LC3B	microtubule-associated protein 1 light chain 3 beta	81631
MAP1LC3C	microtubule-associated protein 1 light chain 3 gamma	440738
MAP2K7	mitogen-activated protein kinase kinase 7	5609
MAPK1	mitogen-activated protein kinase 1	5594
MAPK3	mitogen-activated protein kinase 3	5595
MAPK8	mitogen-activated protein kinase 8	5599
MAPK8IP1	mitogen-activated protein kinase 8 interacting protein 1	9479
MAPK9	mitogen-activated protein kinase 9	5601
MBTPS2	membrane-bound transcription factor peptidase, site 2	51360
MLST8	MTOR associated protein, LST8 homolog (S. cerevisiae)	64223
MTMR14	myotubularin related protein 14	64419
MTOR	mechanistic target of rapamycin (serine/threonine kinase)	2475
MYC	v-myc myelocytomatosis viral oncogene homolog (avian)	4609
NAF1	nuclear assembly factor 1 homolog (S. cerevisiae)	92345
NAMPT	nicotinamide phosphoribosyltransferase	10135
NBR1	neighbor of BRCA1 gene 1	4077
NCKAP1	NCK-associated protein 1	10787
NFE2L2	nuclear factor (erythroid-derived 2)-like 2	4780
NFKB1	nuclear factor of kappa light polypeptide gene enhancer in B-cells 1	4790
NKX2-3	NK2 transcription factor related, locus 3 (Drosophila)	159296
NLRC4	NLR family, CARD domain containing 4	58484
NPC1	Niemann-Pick disease, type C1	4864
NRG1	neuregulin 1	3084
NRG2	neuregulin 2	9542
NRG3	neuregulin 3	10718
P4HB	prolyl 4-hydroxylase, beta polypeptide	5034
PARK2	Parkinson disease (autosomal recessive, juvenile) 2, parkin	5071
PARP1	poly (ADP-ribose) polymerase 1	142
PEA15	phosphoprotein enriched in astrocytes 15	8682

PELP1	proline, glutamate and leucine rich protein 1	27043
PEX14	peroxisomal biogenesis factor 14	5195
PEX3	peroxisomal biogenesis factor 3	8504
PIK3C3	phosphoinositide-3-kinase, class 3	5289
PIK3R4	phosphoinositide-3-kinase, regulatory subunit 4	30849
PINK1	PTEN induced putative kinase 1	65018
PPP1R15A	protein phosphatase 1, regulatory (inhibitor) subunit 15A	23645
PRKAB1	protein kinase, AMP-activated, beta 1 non-catalytic subunit	5564
PRKAR1A	protein kinase, cAMP-dependent, regulatory, type I, alpha (tissue specific extinguisher 1)	5573
PRKCD	protein kinase C, delta	5580
PRKCQ	protein kinase C, theta	5588
PTEN	phosphatase and tensin homolog	5728
PTK6	PTK6 protein tyrosine kinase 6	5753
RAB11A	RAB11A, member RAS oncogene family	8766
RAB1A	RAB1A, member RAS oncogene family	5861
RAB24	RAB24, member RAS oncogene family	53917
RAB33B	RAB33B, member RAS oncogene family	83452
RAB5A	RAB5A, member RAS oncogene family	5868
RAB7A	RAB7A, member RAS oncogene family	7879
RAC1	ras-related C3 botulinum toxin substrate 1 (rho family, small GTP binding protein Rac1)	5879
RAF1	v-raf-1 murine leukemia viral oncogene homolog 1	5894
RB1	retinoblastoma 1	5925
RB1CC1	RB1-inducible coiled-coil 1	9821
RELA	v-rel reticuloendotheliosis viral oncogene homolog A (avian)	5970
RGS19	regulator of G-protein signaling 19	10287
RHEB	Ras homolog enriched in brain	6009
RPS6KB1	ribosomal protein S6 kinase, 70kDa, polypeptide 1	6198
RPTOR	regulatory associated protein of MTOR, complex 1	57521
SAR1A	SAR1 homolog A (S. cerevisiae)	56681
SERPINA1	serpin peptidase inhibitor, clade A (alpha-1 antiproteinase, antitrypsin), member 1	5265
SESN2	sestrin 2	83667
SH3GLB1	SH3-domain GRB2-like endophilin B1	51100

SIRT1	sirtuin (silent mating type information regulation 2 homolog) 1 (S. cerevisiae)	23411
SIRT2	sirtuin (silent mating type information regulation 2 homolog) 2 (S. cerevisiae)	22933
SPHK1	sphingosine kinase 1	8877
SPNS1	spinster homolog 1 (Drosophila)	83985
SQSTM1	sequestosome 1	8878
ST13	suppression of tumorigenicity 13 (colon carcinoma) (Hsp70 interacting protein)	6767
STK11	serine/threonine kinase 11	6794
TBK1	TANK-binding kinase 1	29110
TM9SF1	transmembrane 9 superfamily member 1	10548
TMEM49	transmembrane protein 49	81671
TMEM74	transmembrane protein 74	157753
TNFSF10	tumor necrosis factor (ligand) superfamily, member 10	8743
TP53	tumor protein p53	7157
TP53INP2	tumor protein p53 inducible nuclear protein 2	58476
TP63	tumor protein p63	8626
TP73	tumor protein p73	7161
TSC1	tuberous sclerosis 1	7248
TSC2	tuberous sclerosis 2	7249
TUSC1	tumor suppressor candidate 1	286319
ULK1	unc-51-like kinase 1 (C. elegans)	8408
ULK2	unc-51-like kinase 2 (C. elegans)	9706
ULK3	unc-51-like kinase 3 (C. elegans)	25989
USP10	ubiquitin specific peptidase 10	9100
UVRAG	UV radiation resistance associated gene	7405
VAMP3	vesicle-associated membrane protein 3 (cellubrevin)	9341
VAMP7	vesicle-associated membrane protein 7	6845
VEGFA	vascular endothelial growth factor A	7422
WDFY3	WD repeat and FYVE domain containing 3	23001
WDR45	WD repeat domain 45	11152
WDR45L	WDR45-like	56270
WIPI1	WD repeat domain, phosphoinositide interacting 1	55062
WIPI2	WD repeat domain, phosphoinositide interacting 2	26100

ZFYVE1 zinc finger, FYVE domain containing 1 53349

Table S4. 4138 genes were significantly predicting the prognosis of LUAD patients by both Kaplan-Meier and univariate Cox regression analyses (p-value < 0.05).

Gene Symbol	Kaplan-Meier analysis (p-value)	Univariate Cox regression analysis			
		HR	HR_95L	HR_95H	P-value
ZWINT	0.010076308	1.248155849	1.083554449	1.437761641	0.002125476
ZWILCH	0.007850071	1.468952609	1.140548487	1.891915857	0.002895782
ZSCAN32	0.001277416	0.307179202	0.126693033	0.744784936	0.009000308
ZSCAN30	0.005548986	0.680821577	0.495462405	0.935526117	0.017741039
ZSCAN26	0.003196696	0.580375829	0.418565421	0.804739439	0.001103724
ZSCAN16-AS1	0.004720665	0.668947832	0.504029553	0.887827309	0.005373293
ZRANB2-AS2	0.000381703	0.018741332	0.001419123	0.247503314	0.002524089
ZPR1	0.026075323	1.41684603	1.004743908	1.997974465	0.046927682
ZNF91	0.041961827	0.663990672	0.482686666	0.913395053	0.011845593
ZNF90P1	0.009361041	0.001837136	5.05E-06	0.668570487	0.036279528
ZNF90	0.01265276	0.807399958	0.65936034	0.988677439	0.038438144
ZNF879	0.013194002	0.543399719	0.366071507	0.806627254	0.002476424
ZNF835	0.001775852	0.486134879	0.286569332	0.824676945	0.007476761
ZNF831	0.004050771	0.420886365	0.237248707	0.746665114	0.003088494
ZNF821	0.040425757	0.549456443	0.34306318	0.880019774	0.0127096
ZNF80	0.020489231	0.262220352	0.070251662	0.978759949	0.046380761
ZNF791	0.003589807	0.430264683	0.27511997	0.672898073	0.000218795
ZNF79	0.000564968	0.554273908	0.35237372	0.8718572	0.010670233
ZNF785	0.024207389	0.663032969	0.478926551	0.917912604	0.01328383
ZNF780B	0.02483875	0.587305391	0.392776339	0.878178212	0.009518593
ZNF778	0.027080219	0.574399662	0.383202239	0.860994373	0.007259679
ZNF77	0.000486661	0.444496803	0.292987957	0.674353343	0.000137475
ZNF75D	0.004923222	0.590185845	0.423315276	0.822836669	0.001870722
ZNF737	0.024881955	0.683932994	0.54078605	0.864971165	0.001521034
ZNF736P9Y	0.014185689	2.566580422	1.546879827	4.258465946	0.000263674
ZNF709	0.001441379	0.023540505	0.001496557	0.370286774	0.007662235
ZNF708	0.012902212	0.515388512	0.352110284	0.754381028	0.000649621
ZNF704	0.026916438	0.762237781	0.615245755	0.944348546	0.012998449

ZNF697	0.002127697	1.472556908	1.137041184	1.907075908	0.003352094
ZNF691	0.008077265	0.576559746	0.404479461	0.821849246	0.002328545
ZNF69	0.023854734	0.686499635	0.515395259	0.91440839	0.010119412
ZNF684	0.02771407	0.564519663	0.353228523	0.902199085	0.016839281
ZNF674-AS1	0.001167523	0.604661428	0.438118663	0.834512367	0.002209606
ZNF671	0.009481218	0.684985843	0.520255825	0.901874776	0.007021035
ZNF662	0.015846758	0.706112136	0.524146291	0.95125036	0.02209861
ZNF660	0.038120107	0.499332679	0.269134531	0.926425621	0.027643561
ZNF658	0.038171209	0.348540276	0.156917326	0.774167691	0.009636265
ZNF641	0.031629323	0.658828286	0.466871409	0.929709342	0.017561871
ZNF608	0.047336675	0.787288424	0.635081703	0.975973737	0.02912221
ZNF598	0.021493077	1.328645427	1.019348646	1.731790863	0.035579251
ZNF596	0.004059715	0.521326673	0.294002215	0.924419907	0.025821999
ZNF589	0.012363868	0.605313553	0.450226044	0.813823417	0.000887088
ZNF586	0.006509148	0.609060354	0.414833083	0.894225967	0.011389302
ZNF582-AS1	0.008658013	0.502434132	0.314540827	0.802566902	0.003972038
ZNF564	0.028499266	0.127874955	0.028838861	0.567012821	0.006796997
ZNF563	0.028386251	0.409565374	0.245340247	0.683719031	0.000639832
ZNF559-ZNF177	0.001287155	0.148511992	0.025738405	0.85692224	0.032954819
ZNF557	0.010262205	0.42113422	0.254348956	0.697286257	0.00077539
ZNF555	0.000146148	0.177704028	0.081412715	0.387884396	1.44E-05
ZNF554	0.013459419	0.473674998	0.28261325	0.793904757	0.004570413
ZNF552	0.017025961	0.701325316	0.545494333	0.901672427	0.005652468
ZNF540	0.003261327	0.365192753	0.219775261	0.606827839	0.000101133
ZNF516	0.00524388	0.752434891	0.594554239	0.952239894	0.017920741
ZNF510	0.004380393	0.574677585	0.353791027	0.933472875	0.02521373
ZNF506	0.005729337	0.511915549	0.36341692	0.721093363	0.000127853
ZNF502	0.020545738	0.660260016	0.515948049	0.844936404	0.000970317
ZNF493	0.000476188	0.502271579	0.348391569	0.724118382	0.000224722
ZNF491	0.030990478	0.507472499	0.271688124	0.947882203	0.033347659
ZNF490	0.003343376	0.203542152	0.068294589	0.606627965	0.004275843
ZNF451	0.002775179	0.490140708	0.305508303	0.786354777	0.003111654

ZNF442	0.01826145	0.327643908	0.126707184	0.84723318	0.021336914
ZNF441	0.012797168	0.449037727	0.294659034	0.684298994	0.000195418
ZNF44	0.027032431	0.457304169	0.308965714	0.676861846	9.20E-05
ZNF439	0.031306124	0.594994705	0.421310105	0.840280581	0.003197825
ZNF431	0.036736111	0.700914253	0.528749257	0.92913755	0.013472507
ZNF43	0.000829427	0.652927478	0.502111665	0.849042795	0.001466858
ZNF429	0.004783961	0.527086013	0.372293523	0.746238244	0.000306145
ZNF41	0.03040284	0.601227145	0.404098515	0.894519694	0.012078547
ZNF397	0.026864576	0.725047437	0.549904194	0.955973407	0.022659218
ZNF396	0.017522818	0.380883972	0.224590693	0.645942172	0.000341446
ZNF367	0.005766994	1.342602217	1.079255464	1.670207633	0.00817798
ZNF354C	0.020668113	0.661246143	0.469741154	0.93082426	0.01774731
ZNF347	0.043998522	0.630024876	0.422457036	0.939578017	0.023475946
ZNF330	0.007242717	0.717578636	0.5491324	0.937695715	0.015047636
ZNF322	0.028266804	0.743584853	0.589899092	0.937310196	0.012141298
ZNF3	0.018600314	0.67850796	0.480094403	0.958921932	0.02797548
ZNF287	0.007296392	0.573929167	0.399472042	0.824575074	0.002671024
ZNF266	0.000326758	0.62766717	0.485396943	0.811636913	0.000383294
ZNF254	0.001280108	0.621843185	0.484112894	0.798757793	0.000200046
ZNF253	0.023659429	0.576310809	0.428417972	0.775257273	0.000270085
ZNF25	0.000926555	0.520342914	0.371431367	0.72895499	0.000145897
ZNF248	0.004460066	0.553079174	0.382727766	0.799253673	0.001617011
ZNF24	0.044155648	0.72091663	0.532937928	0.975199474	0.033763604
ZNF214	0.003823822	0.680523257	0.479924817	0.96496761	0.030764981
ZNF205-AS1	0.027280428	0.398234579	0.190549894	0.832279548	0.014360887
ZNF204P	0.013369448	0.8252671	0.69942351	0.973753064	0.022903285
ZNF184	0.024594811	0.644117716	0.442468063	0.937666844	0.021682211
ZNF182	0.000309824	0.602940869	0.423171672	0.859078516	0.005096804
ZNF17	0.046120847	0.566930511	0.325494915	0.987450771	0.045008989
ZNF154	0.010319673	0.655908271	0.476284085	0.903275321	0.009793873
ZNF146	0.0327119	1.356875873	1.078679094	1.706821004	0.009136106
ZNF141	0.006354141	0.518666669	0.333770258	0.80598887	0.003511964

ZNF14	0.028635878	0.525411239	0.381001319	0.72455647	8.68E-05
ZNF136	0.003957811	0.470519707	0.30950529	0.715298903	0.000419076
ZNF101	0.000163719	0.539624426	0.370108098	0.786782354	0.001344039
ZNF100	0.013360814	0.618744527	0.446699564	0.857052077	0.003877851
ZNF10	0.048494027	0.560025889	0.392062266	0.799946906	0.001438006
ZMYND15	0.013768797	0.631572192	0.479429758	0.831995569	0.001083403
ZMYND12	0.017448443	0.702027824	0.549158223	0.897451853	0.004751018
ZMYM6	0.012418048	0.421303356	0.238931519	0.742876111	0.002816617
ZMAT1	0.038119276	0.721739396	0.583061082	0.893401689	0.002741192
ZKSCAN4	0.000345036	0.555229832	0.380853918	0.80944465	0.002219781
ZIC5	0.004991548	2.004460286	1.365881238	2.941588864	0.000380609
ZIC2	0.036477591	1.328651151	1.095088973	1.612027813	0.003965986
ZFP3	0.000581567	0.540187884	0.402090797	0.725714073	4.35E-05
ZFP2	0.017834828	0.436538168	0.256487297	0.742982495	0.002251522
ZFAND4	0.012912507	0.421457691	0.252030952	0.704780837	0.000989014
ZFAND2B	0.032928672	0.697761346	0.488365439	0.996939703	0.048064255
ZEB2-AS1	0.001888445	0.047252495	0.007756637	0.287856486	0.000930613
ZEB2	0.000406332	0.749339965	0.583292123	0.962657236	0.023962429
ZDHHC5	0.044907571	1.655235911	1.093962255	2.504479391	0.017079835
ZDHHC21	0.042004381	0.766327867	0.61469967	0.955358248	0.017984078
ZDHHC16	0.031443689	0.718523256	0.539209956	0.95746687	0.024027406
ZC3H12D	0.000279996	0.429117508	0.28349636	0.649538624	6.33E-05
ZBTB8A	0.032893803	0.540010036	0.313773832	0.929366344	0.026120867
ZBTB5	0.004862952	0.701183285	0.502027035	0.979345663	0.037306518
ZBTB49	0.017690976	0.55399174	0.343983264	0.892214478	0.015139734
ZBTB4	0.048334404	0.761431917	0.600469853	0.965541503	0.024489522
ZBTB18	0.007956821	0.771468351	0.662849592	0.897886073	0.000804714
ZAP70	0.008764393	0.770881191	0.630330003	0.942772528	0.011284387
Z83851.4	0.041059712	1.70163259	1.294793419	2.236305366	0.000137207
YWHAZP6	0.037223408	1.742279188	1.092996863	2.777260274	0.019608699
YWHAZ	0.003425391	1.59557171	1.239110272	2.054578305	0.000292437
YWHAG	0.000117489	1.662334469	1.291427847	2.139767927	7.97E-05

YWHAE	0.045167941	1.435374434	1.046237865	1.969246034	0.025083237
YPEL5P2	0.002869966	0.285396615	0.093971159	0.866768365	0.026950638
YPEL5	0.008716463	0.715703032	0.528777399	0.968707874	0.030325102
YPEL1	0.033344188	0.646187279	0.459599897	0.908525005	0.012012094
YKT6	0.003123671	1.523118026	1.175455769	1.973607671	0.00145861
YARS2	0.017997434	1.535446396	1.170021426	2.015002105	0.001986374
XXYLT1	0.048126115	1.383111776	1.077575407	1.775280107	0.010878009
XXbac-BPGBPG55C20.2	0.000177439	0.256186996	0.106815625	0.61443985	0.002279528
XXbac-BPG34I8.3	0.005111398	2.872941321	1.284812203	6.424123164	0.010159602
XXbac-BPG299F13.14	0.04735443	0.654525736	0.429691492	0.997003542	0.048388552
XXbac-BPG283O16.9	0.016930248	0.766337444	0.589980329	0.995411287	0.046104918
XXbac-BPG252P9.10	0.004594735	1.499790919	1.06369781	2.114672775	0.02076488
XXbac-BPG181B23.7	0.020918047	0.746817292	0.587203205	0.949817819	0.017329957
XXbac-BPG16N22.5	0.047248213	142030.7376	249.9025845	80722376.12	0.000246347
XXbac-BPG13B8.10	0.01389407	0.406007857	0.176916541	0.931752221	0.033441019
XXbac-B444P24.13	0.004577388	0.536008805	0.308744193	0.930561435	0.026714601
XX-FW80269A6.1	0.00167007	0.003649243	1.42E-05	0.940178836	0.047508145
XX-C2158C12.1	1.84E-08	2.19E+25	88799308345	5.38E+39	0.000558388
XRCC5	0.011601889	1.801297672	1.281241219	2.5324453	0.000709821
XRCC2	0.02346973	1.265788704	1.025965225	1.561671881	0.027868056
XKRYP2	0.011697278	1914389475	4.274529501	8.57378E+17	0.03547493
XIRP1	0.017584511	1.668707728	1.088744689	2.557611079	0.018762563
XCR1	0.011044912	0.450060196	0.277673058	0.729470052	0.001194704
WWC2-AS2	0.004867364	3.997128828	1.86486967	8.567375579	0.000367923
WT1-AS	0.034010102	2.087677877	1.049337209	4.15347791	0.035977726
WSB1	0.030069634	0.830742005	0.721223679	0.956890765	0.010143493
WNT7A	0.041409836	1.239853199	1.069532806	1.437296685	0.004350485
WFDC3	0.036009354	0.874489603	0.777297454	0.983834518	0.025675555
WFDC2	0.020191687	0.856700801	0.785129293	0.934796687	0.000511274
WDTC1	0.010525042	0.706142118	0.537518815	0.927663696	0.012443048
WDSUB1	0.034730611	0.708124054	0.50303025	0.996838016	0.047915699
WDR91	0.013690437	0.706872184	0.564986366	0.884389987	0.002407649

WDR86-AS1	0.016292176	0.853588173	0.767624558	0.949178556	0.00346634
WDR76	0.005920215	1.338135979	1.079528191	1.658694893	0.007852038
WDR47	0.020334242	0.641058801	0.457915097	0.897451054	0.00958981
WDR4	0.001503623	1.607574496	1.200507958	2.152668579	0.001439253
WDR37	0.01847378	0.595430701	0.417269692	0.849660848	0.004262648
WDR3	0.03448366	1.451118125	1.082066638	1.94603894	0.012892052
WDR19	0.017275231	0.590202732	0.420630252	0.828136501	0.00227951
WDR1	0.037383129	1.436586432	1.009214892	2.044936705	0.044338177
WDHD1	0.017000316	1.415826581	1.166297608	1.718742192	0.000439579
WDFY4	0.001383491	0.723573751	0.586152274	0.893213243	0.002605258
WBP2NL	0.002914458	0.255451904	0.090949022	0.717497271	0.009597036
WBP11	0.042223984	1.544778825	1.143834495	2.086264778	0.0045612
WASH4P	0.018592874	0.467354565	0.259076657	0.843072052	0.011501903
WASF3	0.004664848	0.764957909	0.608249411	0.962040557	0.021972828
VWA2	0.016897674	0.793432099	0.684636382	0.919516568	0.002104571
VSTM5	0.020765376	1.70902969	1.095735363	2.665591147	0.018123098
VPS13D	0.005532491	0.700793062	0.532890537	0.921598119	0.01095249
VPREB3	0.017894029	0.858634457	0.745145192	0.989408692	0.035102387
VN1R91P	0.018650677	173.6720685	1.593584411	18927.13506	0.031189105
VN1R8P	0.034696189	1.29E+27	329792507.9	5.03E+45	0.004263656
VN1R83P	0.026478251	0.632449766	0.45583402	0.877496389	0.006104481
VN1R78P	0.007663054	0.00017369	1.55E-07	0.195097695	0.015692724
VMAC	0.026894174	0.597746889	0.408159867	0.875395578	0.008201816
VIPR1	0.006652057	0.703255254	0.573822254	0.861883534	0.000693309
VIM-AS1	0.00050274	0.651780375	0.520615969	0.815990447	0.00018864
VENTX	0.012277954	0.664713631	0.447191196	0.988043179	0.043441074
VEGFC	0.011221481	1.373019086	1.201386191	1.569171866	3.27E-06
VDAC2	0.007902772	1.592458648	1.213269074	2.090158398	0.000798918
VDAC1P2	0.000230803	2.558766933	1.244223899	5.262146322	0.010650701
VDAC1	0.000469523	1.876091119	1.439630613	2.444875687	3.21E-06
VAX1	0.000977385	5.278011946	3.010301452	9.254026733	6.37E-09
VANGL1	0.001474443	1.67572544	1.277017239	2.198917653	0.000196257

VAMP8	0.020954418	0.740254758	0.591333435	0.926680405	0.008679882
USP9YP8	0.003525384	49143.36011	1.848603262	1306429504	0.03769397
USP9YP3	0.017435332	3.72262E+11	871703.584	1.58975E+17	5.63E-05
USP9YP24	5.50E-10	1.81E+59	1.57E+27	2.09E+91	0.000291895
USP9YP20	6.69E-07	2.34E+38	1.49885E+15	3.64E+61	0.001185106
USP54	0.001684866	0.795358908	0.644150387	0.982062272	0.033319723
USP5	0.035592305	1.503278804	1.117175383	2.022822196	0.007111853
USP44	0.019316509	0.729174485	0.563511817	0.943539095	0.016308382
USP4	2.77E-05	0.488174876	0.347222325	0.686346159	3.71E-05
USP20	0.00112023	0.696714972	0.514487559	0.943485888	0.01949078
USP17L8	0.000260958	8.86E+38	9.89707E+14	7.93E+62	0.001437407
USF1P1	0.02518575	0.033301225	0.001334234	0.831166941	0.038207527
UQCRFS1P2	0.010638209	3.03E-05	4.57E-09	0.200501679	0.020452126
UQCRFS1P1	0.029864678	2.425345819	1.221784344	4.814517692	0.011323401
UPK1B	0.004131041	1.151884575	1.068210409	1.242113036	0.000237987
UNC45B	0.033501167	0.014277176	0.00114044	0.178736093	0.000983127
UNC13B	0.000216597	0.771897618	0.665524772	0.895272359	0.000620998
UMODL1-AS1	0.00566639	0.552776358	0.336563619	0.907886903	0.019196399
UGT2B28	0.045914604	2.043241496	1.321806676	3.158431475	0.001302224
UGDH-AS1	4.66E-05	0.3899032	0.241760986	0.628821497	0.000112302
UCP2	0.047671163	0.834832643	0.702710939	0.991795493	0.040006144
UCK2	0.000577045	1.348553105	1.158686123	1.569532456	0.000112307
UCA1	0.014645644	1.184473848	1.073822673	1.306526984	0.000716038
UBXN11	0.008131036	0.765097894	0.627662233	0.932627066	0.008039677
UBTD1	0.009347616	0.767059738	0.635813382	0.925398329	0.005610496
UBR2	0.031241993	0.680900414	0.477592572	0.970754993	0.033670774
UBQLN1P1	0.043717697	1.46355E+12	1.24330378	1.72E+24	0.048231768
UBE2V2	0.031196009	1.403093821	1.069519083	1.840707943	0.014477169
UBE2T	0.004512765	1.205715773	1.05412035	1.379112474	0.006356943
UBE2SP1	0.002126336	1.572626383	1.209789184	2.044284882	0.000716948
UBE2S	0.012432895	1.311293253	1.127795283	1.524647266	0.000425753
UBE2MP1	0.001038051	1.97610197	1.346717944	2.899626467	0.000498703

UBE2HP1	0.048591053	4.203494343	1.183465128	14.93019462	0.026388698
UBE2H	0.018278888	1.43012697	1.079534004	1.894579645	0.012655817
UBE2FP3	0.009399706	0.528275883	0.343969535	0.811337577	0.00355692
UBE2E3	0.034486007	1.437044516	1.10819211	1.863482805	0.006241878
UBE2C	0.036479194	1.160711037	1.05461661	1.277478562	0.002309194
UBASH3A	0.036148935	0.695666231	0.516845226	0.936356728	0.016677807
UBALD2	0.009066175	1.349222724	1.054657846	1.726059278	0.017152648
U91328.19	0.001876678	0.418089882	0.261270901	0.669034129	0.000277408
U62631.5	0.016664684	0.570706227	0.349944232	0.930735723	0.024601312
TYRP1	0.0014576	0.747186062	0.578055769	0.965801295	0.026033848
TYMS	0.002123761	1.340003657	1.148940037	1.562840307	0.000192312
TXNP4	0.038429053	1.819060787	1.233254604	2.683129773	0.002550989
TXNDC5	0.01143651	0.791652313	0.630997843	0.993210028	0.043504201
TXNDC15	0.005957687	0.683361799	0.508886188	0.917657739	0.011365009
TXNDC11	0.000281774	0.622009338	0.482973491	0.801070087	0.000234772
TXLNB	0.011740157	0.629763893	0.406877309	0.974747305	0.038012327
TWF1	0.002566668	1.346863982	1.084221602	1.673128983	0.007132352
TUSC2	0.035920377	0.584463971	0.388335772	0.879646322	0.010031474
TUBG1	0.001926231	1.252297476	1.019588004	1.538120262	0.031960361
TUBB8P6	0.01109048	124936.0972	1.520088968	10268496592	0.042103983
TUBA4A	0.003551371	1.258290071	1.052392156	1.504471403	0.011732591
TUBA1C	0.00352913	1.40057816	1.136213859	1.726452435	0.001596885
TUBA1B	0.00972877	1.335656821	1.094316604	1.630222128	0.004422283
TTTY4C	0.002109365	4.41E+38	2.08515E+16	9.32E+60	0.000692208
TTTY4	0.002391955	1.54E+74	4.10449E+13	5.75E+134	0.016375038
TTLL2	0.000737742	0.34403513	0.132784315	0.891371624	0.028041358
TTLL12	0.013201781	1.375299408	1.117939474	1.691905963	0.002572896
TTLL10	0.007249023	0.632190907	0.423796689	0.943059144	0.02462263
TTK	0.000391439	1.254481101	1.070391202	1.470231472	0.005108957
TTC39C-AS1	0.009453999	0.598698147	0.379348458	0.944881843	0.027560318
TTC39B	0.009820516	0.728726876	0.547409961	0.970100834	0.030165095
TTC3-AS1	0.013494503	0.168669561	0.038817859	0.732895163	0.017569976

TTC23L	0.003931474	0.326514183	0.137637338	0.774582777	0.011101023
TTC21A	0.012613149	0.606944292	0.423494247	0.869861576	0.006543251
TTC19	0.016153606	0.669083136	0.498468114	0.898096046	0.007460115
TTC16	0.007480801	0.454274323	0.240282595	0.858843565	0.015171871
TSPYL4	0.002177709	0.71956655	0.548901614	0.943294768	0.017191622
TSPYL2	0.014906319	0.712316881	0.566435081	0.895769622	0.003715131
TSPAN7	0.017711705	0.865860193	0.77419639	0.968376866	0.0116421
TSPAN32	0.043487788	0.515415099	0.336445026	0.789587313	0.00232277
TSPAN11	0.002498506	0.830930849	0.73557523	0.938647807	0.002901145
TSNARE1	0.001659203	0.731259442	0.584035826	0.915595152	0.006357157
TSKU	0.028402616	1.161719502	1.017979186	1.325756184	0.026122384
TSC22D2	0.010395141	1.326269718	1.059906109	1.659572816	0.01356423
TRPC2	0.002039636	0.316023107	0.11589006	0.86177023	0.024409691
TROAP	0.021175191	1.171378337	1.027896772	1.334888137	0.017659627
TRMT10A	0.000542558	1.55713907	1.115553643	2.173523523	0.009251582
TRIP13	0.003552683	1.175426574	1.026963555	1.345352154	0.018967331
TRIP10	0.0233055	1.399977696	1.078807833	1.816762437	0.011390679
TRIM7	0.017014408	1.664871922	1.263164013	2.194329863	0.000296659
TRIM62	0.005532138	0.592335004	0.414136971	0.847209454	0.004130301
TRIM60P5Y	2.83E-06	1.26E+34	6.43102E+12	2.45E+55	0.001694814
TRIM6-TRIM34	0.031670931	3.16884E+14	74.37418659	1.35E+27	0.024424243
TRIM6	0.008326986	1.439346942	1.190868644	1.739670978	0.000165475
TRIM39	0.001822979	0.631043057	0.425775264	0.935271195	0.021830282
TRIM38	0.022256907	0.604624339	0.437719186	0.835171506	0.002267016
TRIM28	0.031877464	1.614798213	1.242575433	2.098523116	0.000337675
TRIM24	0.01410519	0.699396424	0.541911176	0.902648588	0.00601732
TRIM22	0.04800631	0.808646313	0.69192653	0.945055336	0.007573632
TRIM15	0.00430571	1.233300803	1.031928558	1.473969161	0.02113743
TRGV6	0.041373971	0.37002815	0.148805412	0.920133415	0.032431246
TRGV5P	0.01471867	0.193444103	0.055810357	0.670495992	0.009590461
TRG-AS1	0.030677327	0.59256471	0.396737007	0.885052137	0.010572613
TREML1	0.002254504	0.437320216	0.271106407	0.705438772	0.000698272

TREM2	0.024764966	0.874907239	0.778090835	0.983770328	0.02552065
TRDMT1	0.004011776	0.359011051	0.194244153	0.663540871	0.001080218
TRBV7-9	0.034092547	0.805624852	0.665122018	0.975808024	0.027076373
TRBV7-6	0.040946996	0.64671569	0.457389565	0.914409109	0.013652593
TRBV7-4	0.003532154	0.546496106	0.30252342	0.987222721	0.045221915
TRBV6-5	0.009346571	0.737695878	0.56927877	0.955937999	0.021405384
TRBV5-1	0.000877128	0.712536485	0.563381616	0.901179996	0.004680507
TRBV4-1	0.047409579	0.741278649	0.565027171	0.972509048	0.030680001
TRBV3-1	0.006169284	0.712926304	0.546358872	0.930274844	0.012691729
TRBV28	0.026770855	0.836484937	0.72411373	0.96629441	0.015274916
TRBV25-1	0.020793521	0.592732507	0.411349182	0.854096326	0.005013902
TRBV23-1	0.002606628	0.565625178	0.334868129	0.955396512	0.033124572
TRBV21-1	0.008207314	0.411083592	0.205934191	0.820600595	0.011716088
TRBV20-1	0.002753637	0.765466388	0.64385464	0.910048254	0.002463749
TRBV19	0.02280042	0.783709315	0.632522904	0.97103249	0.025828325
TRBV18	0.002013087	0.68213851	0.512909781	0.907202327	0.008553364
TRBV12-4	0.008640457	0.724588245	0.530480109	0.989722549	0.042877884
TRBV1	0.010586298	0.079359228	0.012094518	0.520722455	0.008295216
TRAV9-2	0.00249032	0.636071208	0.470091989	0.860654065	0.003361022
TRAV8-6	0.01049434	0.653468907	0.467507097	0.913401346	0.012770589
TRAV8-5	0.005644946	0.257364429	0.099499095	0.66569901	0.005123264
TRAV8-2	0.013180198	0.606413632	0.425127951	0.865004271	0.005775897
TRAV38-1	0.01834668	0.499680826	0.26509859	0.941841782	0.031934093
TRAV35	0.031761917	0.568540788	0.344314457	0.938789008	0.027326982
TRAV34	0.000433353	0.173300017	0.062920186	0.47731734	0.000697201
TRAV3	0.013726848	0.642524671	0.451233139	0.914910536	0.014161652
TRAV26-1	0.019224601	0.5661922	0.365466313	0.877163219	0.0108727
TRAV23DV6	0.001273189	0.591385264	0.397237922	0.880420801	0.009674929
TRAV22	0.024378765	0.5745678	0.356837172	0.925150693	0.022603075
TRAV21	0.000457313	0.648963643	0.492218686	0.855623368	0.002173643
TRAV15	0.002131904	0.000308081	9.43E-07	0.100704419	0.00619856
TRAV14DV4	0.027365451	0.595983369	0.423500232	0.838715424	0.002988275

TRAV12-2	0.010240456	0.770269186	0.595663354	0.996056943	0.046581365
TRAV12-1	0.004966268	0.675077149	0.515921261	0.883330835	0.004179669
TRAPPC2P8	4.48E-13	1.51E+24	8.91026E+11	2.56E+36	0.000106572
TRAK2	0.013209876	0.688367291	0.510812483	0.927638895	0.014149119
TRAJ54	0.020302382	0.071222284	0.006643567	0.76353771	0.029044885
TRAJ42	0.013017191	0.44047601	0.197292046	0.983410733	0.045415453
TRAJ28	0.017956396	0.467790369	0.237002573	0.923314149	0.028528038
TRAJ14	0.034283992	0.536919121	0.294006977	0.980528237	0.042974405
TRAF3IP3	0.001737377	0.644054759	0.493829543	0.839979175	0.001167106
TRAF3IP2-AS1	0.019413542	0.412548076	0.182315825	0.933522446	0.033580889
TRAC	0.014873165	0.87523316	0.767429622	0.998180241	0.046908739
TPX2	0.002890157	1.25449589	1.123257827	1.401067413	5.78E-05
TPSB2	0.010530289	0.866821378	0.781821168	0.961062878	0.006643901
TPSAB1	0.018235914	0.870627026	0.778642546	0.973478037	0.01502478
TPPP2	0.003915244	7.92E-06	3.70E-10	0.169450923	0.02094807
TPM3	0.00325283	1.554586277	1.126615606	2.145131383	0.007238984
TPI1	0.015972759	1.536454559	1.214783084	1.943303824	0.000339279
TP73-AS1	0.033595176	0.672083258	0.510731802	0.884409202	0.004555332
TP53TG5	0.038847731	0.258909137	0.091202505	0.735001097	0.011139021
TP53I3	0.001634722	1.322512195	1.111737491	1.57324775	0.001600601
TOX	0.002022836	0.825457049	0.699775079	0.973711925	0.022842119
TOP2A	0.036115354	1.168331155	1.045268179	1.305882753	0.006151619
TOMM40	0.013533322	1.392988664	1.13122831	1.715319003	0.001802242
TNS4	0.000102542	1.224208519	1.127616265	1.32907492	1.41E-06
TNS2	0.024843058	0.821792031	0.682563516	0.989420217	0.038240821
TNS1	0.0222543	0.816600497	0.704420053	0.946645924	0.007205714
TNRC6C	0.020936608	0.668867519	0.490262203	0.91253977	0.011167414
TNRC18P1	0.00154507	0.006586146	0.000204269	0.212354252	0.004592029
TNNT1	0.021597299	1.129715721	1.044814162	1.221516377	0.002215223
TNNC2	0.013070501	0.872849217	0.792840966	0.960931371	0.005564228
TNK2-AS1	0.025099788	0.269653956	0.085545111	0.849998972	0.025259609
TNIK	0.003536118	0.805211322	0.675776622	0.959437262	0.015389194

TNFSF8	0.002318008	0.691603359	0.520550889	0.91886349	0.010969221
TNFSF12	0.032770763	0.759646506	0.613120867	0.941189324	0.011925941
TNFRSF1A	0.021839562	1.433220434	1.090619769	1.88344359	0.00981329
TNFRSF19	6.60E-05	0.818650004	0.711739581	0.941619445	0.005071778
TNFRSF13C	0.002041807	0.789157764	0.648145406	0.960849171	0.018393101
TNFRSF13B	0.000788571	0.626542699	0.435786992	0.900797317	0.011604027
TNFRSF10C	0.01365252	0.788023245	0.653087297	0.950838636	0.012917796
TNFAIP8L2	0.005758624	0.822975745	0.700457447	0.966923945	0.017839109
TMX4	0.019194096	0.762639858	0.618609737	0.940204329	0.011170415
TMX2	0.009369247	1.455920976	1.043659059	2.031032903	0.026997684
TMX1	0.01060046	1.370713495	1.067208995	1.760531906	0.013536182
TMTC3	0.025414003	1.296852906	1.000371066	1.681203624	0.04967345
TMPRSS4-AS1	0.00338612	0.270745479	0.0884476	0.82877449	0.022080388
TMPRSS2	0.008022374	0.817441824	0.73645001	0.907340792	0.000152763
TMPRSS11E	0.029688764	1.143517059	1.05459177	1.239940706	0.001166915
TMPO	0.013264053	1.330594739	1.095635378	1.61594121	0.003960099
TMEM98	0.016541127	0.840541055	0.723842141	0.976054343	0.022740114
TMEM97P2	0.003434915	9654097.323	1507.502664	61825161122	0.000322569
TMEM81	0.044779386	1.528255277	1.103374388	2.116746788	0.010715384
TMEM64	0.046044134	1.208838283	1.008021878	1.449660991	0.040740285
TMEM63C	0.041002868	0.829572244	0.715988597	0.961174676	0.012880344
TMEM53	0.01409306	0.701452594	0.505170549	0.973999263	0.034237485
TMEM50B	0.000361604	0.615719081	0.484794779	0.782000969	7.01E-05
TMEM5	0.003397148	1.530529228	1.07187875	2.18543349	0.019185706
TMEM44	0.020758255	1.287361996	1.011840729	1.637906896	0.039805214
TMEM38B	0.048503053	1.24776176	1.009059961	1.542930519	0.041030987
TMEM35	0.031769311	0.75592476	0.584228057	0.978080794	0.03329073
TMEM252	0.003784097	0.375712292	0.164835816	0.856365619	0.019867506
TMEM25	0.00664003	0.662113482	0.524235688	0.836254137	0.000538166
TMEM243	0.018868394	0.73499172	0.596941682	0.904967512	0.003723068
TMEM241	0.010188304	0.633735701	0.470367079	0.853845766	0.002710833
TMEM239	0.004724277	45.85216776	1.069635042	1965.550123	0.046035019

TMEM194B	0.002568296	2.002883641	1.300185176	3.085362724	0.001628681
TMEM185B	0.03125918	1.359638326	1.039399354	1.778542935	0.024963571
TMEM163	0.007852557	0.81772951	0.723584756	0.924123325	0.001262292
TMEM158	0.02357504	1.167637455	1.00837169	1.352058213	0.038322508
TMEM130	0.000581659	0.817588775	0.713828825	0.93643095	0.003631873
TMEM125	2.01E-06	0.737108971	0.647380247	0.839274348	4.11E-06
TMEM110	0.001745017	0.228763982	0.097983389	0.534100322	0.000650307
TMEM11	0.013493277	1.693487	1.225167939	2.340820494	0.001425037
TMED7-TICAM2	0.002607589	4.572642251	1.40289734	14.90419616	0.011684395
TMA16	0.008569319	1.754359525	1.239240882	2.483598942	0.001527472
TM9SF3	0.004524385	1.433687035	1.073280658	1.915117448	0.014740136
TM6SF1	0.001952087	0.632317224	0.483090764	0.82763965	0.000845734
TLR8-AS1	0.018596614	0.769510996	0.595477013	0.994408112	0.045195482
TLR7	0.000361154	0.694464942	0.541629269	0.890427427	0.004039258
TLR4	0.041753727	0.834250401	0.698960989	0.995726146	0.044706315
TLR2	0.015964115	0.810264674	0.709776774	0.924979325	0.001843843
TLR10	0.00098623	0.681544066	0.533271634	0.871042605	0.002191497
TLE2	0.00145303	0.787367271	0.668779984	0.926982316	0.004100582
TLE1P1	0.042474393	1.59249725	1.12033218	2.263656742	0.009508267
TLE1	1.88E-05	1.813765636	1.434127223	2.293900937	6.73E-07
TLDC1	0.013081309	1.647991124	1.245903217	2.179844073	0.000464156
TK1	0.000278583	1.309411925	1.145019451	1.497406518	8.20E-05
TIMM10B	0.005393225	0.569956332	0.403588685	0.804904182	0.001411198
THYN1	0.004392055	0.618773793	0.464027199	0.825126218	0.001079203
THSD1	0.004069669	0.647182721	0.466234789	0.898357403	0.009306628
THRA	0.017695974	0.727488235	0.56759531	0.93242337	0.011987647
THEMIS2	0.031766845	0.824924128	0.69743532	0.975717457	0.024642997
THAP10	0.008628436	1.467438179	1.033817693	2.082934763	0.031867412
TGIF1	4.80E-05	1.592238359	1.237244662	2.049087841	0.000301447
TFF1	0.024613164	1.055758341	1.007696425	1.106112562	0.022460786
TFEC	0.044580033	0.769355198	0.59917137	0.987876675	0.039822247
TFEB	0.009860164	0.664306628	0.516552394	0.854324365	0.001439479

TFAP2A	0.000106161	1.277337504	1.116706565	1.461074154	0.000357296
TEX30	0.013292214	1.246906004	1.003721554	1.549009858	0.046204246
TESPA1	0.000484363	0.589321208	0.425778289	0.81568153	0.00143063
TESK2	0.009179266	0.569008918	0.395566315	0.818500302	0.002368644
TECPR1	0.006336142	0.731734592	0.572491041	0.935273175	0.012618118
TEAD4	0.019725154	1.459298644	1.186040499	1.795514179	0.000353108
TDG	0.039754396	1.356670401	1.060062357	1.736270103	0.015378056
TCTA	0.011087331	0.623434382	0.498118186	0.780277532	3.68E-05
TCP10L2	0.013067194	0.000658801	2.68E-06	0.161769766	0.009089123
TCP1	0.025084688	1.447545826	1.110715255	1.886522139	0.006200624
TCOF1	0.036189246	1.462474676	1.086907163	1.967814963	0.012062905
TCN1	0.001811616	1.112161517	1.046075532	1.182422494	0.000670996
TCERG1L-AS1	0.041319987	0.300452688	0.111919605	0.806577347	0.017005081
TCEB3-AS1	0.001906652	0.540105773	0.352732027	0.827013777	0.004601154
TCEB1P28	0.008742402	0.20121897	0.058857802	0.687913456	0.010575483
TCEB1P19	0.026705849	0.727175456	0.530498066	0.99676922	0.04769495
TCEANC	0.00132575	0.442909881	0.228702941	0.857746566	0.01573539
TCEAL4P1	0.014953451	8873923.65	1.704194377	4.62075E+13	0.042608917
TCEA3	0.020803158	0.860858671	0.762812246	0.97150728	0.015161046
TBX5-AS1	0.038102528	0.704861459	0.540163733	0.919776072	0.009999732
TBX4	0.007912315	0.777173099	0.65042288	0.928623583	0.005517353
TBRG4	0.008213706	1.399149612	1.077564644	1.816707374	0.011715504
TBCD	0.008688867	0.649938075	0.489145057	0.863587385	0.002965121
TBC1D31	0.002574062	1.483796049	1.097733304	2.005633524	0.010275211
TBC1D27	0.005346123	0.485351858	0.274228433	0.859015324	0.013076558
TBC1D24	0.045670879	0.769489091	0.602499703	0.982761416	0.035792442
TBC1D20	0.016127028	0.682529792	0.474233106	0.982316316	0.039781962
TBC1D10C	0.004510323	0.762723775	0.627363127	0.927290006	0.006582251
TAX1BP3	0.040776291	1.33395971	1.015420778	1.752424754	0.038462532
TAS1R2	0.017998773	7.65E-22	8.95E-40	0.000653253	0.020994483
TAS1R1	0.020515092	0.628807073	0.422410196	0.936053008	0.02228251
TAPT1	0.005824512	0.673804844	0.51944685	0.874031612	0.002937271

TANC1	0.014488945	0.677158191	0.500153119	0.916805669	0.011674409
TAF2	0.043069951	1.340939354	1.017324938	1.767496581	0.037355742
TAF1A	0.005042674	1.442664452	1.04297541	1.99552233	0.026816544
TACC3	0.000139728	1.367207698	1.153763353	1.6201389	0.000304446
TAC4	0.018221514	0.81311928	0.67325475	0.982039806	0.031701667
TAAR8	0.001786196	310318166	3356.920875	2.86862E+13	0.000803437
SYT16	0.018031838	1.863409077	1.137432733	3.052746141	0.013463878
SYT15	0.009556169	0.306320085	0.134606837	0.697081933	0.004801024
SYT11	0.006302072	0.795328548	0.642957375	0.983809384	0.03482454
SYS1	0.009695611	0.602577772	0.407115624	0.891884149	0.011345466
SYNPR-AS1	0.005533726	0.737382584	0.616420802	0.882080997	0.000860824
SYNGR4	0.017155143	1.283605711	1.010326554	1.630803044	0.040946241
SYNE3	0.010310084	0.60482113	0.383670534	0.953444601	0.030367939
SYNE1	0.045716589	0.717043718	0.574109582	0.895563686	0.003363569
SWT1	0.008843481	0.658817263	0.434538137	0.998854069	0.049372127
SUSD6	0.009060715	0.772239531	0.611025134	0.975989138	0.03051085
SUSD2	0.003641168	0.88224595	0.816723374	0.953025151	0.001462838
SUOX	0.020935784	0.627930674	0.463493756	0.850706026	0.002667551
SULT2B1	0.017188295	1.230147423	1.07664229	1.405538957	0.002320028
SULT1A1	0.00083821	0.733585471	0.592020106	0.90900231	0.004623899
STYX	0.037067493	1.459554929	1.062751377	2.004514542	0.019494421
STYK1	0.04558244	1.328194326	1.114805375	1.582428832	0.001492288
STXBP5	0.012090526	1.46657123	1.056410429	2.035980633	0.022147437
STXBP1	0.023049985	0.79530979	0.680537345	0.929438576	0.003974098
STX3	0.030582147	0.669366018	0.50194959	0.892621241	0.006267163
STX16-NPEPL1	0.009468824	0.587000744	0.371224061	0.928199193	0.022687047
STRIP2	2.45E-05	1.263513168	1.097195666	1.45504177	0.001161906
STRAP	0.009623053	1.481561896	1.182124991	1.856847346	0.000644008
STK33	6.33E-05	0.579860739	0.446968901	0.752263696	4.07E-05
STK32A	0.014082955	0.782511435	0.667344411	0.91755342	0.002533838
STK17B	0.00770028	0.691967881	0.556729422	0.860057918	0.000904309
STIL	0.018837859	1.276577546	1.02643908	1.587673601	0.028200414

STEAP1	4.88E-05	1.140063667	1.027349666	1.265143901	0.013588133
STC1	0.00248604	1.21021998	1.077763938	1.35895473	0.001254282
STAP1	0.000157028	0.605872287	0.469089274	0.782540228	0.000123926
ST6GALNAC6	0.00042975	0.643386514	0.507221687	0.816105101	0.000278121
ST6GALNAC4	0.001987699	0.790478084	0.66488089	0.939800813	0.007739078
ST6GAL1	0.024663282	0.813685796	0.685090584	0.966419024	0.018816631
ST3GAL6	0.00164056	0.628130215	0.47330396	0.833602928	0.001280211
ST3GAL5-AS1	0.019647593	0.597245115	0.433782925	0.822304676	0.001582707
ST3GAL4-AS1	0.014353301	1.566814971	1.267632335	1.936609762	3.27E-05
ST3GAL4	0.009671338	1.239821144	1.052814974	1.460044269	0.009968083
ST13P16	0.009843563	0.000247379	2.85E-07	0.214698817	0.016144384
SSU72P6	0.006237432	1.49651E+15	69.91719524	3.20E+28	0.025669834
SSTR4	0.00525094	6.87E-06	3.60E-10	0.131348043	0.018100551
SSRP1	0.047385806	1.452477447	1.078332222	1.956438556	0.014040782
SSBP3	0.040545935	0.771607526	0.60026937	0.991851666	0.042987948
SRSF9	0.018429224	1.443563468	1.065309557	1.956122023	0.017881911
SRGAP2-AS1	0.004037589	2.44E-10	1.80E-17	0.003319675	0.008263599
SRGAP1	0.003624542	2.189956	1.53143054	3.131651849	1.74E-05
SPRR1B	0.001112303	1.08607953	1.02026258	1.156142318	0.009628593
SPOP	0.047986884	0.63131379	0.419903594	0.949163349	0.027054022
SPOCK1	0.00071204	1.223805631	1.089638809	1.374492365	0.000652121
SPNS3	0.013660155	0.58336845	0.380715765	0.893891926	0.013319236
SPN	0.020807308	0.78024576	0.652622982	0.932825631	0.006468699
SPIN4	0.008780089	1.378897177	1.104027448	1.722201226	0.004619471
SPIN2B	0.038977421	0.548392636	0.342960002	0.876879174	0.012121471
SPHK1	0.034273147	1.324008324	1.132246235	1.548248066	0.000438349
SPECC1	0.000252704	1.576677199	1.214535664	2.046799499	0.00062679
SPDL1	0.011396644	1.61683881	1.250198239	2.091002576	0.000250443
SPC25	0.001912394	1.393815562	1.173296875	1.655780272	0.000157647
SPC24	0.005707306	1.242822422	1.063434868	1.45247031	0.006270614
SPATA7	0.007587421	0.563673977	0.344978858	0.921008186	0.022112041
SPATA6L	0.018790379	0.572966344	0.380465785	0.86286453	0.007675016

SPATA6	0.000245209	0.576226472	0.434551443	0.764091231	0.000128763
SPATA5	0.022487043	1.643605568	1.171901278	2.30517648	0.003988393
SPATA31D3	0.004536927	2.17E+72	2752319490	1.72E+135	0.024188855
SPATA18	0.048049516	0.791837009	0.674967558	0.928942201	0.004175005
SPATA13	0.006648395	0.686905228	0.526653794	0.895918339	0.005591175
SPATA1	0.029515513	0.193068232	0.053770958	0.69322443	0.011677346
SPARCL1	0.011959095	0.861935012	0.756984429	0.981436257	0.024907932
SPAG5	0.000506677	1.250162067	1.08510707	1.440323482	0.001997746
SP6	0.000265247	1.187675769	1.026549543	1.374092213	0.020765492
SOWAHC	0.011481109	1.593664115	1.277525035	1.988035649	3.61E-05
SORD	0.025280403	1.295308921	1.027841366	1.632377579	0.028331143
SORCS2	0.035806872	0.839007709	0.73120294	0.962706655	0.012363137
SOCS5P1	0.014525865	1.17E+26	92.77837999	1.49E+50	0.034010055
SNX9	0.022347366	1.350749055	1.040631929	1.75328371	0.023868164
SNX7	0.038976131	1.308203353	1.064726272	1.607357738	0.010561941
SNX30	0.002457744	0.7205685	0.608945995	0.852651906	0.000135519
SNX29P2	0.019858925	0.011151652	0.000394825	0.314973442	0.008346796
SNX22	0.002540611	0.633926127	0.451380354	0.890296466	0.008524425
SNX20	0.036315155	0.733611029	0.588062137	0.915184141	0.006042445
SNX18P7	0.037120389	1.651769576	1.123968672	2.427418841	0.010620747
SNW1	0.002114636	1.761406758	1.183451447	2.621614747	0.005269167
SNTG2	0.002744507	0.063870378	0.007726975	0.527945972	0.010689025
SNRPGP3	0.044657117	2.012390736	1.061394716	3.815466961	0.032152439
SNRPGP14	0.003403028	0.588347538	0.391048428	0.885191708	0.010924681
SNRPEP2	0.029436356	1.347283832	1.004337407	1.807334579	0.046718834
SNRPD2	0.034226484	1.230659697	1.001168843	1.512755116	0.048717272
SNRPCP17	0.015671952	0.002890423	9.11E-06	0.916940842	0.046649178
SNRPCP10	0.04775917	0.037975589	0.002113487	0.682353547	0.026466474
SNRPA	0.02048737	1.452446351	1.053875033	2.001755745	0.022573093
SNORD115-46	0.000781506	57609.36517	14.08563256	235618737	0.009784078
SNORD115-20	0.000151279	415.0113548	16.90467691	10188.56648	0.000222984
SNORD115-2	0.00365951	509.2414076	2.474457292	104801.49	0.021829435

SNORD115-11	5.50E-10	9881.257336	68.130463	1433121.723	0.000291895
SNORD114-13	0.000592796	4.109735465	1.525358599	11.07275732	0.005191168
SNORD113-6	0.000292731	299.968149	4.742060831	18975.06034	0.007027237
SNORD112	0.04271067	0.519825654	0.278225274	0.971222728	0.040216075
SNORA67	0.023134023	0.555490046	0.344698653	0.895185369	0.015745912
SNORA55	0.034654178	0.793526266	0.64395222	0.977842635	0.029989651
SNN	0.000680528	0.621730392	0.483083667	0.800169217	0.000222785
SNHG7	0.029633893	0.791192258	0.649751148	0.963422982	0.019764744
SNHG3	0.017168726	0.798890086	0.668688216	0.95444387	0.013374359
SNHG19	0.042523644	0.840150815	0.723770262	0.975245087	0.022052996
SNHG12	0.006086101	0.780873638	0.653913279	0.932483952	0.006293065
SNAI3	3.45E-05	0.523362667	0.376407366	0.727691608	0.000118018
SNAI1	0.031019291	1.213985484	1.030266168	1.43046603	0.020552245
SMS	0.002238013	1.582207473	1.269493177	1.971952691	4.43E-05
SMOX	0.004891698	1.342711854	1.155618966	1.560094786	0.000118531
SMNDC1	0.016065249	1.777805408	1.183554643	2.670423447	0.005574887
SMIM4	0.031939643	0.720636273	0.545540721	0.95193011	0.02106382
SMIM17	0.005354305	0.263004652	0.103681826	0.667151128	0.004920822
SMIM15	0.001851951	1.564855007	1.144346129	2.139886816	0.005041326
SMG7	0.026961455	1.402656512	1.012490876	1.943173355	0.041890492
SMG1P5	0.041653319	0.180118322	0.038299878	0.847068229	0.030000375
SMCR5	0.016278695	0.012769388	0.000176662	0.922991884	0.045862599
SMCO2	0.002769635	2.8492006	1.601349124	5.069440473	0.00036862
SMC2	0.010250087	1.424727115	1.149049014	1.766545489	0.001254286
SMARCD3	0.014217469	0.743860312	0.613496207	0.901925974	0.002612792
SMARCAL1	0.002069892	2.094651609	1.274244203	3.443268845	0.003549596
SMAGP	0.046382456	1.228567088	1.009845639	1.494661196	0.039598625
SMAD9	0.002726069	0.684471749	0.536410755	0.873400786	0.002300667
SLMO2P1	0.008143943	6.41E-05	5.72E-09	0.718500799	0.042412074
SLIT3	0.001522656	0.78803757	0.65841627	0.943177196	0.009376906
SLFN12	0.037630039	0.749125506	0.576979642	0.972632279	0.030138495
SLCO1B7	0.005838261	11.7848204	2.931762227	47.37150601	0.000510288

SLC7A8	0.002252924	0.8317752	0.723496952	0.956258326	0.009638622
SLC6A8	0.024162805	1.173805677	1.031842508	1.335300451	0.014827249
SLC6A18	0.035050805	0.096140815	0.014060488	0.657378038	0.016956623
SLC6A17	0.030285721	1.856307234	1.362970728	2.528210237	8.69E-05
SLC52A3	0.029654341	1.255842681	1.043396455	1.511545139	0.015985946
SLC52A1	0.035634317	0.742674999	0.571860087	0.964512415	0.02568634
SLC4A8	0.017801777	0.546033354	0.383111673	0.778238943	0.000817708
SLC4A5	0.000490342	0.530426193	0.332561884	0.84601381	0.007768012
SLC47A1	0.000523268	0.68548787	0.5714764	0.822245012	4.73E-05
SLC46A3	0.002543816	0.703549515	0.582710725	0.849447073	0.00025516
SLC44A4	0.005524592	0.903483955	0.823524844	0.991206596	0.031811022
SLC44A1	3.61E-05	1.463522646	1.14594525	1.869110705	0.002277075
SLC43A1	0.005909948	0.79470473	0.648979346	0.973152093	0.026196807
SLC3A2	0.029349342	1.344914499	1.067044187	1.695145365	0.01208975
SLC35F2	0.007840007	1.233755841	1.020788523	1.491154574	0.029796475
SLC35E2B	0.026967791	0.74788506	0.596279991	0.938035941	0.011957633
SLC34A2	0.007399669	0.881398002	0.823566105	0.94329093	0.000266352
SLC2A7	0.016722581	4.536053011	1.268947893	16.21483201	0.019995187
SLC2A11	0.006477049	0.598297391	0.409431789	0.874284259	0.007950623
SLC2A1-AS1	0.017255666	1.56229802	1.000287307	2.440074053	0.049852578
SLC2A1	4.18E-05	1.260694284	1.134365867	1.401091237	1.71E-05
SLC27A1	0.020576209	0.705548665	0.549172707	0.9064524	0.006367331
SLC26A5	0.033705602	0.555171671	0.319137078	0.965778049	0.037230048
SLC26A11	0.005418751	0.704467226	0.539220733	0.920354212	0.010214127
SLC25A6P5	0.038928355	8.30E-05	2.54E-08	0.271103834	0.022837975
SLC25A5-AS1	0.00216647	0.488913354	0.267345908	0.894108573	0.020158585
SLC25A47P1	0.009806074	0.507217461	0.320200697	0.803463437	0.003823812
SLC25A47	0.009890396	4.030671139	1.124282587	14.4503793	0.032371705
SLC25A42	4.18E-05	0.522023114	0.394113165	0.691446407	5.82E-06
SLC25A34	0.036016917	0.445306837	0.247564161	0.800997118	0.006918293
SLC25A33	0.038929969	0.71585254	0.5207766	0.984001314	0.039463602
SLC25A16	0.018367532	0.684985932	0.517905131	0.905968485	0.007997387

SLC25A14	0.014749908	0.668592072	0.466079204	0.959097413	0.028755788
SLC24A4	0.002097297	0.073944283	0.011482409	0.476185526	0.00613011
SLC22A31	0.028722535	0.904600983	0.839884635	0.974303974	0.008113373
SLC22A23	0.001665846	0.801163412	0.68439443	0.93785511	0.005811569
SLC22A17	0.033232019	0.836828005	0.70926402	0.987334885	0.034771404
SLC22A15	0.018728689	0.751497292	0.568841967	0.992803298	0.044346499
SLC22A10	0.003036752	5.34E-05	5.23E-08	0.054535547	0.005387945
SLC18A2	0.002956324	0.521715876	0.354408158	0.768005614	0.000974075
SLC16A3	0.000238722	1.361666929	1.163643214	1.593389453	0.000118064
SLC16A11	0.01450063	0.719450022	0.578318395	0.89502312	0.003122391
SLC15A2	0.00265694	0.783666021	0.67372708	0.911544823	0.001573174
SLC14A1	0.006336396	0.586291683	0.373149946	0.921179117	0.020552916
SLC13A5	0.01458866	1.358636661	1.005396477	1.835985722	0.046043196
SLC11A2	0.002944071	0.671351491	0.506796955	0.889336094	0.005478606
SLBP	0.00555034	1.315699273	1.012343453	1.709957793	0.04019861
SLAMF1	0.000487719	0.629991215	0.479697178	0.827373914	0.00089156
SKIL	0.04842009	1.300860965	1.019627237	1.659664618	0.034314325
SKA3	0.001897112	1.396328009	1.172572265	1.662781873	0.00017926
SKA1	0.037868553	1.310862579	1.112472032	1.544632722	0.001225086
Six3os1_5	0.002541855	2.290810947	1.353829563	3.87627434	0.002009488
Six3os1_2	0.017257943	1.505459378	1.093159591	2.073263554	0.01222863
SIT1	0.0122575	0.803413282	0.676813031	0.953694555	0.012353402
SIRT4	0.035147917	0.593708813	0.402728179	0.87525575	0.008468547
SIRT2	0.004689274	0.632634483	0.450990347	0.887438925	0.00801337
SIGLEC6	0.005150969	0.555289558	0.358858431	0.859242716	0.008265066
SIGLEC18P	0.010504373	0.1980972	0.045352966	0.865268663	0.031369513
SIGLEC17P	0.035108393	0.519691333	0.349303014	0.77319425	0.001242571
SIGLEC11	0.046162228	0.574334728	0.330047581	0.999432806	0.049765828
SIDT2	0.017210576	0.699705985	0.529288859	0.924992956	0.012160675
SIAH2-AS1	0.001699471	0.344657244	0.150959465	0.786890813	0.011439796
SIAH2	0.017442477	1.351957704	1.01323533	1.803914233	0.04043088
SHISA4	0.017639527	0.779687876	0.638602715	0.951942687	0.014543565

SHISA2	0.000295884	0.819458479	0.728685111	0.921539618	0.00088717
SHE	0.049547228	0.866168953	0.752333333	0.99722905	0.045655749
SHCBP1	0.000994456	1.45928806	1.210297983	1.759501934	7.51E-05
SHC1	0.003677559	1.626115551	1.296593548	2.039383728	2.58E-05
SH3TC2	0.039257345	3.187706116	1.54284487	6.586190535	0.00174126
SH3PXD2A-AS1	0.019422279	1.390425753	1.125946636	1.717029666	0.002199156
SH3GL1	0.043685798	1.584839338	1.168764748	2.149034466	0.00304036
SH3BP5-AS1	0.024381282	0.707546778	0.557128316	0.898576554	0.004554773
SH3BP5	0.033303264	0.712647286	0.572367037	0.887308529	0.002453685
SH2D5	0.004887123	1.850477031	1.333930816	2.567048608	0.000228436
SH2D3A	0.000912535	1.300399387	1.038329514	1.628614561	0.022165151
SH2B1	0.034716535	0.721226141	0.573893292	0.906383039	0.005062297
SGOL2	0.000419027	1.522707975	1.214902772	1.908498054	0.000262783
SGOL1	0.001673449	1.468132389	1.183132284	1.821785052	0.000488264
SGK3	0.000723066	0.606416619	0.406053363	0.905647261	0.014514936
SGCG	0.038495356	0.408417513	0.214049457	0.77928189	0.006597754
SFXN2	0.028992505	0.691120781	0.510161156	0.936268723	0.017074044
SFXN1	0.001608609	1.53735102	1.173279317	2.014395145	0.001815377
SFTPD	0.006936968	0.903031336	0.850333872	0.958994602	0.000884913
SFTPC	0.027058298	0.941646911	0.903875969	0.980996216	0.003995106
SFTPB	0.000420472	0.909053377	0.868133865	0.95190163	4.96E-05
SFTA3	0.000375116	0.831294149	0.767114284	0.900843559	6.57E-06
SETP22	0.002956512	7.717466976	1.30909213	45.49664241	0.023976481
SETP17	0.000429722	0.045773133	0.007417788	0.282453431	0.000895134
SETDB2	0.00622053	0.418656837	0.28319319	0.61891865	1.27E-05
SESN3	0.01063183	0.80675798	0.685687035	0.949206278	0.009644701
SERPINH1	0.040692791	1.322745302	1.072213044	1.631816684	0.00903401
SERPINE1	0.02050309	1.152224221	1.037188174	1.280019084	0.008281732
SERPINB7	0.017114402	1.319618469	1.131600798	1.538875641	0.000405583
SERPINB5	0.000204902	1.183861083	1.087206183	1.289108806	0.000102727
SERPINA15P	1.29E-14	5.21269E+16	294199590.1	9.24E+24	7.12E-05
SERF1B	0.004461899	0.193412334	0.051986763	0.71957416	0.014249753

SERBP1P4	0.005232317	14589.02112	9.320945577	22834543.51	0.010626186
SEPW1	0.011045626	0.748005176	0.620638542	0.901509825	0.002298627
4-Sep	0.039191476	0.620096565	0.430793289	0.892585283	0.010128556
2-Sep	0.014676854	1.529432127	1.072468621	2.181101233	0.018960606
1-Sep	0.004414138	0.69683584	0.564402117	0.860344377	0.000783193
SEPP1	0.003520185	0.808846912	0.711149774	0.919965599	0.001237539
SENP8	0.001828494	0.454651799	0.253244177	0.816240914	0.00828975
SENP7	0.026943713	0.586997855	0.419670103	0.821041289	0.001860101
SEMA4B	0.015295535	1.28689839	1.098151071	1.508087102	0.001827278
SEMA4A	0.008111685	0.743375501	0.610544882	0.905104852	0.003150203
SEMA3C	0.018469525	1.228476497	1.075508918	1.403200362	0.002422543
SEMA3A	0.025443266	1.169107928	1.03266957	1.323572793	0.013598233
SELP	0.006373339	0.791596917	0.657289781	0.953347667	0.013755197
SELK	0.001977806	0.586609152	0.429183784	0.801778421	0.000820796
SELENBP1	0.024496317	0.836016789	0.753471769	0.927604856	0.000733344
SEC61G	0.003598183	1.491153234	1.21649894	1.827817431	0.000119672
SEC23A	0.032817908	1.225670454	1.006970915	1.491868374	0.042433983
SEC14L6	0.011141842	0.772204263	0.650728491	0.916356717	0.003074103
SEC14L4	0.013974454	0.78067944	0.650170867	0.937384952	0.007983539
SEC11C	0.001353537	0.848683004	0.739235351	0.974334953	0.019856473
SDK1	0.010221875	0.806134804	0.697401657	0.931820732	0.003554694
SDHAP3	0.046139119	0.85759171	0.756989314	0.971563967	0.015817647
SDCBP2	0.010577382	1.184959597	1.070598144	1.311537156	0.001047835
SCPEP1	0.000615373	0.772507496	0.657904376	0.907073814	0.001630631
SCNN1B	0.000434671	0.840445752	0.761426061	0.927665993	0.000559833
SCN7A	0.030337012	0.743884952	0.618459856	0.894746549	0.001687029
SCN2B	0.00292141	0.477820625	0.253055718	0.902222449	0.02277189
SCN1A	0.02399614	0.56794418	0.363125671	0.888289145	0.013173317
SCML4	0.001087933	0.424179032	0.23156799	0.776997937	0.005486426
SCML2	0.013949833	1.290534177	1.015458681	1.640124302	0.037033892
SCMH1	0.007617398	0.686091476	0.502679406	0.936424903	0.017603794
SCIMP	0.001405492	0.776158321	0.629392635	0.957147742	0.017811626

SCGB3A1	0.023046646	0.925694981	0.882404786	0.971108963	0.001579379
SCARF1	0.032191459	0.730002302	0.586982259	0.907869619	0.004673161
SCAI	0.026908213	0.54299006	0.342723706	0.860279578	0.009296003
SBK3	0.020126129	1.587117111	1.130344931	2.228470844	0.007641443
SBK1	0.025796224	0.879287552	0.773999877	0.998897573	0.048051264
SATB2-AS1	0.000226043	9.331391268	2.595910058	33.54309704	0.00062326
SATB2	0.002343467	1.655017806	1.204426773	2.274180549	0.001889493
SATB1	0.0131421	0.766827787	0.605223474	0.971583026	0.027898482
SAT2	0.001657248	0.718579533	0.558373355	0.924751405	0.010234283
SASH3	0.003178058	0.803210553	0.692838209	0.931165725	0.003665778
SARM1	0.008540348	0.471178405	0.28408744	0.781481538	0.003555735
SARAF	0.001987075	0.740811175	0.578154037	0.94923007	0.017696699
SAPCD2	0.001692338	1.249134406	1.076237231	1.449807457	0.00342756
SAMM50	0.006515159	1.721782489	1.169272658	2.535366681	0.005923148
SAMD4B	0.008747368	1.315933574	1.007666004	1.71850709	0.043795571
SAMD3	0.049599237	0.390862464	0.176080829	0.867632588	0.020946116
SAMD13	0.002894472	1.428150208	1.093232711	1.865671413	0.008956334
SALRNA1	0.041228121	0.41982334	0.209765519	0.840231688	0.014218433
SAE1	0.03575434	1.519887556	1.125487234	2.052496122	0.006310148
SAAL1	0.016917875	1.457617723	1.056419716	2.011179264	0.021783081
S100P	0.038375595	1.069965238	1.016166739	1.12661197	0.010191582
S100A16	1.04E-05	1.260782145	1.100878049	1.443912537	0.000811437
S100A10	0.000707415	1.27585334	1.08432146	1.501216941	0.003331015
RXRB	0.00823646	0.616240577	0.433873159	0.875261447	0.006847507
RXFP1	0.003921	0.395707797	0.195712404	0.800075304	0.009853851
RUVBL2	0.003507675	1.441756286	1.080054659	1.92458888	0.013045729
RUFY3	0.041880225	0.641512101	0.477279205	0.862257921	0.003259065
RTN1	0.005672089	0.692420338	0.529796124	0.904963065	0.007121807
RTCA-AS1	0.015254474	0.630470684	0.435066749	0.91363747	0.014802832
RSRC1	0.005752897	1.619039111	1.188932841	2.204739875	0.002224927
RSPO1	0.002585987	0.495663014	0.263717293	0.931610592	0.029257725
RSPH9	0.003502943	0.724345039	0.541859042	0.968288236	0.029438574

RRN3P2	0.041454778	0.53536372	0.308913071	0.927815427	0.025946565
RRM2	0.000569177	1.322748251	1.164307198	1.502750252	1.73E-05
RRM1	0.011379293	1.388047437	1.083412206	1.778340393	0.009495509
RPSAP35	0.030921983	3.01E+31	137.4496163	6.58E+60	0.035483662
RPS6KL1	0.001393071	0.574153867	0.387080659	0.851638167	0.005810189
RPS6KA5	0.023535793	0.492294663	0.242419921	0.999728216	0.049912157
RPS6KA4	0.01799947	1.464382599	1.10682382	1.937450529	0.007572335
RPS6KA1	0.004880836	0.590566723	0.443361394	0.786647325	0.000317583
RPS4XP12	0.022251219	0.016461128	0.000397779	0.681203859	0.03061267
RPS3AP34	0.037824709	0.4227623	0.209936615	0.8513425	0.015926873
RPS29P25	7.71E-06	55689339706	550579.8241	5.63279E+15	2.58E-05
RPS17P13	0.001296182	9.73E-12	4.28E-21	0.022141505	0.021078038
RPP25	0.002004059	1.313728484	1.09008624	1.583253202	0.004158164
RPLP2P1	0.016718296	9.605246177	1.102141241	83.71046347	0.040559399
RPL7P53	0.002103009	1.51E-08	1.29E-14	0.017589851	0.011508408
RPL7P35	0.018385313	3.90E-07	7.56E-13	0.201610584	0.027908843
RPL7AP64	0.009201905	0.613913261	0.402132155	0.937227943	0.02380293
RPL6P21	0.012456452	7738.856735	10.81468864	5537829.663	0.007587366
RPL4P6	0.016444596	2.032024533	1.192268438	3.463250032	0.009149382
RPL38P1	0.000605568	281.4117428	12.70019623	6235.539002	0.000359956
RPL37P21	0.004883058	6339111293	23.17979438	1.73359E+18	0.022780858
RPL35P4	0.019486149	3.087173603	1.121431763	8.498636444	0.029125617
RPL34P26	0.043276415	0.11809051	0.018211536	0.765743659	0.025103724
RPL34P22	0.008088489	0.156470513	0.02976156	0.822639044	0.028485817
RPL32P1	0.004837498	0.557286734	0.391742779	0.7927868	0.001149445
RPL31P40	0.001279851	0.324053212	0.126621446	0.829326211	0.018758824
RPL31P13	0.006531691	327885.9031	4.89703668	21953922844	0.025079494
RPL26P3	0.044250036	0.012318461	0.000284589	0.53320491	0.022190962
RPL23AP81	0.029742458	0.301581615	0.116211456	0.782637737	0.013751877
RPL23AP64	0.037038588	0.796211608	0.645288949	0.982432639	0.033565842
RPL21P126	0.014222976	1837.601055	4.601903751	733778.4145	0.013914801
RPL18AP7	0.019085071	0.301044247	0.115966683	0.781497201	0.01364389

RPL17P50	0.02455404	1.300632284	1.001778428	1.688641211	0.048461011
RPL13AP17	0.005854551	0.652963566	0.484870948	0.879329685	0.005004086
RPIA	0.03812271	0.705370805	0.524731977	0.948194494	0.020755974
RPE	0.000753031	1.688217013	1.241091338	2.296427826	0.000850394
RPARP-AS1	0.0065741	0.603460897	0.453314138	0.803339283	0.000539929
RP6-159A1.4	3.31E-05	0.596165748	0.446005431	0.796881773	0.000476766
RP5-997D24.3	0.01736745	0.000650805	4.46E-07	0.950150589	0.048413687
RP5-994D16.9	0.001199596	0.559876111	0.367551201	0.852836988	0.006906551
RP5-991C6.2	0.04592242	0.5377413	0.329542089	0.877477309	0.013023732
RP5-986I17.2	3.28E-05	6.2536E+11	82115.53789	4.7625E+18	0.000780466
RP5-935K16.2	0.003993778	0.017701934	0.00068402	0.458113196	0.015088721
RP5-907D15.4	0.014901097	1.425179694	1.063809958	1.90930452	0.017571167
RP5-905H7.9	0	1.93E+32	3.9127E+17	9.53E+46	1.66E-05
RP5-905G11.3	0.03953893	0.062677785	0.004471551	0.878555284	0.039774924
RP5-903G2.2	0.014579112	0.699120642	0.492865999	0.991688762	0.04477566
RP5-902P8.10	0.044220517	0.731040775	0.551028131	0.969860856	0.029844707
RP5-901A4.4	0.003968692	0.08237203	0.009248556	0.733644411	0.025249158
RP5-892G5.2	0.009885148	0.047870629	0.005241237	0.43722445	0.00708065
RP5-887A10.1	0.02545947	0.694816404	0.533077876	0.90562722	0.00707751
RP5-837O21.1	0.020476554	1.73E-06	8.55E-12	0.348310723	0.033239029
RP5-837M10.4	0.037876301	0.01031543	0.000251135	0.423708513	0.015823835
RP5-831C21.1	0.001753868	0.746434941	0.575869341	0.967520026	0.02714529
RP5-827L5.1	9.22E-06	9.93E+32	5.67458E+11	1.74E+54	0.002331221
RP5-1189B24.1	0.012219708	0.031356659	0.001566313	0.627741741	0.02354323
RP5-1173I20.1	0.047642276	0.023275822	0.0009233	0.58676913	0.022386665
RP5-1171I10.5	0.012872094	0.72400131	0.58961884	0.889011444	0.002049184
RP5-1132H15.3	0.031840651	0.700319593	0.565277053	0.867623284	0.00111751
RP5-1114G22.1	3.82E-05	47319569.96	328.4832345	6.81661E+12	0.003544322
RP5-1070G24.2	0.002564731	0.262158173	0.095320781	0.721006551	0.009495825
RP5-1068H6.1	0.000456086	5.29685E+12	230880.482	1.22E+20	0.000703763
RP5-1059L7.1	0.010167461	1.364183842	1.124998231	1.654222649	0.001591492
RP5-1057J7.7	0.019302504	0.765775534	0.608030362	0.964445534	0.023355232

RP5-1050D4.5	0.045623139	0.585137917	0.368182895	0.9299356	0.023372854
RP5-1049N15.2	0.039534487	0.028684781	0.000952411	0.863930552	0.040938556
RP5-1028L10.2	0.03067542	4.517280612	1.821406456	11.20333358	0.001138639
RP5-1028K7.1	0.027502466	1.05E-29	6.86E-58	0.160442636	0.043880946
RP5-1018K9.1	0.003655355	0.142070248	0.027532665	0.733091218	0.019763365
RP5-1009E24.9	0.007626345	0.334026431	0.173908394	0.641565678	0.00099202
RP5-1000K24.2	0.026922059	0.249531741	0.088024345	0.707373504	0.009023462
RP4-815D20.1	0.016476726	4.03E+38	48293.18811	3.36E+72	0.025707844
RP4-803A2.2	0.00116905	0.590183473	0.379807699	0.917086549	0.019035198
RP4-803A2.1	0.028281312	0.651232613	0.442888323	0.957586585	0.029236623
RP4-800J21.3	0.00079532	0.302753131	0.117946806	0.777125396	0.012983397
RP4-785G19.5	0.032284058	1.810227961	1.221071506	2.683647318	0.003134638
RP4-758J18.7	0.045468904	0.710092375	0.513390065	0.982159989	0.038570799
RP4-756G23.5	8.92E-05	0.303680042	0.146863626	0.627940153	0.001302911
RP4-753P9.3	0.022821304	0.57204167	0.375900289	0.870527854	0.009129031
RP4-742C19.13	0.043378394	0.496450455	0.259584833	0.949450904	0.03428027
RP4-736H5.3	0.013435938	0.035500003	0.001484783	0.848777489	0.039283894
RP4-724E13.2	0.043187955	2.183932777	1.194312069	3.993564576	0.01119354
RP4-715N11.2	0.027722848	27.53321697	5.269817784	143.8527987	8.49E-05
RP4-694A7.2	0.017658811	1.806634065	1.347649636	2.421940062	7.65E-05
RP4-682C21.2	0.028684325	0.476831368	0.237761984	0.956284722	0.036991379
RP4-671O14.5	0.002115864	0.038800867	0.001595726	0.943462403	0.045965571
RP4-639F20.1	0.048480548	0.71085992	0.580554767	0.870411982	0.000955424
RP4-635E18.7	0.02998855	0.19040553	0.046370613	0.781837108	0.021365455
RP4-631H13.6	0.007898822	0.824941358	0.696394912	0.97721599	0.025971845
RP4-620E11.4	0.0382017	0.148629778	0.033882626	0.651980481	0.011504999
RP4-614C15.2	0.047917619	40.77671178	1.164892631	1427.376378	0.040943584
RP4-605O3.4	0.009835471	0.54723379	0.333441979	0.898101739	0.017072078
RP4-583P15.16	0.01186747	0.577759817	0.35424566	0.942302036	0.027943277
RP4-569M23.2	0.003668864	0.61975208	0.430298221	0.892619634	0.010163785
RP4-562D20.2	0	61946478592	940036.342	4.08215E+15	1.14E-05
RP4-545K15.5	0.004003523	0.477040729	0.272726042	0.834419239	0.00947283

RP4-543J13.2	0.00680471	74971509.06	123.3208096	4.55781E+13	0.007617968
RP3-525N10.2	0.001201952	0.12988875	0.031724815	0.53179466	0.004539196
RP3-510D11.2	0.027412446	0.617661738	0.435144409	0.876734286	0.007015984
RP3-510D11.1	0.001290931	0.372209556	0.195667536	0.708037503	0.002592769
RP3-509I19.1	0.017011931	0.013839994	0.000317728	0.602859166	0.026230946
RP3-508I15.10	0.006552731	0.652796009	0.434139474	0.981580009	0.040433003
RP3-496C20.1	0.000279596	0.013518348	0.00048344	0.378010901	0.011328436
RP3-492J12.2	0.000451276	0.07242993	0.012378686	0.423800629	0.003586638
RP3-477O4.14	0.00441841	0.417524393	0.225157134	0.774244261	0.005570653
RP3-476K8.4	0.010145066	0.332693378	0.144382979	0.766606178	0.009765908
RP3-475N16.1	0.002188811	0.515611375	0.333796369	0.796458903	0.002828549
RP3-467N11.2	0.027475606	5.643777419	1.44313446	22.07155635	0.01287694
RP3-467L1.4	0.035658939	0.565683774	0.346397635	0.923788443	0.022800719
RP3-449M8.9	3.06E-05	0.735949869	0.542229796	0.998879465	0.049165048
RP3-449M8.6	0.001232067	0.675557516	0.490042006	0.931303749	0.016645352
RP3-428L16.1	0.048568671	1.642783263	1.22182276	2.208779324	0.001015038
RP3-415N12.1	0.040219662	0.090641801	0.011958647	0.687028879	0.020168175
RP3-406A7.1	0.049094154	39.87895921	1.154319898	1377.72154	0.041413279
RP3-404K8.2	0.04775542	24.74025297	2.608028356	234.6907446	0.005189146
RP3-391O22.1	0.038783202	0.158304925	0.02934327	0.854044189	0.032078419
RP3-388N13.5	0.023997136	0.311658676	0.103824625	0.935530761	0.037636635
RP3-380C13.2	5.50E-10	3.15548E+18	308877369.4	3.22E+28	0.000291895
RP3-378P9.1	0.00419565	4265.945466	14.66045717	1241318.092	0.003881758
RP3-366N23.4	0.010386394	0.256898605	0.0682567	0.966892522	0.044457554
RP3-364H10.1	8.23E-05	9541030.94	785.5907288	1.15876E+11	0.000810248
RP3-348I23.3	0.034221169	0.013420342	0.000301672	0.597025117	0.025991507
RP3-340B19.3	0.005472829	0.749777981	0.598779191	0.938855307	0.012078081
RP3-339A18.3	0.012863861	0.000409163	2.36E-07	0.710213752	0.040376451
RP3-329A5.1	0.004635677	0.02832474	0.003610734	0.222196065	0.000695783
RP13-483F6.2	0.042582236	2778.39408	7.179902427	1075150.219	0.009096457
RP13-463N16.6	0.000786458	1.241934745	1.017576345	1.515760383	0.033059637
RP13-346H10.2	0.041874686	0.007860425	0.000129718	0.476313241	0.020659461

RP13-329D4.3	0.029034659	6.667651414	1.209245611	36.76471925	0.029399926
RP13-228J13.10	0.015261865	0.102959097	0.016954338	0.625242664	0.013502466
RP13-225O21.2	0.041309272	0.629902115	0.398408718	0.995903595	0.04798211
RP13-216E22.4	0.006164489	0.580300643	0.404955275	0.83157044	0.003029214
RP13-131K19.2	0.01925312	0.360692447	0.171862148	0.756996483	0.007017775
RP11-9M16.2	0.046953658	0.303698713	0.11551843	0.798425915	0.015673952
RP11-998D10.4	0.042906687	2.213646316	1.600514079	3.061660047	1.57E-06
RP11-98I9.4	0.00645326	0.593163443	0.404354133	0.870135463	0.007551432
RP11-98H4.4	9.54E-05	498566.5226	73.12747216	3399113495	0.003579951
RP11-98D18.9	0.045048314	0.659582557	0.474306812	0.917231501	0.013380597
RP11-98D18.15	0.025185624	0.500979359	0.265282804	0.94608589	0.033103961
RP11-989E6.3	0.016861038	20654105323	683.0830906	6.2451E+17	0.006879525
RP11-977G19.11	0.001642418	0.547315005	0.360788449	0.830275238	0.004586244
RP11-972P1.8	0.02203448	0.096509458	0.015603566	0.596919667	0.011904465
RP11-96K19.2	0.000611863	1.85767459	1.16904343	2.951947544	0.00876908
RP11-96D1.11	0.014689536	2.22E+32	112031.7695	4.38E+59	0.020204756
RP11-96B2.1	0.006336208	3.05675124	2.123413872	4.400333003	1.84E-09
RP11-95O2.5	0.035703365	0.623106335	0.416333664	0.932572929	0.021489248
RP11-95I16.2	0.022947569	0.391573726	0.181166044	0.846350559	0.017117485
RP11-93H12.4	0.007820534	8.542487653	2.533656802	28.80188637	0.00054186
RP11-92G12.3	0.044847156	1.524245308	1.102320892	2.10766554	0.010799427
RP11-923I11.8	0.018455166	0.013978829	0.000336258	0.58112456	0.024744033
RP11-91J19.2	0.000229805	0.033425594	0.00329002	0.339593793	0.004066149
RP11-90L1.8	0.029992391	1.413313955	1.012951451	1.971917149	0.041781671
RP11-902B17.1	0.000712684	0.251246104	0.104531027	0.603883902	0.002020322
RP11-8P13.5	1.43E-05	1.809335069	1.340880154	2.44145115	0.000105028
RP11-8L21.1	0.010283527	561.2448107	23.91086156	13173.75105	8.44E-05
RP11-89K21.1	0.000466897	1.355909219	1.10815322	1.659057409	0.003101545
RP11-896J10.3	0.000179249	0.551620312	0.381665296	0.797256056	0.001547166
RP11-890B15.2	0.014849971	1.756403421	1.021696105	3.019442828	0.041588966
RP11-88H9.2	0.015206315	0.190477913	0.036504094	0.993911417	0.0491591
RP11-887P2.3	0.043418589	2.190528098	1.205792859	3.979467378	0.010043315

RP11-882I15.1	0.029996765	4.31E-09	3.88E-17	0.478426925	0.041553641
RP11-881M11.1	0.010899572	0.039720373	0.002463712	0.640378329	0.022955888
RP11-87H9.5	0.003312965	0.243180491	0.08636298	0.684746538	0.007429677
RP11-87H9.2	0.00376133	0.599333203	0.413569726	0.868536225	0.006838926
RP11-87E22.2	0.003701741	0.094266396	0.013252953	0.670503654	0.018309706
RP11-876N24.5	0.003546689	0.674507338	0.515096035	0.88325306	0.004204767
RP11-876N24.4	0.000676683	0.553888828	0.378952898	0.809580388	0.002282483
RP11-876N24.3	0.019385174	0.772787689	0.645457429	0.925236561	0.005018145
RP11-875O11.1	0.000760271	0.262587812	0.123968841	0.556207177	0.00047974
RP11-874J12.4	0.02216001	1.351476239	1.036107113	1.762837067	0.026311059
RP11-872J21.3	0.030452431	0.673210216	0.490340795	0.924279603	0.014410207
RP11-863K10.7	0.032831104	0.09021302	0.010010095	0.813018149	0.031992881
RP11-861E21.1	0.001800128	0.68419542	0.498654015	0.938773897	0.018701587
RP11-861A13.3	0.045926651	0.433620657	0.201972362	0.930953488	0.032073098
RP11-856M7.6	0.043404657	0.163616206	0.028894867	0.926471216	0.040727293
RP11-84C13.2	0.000905099	0.480714956	0.257052894	0.898985675	0.02182641
RP11-848P1.7	0.012661402	0.724070337	0.56460273	0.928578317	0.01096592
RP11-848D3.4	0.008290834	990.3243017	10.30043465	95213.6736	0.003065495
RP11-845M18.6	0.049224708	1.210712493	1.003248794	1.461077999	0.046175547
RP11-83J21.3	0.042811209	3.349644692	1.400640008	8.01070903	0.006581029
RP11-83A24.2	0.000998554	0.379451945	0.193280855	0.744945889	0.004870849
RP11-83A24.1	0.028074926	0.092865732	0.011743974	0.73433782	0.024281553
RP11-831F12.4	0.038912278	16.58271052	2.264280851	121.4453093	0.005702062
RP11-830F9.6	0.000163702	0.0143151	0.000403322	0.50808529	0.019712746
RP11-830F9.5	0.008279324	0.25489478	0.081928864	0.793021473	0.018253906
RP11-82L20.1	0.009018953	0.065493361	0.006497531	0.660155404	0.02076468
RP11-82L18.2	0.030166481	0.780504678	0.66037371	0.922489104	0.003659851
RP11-82K18.2	0.004198635	0.4391168	0.212188929	0.908735274	0.026563758
RP11-81M19.1	1.35E-05	4.85E+76	5.28E+36	4.46E+116	0.000169245
RP11-817O13.9	0.017317681	0.403220161	0.256322703	0.634303931	8.52E-05
RP11-817I4.1	0.018539785	1.502036325	1.056268221	2.135928242	0.023530867
RP11-816B4.1	0.009959378	10.55951354	2.643839188	42.17477622	0.000849905

RP11-815I9.5	0.006018056	0.063066772	0.00488632	0.813990482	0.034203017
RP11-810P8.1	0.015884532	0.001368607	1.56E-05	0.119885092	0.003858701
RP11-80P20.3	0.000284245	0.512658671	0.348636196	0.753848612	0.000683097
RP11-809N8.4	0.012150097	0.390834034	0.206538367	0.739578047	0.003889102
RP11-809H16.4	0.01457273	5.537951517	2.137074325	14.35088459	0.000426407
RP11-807H22.7	0.001669058	0.320279885	0.120606139	0.85053054	0.022321416
RP11-806O11.1	0.01347791	0.831466802	0.725226382	0.953270675	0.008143128
RP11-806L2.5	0.013455971	2.490248115	1.192148736	5.201813738	0.015198774
RP11-805I24.3	0.003169703	0.004840282	9.30E-05	0.251984204	0.008205443
RP11-804H8.7	0.004969333	0.312353869	0.102800573	0.949069999	0.040154902
RP11-802O23.3	0.000239323	0.446308756	0.211354999	0.942449936	0.03439725
RP11-800A3.2	0.049063706	0.600198992	0.427123356	0.843407004	0.003269812
RP11-7O11.3	0.026671204	0.574626687	0.373640574	0.883725837	0.011641798
RP11-7I15.4	0.004723914	0.383127428	0.228768086	0.641639438	0.000265806
RP11-7F17.4	0.002560774	0.055822566	0.005170343	0.602698644	0.017450592
RP11-799D4.4	0.023931314	0.594830414	0.391187638	0.90448467	0.015121332
RP11-798K23.4	0.010213115	2474.645359	13.90092595	440536.8159	0.003122071
RP11-795J1.1	0.00420845	0.060519036	0.005487117	0.66748234	0.022020572
RP11-795H16.3	0.015872203	2364.348066	23.72207228	235651.4941	0.000937769
RP11-793H13.3	0.030730641	0.331954704	0.116843187	0.943092436	0.038457693
RP11-78O7.3	0.04820886	0.453189891	0.271483169	0.756514953	0.002467811
RP11-78I14.1	0.001577037	0.111657669	0.02307557	0.540287206	0.006424514
RP11-78A19.4	0.00024569	1.976044692	1.503458176	2.597180743	1.04E-06
RP11-789F5.1	0.007013685	1.33651E+14	38.58643531	4.63E+26	0.027249287
RP11-783K16.5	0.008153637	1.272021484	1.073471539	1.507295346	0.005456712
RP11-783K16.14	0.005814534	0.225354515	0.077166322	0.658119456	0.006428517
RP11-77H9.8	0.002222592	0.182401022	0.065484836	0.508058584	0.001131644
RP11-775D22.2	0.005164279	0.652855604	0.499755431	0.852858045	0.001764275
RP11-76K13.2	0.011196214	7.926925681	1.443080562	43.54306504	0.017220443
RP11-763K15.1	0.002807771	1421.175093	9.831646032	205432.4003	0.004227669
RP11-762H8.1	0.033999457	0.331399646	0.134862991	0.814350363	0.016055171
RP11-75L1.1	0.00209453	0.157249168	0.050803238	0.486726867	0.001331868

RP11-75C23.1	0.000327137	457.4668549	51.55737558	4059.087976	3.80E-08
RP11-758H9.2	0.030176564	0.71346434	0.522345246	0.974511339	0.033815895
RP11-750H9.5	0.000628311	0.689283265	0.540572237	0.878904589	0.00269119
RP11-74D3.2	8.72E-07	1.16705E+16	2446644376	5.57E+22	2.41E-06
RP11-749H20.3	0.016711069	28397488463	21.99332881	3.66665E+19	0.024530392
RP11-744K17.7	0.002010321	93.35709393	2.045711452	4260.398982	0.019958215
RP11-73M7.6	0.02355517	0.206437723	0.06413125	0.664520549	0.008165713
RP11-73M18.11	0.001701745	0.318895538	0.123513728	0.823344624	0.018195108
RP11-739N20.3	0.012448122	6.81978044	1.549233493	30.02091387	0.011120751
RP11-739N20.2	0.002808994	1.629211214	1.28767679	2.061331851	4.77E-05
RP11-736P16.1	0.018380175	1983694733	397085.9859	9.90981E+12	8.35E-07
RP11-735G4.1	0.001499712	0.022733083	0.000798013	0.647599409	0.026814616
RP11-71J2.1	0.009419871	2987792891	55.86148055	1.59804E+17	0.016258945
RP11-71G12.1	0.00332539	0.17421494	0.054469278	0.557210346	0.003220972
RP11-71B7.1	0.002255795	0.019690677	0.00040588	0.955265119	0.047359424
RP11-713M15.1	0.025271671	0.072054429	0.009131529	0.568562069	0.01257055
RP11-70P17.1	0.001958334	0.476465057	0.260106063	0.872793767	0.016372231
RP11-70F11.7	0.000585204	3.27E-05	1.22E-08	0.088067264	0.010378599
RP11-70F11.2	0.045820006	0.034246126	0.001243338	0.943264741	0.046098607
RP11-709D24.8	0.000977731	0.133294835	0.021471272	0.827501665	0.030524548
RP11-709D24.5	0.02181108	2.88E-08	1.79E-13	0.004631805	0.004529131
RP11-706P11.2	0.032282289	1.756557392	1.033453522	2.985614551	0.037383669
RP11-705C15.5	0.000991268	0.138756585	0.024768483	0.777334241	0.024674193
RP11-702F3.2	0.036105387	1.94E-09	1.08E-17	0.348345066	0.038572463
RP11-702F3.1	0.002355844	0.014195852	0.000428963	0.469789398	0.017167517
RP11-700H6.2	0.028788111	0.023017604	0.000535932	0.988577058	0.049304081
RP11-6F2.3	0.007868586	0.024805502	0.000629815	0.976973556	0.048564697
RP11-6E9.5	0.013778926	174.9543697	2.029149837	15084.65807	0.023138223
RP11-697E2.10	0.027035837	0.163676555	0.028967021	0.924845333	0.040521846
RP11-693J15.5	0.009863097	0.064561764	0.005677887	0.734114887	0.027163557
RP11-690P14.4	0.02201297	0.000556448	7.98E-06	0.038809797	0.000539868
RP11-690J15.1	0.002973586	0.000244385	4.93E-07	0.121082833	0.008619226

RP11-690D19.3	0.041318626	0.565043475	0.341736181	0.934270783	0.026084049
RP11-690C23.3	0.002829972	0.337665318	0.12929077	0.88187167	0.026649281
RP11-68I3.4	0.038400485	0.647814738	0.446159982	0.940613127	0.022505241
RP11-689K5.3	0.026339474	1.252497528	1.029152665	1.524312293	0.024657541
RP11-687F6.5	0.022581838	0.034790545	0.001359924	0.89003678	0.042316731
RP11-686G8.2	0.034712117	0.551227102	0.342770233	0.886457715	0.014003426
RP11-686D22.7	0.02876715	0.755323048	0.588042346	0.970190175	0.028027987
RP11-686D22.5	0.004351275	0.381107131	0.199504529	0.728016783	0.003486862
RP11-680F8.1	0.028343193	1.387530603	1.145132545	1.681238719	0.00082777
RP11-67H24.3	0.038719544	1.54E+43	33006508.51	7.17E+78	0.017639137
RP11-678G14.3	0.002022022	0.604393119	0.404790782	0.90241937	0.013816684
RP11-677M14.3	0.004619622	0.685134713	0.498930844	0.94083094	0.019444833
RP11-66N24.6	0.003766772	0.852564902	0.766187207	0.948680565	0.00342707
RP11-667K14.3	0.030481546	0.576002388	0.393446124	0.843263488	0.004560376
RP11-666O2.2	0.00020472	0.644661926	0.462219949	0.899115235	0.009696352
RP11-666F17.1	0.025542822	0.138940277	0.02717615	0.710343463	0.017750874
RP11-664D7.4	0.036790783	0.871399376	0.774972726	0.979823995	0.021413597
RP11-664D1.1	0.011692505	0.075547686	0.014215838	0.401485496	0.002439555
RP11-662I13.3	0.006294668	0.018304234	0.000975139	0.343586829	0.007494585
RP11-660M5.1	0.006111628	0.084587656	0.009352919	0.76500949	0.027922236
RP11-660L16.2	0.024873635	0.76864539	0.633836888	0.93212583	0.007487419
RP11-655M14.4	0.003695639	0.533897908	0.326990794	0.871727833	0.012115555
RP11-647P12.2	0.001203687	0.643353267	0.420722484	0.983792028	0.041813465
RP11-646I6.5	0.046756311	0.486414748	0.281665741	0.840000299	0.009725339
RP11-635O16.2	0.00507755	0.715399812	0.550559011	0.929594978	0.012200172
RP11-632L2.2	0.007647057	1.986255362	1.195461096	3.300157885	0.008069297
RP11-632K21.6	0.003852676	5.91E-05	1.51E-08	0.230698107	0.021022717
RP11-630A13.4	0.038782522	0.730773992	0.55681016	0.959089228	0.023753713
RP11-62I21.1	0.019148929	0.519820076	0.327141936	0.825980658	0.005620694
RP11-62H7.3	0.000738776	0.387951618	0.240591408	0.625568716	0.000102628
RP11-62H7.2	0.008988215	0.603620511	0.45418413	0.802224689	0.000504404
RP11-62G11.2	0.005511634	8.40E-13	8.61E-24	0.08196031	0.031261602

RP11-627K11.3	0.002106601	0.302192431	0.094451298	0.966850297	0.043718687
RP11-627G23.1	0.045781443	0.766040555	0.627663952	0.934924063	0.008743043
RP11-626K17.2	0.018574534	33.12092157	2.160532617	507.7430616	0.011968645
RP11-626E13.1	0.015333536	0.367209376	0.145552999	0.926416677	0.033850851
RP11-624M8.1	0.001146257	0.001682449	1.77E-05	0.159603915	0.005959302
RP11-622J9.1	0.02877795	21401.36135	29.82827541	15355170.93	0.002958558
RP11-61K20.1	0.019472217	11127292.01	1.242714372	9.9634E+13	0.046970086
RP11-61I13.3	0.015716658	0.440206506	0.210506117	0.92055172	0.029265207
RP11-618I10.3	1.37E-05	1.25234E+18	7655588.549	2.05E+29	0.001560656
RP11-617F23.1	0.020625736	0.745365732	0.590317404	0.941137887	0.013518667
RP11-616M22.5	0.014048239	0.787828611	0.631116491	0.983453814	0.035082386
RP11-616K22.2	0.024188489	0.096355461	0.012928562	0.71812897	0.022427311
RP11-607P23.1	0.042983564	0.299705289	0.103527376	0.867628101	0.02629827
RP11-603J24.21	0.034933125	0.386852523	0.209964141	0.712763969	0.002319463
RP11-598F7.3	0.047202905	0.743902337	0.583726547	0.948030702	0.016786752
RP11-598F17.2	7.73E-12	2.32E+24	5.58357E+11	9.63E+36	0.000153965
RP11-595O22.1	0.017633276	2.25E+28	1.95186E+11	2.60E+45	0.00112631
RP11-58O9.2	7.00E-05	1.39684581	1.2151112	1.605761035	2.61E-06
RP11-58O3.1	0.001839467	56641.05219	328.8271404	9756520.673	3.10E-05
RP11-586K12.10	0.008694807	2.43E+125	2.83404E+12	2.08E+238	0.029549455
RP11-582J16.4	0.004508388	0.151673474	0.053313227	0.431503468	0.000406982
RP11-573D15.8	0.009882889	63.7023345	2.55323557	1589.350967	0.011371247
RP11-571F15.3	0.000319737	0.268277271	0.115081966	0.625403759	0.002312442
RP11-56B16.2	0.026831096	0.460776803	0.212954259	0.996999371	0.049114645
RP11-568N6.1	0.00643804	0.499200637	0.253025468	0.984886137	0.045081806
RP11-567M16.6	0.005674434	0.602114768	0.442065417	0.82010983	0.001291266
RP11-567L7.3	0.01496058	0.036451039	0.002092883	0.634855622	0.02310968
RP11-566K19.12	0.038193212	1.08E-15	1.95E-30	0.602000054	0.04667245
RP11-566K11.5	0.024184041	1.88722E+15	2497.75971	1.43E+27	0.011716362
RP11-565F19.2	0.000575188	0.367721879	0.184376663	0.733386634	0.004506837
RP11-564H18.1	0.00763204	624029.9928	125.5820247	3100869196	0.0021197
RP11-564A8.8	0.007886255	0.452436875	0.235486984	0.869258772	0.017288277

RP11-563J2.3	0.021108757	0.335836432	0.149386029	0.754997704	0.008292454
RP11-563J2.2	0.0244639	0.221086123	0.079440758	0.615289618	0.003853009
RP11-560F18.1	0.038941295	90.29584267	4.652305256	1752.537453	0.002920706
RP11-558F24.2	0.018856575	0.000481295	1.41E-06	0.164379019	0.010269636
RP11-558A11.3	0.001372334	5.635049013	2.18776742	14.51423816	0.000341288
RP11-557H15.4	0.000647793	0.370524701	0.185597522	0.739711138	0.004882114
RP11-555H7.2	0.048065868	0.002818337	8.98E-06	0.88467148	0.045312727
RP11-554J4.1	0.018966991	0.601320876	0.420034387	0.860850463	0.005461712
RP11-554F20.1	0.015105207	0.091690383	0.009259579	0.907938251	0.041099539
RP11-553L6.3	0.023436964	0.003269546	1.96E-05	0.544988519	0.028343438
RP11-553L6.2	7.36E-05	0.00321953	4.24E-05	0.244627388	0.009397988
RP11-553D4.2	0.017345786	0.208055434	0.061006621	0.709546979	0.012136713
RP11-552M11.4	0.003236625	0.350534708	0.141998022	0.865326007	0.022984013
RP11-552J9.11	0.004062048	505833.7573	5.561296464	46008658535	0.024165191
RP11-551L14.7	0.031784739	8.081448655	1.163675083	56.12375249	0.034578003
RP11-54J7.2	1.29E-14	2.12E+30	2.30899E+15	1.96E+45	7.12E-05
RP11-54H7.4	0.027869393	1.3088062	1.168241739	1.466283571	3.44E-06
RP11-549B18.1	0.039884217	1.441317444	1.129198424	1.839708531	0.003326932
RP11-548P2.2	0.040907015	0.653419541	0.49867358	0.856185517	0.002028967
RP11-545M17.1	0.004511503	0.10317743	0.013900143	0.765861338	0.026365544
RP11-543C4.1	0.040365452	0.085948342	0.009693312	0.76208395	0.027525727
RP11-542M13.3	0.004507468	0.260619981	0.070238056	0.967036658	0.044423932
RP11-541H12.1	0.012714497	0.040023991	0.00311814	0.513742166	0.01345714
RP11-53M11.3	0.003985529	1.915265565	1.375659248	2.666534021	0.000118643
RP11-53B2.6	0.048385503	0.464909349	0.230334263	0.938378423	0.032560573
RP11-53B2.5	0.00150713	0.510048196	0.267424902	0.972793334	0.040983596
RP11-53B2.2	0.006122809	0.188539354	0.043241743	0.822054941	0.026365893
RP11-539E17.5	0.000847274	2.124801969	1.503088692	3.003670662	1.98E-05
RP11-531A21.4	0.011826503	0.1179257	0.016361682	0.849941405	0.033896157
RP11-523O18.5	0.003301367	0.124030044	0.0274364	0.56069499	0.006695474
RP11-522L3.3	0.035995061	0.017430594	0.000361606	0.840211596	0.040558594
RP11-521C20.2	0.02737086	0.355929594	0.16269061	0.778691999	0.009704223

RP11-51J9.5	0.004959483	0.737109145	0.586951514	0.925681046	0.008679628
RP11-514F8.2	0.021920495	1271.76398	20.27782315	79761.20563	0.00071124
RP11-514D23.2	0.00978491	0.051264	0.005468494	0.480570676	0.009275953
RP11-513N24.1	0.00024522	0.294944667	0.105430544	0.825115313	0.020007464
RP11-513G19.1	0.03662424	0.463270413	0.260443438	0.824054072	0.008830694
RP11-513G11.4	0.012793072	0.792944221	0.637918685	0.985643707	0.036595736
RP11-511H9.3	0.029028063	0.335222058	0.128232908	0.876325976	0.025798674
RP11-510M2.1	0.00231432	7.992423738	1.46462488	43.61446952	0.016362747
RP11-508N12.4	0.022810073	0.004754888	9.58E-05	0.236084084	0.00726375
RP11-508M8.1	0.00263415	0.001742406	3.34E-05	0.090769533	0.001634798
RP11-506K6.4	0.014510524	0.419738659	0.214508153	0.821323289	0.011255088
RP11-501J20.5	0.039408822	0.247835597	0.074676749	0.822511472	0.022654761
RP11-501C14.7	0.04416165	1.709081286	1.314049076	2.222868913	6.43E-05
RP11-501C14.6	0.014066317	4.423221311	1.964459262	9.959426058	0.000330084
RP11-501C14.5	0.015174156	1.549494741	1.053062345	2.279954234	0.026260624
RP11-4M23.3	0.00479064	0.030244892	0.001133984	0.806672334	0.036779806
RP11-4M23.2	0.012686548	0.083341585	0.011150783	0.622899744	0.015468519
RP11-4K3__A.3	0.006769738	0.528399713	0.328768689	0.84924832	0.008415733
RP11-4C20.3	0.027457419	0.602123014	0.368103673	0.984918516	0.04333266
RP11-4B16.3	0.003839804	0.275033974	0.114076602	0.663095548	0.004040711
RP11-49G2.3	0.02271267	0.196289686	0.068778479	0.560199081	0.002342723
RP11-49G10.3	0.000432351	397616.7027	122.1964474	1293810463	0.001780668
RP11-497G19.3	0.001476207	0.107545422	0.021989381	0.525981973	0.005900358
RP11-497D6.5	0.01553779	5.01E-18	2.52E-34	0.099802112	0.03749639
RP11-496H15.2	0.04743426	0.094085865	0.009791312	0.904082125	0.040627908
RP11-492E3.51	0.046324601	0.544674798	0.314981023	0.941868282	0.02968328
RP11-490P13.2	0.008694807	2.96255E+14	27.36719424	3.21E+27	0.029549455
RP11-490O6.2	0.000695582	0.141796688	0.043487282	0.462348991	0.001198572
RP11-489O18.1	0.037014303	0.349869643	0.148009546	0.827032924	0.016727959
RP11-488P3.1	0.005690796	1.487026883	1.290983295	1.712840871	3.78E-08
RP11-488L18.4	0.004207761	0.804102577	0.670207313	0.964747686	0.01896828
RP11-488L18.3	0.021002167	0.518732997	0.275105778	0.978110761	0.042523587

RP11-488C13.1	0.00360778	1.390744754	1.043820513	1.852972754	0.024265559
RP11-487E1.2	0.043244799	0.314043463	0.114310645	0.862765636	0.024688595
RP11-486I11.2	0.001001002	0.732585001	0.557889581	0.961983878	0.025168111
RP11-481H12.1	0.01750367	0.722174174	0.582616413	0.89516108	0.00297003
RP11-481G8.2	0.035848304	8.27E-07	4.76E-12	0.143438924	0.022879544
RP11-481A20.10	0.031579753	3.259316106	1.057069426	10.04961568	0.039727714
RP11-479G22.8	0.000127525	1.786644725	1.408339509	2.266569499	1.75E-06
RP11-476C8.2	0.005283575	0.037237024	0.002253306	0.615360744	0.021490974
RP11-475O23.2	0.000350998	0.312154257	0.121656845	0.800943668	0.01545026
RP11-475D12.1	0.016553813	1097880.473	4.78038601	2.52143E+11	0.027218551
RP11-474N24.6	0.001565437	0.622544243	0.431873727	0.897395025	0.011078698
RP11-474B16.1	0.027512996	9.244017978	1.808669837	47.24569772	0.007542083
RP11-473E2.3	0.016330567	40.03877172	2.290900342	699.7699601	0.011475936
RP11-470M17.2	0.045247108	0.878981896	0.780768513	0.989549605	0.032863918
RP11-470B22.1	0.000251716	19.82325524	3.670335266	107.0641835	0.000518483
RP11-46J23.1	0.003068485	0.537574773	0.327952603	0.881184153	0.01383147
RP11-46H11.3	0.003452743	0.374419358	0.198700194	0.705534568	0.002373903
RP11-468H14.2	0.007921836	0.00524565	8.88E-05	0.309871768	0.011637472
RP11-468E2.10	0.0430117	0.708469091	0.511401284	0.981476717	0.038229098
RP11-466I1.1	0.02070921	17.70506461	2.93733391	106.7189916	0.001714984
RP11-463I20.2	0.011264051	47.9812528	1.293442472	1779.901828	0.035770433
RP11-462L8.1	0.000224622	1.482103496	1.265463374	1.735831172	1.06E-06
RP11-462G12.1	0.019324585	0.738813842	0.576300861	0.947154394	0.016925208
RP11-461K13.1	0.003050138	94244.78887	31.25604852	284171565.2	0.005077324
RP11-459F1.2	9.67E-05	478.45458	27.37278433	8363.006931	2.37E-05
RP11-457D20.1	0.014583406	8.70188385	1.842371958	41.10070294	0.006306586
RP11-455O6.2	0.008326503	0.306028434	0.131266648	0.713459236	0.006111241
RP11-455F5.5	0.012091356	0.61975702	0.429560186	0.894167516	0.010525436
RP11-454E5.4	0.027108461	0.485452657	0.315122823	0.747848981	0.00104608
RP11-453O22.1	0.005084353	5.301279629	2.18148875	12.88274611	0.000231697
RP11-450I1.2	9.22E-06	1.70E+95	8.36E+33	3.45E+156	0.002331221
RP11-44F14.6	0.004347514	0.499528714	0.310915183	0.802562725	0.004115797

RP11-449J21.3	0.042069867	0.810277182	0.676031341	0.97118147	0.022823766
RP11-449H15.2	0.033015026	1.20E-10	2.18E-20	0.664336374	0.0459669
RP11-448P19.1	0.026584535	5.64E-05	4.72E-09	0.672367851	0.041067536
RP11-440L16.1	0.006324977	1710607060	132.0527448	2.21591E+16	0.010947252
RP11-440D17.6	0.000178978	9.28E+29	7.41275E+16	1.16E+43	7.31E-06
RP11-439E19.9	0.003580714	0.180400673	0.049231086	0.661053931	0.009747434
RP11-438L7.1	0.001385233	42.98993767	2.954534711	625.5248022	0.005906155
RP11-438D14.3	0.000622536	2.829670122	1.773489044	4.514847738	1.28E-05
RP11-438D14.2	0.014202489	3.831150816	2.0013402	7.333943813	5.03E-05
RP11-438B23.2	7.71E-06	12.08591173	4.830845969	30.23678737	1.00E-07
RP11-434I12.2	0.045600955	0.621941627	0.38934739	0.993486531	0.046888113
RP11-432M8.19	0.006278335	830.6641242	1.155172411	597315.9338	0.045183258
RP11-429A20.2	0.039317384	0.000160707	1.00E-07	0.25713116	0.0202991
RP11-427P5.3	0.035048723	0.684398849	0.484393404	0.966986299	0.03152933
RP11-424M22.1	0.013367428	0.104712117	0.012521413	0.875670139	0.037297797
RP11-424C20.2	0.007557906	1.756846643	1.257700021	2.454090861	0.000951485
RP11-421P23.2	0.026670685	0.016052908	0.000340255	0.757360524	0.035614565
RP11-420K14.6	0.000115267	0.038662336	0.003834515	0.389821469	0.005797965
RP11-420C9.1	0.002771208	1.716817572	1.161697224	2.537203769	0.006686543
RP11-41O4.2	0.004029352	16.08562498	5.781385257	44.75524801	1.03E-07
RP11-418I22.2	0.048242479	0.04606236	0.004191683	0.506178786	0.011845482
RP11-417L19.4	7.37E-05	1.309077011	1.014153165	1.689767069	0.038652164
RP11-417L14.1	0.001016364	4.532201578	1.29680655	15.83956461	0.017929893
RP11-417F21.1	0.025703436	0.412520191	0.183879627	0.925458199	0.031723847
RP11-417E7.1	0.030396192	1.358717999	1.058985697	1.743285678	0.015923404
RP11-415F23.2	0.013296214	0.427662593	0.272633255	0.670847339	0.000217365
RP11-411G2.1	0.00093028	1.964710683	1.210794828	3.188061244	0.006248764
RP11-410N8.4	0.006102151	1588.187643	2.71489077	929076.0484	0.02337865
RP11-410D17.2	0.021531445	6.37E-06	1.48E-10	0.274178425	0.027974179
RP11-409C19.2	0.03993511	0.118883013	0.018001409	0.785114715	0.027025311
RP11-406H23.2	0.015137505	0.069727324	0.014380396	0.33809219	0.000945452
RP11-404O13.5	0.00477848	0.056528507	0.003820063	0.836497327	0.036633239

RP11-404O13.4	0.001220575	0.306394471	0.126178953	0.744003415	0.008968477
RP11-404K5.2	0.007318835	6.02E-07	5.01E-12	0.072235713	0.016380214
RP11-404J24.1	0.039939549	1843.929768	64.61051996	52624.201	1.09E-05
RP11-404F10.6	0.0308525	0.239059671	0.080228524	0.712334257	0.010202772
RP11-403P17.5	0.013500926	0.008424499	0.000231592	0.306453281	0.009188761
RP11-403A3.3	0.012058004	0.346125188	0.128426964	0.932846512	0.035959383
RP11-401P9.5	0.001487886	0.293197079	0.09357345	0.918685029	0.0352469
RP11-401P9.4	0.049525131	0.662660492	0.507115268	0.865915416	0.002572192
RP11-401N18.1	0.019768977	76474248772	243.3079991	2.40367E+19	0.012061344
RP11-401F2.2	0.038675598	100.6927268	4.826426019	2100.73151	0.002925031
RP11-400F19.18	0.045202793	0.594227152	0.365519943	0.966037325	0.035788071
RP11-3N2.11	0.000178018	566875.1084	68.87970594	4665342051	0.003975826
RP11-399H11.2	0.035067402	0.461077212	0.24343424	0.873304411	0.017517176
RP11-398K22.12	0.00221025	0.50794655	0.260900924	0.988918301	0.046289197
RP11-398A8.2	0.039313451	0.022025814	0.000499439	0.971363015	0.048267754
RP11-397A15.4	0.017446962	3.641016815	1.012750997	13.09009173	0.047774213
RP11-395G23.3	0.039267473	1.252955461	1.011121526	1.552629774	0.039298235
RP11-390E23.6	0.00536204	0.705409179	0.50941512	0.976810643	0.035620403
RP11-38M8.1	0.018626067	1.394870221	1.102724406	1.764414503	0.005512587
RP11-38G5.3	0.014152209	77.81805857	2.0598213	2939.891067	0.01877629
RP11-389C8.2	0.009676285	0.679207778	0.488219947	0.944908558	0.021655194
RP11-387M24.5	0.01719637	0.543729215	0.323662078	0.913426314	0.02132994
RP11-386O9.2	0.01208456	0.027061484	0.002208818	0.331545644	0.004749901
RP11-385M4.3	0.034934055	43.83676775	1.878792174	1022.817869	0.018654326
RP11-385D13.3	0.021239012	0.446929925	0.247730036	0.806306579	0.007471137
RP11-384O8.1	0.000365274	1.536208944	1.211225972	1.948387809	0.000399849
RP11-383J24.1	0.009346607	1.473051691	1.088589312	1.993296518	0.012072373
RP11-380I10.4	0.001220062	0.023017283	0.002703674	0.195953847	0.000557311
RP11-380I10.2	0.004967046	0.002179852	1.02E-05	0.463842123	0.025035355
RP11-379F4.9	0.007360173	0.356209496	0.127125409	0.998110496	0.049580946
RP11-378G13.2	0.005433652	4.771987759	2.015560754	11.29803065	0.000379605
RP11-378A13.1	0.005300068	0.555586094	0.364972768	0.845750518	0.006118047

RP11-375N15.2	0.044670405	0.242120506	0.065148337	0.899828636	0.034213065
RP11-374M1.3	0.003031584	185967803.9	1.176473737	2.93963E+16	0.048060069
RP11-373A6.1	0.023724009	5.303353936	1.465989008	19.18538463	0.01098876
RP11-372B4.3	0.00087678	0.576180485	0.377500893	0.879425604	0.010603009
RP11-370I10.6	0.047902515	0.177278295	0.037623464	0.835318982	0.02870712
RP11-370I10.4	0.041156987	0.431333323	0.186942049	0.995219839	0.048701391
RP11-370I10.2	0.000122062	0.302339862	0.108782914	0.840291814	0.021813277
RP11-367J7.3	0.017553588	0.07357504	0.005609465	0.965027309	0.046914395
RP11-367G6.3	0.003553798	0.559508212	0.337747575	0.926873982	0.024144164
RP11-363N22.3	0.012836517	0.291901188	0.122498985	0.695567425	0.00544606
RP11-363G10.3	0.022276604	0.050840858	0.003933005	0.6572056	0.022523375
RP11-361L15.5	0.004578773	0.573285044	0.361468217	0.909224455	0.018060664
RP11-360A10.1	0.016083239	0.319592231	0.150920298	0.676775725	0.002884076
RP11-35G9.5	0.010123269	0.442382426	0.229523337	0.852646243	0.014845783
RP11-359N11.1	0.000261668	0.200531921	0.076136277	0.528172022	0.001146628
RP11-359M6.1	0.006226523	0.739423692	0.593580691	0.921100374	0.007077783
RP11-357J22.1	0.024350642	6.13E-06	4.27E-11	0.881370004	0.047613145
RP11-354P11.3	0.014399997	0.050235088	0.006137254	0.411187827	0.005295518
RP11-354E11.2	0.001940237	0.372831712	0.191314992	0.726568704	0.003752045
RP11-352M15.2	0.02240517	0.514332429	0.317680038	0.832717879	0.006838351
RP11-351I24.1	0.039481537	3.797005799	1.992235034	7.236722973	5.02E-05
RP11-34P1.1	0.016424446	0.018261896	0.000781528	0.426724161	0.012787707
RP11-349G13.3	0.030988054	0.069945266	0.010429857	0.4690707	0.00615123
RP11-348N5.7	0.014820196	0.457260714	0.230475983	0.90719804	0.025182503
RP11-347P5.1	0.005060165	0.304851502	0.131239612	0.708127954	0.005734682
RP11-347E10.1	0.012870125	1.52510323	1.184254288	1.964054415	0.001074215
RP11-347C18.3	0.000734578	0.445995746	0.302131293	0.658363467	4.83E-05
RP11-346E8.1	0.016836621	0.025874585	0.001397537	0.479052973	0.014120227
RP11-346C16.2	0.000965706	5.55E-05	1.47E-08	0.210052141	0.019743546
RP11-345P4.9	0.000110471	0.522804684	0.401106108	0.681427514	1.61E-06
RP11-344B5.2	0.000281507	0.73409887	0.613627873	0.878221435	0.000725366
RP11-343J3.7	0.026137111	3728.00442	1.053341408	13194218.75	0.048560733

RP11-342M3.5	0.001234273	0.014469899	0.000701123	0.298632219	0.006098253
RP11-342K6.1	0.027944966	1.648998791	1.205377815	2.25588772	0.001758587
RP11-341B24.3	0.023206408	0.189414432	0.062573722	0.573368918	0.003237426
RP11-341A22.1	1.34E-09	26219817.17	8364.563095	82189446686	3.20E-05
RP11-33B1.1	0.041385192	0.563026873	0.373757259	0.848142082	0.005998445
RP11-335L23.4	0.007410441	7.05930897	1.200271344	41.51881437	0.030626029
RP11-333E13.4	0.00460954	0.251834489	0.06395587	0.991630787	0.048611599
RP11-330H6.6	0.004222348	0.169256803	0.034674023	0.826205413	0.02809363
RP11-330C7.4	0.029595386	0.025338844	0.000697884	0.920005859	0.044914223
RP11-32B5.1	0.0433024	0.0004594	8.94E-07	0.236198226	0.015819643
RP11-328C8.4	0.005901088	7.23E-06	3.63E-09	0.014411783	0.002260459
RP11-327L3.3	0.002501719	0.147257718	0.033073326	0.655659342	0.011939332
RP11-327F22.1	0.01162284	0.326704313	0.116964861	0.912545074	0.032794603
RP11-327E2.5	0.007664472	0.461250503	0.247715667	0.858855757	0.014700486
RP11-326L9.1	0.000815597	1.18E+21	810953.6232	1.71E+36	0.006452906
RP11-326K13.4	0.003355474	0.23231834	0.102136597	0.528427739	0.000499142
RP11-326I11.3	0.002889364	0.628152747	0.437472812	0.901943763	0.011765856
RP11-325L7.2	0.031157011	0.024675468	0.000962969	0.632293228	0.025288898
RP11-325K4.3	0.012521101	0.659653267	0.489803254	0.888402494	0.006162887
RP11-325K4.2	0.000609736	0.580308855	0.418558775	0.804566496	0.001097176
RP11-325I22.3	0.022888157	0.503519779	0.267204946	0.948830371	0.03380059
RP11-325F22.2	0.000347799	0.649445381	0.455446472	0.926078758	0.017118756
RP11-323F24.3	0.033032966	0.182450798	0.062489664	0.532700794	0.001858284
RP11-322A17.3	0.000378925	8.35E+25	2.20618E+12	3.16E+39	0.000182744
RP11-321E2.4	0.023215624	162.8994328	4.548841862	5833.622275	0.005275306
RP11-320N7.2	0.020984957	0.844496742	0.730729038	0.975977018	0.022060067
RP11-316I3.2	1.49E-05	9.903241487	2.190701628	44.76839323	0.002893861
RP11-316I3.1	0.000283885	23.9453759	6.43894715	89.0488792	2.15E-06
RP11-315L6.1	0.028620499	16399.96556	121.1649193	2219775.094	0.000106315
RP11-315A16.3	0.00406638	26055586.32	28.28098158	2.40053E+13	0.01481215
RP11-313E19.1	0.001263652	393669.7964	7.303060984	21220678417	0.020468331
RP11-310E22.6	0.01077843	0.355366885	0.162541506	0.776943846	0.009532043

RP11-30P6.6	0.005805069	1.729931397	1.147152863	2.608774066	0.008923889
RP11-30B1.1	0.001263628	0.037996271	0.002764015	0.522325884	0.014458554
RP11-308D13.3	0.000533258	2.457661906	1.255176594	4.812153185	0.008718336
RP11-304F15.3	0.043947874	0.778869738	0.609627367	0.995096516	0.045577171
RP11-303E16.6	0.00850633	1.617750855	1.123341425	2.329761701	0.009738677
RP11-303E16.2	0.003017084	1.280204216	1.063017416	1.541764802	0.009207802
RP11-302K17.3	0.01732738	0.383797417	0.154612981	0.952704334	0.038978826
RP11-302F12.5	0.005437453	0.012543526	0.000334071	0.470977765	0.017933045
RP11-302F12.1	0.009557323	0.855104603	0.771300104	0.948014759	0.002935825
RP11-300J18.1	0.003816554	0.043374198	0.004039525	0.465728258	0.009572112
RP11-2I17.1	0.007521407	0.001102961	1.49E-05	0.081375117	0.001914712
RP11-2H3.6	0.001888603	0.467168931	0.27250188	0.800900198	0.005653612
RP11-2C24.7	0.004880404	0.434677129	0.206498571	0.914990383	0.028242053
RP11-299J5.1	0.04329244	138.3617331	1.325299021	14445.01874	0.037643195
RP11-297L17.2	0.014540245	1.975979708	1.411804857	2.765605873	7.17E-05
RP11-297B17.3	0.007582399	0.333101964	0.120564337	0.920312934	0.033996141
RP11-296L22.8	0.002692244	0.003877978	3.73E-05	0.403542989	0.019135758
RP11-296A18.6	0.034949725	0.381237965	0.161745431	0.898587273	0.027496035
RP11-295P9.12	0.030466179	0.625280495	0.409463311	0.954849158	0.029715556
RP11-295D4.4	0.000255201	0.384564621	0.205014484	0.721363411	0.002904796
RP11-293P20.4	0.038485512	299.5808802	1.520274336	59034.54503	0.034399647
RP11-293A21.1	0.001160751	0.561549169	0.397733722	0.792835638	0.001041464
RP11-290L7.5	0.00063575	824352538.9	4217.80251	1.61116E+14	0.000957209
RP11-286H15.1	0.024191609	0.490417306	0.285713889	0.841783139	0.009743932
RP11-285G1.14	0.024300097	0.402580173	0.197701088	0.819776954	0.012153237
RP11-285E9.5	0.001366116	0.673106566	0.457882996	0.989493939	0.044041999
RP11-285E18.2	0.000977355	6.22922E+12	31019.57219	1.25E+21	0.002525557
RP11-285A15.1	0.000479048	771604689.7	1586.15945	3.75356E+14	0.002191886
RP11-284F21.9	0.006716715	1.285227422	1.139914252	1.449064719	4.15E-05
RP11-284F21.7	0.033422423	1.247639799	1.105909461	1.40753391	0.000322907
RP11-284F21.10	0.018797413	1.212043178	1.097989481	1.337944206	0.000136788
RP11-284B18.3	0.01019215	0.041188694	0.004978269	0.340782825	0.003091723

RP11-282O18.7	0.016786742	0.43966398	0.20898176	0.924982233	0.030352582
RP11-281O15.2	0.009171242	0.002601174	2.21E-05	0.305890502	0.014406736
RP11-27G24.1	0.046795221	0.335472668	0.157782324	0.713273246	0.004541041
RP11-279O22.1	0.022518386	1696786.058	10.17558054	2.8294E+11	0.019380792
RP11-279F6.1	0.002883282	0.876088856	0.785076521	0.977652068	0.018087372
RP11-278H7.1	0.015827545	0.001527291	2.09E-05	0.111466609	0.003053574
RP11-274J16.5	0.000394525	2.665154301	1.206022079	5.889649597	0.015393049
RP11-26O3.1	0.040047834	4881.265909	4.189221119	5687634.096	0.018392724
RP11-26N15.2	0.031064387	0.013475187	0.000330687	0.54910201	0.022793451
RP11-26L20.4	0.018039054	0.214815318	0.060272515	0.765616314	0.0176997
RP11-269F21.1	0.017555328	5.390974272	1.802875167	16.12014195	0.002573337
RP11-268F1.3	0.017446698	0.541922692	0.338190567	0.86838674	0.010879164
RP11-267M23.1	0.014212145	1.508668198	1.115623689	2.04018591	0.007574042
RP11-267J23.4	0.041268124	1.293868329	1.003239266	1.668689922	0.047157872
RP11-266L9.8	0.038194594	0.784194153	0.627878106	0.979426521	0.032093957
RP11-266L9.4	0.013338871	0.589018352	0.390409299	0.888663819	0.011652609
RP11-264B17.4	0.011982863	0.51110087	0.263708585	0.990578673	0.046811533
RP11-264B14.1	0.001979732	0.295830877	0.132582614	0.660085855	0.002935952
RP11-263K4.3	0.020292836	1.84E-05	1.22E-09	0.279162892	0.026432909
RP11-263K4.1	0.037843935	0.133780191	0.020767059	0.861804229	0.034306873
RP11-263C24.1	0.001954815	0.005825188	8.81E-05	0.385131289	0.016121645
RP11-259N19.1	0.000836504	1.645783844	1.281943366	2.112889331	9.29E-05
RP11-259K15.2	0.015583033	0.830245693	0.726013281	0.949442564	0.006569216
RP11-255I10.2	0.035691423	3832246620	13054.09288	1.12502E+15	0.000591894
RP11-255H23.2	0.01992838	0.734492199	0.577960478	0.933418133	0.01162198
RP11-255G12.3	4.01E-06	3.599678011	2.125475194	6.096369329	1.89E-06
RP11-253M7.4	0.023217744	0.464456674	0.265980877	0.811035757	0.007010127
RP11-253E3.3	0.041509022	1.50603554	1.078838351	2.102393786	0.01613676
RP11-252A24.2	0.03678481	0.501267324	0.302677272	0.830154601	0.007292932
RP11-251M1.1	0.049398861	0.492260068	0.322255967	0.751948758	0.001042311
RP11-248J23.7	0.002293961	0.000284614	2.37E-06	0.034211733	0.000834019
RP11-248E9.1	0.023735171	18.87284799	5.069142157	70.26522045	1.19E-05

RP11-247I13.11	0.02960616	0.415228731	0.199203027	0.86552349	0.019009813
RP11-245J9.6	0.008322634	0.440265469	0.197197532	0.98294173	0.045290103
RP11-245J9.5	0.021700446	0.505741865	0.301897118	0.847225158	0.009604122
RP11-245G13.2	0.022662934	0.383745013	0.157542691	0.934732254	0.034982993
RP11-245D16.4	0.004440937	0.605016974	0.416915467	0.87798503	0.008172151
RP11-243M5.4	0.027933915	0.178960888	0.035645584	0.898484352	0.036618484
RP11-243M5.3	0.000379015	0.413892714	0.214155439	0.799919814	0.008689862
RP11-243M5.2	0.003005556	0.441573103	0.197462795	0.987460981	0.046514526
RP11-242D8.1	0.01693488	0.649687337	0.471169767	0.895841936	0.00851392
RP11-23P13.6	0.021739216	0.602299657	0.369665365	0.98133315	0.0417885
RP11-23N2.4	0.007184463	0.656630226	0.431353551	0.999558839	0.049759899
RP11-23J9.4	0.002112699	0.010228197	0.000226521	0.461838794	0.018404825
RP11-23J18.1	0.000671861	0.539946423	0.361756167	0.805907864	0.002561531
RP11-23B7.4	0.0016744	14236.98118	24.44967182	8290157.613	0.00324014
RP11-238I10.1	0.047353402	4.818105311	1.260078734	18.42276849	0.021573801
RP11-235G24.3	0.000178767	1.611952862	1.218250979	2.132887288	0.000832661
RP11-235G24.2	0.015528249	744.5527242	8.065643829	68730.87516	0.004181148
RP11-230F18.5	0.045613655	1.662668318	1.138147309	2.428917518	0.008560748
RP11-22C11.2	0.001819701	0.71252801	0.52720116	0.963002746	0.027436678
RP11-227F8.2	0.039005706	4.23E-05	4.71E-09	0.379000194	0.030090283
RP11-224O19.5	0.014319577	0.090257316	0.008193916	0.994198955	0.049447041
RP11-221N13.4	0.019137126	2.078799399	1.334961865	3.237101412	0.001201686
RP11-21I4.4	0.011961422	820.9501513	9.631283943	69976.0442	0.003090471
RP11-21C4.1	0.038021497	0.142263353	0.020792292	0.973382888	0.046871564
RP11-21B23.2	0.00103701	1.577197767	1.3398934	1.856530375	4.33E-08
RP11-219H23.1	0.007231558	801367.161	56.04372046	11458720469	0.005357645
RP11-218I7.2	0.007087518	37.1689936	6.940095776	199.0655647	2.41E-05
RP11-218C14.5	0.008709809	0.011260674	0.000210589	0.602134537	0.027117285
RP11-216N14.8	0.037083135	0.00312039	1.48E-05	0.658766527	0.034617562
RP11-216B9.6	0.03670088	0.39866297	0.209611857	0.758221245	0.005050142
RP11-214K3.21	0.007671011	0.527279931	0.284939276	0.975731145	0.041529611
RP11-214D15.2	0.002326869	0.001138173	1.34E-05	0.096615298	0.002777953

RP11-213O5.4	0.001105481	0.272327726	0.100643224	0.736884085	0.010432927
RP11-213H15.1	0.007253763	0.452598575	0.282945315	0.72397548	0.000940924
RP11-213G2.3	0.001426533	0.610398789	0.437508353	0.851610442	0.003668708
RP11-212I21.4	0.014288224	0.139837694	0.02268436	0.862029181	0.034010045
RP11-212I21.2	0.005758642	0.677796091	0.517894946	0.887067047	0.004613464
RP11-211G23.2	0.03116426	1.126196765	1.002887433	1.264667511	0.044569384
RP11-210K20.4	0.042866649	0.004436441	2.32E-05	0.849385093	0.043295013
RP11-210K20.2	0.005553389	0.24511122	0.077990574	0.77034322	0.016104166
RP11-206P5.2	0.000186937	0.679552621	0.535272001	0.862723556	0.001510742
RP11-205K6.3	4.59E-05	7.35E-10	4.92E-15	0.000109672	0.00054015
RP11-204L24.2	0.041089722	0.296430373	0.105135657	0.835786529	0.021496675
RP11-204C16.4	0.015249678	1.350299011	1.057211316	1.724638576	0.016146203
RP11-203M5.7	0.019744496	0.705947776	0.5227325	0.953379144	0.023123543
RP11-203F8.1	0.003039212	78632.70131	2.577326112	2399037392	0.032381491
RP11-201O14.2	0.035144287	0.447689208	0.215980477	0.927980296	0.030699614
RP11-201M22.1	0.004902451	7.27E+44	352.7040348	1.50E+87	0.037713117
RP11-1E6.1	4.76E-05	2.557663542	1.202920485	5.438134003	0.014687441
RP11-19J5.1	0.004500291	0.026241783	0.001055365	0.652505082	0.026394211
RP11-19F9.1	0.030707462	1.12E-08	1.48E-16	0.853727807	0.048036566
RP11-196G11.5	0.041083522	0.643815894	0.517616767	0.800783382	7.63E-05
RP11-196B3.2	0.002410547	2.38E-05	3.37E-09	0.167462043	0.018510517
RP11-195M16.1	0.000936082	0.025059906	0.00173151	0.362688529	0.006854519
RP11-18O11.1	6.69E-07	3.07E+22	783522812.7	1.20E+36	0.001185106
RP11-18H21.1	0.001014148	0.266992334	0.124796653	0.571208482	0.00066171
RP11-182L21.5	0.002218822	0.003638294	2.79E-05	0.474208462	0.02380685
RP11-182J1.1	0.011763412	0.080931273	0.009688177	0.676068451	0.020264471
RP11-181B11.1	0.002782608	0.242366202	0.081117586	0.724150933	0.011151718
RP11-17M24.1	0.045527992	0.132094284	0.021429335	0.814252999	0.029153696
RP11-17J14.2	0.022465571	0.451342876	0.228207673	0.892653559	0.022235401
RP11-179A7.2	0.011783086	0.058165683	0.003635323	0.93065904	0.044350408
RP11-178C3.2	0.043014564	0.324995235	0.106170672	0.994831258	0.048948199
RP11-177G23.2	0.041471394	0.317407625	0.119187374	0.845287523	0.021659321

RP11-177F15.1	0.030324798	7.567222592	1.703261454	33.61953483	0.007816892
RP11-16E12.2	0.013699653	0.78843568	0.647695088	0.959758431	0.017818156
RP11-169K17.4	0.01242557	0.063482309	0.00472338	0.853203352	0.037551073
RP11-168K9.1	0.033467773	3.006298609	1.029425187	8.779493106	0.044114541
RP11-166P13.3	0.022969595	0.681331991	0.534179903	0.869020491	0.001996045
RP11-166N17.3	0.039496647	0.074485796	0.006069888	0.914042147	0.042333852
RP11-164O23.8	0.046292628	0.702768114	0.505716358	0.976600843	0.035641356
RP11-164N3.2	0.011770779	1.23E-05	6.72E-10	0.224197738	0.023907372
RP11-164J13.1	0.014919678	0.604145284	0.420186477	0.868641765	0.006526583
RP11-164C1.2	0.020633778	5331.381573	15.39538865	1846243.062	0.004022395
RP11-161H23.5	0.042434603	1880.604222	2.149427996	1645401.589	0.029156813
RP11-161D15.3	0.030880124	99119.14957	9.43245297	1041574853	0.014893261
RP11-161D15.2	0.000859951	5.987370167	1.692307077	21.18327224	0.005503051
RP11-161D15.1	0.000225546	3.959279986	1.818333237	8.621025941	0.000528282
RP11-15M15.2	0.031338825	6.991763188	1.328987822	36.78344652	0.021692327
RP11-15H20.6	8.39E-05	0.403234377	0.237807223	0.683738537	0.000748803
RP11-15B24.5	0.020787711	13.04256736	3.011649176	56.4835256	0.0005943
RP11-159K7.1	0.010441078	0.000399211	1.65E-07	0.965037594	0.048962568
RP11-153M7.3	0.00021996	0.560116356	0.330838341	0.948288918	0.030957748
RP11-152F13.10	0.015326903	111416.2197	99.36374016	124930623.5	0.001180615
RP11-14N7.2	0.000222027	1.953776376	1.493080942	2.556621025	1.05E-06
RP11-14C22.6	0.018676964	194.3777266	1.183209477	31932.38504	0.042908844
RP11-149I9.2	0.013491202	0.59302427	0.385460854	0.912356679	0.017441027
RP11-148O21.2	0.008188485	0.64540522	0.47046609	0.885394095	0.006636219
RP11-147L13.15	0.005675696	0.575093226	0.412115146	0.802523813	0.001138295
RP11-145P16.3	0.034408909	0.098802284	0.015448205	0.631911056	0.014494089
RP11-145H9.3	0.04477994	13.98668925	1.074121411	182.1278993	0.043950075
RP11-142A5.1	0.004832678	0.176622871	0.036251231	0.860540127	0.031884027
RP11-141O11.2	0.041111044	0.619874779	0.432861332	0.887685533	0.009048498
RP11-141M1.3	0.006551555	0.203519279	0.044609732	0.928499117	0.039805556
RP11-1415C14.4	0.028044234	117.3707333	1.440855655	9560.908464	0.033782871
RP11-13M3.1	2.83E-06	2.20173E+18	7762140.399	6.25E+29	0.001694814

RP11-139H15.6	0.005506374	0.492439902	0.295604265	0.820343568	0.006518501
RP11-1396O13.14	0.015889109	11662.93436	1.167950341	116463888.1	0.046260745
RP11-1379J22.5	0.0048526	0.462534848	0.260403722	0.821564625	0.008525713
RP11-136K7.3	0.007396106	516779.2022	46.08164866	5795381711	0.005691251
RP11-135M8__A.1	2.01E-06	3.2847E+14	39337362.26	2.74E+21	3.95E-05
RP11-133F8.2	0.012418027	0.159892014	0.032637673	0.783311236	0.023746912
RP11-132A1.3	0.034691818	1539.349865	24.60086402	96321.73919	0.000505976
RP11-131M11.3	0.016719345	0.103442414	0.020279613	0.527639904	0.006352442
RP11-131M11.2	0.019494778	0.434407625	0.216396432	0.872056821	0.019027087
RP11-131K17.1	0.003426469	2498338.063	7.606614123	8.20561E+11	0.023023419
RP11-131H24.4	0.041957462	0.354258171	0.148095383	0.847419072	0.019699902
RP11-1319K7.1	0.005812109	0.067699146	0.011665286	0.392890007	0.002688774
RP11-12J10.3	0.006632006	0.134604627	0.026011549	0.69655235	0.016796717
RP11-12A20.13	1.13E-07	231210053.6	3007.026526	1.77777E+13	0.000793028
RP11-128P17.2	0.029470265	26105.04685	4.335977425	157167209.3	0.022002135
RP11-128N14.4	0.01751921	0.39698684	0.166502222	0.946525217	0.037166361
RP11-128L5.1	0.01778508	0.45549855	0.234756413	0.883805157	0.020061464
RP11-127I20.7	0.013932907	0.064313347	0.006948098	0.595300582	0.015657336
RP11-1275H24.3	0.000739148	0.600076485	0.462556595	0.778481578	0.000120285
RP11-1275H24.1	0.003764813	0.627369439	0.469193246	0.838870584	0.001659194
RP11-126O1.6	4.86E-05	0.433186947	0.275596434	0.680890271	0.000288127
RP11-126K1.6	0.003997819	0.619780891	0.439053203	0.874901608	0.006532878
RP11-125N22.2	0.003161597	197.1707067	1.663235875	23373.8871	0.030099374
RP11-124O11.1	0.001555113	0.006930037	9.88E-05	0.486019021	0.021866999
RP11-124N2.1	0.015562556	0.559834971	0.326046209	0.96126005	0.035447488
RP11-1246C19.1	0.007326221	1.407041069	1.134372497	1.745250854	0.001889095
RP11-121A8.1	0.003061077	0.530727905	0.282446399	0.99725863	0.049011232
RP11-120M18.2	0.009329588	0.129709001	0.019189317	0.876759955	0.036183426
RP11-11C20.1	0.012168242	46.86748928	1.171734621	1874.623751	0.040936678
RP11-1191J2.5	0.006967175	0.624467134	0.461349699	0.845257301	0.002301088
RP11-118B22.4	0.04095119	0.522296141	0.303302676	0.899409338	0.019166382
RP11-117D22.2	0.002869841	0.225296452	0.087071861	0.582949424	0.002122479

RP11-114N19.3	0.004435055	0.444602103	0.23085663	0.856250174	0.015347328
RP11-114M1.1	0.000552683	0.631032752	0.497528492	0.800360866	0.000146963
RP11-113K21.2	0.018896286	0.212453404	0.08427554	0.535581841	0.001025179
RP11-1137G4.3	0.014513606	0.029886306	0.002599132	0.343649848	0.004844812
RP11-1136G4.2	0.004993355	0.669769778	0.511837676	0.876433244	0.003486564
RP11-111E14.1	0.009558625	0.422063867	0.210410245	0.846621836	0.015150044
RP11-1114A5.4	0.029235151	0.576091222	0.358452332	0.925872331	0.022719589
RP11-10A14.5	0.000264613	1.346602848	1.18891564	1.525204288	2.82E-06
RP11-109E24.2	0.036476251	0.393860649	0.175332503	0.884754442	0.02403952
RP11-1094M14.8	0.017178304	0.745219975	0.584049757	0.950865581	0.018021387
RP11-1094M14.5	0.021699591	0.667809062	0.470380979	0.948101566	0.023944926
RP11-1094M14.11	0.01227031	0.785721295	0.634091903	0.973609582	0.02749297
RP11-1090M7.3	0.022131907	0.476897245	0.227997207	0.997516528	0.049233032
RP11-1084J3.3	0.015917917	0.121837594	0.015519002	0.956530542	0.045259403
RP11-1084A12.2	0.016852393	0.019655588	0.000993769	0.388764543	0.009868817
RP11-1082A3.1	4.11E-05	8.48923E+17	1450448420	4.97E+26	6.12E-05
RP11-1081L13.4	0.025048979	0.552881337	0.313666825	0.974530133	0.040444855
RP11-107N15.1	0.048400724	0.303071866	0.12729974	0.721545511	0.006988746
RP11-1079J22.1	0.031949615	25.00269609	3.912277751	159.7879423	0.000670472
RP11-1070A24.2	0.018852845	2.38293443	1.058015352	5.367007662	0.036073553
RP11-106M3.3	0.036380994	0.393190727	0.195780482	0.78965455	0.008696539
RP11-1058N17.1	0.014148709	0.093426994	0.017822899	0.489740944	0.00503912
RP11-1057N3.2	0.00576612	141.0214644	10.0391412	1980.951659	0.000241831
RP11-1046B16.3	0.000422052	0.139598219	0.041634892	0.468060843	0.001423599
RP11-103H7.3	0.037640519	3.754167755	1.584661012	8.893874099	0.002646004
RP11-102N12.3	0.045683221	0.517909773	0.298403592	0.89888507	0.019340196
RP11-102K13.5	4.30E-05	2.050072655	1.630830947	2.577089857	7.75E-10
RP11-102G14.1	0.01901855	1.446408474	1.009407232	2.072600043	0.044327524
RP11-1017G21.5	0.020177512	0.746712863	0.567141082	0.983141792	0.037424102
RP11-100N20.1	0.000122057	6.290024126	3.054478061	12.95291789	6.05E-07
RP11-100F15.2	0.024726172	1.33929E+14	10.28541645	1.74E+27	0.034751781
RP11-1008C21.2	0.019815684	0.50438654	0.303078428	0.83940577	0.008448692

RP1-99E18.2	0.018237029	2.557028249	1.037646223	6.30117792	0.041323108
RP1-78O14.1	0.003866661	0.726206423	0.552590607	0.954369766	0.02173295
RP1-77H15.1	0.008955488	0.044941787	0.002165988	0.932491016	0.044948178
RP1-66C13.4	0.037756677	0.00500927	2.92E-05	0.860193036	0.043661701
RP1-47M23.3	0.007327388	0.359685791	0.169166061	0.764774374	0.007890051
RP1-39G22.7	0.030135085	0.671823616	0.522831635	0.863274026	0.001875826
RP1-315G1.3	0.011390095	0.673043722	0.47035798	0.963070408	0.030327194
RP1-308E4.1	0.000169089	0.129280317	0.027740079	0.602500096	0.009182689
RP1-301L19.1	0.032812187	0.000226991	8.63E-08	0.597261071	0.036776559
RP1-290I10.3	0.037521969	40.17651971	2.543753781	634.5554151	0.00871444
RP1-253P7.4	0.046611036	0.439525782	0.238313723	0.810624377	0.008482816
RP1-240B8.3	0.013890936	0.42600198	0.225791804	0.803739036	0.008426206
RP1-232P20.1	0.039653044	0.620342059	0.421377127	0.913253819	0.015527758
RP1-224A6.3	0.020175747	0.349369186	0.143955549	0.847892486	0.020087227
RP1-223B1.1	0.002866195	0.635581262	0.411175965	0.98245903	0.041389771
RP1-20C7.6	0.023961886	0.650099519	0.46395081	0.910935764	0.012351778
RP1-207H1.3	0.025514437	0.091755454	0.008532419	0.986714743	0.048723886
RP1-18D14.7	0.00600101	0.029990569	0.001670682	0.538363518	0.017300701
RP1-170O19.24	0.004566087	6.641559535	1.216974683	36.24587567	0.028759782
RP1-170O19.23	0.028892757	481.0094946	4.418593546	52362.84612	0.009854763
RP1-163G9.2	0.003237262	0.696144895	0.528951665	0.916185253	0.00974861
RP1-153P14.5	0.03798454	0.017092904	0.000630516	0.463378542	0.015656036
RP1-153G14.4	0.033764189	0.137071058	0.027417178	0.685281136	0.015510646
RP1-151F17.1	0.006162617	0.61256081	0.443826572	0.845444528	0.002871014
RP1-13D10.5	5.04E-05	14759631582	84566.51845	2.57604E+15	0.000143377
RP1-136J15.5	0.039398964	8.95E-10	1.70E-18	0.472339723	0.04203637
RP1-130G2.1	0.038757535	0.329978	0.137478301	0.792019391	0.013067666
RP1-127H14.3	0.001630508	0.714223007	0.541255815	0.942464707	0.01736967
RORA	0.011606109	0.620457045	0.43999247	0.874939849	0.006492207
ROGDI	0.047967772	0.691881052	0.535376147	0.894136567	0.004875099
RNY1P9	0.036563386	0.666414863	0.450866608	0.985011446	0.041778881
RNY1P10	0.029366819	0.088888872	0.009789057	0.807149411	0.0315311

RNVU1-15	0.011229172	0.656256216	0.445870345	0.965913579	0.032693892
RNU7-99P	7.43E-05	18.79924648	4.520045362	78.18763751	5.47E-05
RNU7-57P	0	152167642.7	22612.60032	1.02399E+12	2.80E-05
RNU7-49P	0.003618348	0.806189866	0.656169216	0.990509895	0.0402931
RNU7-14P	0.041869704	38.73861209	1.702991991	881.2020692	0.021794546
RNU7-123P	0.039392365	0.629291109	0.457325381	0.865920231	0.004456067
RNU6ATAC4P	0.034243253	0.231258036	0.078922544	0.677629949	0.007597926
RNU6ATAC24P	0.021529947	0.583253904	0.397341819	0.856152312	0.00590479
RNU6-992P	0.019907016	34.29839174	1.692128648	695.2069971	0.021303487
RNU6-984P	0.04065858	642.5217465	4.348790032	94930.81791	0.011191176
RNU6-955P	0.020739949	0.277667554	0.100700107	0.765632465	0.013285972
RNU6-926P	0.026550649	22.61188943	1.844142015	277.2549725	0.014746837
RNU6-877P	0.016805129	0.218787124	0.062725479	0.763131767	0.017123186
RNU6-826P	0.044629967	7.105848665	1.657037511	30.47190237	0.008294168
RNU6-795P	0.020966871	0.383910518	0.169435339	0.869873348	0.021789572
RNU6-684P	0.028618265	388.5305673	2.596648041	58134.94911	0.019626996
RNU6-646P	0.006999528	0.246106258	0.093318449	0.649049475	0.004602949
RNU6-627P	0.002960166	9516.020309	5.748052032	15753970.57	0.015417289
RNU6-606P	0.031478235	0.338537502	0.122610258	0.934731259	0.036598131
RNU6-5P	0.002116501	0.490096549	0.295235195	0.813570439	0.005818551
RNU6-584P	0.019670178	0.613786505	0.419951615	0.897088762	0.011707835
RNU6-561P	1.13E-07	19808.96499	61.21135973	6410494.652	0.000793028
RNU6-546P	0.021694949	364.7337454	1.625486436	81840.55064	0.03269119
RNU6-491P	4.77E-05	11172.32617	29.32747905	4256106.427	0.002110381
RNU6-445P	0.021689594	37.75944183	1.590292457	896.5492108	0.02463744
RNU6-439P	0.010632934	227.8447134	1.555886201	33365.68794	0.03286673
RNU6-435P	0.004743392	8.808677298	1.880835996	41.25441873	0.005747345
RNU6-38P	0.00227631	58026.16941	25.95188346	129741501.9	0.00531201
RNU6-268P	0.021145402	0.229567479	0.064571726	0.816165695	0.02297484
RNU6-256P	0	2.68554E+12	4114370.199	1.75291E+18	2.80E-05
RNU6-1335P	5.71E-05	2149096.06	104.1128852	44361597202	0.00402227
RNU6-1319P	0.013555235	17.11634085	1.182454547	247.7635396	0.03726253

RNU6-1299P	0.004687717	29.82152204	3.750710395	237.1079298	0.001328945
RNU6-127P	1.43E-06	514850.8747	159.237196	1664632573	0.001424204
RNU6-1279P	0.041668352	3.649088401	1.082947037	12.2959348	0.036749983
RNU6-1191P	0.023005636	1.507441024	1.070346623	2.123030419	0.018820165
RNU6-1176P	0.047512807	0.268071247	0.083788444	0.857662347	0.026504611
RNU6-1117P	0.003244106	3078.680852	3.78815945	2502079.417	0.018795634
RNU6-1095P	0.025705947	0.666387868	0.488366431	0.909302445	0.010481394
RNU6-1066P	0.040139181	21.01779145	1.197467474	368.9015086	0.037229436
RNU6-1037P	0.003595066	34718.58453	10.60289789	113684025.2	0.011350512
RNU4-72P	0.002389256	16.25840774	3.057068947	86.46707899	0.001073422
RNU4-34P	0.029577102	0.119144475	0.01782027	0.796587613	0.028195217
RNU2-68P	0.021661598	0.268447678	0.084690319	0.850913737	0.025466965
RNU1-61P	0.019419694	0.581206598	0.366151974	0.922570773	0.021345498
RNU1-57P	0.005533126	3676.740118	2.91726456	4633936.216	0.024202642
RNMTL1P2	0.001399447	8.52E+28	1.29558E+13	5.60E+44	0.000337445
RNF4	0.037676484	1.530577625	1.034004608	2.265626137	0.033414328
RNF185	0.011736466	0.649965773	0.424436729	0.995332111	0.047537176
RNF180	0.008071118	0.65003131	0.49017293	0.862023745	0.002781362
RNF175	0.001392505	0.558997225	0.357127026	0.874976897	0.01095285
RNF166	0.028621911	0.676811407	0.511471077	0.89560036	0.006304737
RNF146	0.003424476	0.64548004	0.459448663	0.906835769	0.011610671
RNF144B	0.013040201	0.811752017	0.672082827	0.980446621	0.03039173
RNF138P1	0.014265498	0.264996016	0.079437605	0.884000579	0.030729848
RNF130	0.016814914	0.620321529	0.463190729	0.830756697	0.001354817
RNF125	0.045896406	0.705816095	0.510478218	0.975901306	0.035072033
RNF103-CHMP3	0.003250884	2.674483545	1.170296331	6.11200945	0.01965424
RND3	0.031056314	1.2203881	1.063527699	1.400383944	0.004548109
RNASE1	0.001974356	0.850188854	0.773462434	0.93452643	0.000770455
RNA5SP86	0.013684832	1222.613026	1.880502569	794884.6418	0.031471154
RNA5SP520	0.000220256	13633.94257	14.64641089	12691463.55	0.006342246
RNA5SP516	0.005533126	442.7277816	2.213530514	88549.8923	0.024202642
RNA5SP487	0.002795716	1183.241873	3.441275721	406843.6954	0.017562591

RNA5SP472	5.25E-06	1797036.361	194.2041492	16628582330	0.001996738
RNA5SP47	0.021182708	0.115551645	0.019141456	0.697553153	0.018641894
RNA5SP443	0.009141666	11.15751267	1.364646104	91.22518188	0.024451532
RNA5SP323	0.006952329	1.5760271	1.028444832	2.415162527	0.036730416
RNA5SP270	0.004285649	815646.2756	7.510208931	88583267535	0.021404652
RNA5SP226	0.000152776	27.63359547	5.486952413	139.1693496	5.73E-05
RNA5SP220	5.50E-10	2065761757	18835.39959	2.26561E+14	0.000291895
RNA5SP177	1.11E-16	130814891.9	16394.24824	1.04381E+12	4.56E-05
RNA5SP133	0.001171242	1518.163328	5.891829959	391189.1395	0.009706807
RN7SL42P	0.023899072	0.036813081	0.002100085	0.64530876	0.023837507
RN7SL403P	0.000168313	0.449200626	0.234550349	0.8602895	0.015784097
RN7SL400P	5.50E-10	3.09E+25	4.9883E+11	1.91E+39	0.000291895
RN7SL338P	0.006278221	0.152396758	0.039118626	0.593701118	0.006699748
RN7SL201P	0.011306264	124223.6246	24.93450713	618881649.8	0.00692568
RN7SL145P	0.002717321	0.470244163	0.290387361	0.761498615	0.002156286
RN7SKP82	0.006237432	13018902.54	7.32489404	2.31392E+13	0.025669834
RN7SKP75	0.018907992	0.02134042	0.000671114	0.678592941	0.029284306
RN7SKP51	0.030848177	0.753862533	0.595374652	0.954539661	0.018959384
RN7SKP285	0.019346647	0.03865057	0.001855198	0.805232841	0.0357478
RN7SKP273	0.015143021	0.082751383	0.008121588	0.843159167	0.035377847
RN7SKP223	0.001713882	15.31937433	2.28594551	102.6635276	0.00492669
RN7SKP218	0.037685724	3536811.992	27.17009966	4.60397E+11	0.012089192
RN7SKP212	4.48E-13	23227205100	132760.5549	4.06373E+15	0.000106572
RN7SKP196	0.004883058	1.47135E+12	49.49648816	4.37E+22	0.022780858
RN7SKP142	4.45E-06	6999.234623	9.440566615	5189231.463	0.008644765
RN7SKP129	6.69E-07	6.16E+20	167060554	2.27E+33	0.001185106
RN7SKP104	0.038099202	3483810.326	128.6696282	94326334498	0.003819263
RMDN2	0.000785398	0.628326026	0.47802669	0.825881908	0.000864019
RIPK2	0.00264483	1.442090592	1.177269517	1.766481886	0.000405551
RIMS2	0.001468949	1.410618466	1.034305484	1.923845991	0.029779042
RIMKLA	0.008884589	0.755851685	0.61328398	0.931561542	0.008671731
RILPL2	0.006950855	0.674597421	0.512571265	0.887840796	0.00497222

RIIAD1	0.011734107	0.674884523	0.472723033	0.963501009	0.030414473
RIC3	0.042337412	0.600685474	0.412277833	0.875193887	0.00795029
RIBC1	0.020455097	0.749738759	0.569558388	0.986919372	0.039989904
RHPN2	0.034174723	1.303731723	1.123147891	1.513350484	0.000489119
RHOV	0.000853386	1.2476257	1.148070188	1.355814221	1.84E-07
RHOT1P1	0.001071863	1.4238211	1.004641413	2.017900613	0.047034328
RHOQ	0.032656898	0.599506298	0.436963835	0.822511551	0.001519725
RHOH	0.007040639	0.742242139	0.592176893	0.93033585	0.00969406
RHOF	0.005810654	1.657767547	1.319881477	2.082151534	1.38E-05
RHOC	0.005294144	1.343790455	1.091543888	1.654329072	0.005340609
RHOBTB2	0.03027063	0.844631576	0.74087837	0.962914466	0.011566694
RHOA-IT1	0.048609649	0.574435597	0.336566226	0.980419987	0.042106933
RHNO1	0.007043527	1.49608479	1.10707892	2.02177971	0.008739792
RGS20	0.01029678	1.606566952	1.325284724	1.947549328	1.38E-06
RGS18	0.015136648	0.756041614	0.573145732	0.997301194	0.04780602
RGS13	0.002776865	0.273415864	0.122052807	0.612490906	0.001625761
RGS12	0.017285812	0.712085923	0.517863457	0.979150692	0.036651342
RGPD8	0.005654151	0.230999977	0.059177183	0.901715607	0.034957207
RGN	0.001642974	0.72605556	0.595278035	0.885563796	0.001581206
RGL4	0.033760421	0.360204458	0.174439425	0.743795453	0.005779485
RGL2	0.016127862	0.730287967	0.580123184	0.919322877	0.007447031
RGAG4	0.001017789	0.674814806	0.543463341	0.837913045	0.000369341
RFXAP	0.00021435	0.483801099	0.325513854	0.719058499	0.000329118
RFX3	0.006650669	0.653745533	0.461393991	0.926286927	0.016818907
RFTN1	0.003769824	0.804969971	0.689369592	0.939955377	0.006091113
RFESDP1	0.00508911	27.53818099	4.441442583	170.7443917	0.000368656
REV3L	0.048711296	0.663795469	0.487920591	0.903065853	0.009076384
REV1	0.02714726	0.602023755	0.432553044	0.837891691	0.002625035
REREP1Y	2.89E-07	7.22399E+13	417912.973	1.25E+22	0.000975953
RENBP	0.046470018	0.816616961	0.695982133	0.958161465	0.012991193
RELA	0.044532103	1.744967578	1.128680147	2.697763272	0.01226212
RCSD1	0.001599957	0.749234871	0.61141246	0.918124717	0.005376353

RCOR3	0.047955669	0.704941568	0.50793502	0.978358637	0.036546269
RCOR1	0.02360241	1.450388374	1.088421484	1.93273145	0.011136718
RCN1	0.000668187	1.41179792	1.109692514	1.796149241	0.004997389
RCCD1	0.043012195	1.531402413	1.169368611	2.00552104	0.001955329
RCBTB2	0.003784927	0.608814655	0.46330946	0.800016655	0.000369241
RCBTB1	0.003299434	0.640201436	0.470680831	0.870776652	0.004488494
RCAN2	0.019730339	0.792957598	0.680517732	0.923975559	0.002945001
RBSN	0.000335963	0.633232305	0.453963248	0.883294306	0.007128883
RBPMS-AS1	0.00682643	0.756065809	0.615656741	0.928497114	0.007636208
RBPMS	0.002313031	0.818425402	0.690447145	0.970125149	0.020912592
RBP5	0.002053304	0.736180305	0.595254588	0.910469994	0.004726285
RBMY3AP	0.00580113	468847.2339	34.93931522	6291414909	0.007085986
RBMY1E	0.011983694	7962.382441	14.97365257	4234072.738	0.005030098
RBMY1A3P	0.011707807	243.0754158	3.724680988	15863.2801	0.009972418
RBM42	0.030059527	1.35963868	1.075161502	1.719385726	0.010316407
RBL2	0.008126397	0.728048419	0.573071745	0.924935672	0.009351784
RBCK1	0.011545032	1.353675353	1.028332308	1.781950199	0.030837134
RASSF2	0.001849044	0.788485363	0.664620078	0.93543543	0.006422079
RASSF1-AS1	0.009610981	0.42141432	0.212942006	0.833983078	0.013092895
RASGRP4	0.02222436	0.616617998	0.419525108	0.906305124	0.01386932
RASGRP2	0.004290593	0.655902627	0.501605446	0.85766265	0.002055809
RASGRF1	0.014877584	0.816463495	0.685342347	0.97267102	0.023196045
RASAL3	0.024909841	0.782964891	0.644663414	0.950936578	0.013614386
RARS	0.000471457	1.443401428	1.055421153	1.974005994	0.021581101
RARRES2P3	2.05E-14	8.28E+22	9.49467E+15	7.23E+29	9.69E-11
RAPGEF5	0.002658281	0.81719961	0.672158634	0.993538087	0.042867946
RAPGEF3	0.031579649	0.711178345	0.525206944	0.963000669	0.027543353
RAP1BP2	0.000186191	15.76413159	4.145618321	59.94469958	5.20E-05
RAP1AP	0.00482877	0.561479431	0.367134076	0.858703052	0.007751138
RANGAP1	0.004865678	1.485760563	1.141621892	1.933638856	0.003227052
RANBP1	0.031052613	1.318835472	1.059839651	1.641122788	0.01310252
RALGPS2	0.019528207	1.284763307	1.018645615	1.620403337	0.03434768

RALGDS	0.017363477	0.717446354	0.552626848	0.931422845	0.012651979
RAET1L	0.002417644	1.456163922	1.112693444	1.905658185	0.006182577
RAD51AP1	0.007851306	1.20215542	1.029025882	1.404413319	0.020308611
RAD51	0.018434057	1.400749598	1.157339712	1.695353071	0.000539485
RAD23A	0.000494587	1.49729313	1.060190207	2.114608022	0.02191663
RACGAP1	0.005580173	1.2839374	1.09504127	1.505418371	0.002082826
RAC1P2	0.003044859	1.436413707	1.162623065	1.774680377	0.000789381
RAC1	0.025452082	1.571196284	1.17604232	2.099123238	0.002234985
RAB9B	0.000104468	0.492431215	0.271725803	0.892401452	0.019531228
RAB44	0.016840757	0.130918093	0.038431604	0.445975321	0.001149199
RAB3B	0.033540957	1.221080454	1.022597416	1.458088444	0.027323771
RAB39B	0.010032717	0.649928395	0.451026707	0.936545245	0.020795953
RAB35	0.002672655	1.719246905	1.178044891	2.509080888	0.00496177
RAB30-AS1	0.025697978	0.763315781	0.585565717	0.99502236	0.045840612
RAB30	0.034331614	0.642338293	0.454472011	0.907863352	0.012157164
RAB27B	0.013126969	1.206541758	1.057428529	1.376682181	0.005277269
RAB11FIP4	0.019688825	0.690727792	0.514877969	0.926636818	0.013578301
RAB11FIP1P1	0.029307961	0.675833357	0.5034216	0.907292669	0.009122966
RAB11A	0.029600786	1.416798868	1.024155949	1.959974001	0.035368281
RAB10	0.034944717	1.510303835	1.152627265	1.978972513	0.002789224
R3HDM1	0.001342383	1.598733616	1.164350702	2.195171242	0.003724183
PYHIN1	0.015370591	0.70057548	0.515428482	0.952229108	0.023052187
PYGL	0.030210385	1.230227326	1.052555746	1.437889898	0.00922522
PXMP4	0.000563563	0.750500724	0.645181014	0.873012884	0.000199023
PXK	0.002186531	0.560721912	0.40379033	0.778644358	0.000553281
PWP1	0.041598215	1.468080363	1.014750263	2.123931405	0.041582678
PWAR6	0.017553015	0.712383682	0.516948309	0.981704557	0.038188882
PVR	0.00426489	1.429930251	1.167020472	1.752069114	0.00056068
PUS10	0.013594473	0.567606363	0.365056203	0.882540771	0.01190951
PTX3	0.021730507	1.204691412	1.001941768	1.448468806	0.047636619
PTTG1IP	0.001024457	1.387302982	1.06666427	1.804325521	0.014637345
PTTG1	0.00101369	1.300257563	1.122952342	1.505557864	0.000447615

PTPRO	0.045206757	0.696900665	0.516521056	0.940272484	0.018130054
PTPRH	0.000361343	1.285293235	1.144360923	1.443581888	2.28E-05
PTPRC	0.003422284	0.828289906	0.723899366	0.947734174	0.006125354
PTPN7	0.045858515	0.806825682	0.662661847	0.98235274	0.032577933
PTPN6	0.046108677	0.644096667	0.477389687	0.869018598	0.003993816
PTPLAD2	0.002568798	0.721847875	0.552426345	0.943228647	0.016930702
PTP4A1P4	0.013369015	63.72327716	1.577174615	2574.639494	0.027708159
PTK7	0.023946925	0.782969631	0.662006625	0.926035209	0.004271005
PTK2B	0.048794168	0.790313446	0.639468535	0.976741323	0.029424904
PTGFRN	0.045667395	1.311929691	1.071999292	1.605560308	0.008422598
PTGES3	0.000609386	1.561016495	1.20705963	2.018767288	0.000688032
PTGDS	0.016458927	0.844159954	0.760570386	0.936936332	0.00145074
PTGDR2	5.88E-05	0.307964502	0.128584732	0.73758473	0.008217463
PTDSS1	0.016606423	1.440405528	1.07195386	1.935501295	0.015481485
PTCSC3	0.023029385	0.799709404	0.696086219	0.918758502	0.001595863
PTCRA	0.046993275	0.611606805	0.390128693	0.958819206	0.032090512
PTCH1	0.004202599	0.640335598	0.449291022	0.912614894	0.013671516
PTBP1	0.001782512	1.943581584	1.310603814	2.882266427	0.000948538
PSTPIP1	0.001047455	0.745607296	0.589258229	0.943440775	0.014490901
PSME3	0.005069902	1.531294299	1.124645269	2.084979411	0.00681179
PSMD8P1	0.00186835	0.21449776	0.04850671	0.9485139	0.042391692
PSMD6-AS2	0.010181462	0.526383134	0.283651835	0.976828525	0.041923135
PSMD2	0.013894753	1.645442474	1.284403263	2.107967968	8.14E-05
PSMD11	0.048432372	1.619519454	1.23451743	2.124589898	0.000499262
PSMD10P2	0.000435817	1.512647158	1.228949372	1.861835382	9.41E-05
PSMD10P1	0.000680517	0.388135045	0.210893907	0.714334595	0.002359102
PSMD1	0.004634126	1.42760195	1.111511565	1.833581756	0.005305274
PSMC6	0.000122003	1.393761162	1.057082475	1.837671349	0.018598891
PSMC4	0.012532891	1.42585832	1.097662239	1.852183557	0.007857508
PSMC1	0.012683063	1.460578658	1.101764605	1.936248458	0.008445412
PSMB7	0.002742906	1.655386498	1.209952673	2.264803012	0.001623605
PSMB6	0.009610775	1.361219884	1.038741282	1.783812395	0.025384425

PSMB5	0.049417321	1.377838443	1.074215285	1.767279615	0.011614504
PSMA5	0.005712038	1.374529693	1.0441463	1.809451298	0.023332115
PSMA2	0.003603809	1.440893245	1.039727941	1.996842886	0.028237287
PSEN1	0.042803167	1.574897514	1.035498553	2.39527344	0.033752401
PRYP5	0.004286602	181498.3463	6.3724318	5169400119	0.020675951
PRUNEP1	0.014972701	4325116.493	79.03131103	2.36699E+11	0.00605124
PRRT3	0.014744785	0.685764014	0.532525013	0.883098955	0.003462213
PRRG1	0.017244661	1.413132455	1.093439316	1.826295529	0.008227522
PRR15	0.036954249	1.132400412	1.034048352	1.240107091	0.007313584
PRR11	0.0042483	1.323922873	1.138220307	1.539923127	0.000273767
PRKG1-AS1	4.98E-06	3.906361647	1.845326616	8.269355237	0.000369282
PRKCZ	0.030208281	0.757770661	0.624098172	0.920073796	0.00509077
PRKCE	0.035261532	0.556277152	0.386567894	0.800491385	0.001586854
PRKCD	0.000103543	0.597524812	0.471599404	0.757074537	2.00E-05
PRKCB	0.000859776	0.713159816	0.5642391	0.901385463	0.004673366
PRKAR2A-AS1	0.027499401	0.546374377	0.307871079	0.969642751	0.038894996
PRKAG2-AS1	0.009890174	0.735388866	0.596562218	0.906522015	0.00398492
PRINS	0.034609861	0.64268599	0.455497024	0.906801275	0.011838035
PRICKLE4	0.024818827	0.819677144	0.678598374	0.99008581	0.039079006
PRICKLE2-AS1	0.022819421	0.000243917	8.86E-08	0.671536124	0.039542414
PRICKLE2	0.024397255	0.665761346	0.495156653	0.895147359	0.007075584
PREX2	0.034948818	0.677242727	0.477109876	0.961325124	0.029208564
PRELP	0.048255528	0.867175138	0.768919276	0.977986563	0.020192274
PRDM2	0.000297589	0.608401825	0.445954276	0.830024064	0.001715643
PRDM16	0.000164238	0.738962821	0.615706429	0.886893534	0.001157328
PRCD	0.011025013	0.190925213	0.048997779	0.743961001	0.017024665
PRC1	6.63E-05	1.383039781	1.188043079	1.610041814	2.89E-05
PPT2	0.008937589	1.45286832	1.151403005	1.833264587	0.001643188
PPP4R4	0.038059672	0.633725059	0.406715327	0.98744115	0.043819148
PPP4R1L	0.008486459	0.661947249	0.488664694	0.896676525	0.007716283
PPP3CC	0.028672276	0.611672708	0.433243602	0.863586906	0.005215714
PPP2R5A	0.044755664	0.768623015	0.597505305	0.988746601	0.040555901

PPP2R1A	0.008253576	1.789921107	1.209023841	2.649920918	0.00363578
PPP2CA	0.016100366	1.743006107	1.172759253	2.590531926	0.005992416
PPP1R3G	0.000222813	1.741461004	1.344646155	2.255378798	2.62E-05
PPP1R32	0.045877849	0.720931169	0.549680428	0.945534393	0.018044549
PPP1R16B	0.016315428	0.797299169	0.661318333	0.9612405	0.017582256
PPP1R13L	0.001136197	1.322017383	1.10403727	1.58303529	0.002393022
PPP1R13B	0.000346985	0.657760508	0.536458519	0.806490849	5.63E-05
PPOX	0.008375562	0.667553546	0.511510916	0.871198881	0.002930044
PPM1M	1.94E-05	0.605219646	0.477388428	0.767280476	3.35E-05
PPM1G	0.001115905	1.50853687	1.109054012	2.05191403	0.008807902
PPIL1P1	0.017428318	52.04418726	1.513068958	1790.13482	0.028568485
PPIEL	0.002116859	0.674701777	0.486369323	0.935960528	0.018459141
PPID	0.040523691	1.571043303	1.119964901	2.20379858	0.008894485
PPIAP22	0.00302567	1.38310517	1.102917179	1.734472857	0.00498342
PPIAL4B	0.007264673	3.271927422	1.068803274	10.01635129	0.037845247
PPIA	0.000467789	1.456800245	1.127931036	1.881557371	0.00394961
PPFIBP2	0.021126313	0.673105467	0.520390689	0.870636197	0.002568747
PP2672	2.77E-05	2.11E-07	1.05E-11	0.004207044	0.002343879
POU6F1	0.009725475	0.5893922	0.405169031	0.857378375	0.005698154
POU2AF1	0.011525155	0.829288577	0.719285111	0.95611536	0.009936635
POMT1	0.046224902	0.69792418	0.491663288	0.990714932	0.044203587
POM121L14P	0.048656392	0.000155614	3.33E-08	0.726417004	0.041939996
POM121L13P	0.020842941	367887.8199	14.39115682	9404487051	0.013325793
POLR3G	0.014336615	1.512264976	1.165276791	1.962576939	0.001869982
POLR2J4	0.019021567	0.69288113	0.511415043	0.938737073	0.017884937
POLQ	0.009902494	1.332298783	1.016379818	1.746414104	0.037744668
POLE2	0.005909857	1.252388863	1.029182609	1.524003467	0.024628154
POLD2	0.003499067	1.416466661	1.108337866	1.810258281	0.005405488
POC1A	0.001423275	1.292417852	1.063173902	1.571091897	0.010027678
PNRC2	0.00502463	0.584935868	0.410600615	0.833291421	0.002977737
PNPLA7	0.000261023	0.713511437	0.556808068	0.91431608	0.007630953
PNMA2	0.000486639	0.806225273	0.706310515	0.920273984	0.001419106

PNLIPP1	0.008656591	216674.3308	4.459019708	10528719026	0.025649244
PNISR	0.035436638	0.781027854	0.617561077	0.987763853	0.039139298
PMEPA1	0.000754934	1.268411512	1.100652636	1.4617398	0.001019958
PMAIP1	0.018551651	1.139919991	1.006914004	1.290495099	0.038563712
PLOD2	0.037826572	1.200844484	1.070346461	1.347252994	0.001819781
PLK4	0.010103475	1.345290201	1.100099648	1.645128901	0.003861536
PLK1	8.54E-05	1.404974984	1.215060666	1.624572961	4.46E-06
PLIN5	0.000474374	0.670934706	0.507951565	0.886213196	0.00494275
PLIN3	0.006538594	1.511021793	1.216180203	1.877342563	0.000193695
PLEKHD1	0.002991325	0.469725592	0.227587885	0.969481005	0.040973404
PLEKHB1	9.52E-05	0.698615755	0.603945137	0.80812634	1.38E-06
PLEKHA3P1	0.001298826	0.223285631	0.085071556	0.586053379	0.002324556
PLEK2	0.01061803	1.413709071	1.222062577	1.635409983	3.19E-06
PLEK	0.024392104	0.866134522	0.758616665	0.98889076	0.033573342
PLD4	0.003834387	0.661725665	0.498112712	0.879079866	0.004381271
PLCG1-AS1	0.015218373	0.28434752	0.08665546	0.933045789	0.038054045
PLCG1	0.006825122	0.781432046	0.624166109	0.978322971	0.031467269
PLCD3	0.008443954	1.51561845	1.285506091	1.786922134	7.45E-07
PLCB2	0.02674174	0.808637791	0.67524432	0.968382936	0.020929398
PLAUR	0.00182843	1.224061901	1.064176343	1.407969222	0.004641323
PLA2G4F	0.028430204	0.791795252	0.67057427	0.934929582	0.005894686
PLA2G3	0.017569023	0.735870457	0.581953471	0.930495919	0.010417973
PLA2G1B	0.024528902	0.874904758	0.794501698	0.963444555	0.006585283
PLA2G12B	0.038154289	0.875168473	0.778430646	0.983928189	0.025676698
PKP2	9.14E-05	1.377565091	1.218154486	1.557836548	3.31E-07
PKNOX2	0.017067077	0.666305459	0.459179461	0.966861549	0.032567646
PKMYT1	0.001025895	1.350321902	1.131128188	1.611991691	0.000889345
PKMP3	0.036588361	0.58476563	0.352918909	0.968921851	0.03729659
PKM	0.032812135	1.675783989	1.28511866	2.185208312	0.000137694
PKD2L2	0.00546899	0.035108841	0.001325392	0.930011994	0.045138236
PITX3	2.86E-07	10.44718305	5.612774875	19.4455748	1.34E-13
PITX1	0.025255914	1.12658911	1.014217705	1.251410833	0.026195967

PITPNC1	0.034643494	1.174633827	1.002315116	1.37657769	0.046752776
PINLYP	0.002933362	0.572847266	0.379681048	0.864288569	0.00793094
PIK3R1	0.001224051	0.740850366	0.578366037	0.948982528	0.017573195
PIK3IP1	0.007705053	0.755408741	0.620750152	0.919278657	0.005107216
PIK3CG	0.007983767	0.671089877	0.528106144	0.852786184	0.001103982
PIK3CD	0.000226691	0.768414865	0.630254624	0.936861679	0.009190376
PIGR	0.033746966	0.936841376	0.887638642	0.988771469	0.017778395
PIGFP3	0.014829762	7060888708	5.045061405	9.88217E+18	0.034807865
PIGA	0.038943313	0.789020842	0.626120062	0.994304331	0.044603392
PIFO	0.008116944	0.834233492	0.723594156	0.961789857	0.012537833
PHYKPL	0.044507125	0.744840219	0.569213674	0.974654997	0.031787635
PHF5A	0.00043213	1.276369042	1.003127574	1.624038629	0.047103255
PHF11	0.015562283	0.626703754	0.447151583	0.878354478	0.00666711
PHF1	0.005509507	0.579661101	0.422570079	0.795150932	0.000721423
PHACTR1	0.008377535	0.697098967	0.540584221	0.898929252	0.005414876
PGS1	0.012198398	0.592391738	0.438937185	0.799494742	0.000619675
PGPEP1	0.008388266	0.579023332	0.435016278	0.770702238	0.000180315
PGM5	0.000961084	0.727303361	0.582771989	0.907679485	0.004849119
PGM2	0.011504405	1.463110711	1.182558153	1.810222142	0.00045877
PGAP3	0.004449906	0.754548827	0.608766388	0.935242062	0.010135232
PGAM4	0.011117787	4.778295186	1.898671388	12.02530624	0.000895218
PGAM1P1	0.000982297	1060818.526	406.2034082	2770375437	0.000547517
PFN1	0.029423732	1.573993814	1.126598418	2.19905912	0.007846603
PFKP	0.001171613	1.282811473	1.123308769	1.464962547	0.000236541
PFDN1	0.006277718	1.44566563	1.029956535	2.029162437	0.033122892
PES1	0.007644	1.424423534	1.035696331	1.959051457	0.029580015
PERP	0.000459792	1.352095568	1.149249115	1.590745123	0.000275495
PELO	0.013346214	1.405730868	1.040815219	1.89858799	0.026361837
PEF1	0.003593047	0.64501196	0.437361466	0.951250764	0.026960262
PECAM1	0.013642631	0.778943203	0.642291645	0.944668233	0.011138327
PEBP4	0.027245299	0.895951023	0.828103289	0.969357623	0.006246473
PEBP1	0.000694846	0.603658936	0.464865289	0.78389185	0.000152746

PDZRN3-AS1	0.003297545	1.97E-09	2.79E-15	0.001392107	0.003532652
PDZD9	0.026567046	0.031223536	0.003121461	0.31232467	0.003173685
PDLIM7	0.021325804	1.3306193	1.100744819	1.608499709	0.003158082
PDK2	0.037143259	0.630424174	0.473513952	0.839330368	0.001581
PDIK1L	0.001752681	0.588292962	0.437780576	0.790552683	0.000433563
PDGFB	0.001774823	1.242958935	1.042152214	1.482458027	0.015554073
PDE6B	0.003966749	0.745103225	0.5889743	0.942619765	0.014186109
PDE4B	0.000260251	0.798958845	0.641736699	0.994699596	0.044696645
PDCD5	0.012659623	1.269731873	1.027614585	1.568894654	0.026944586
PDCD4	0.036885372	0.769388724	0.601733775	0.983755662	0.036566904
PCSK9	0.030033246	1.166094902	1.039846564	1.307671119	0.008581461
PCP4L1	0.011964113	0.888057397	0.819086695	0.962835735	0.004000685
PCP2	0.022726085	0.694565897	0.552198585	0.873638214	0.001844128
PCMTD2	0.04995344	0.591930265	0.433919594	0.807480104	0.000934179
PCED1B-AS1	0.042771555	0.773060401	0.633641911	0.943154759	0.011188777
PCDHB8	0.013250124	1.242077063	1.024519683	1.505832884	0.027347296
PCDH7	0.004697663	1.295245062	1.13477863	1.478402682	0.000126275
PCDH15	0.015060184	0.0714725	0.006826744	0.748280364	0.02766734
PCBP1-AS1	0.044126904	0.33969616	0.172675933	0.668266151	0.001762958
PCAT29	0.000477785	0.57348687	0.351163613	0.936563975	0.026293382
PBXIP1	0.028432095	0.771162932	0.636850997	0.933801267	0.007780248
PBK	0.00264619	1.257299261	1.106847348	1.428201852	0.00042976
PAXBP1P1	0.000372143	1.52544E+17	838782.5258	2.77E+28	0.002779899
PAX5	0.007105977	0.717185429	0.546696851	0.940841234	0.016382379
PARVG	0.019452247	0.762687786	0.613574828	0.948038661	0.014658599
PARPBP	0.001782188	1.282294635	1.031971711	1.593337796	0.024833701
PARP15	0.000548311	0.679539095	0.54218971	0.851682304	0.000798
PARM1	0.031110884	0.855092201	0.770206186	0.94933368	0.003338856
PARD3B	0.018301759	0.682855595	0.506911825	0.9198676	0.012093118
PAQR4	0.014954258	1.212079298	1.012946378	1.450359324	0.035690256
PAPLN	0.047097843	0.775097634	0.639878519	0.938891251	0.009197717
PAPL	0.022632543	1.571616351	1.080959682	2.2849862	0.01790107

PAOX	0.004862405	0.546213145	0.383897621	0.777157198	0.000775974
PANX1	0.000325167	1.489050302	1.147473524	1.932306722	0.002747481
PAN3-AS1	0.003027873	0.44404724	0.264026001	0.746812626	0.002209062
PAK4	0.004637593	1.363758553	1.070103856	1.737997092	0.012154974
PAK2	0.010463805	1.518265221	1.125976203	2.047227354	0.006182568
PAIP2B	0.00887442	0.760356826	0.596521604	0.969189547	0.026916865
PAICSP4	0.023427062	2.554709706	1.116131258	5.847467879	0.026417932
PAICSP1	0.000440817	0.168225234	0.049149821	0.57578499	0.004521501
PAICS	0.012166354	1.461870099	1.186330761	1.801406703	0.000366017
PAFAH1B3	0.034888782	1.189487117	1.006484125	1.405764448	0.041771323
PAFAH1B2	0.000429904	1.662549078	1.186570956	2.329459879	0.003136434
PABPC1P6	0.046026308	5.15061E+18	20.45258646	1.30E+36	0.03506566
PABPC1P12	0.00128116	2.17464E+19	72689039.65	6.51E+30	0.000957865
PABPC1	0.032697925	1.319162476	1.062375382	1.63801766	0.01214992
PA2G4	0.011625021	1.596230438	1.183701458	2.152528912	0.002173376
P4HTM	0.048020561	0.693349339	0.522090422	0.92078553	0.011402031
P4HA1	0.044589788	1.307365087	1.078684643	1.584525638	0.00629393
P3H4	0.029485136	1.172174782	1.001707804	1.371651208	0.047563824
P2RY8	0.003078818	0.708596756	0.569797883	0.881206087	0.001955353
P2RY14	0.000654565	0.673765249	0.507597472	0.894329929	0.006277957
P2RY13	0.003250369	0.723920487	0.600130015	0.873245562	0.000734142
P2RY12	0.002510761	0.676308028	0.51411045	0.889677595	0.005181982
P2RX7	0.015951374	0.793271502	0.636921525	0.988001898	0.038664835
P2RX3	0.025354705	0.131725406	0.019054134	0.910646599	0.039893625
P2RX2	0.003998371	0.483750006	0.239160697	0.978480459	0.043332371
P2RX1	0.006164687	0.589423989	0.435608964	0.797551629	0.000612313
OTUD6B	0.001477085	1.378388618	1.064286704	1.785191127	0.015009008
OSGIN2	0.006534025	1.332948209	1.090189043	1.629764067	0.005081788
OSBPL2	0.004446078	0.678470636	0.493697539	0.932397607	0.016780203
ORMDL3	0.001392217	0.616727921	0.485462537	0.783486468	7.55E-05
ORC1	0.009018638	1.307014356	1.09724771	1.5568832	0.00270233
OR8T1P	0.003252785	0.13407019	0.02693109	0.667437359	0.014140819

OR8J2	7.49E-05	1.50407E+12	73960.34805	3.06E+19	0.001091732
OR7E85P	0.034925743	36.53522694	4.868306469	274.1862733	0.000466875
OR7E4P	0.002337325	18508.85793	92.85231752	3689491.344	0.000275686
OR7E47P	0.010496863	0.680807585	0.54201128	0.855146349	0.000949144
OR6Q1	1.11E-16	4.28E+70	4.74E+36	3.85E+104	4.56E-05
OR6K4P	0.032736156	7.37E-05	5.88E-09	0.923370459	0.048095495
OR6K3	0.049625301	0.007044233	5.29E-05	0.938267339	0.047089509
OR6C71P	0.000968958	8.99052E+14	7358204.857	1.10E+23	0.000289867
OR5W2	0.017406493	66804.50636	4.547898812	981297573.8	0.023245949
OR5T3	0.02677595	2427737562	27.62257831	2.13373E+17	0.02058235
OR5K4	0.023991412	2.21E+23	8278.577592	5.89E+42	0.018509599
OR5D2P	0.004033771	1.8725E+13	376729.883	9.31E+20	0.000724973
OR5BP1P	0.014883792	155391899.3	65.50924864	3.68599E+14	0.011790049
OR5BE1P	0.019885824	2.18E+22	4.400200802	1.08E+44	0.043580945
OR56A4	0.001263996	3462.501493	7.873803182	1522633.512	0.008677781
OR52E2	0.016249448	6.737311753	2.258273762	20.10002969	0.000624779
OR52B5P	0.028637759	0.000401318	5.23E-07	0.308017008	0.021032681
OR51T1	0.002488838	54932.50069	4.011194869	752289462.5	0.024716538
OR51L1	0.019441434	1117582158	11.77796486	1.06045E+17	0.026207637
OR51I2	0.017853094	3.25E-08	5.26E-15	0.201393519	0.030710294
OR4Q3	0.001602736	1.28E+41	2.23187E+16	7.34E+65	0.001137208
OR4N4	0	2.97E+69	7.17E+37	1.23E+101	1.66E-05
OR4M2	0.029257979	4.44054E+19	1239.072374	1.59E+36	0.020009291
OR4F7P	0.001820552	2.05241E+18	31429.50906	1.34E+32	0.009376457
OR4A17P	0.005792065	3.29E+33	419277.6146	2.58E+61	0.018521635
OR2A7	0.00797356	0.500212437	0.315836348	0.792221933	0.003149329
OR1M4P	1.15E-08	2.69198E+15	5514689.788	1.31E+24	0.000500074
OR1F12	0.046404572	0.035003079	0.001292525	0.947923755	0.046399682
OR14L1P	0.041705698	0.004906068	0.000113401	0.21225119	0.005668681
OR11K2P	0.000122193	1.29317E+13	1166062.91	1.43E+20	0.000264516
OR10J6P	0.000542337	3093.36109	203.3399199	47058.55515	7.18E-09
OPN1MW	0.012824144	5.41417E+18	12.73344032	2.30E+36	0.037268275

OPHN1	0.020775594	0.694525361	0.535694966	0.900448031	0.005932711
OPA3	0.015501975	1.600097213	1.154401021	2.217869739	0.004773663
OPA1	0.041183446	1.320324452	1.003759657	1.73672716	0.046945149
OMG	0.025739573	0.788864124	0.634641651	0.980563764	0.032613655
OLFM1	0.002270639	0.777632144	0.647599922	0.933773663	0.007061774
OIP5	0.00029949	1.391319232	1.177332901	1.644198684	0.000106219
OGT	0.001018859	0.742131874	0.617440325	0.892004777	0.001484588
OGFRP1	3.26E-06	3.028569095	1.85303757	4.949835292	9.83E-06
OAS1	0.027037494	1.184131394	1.035169489	1.354529063	0.013744829
NXNP1	0.012767468	831055109.3	1080.904606	6.38958E+14	0.00297602
NXNL2	0.001781088	2.507219963	1.456190543	4.316847114	0.000914374
NXF5	0.047149623	0.000291623	8.62E-08	0.987041106	0.049633433
NWD1	0.017330185	0.713540944	0.553580634	0.919722706	0.009157389
NUSAP1	0.000263608	1.273267628	1.103049945	1.469752535	0.000968624
NUS1P2	0.029950384	1.484421386	1.143036207	1.92776645	0.003050607
NUP62CL	0.000253576	1.428136812	1.163333836	1.753215364	0.000659593
NUP37	0.011708088	1.447964409	1.113882322	1.882246345	0.005677773
NUP210L	0.012233981	0.667974315	0.455496773	0.979567172	0.038861701
NUP107	0.041116194	1.255703541	1.010780248	1.559974471	0.039703261
NUMBL	0.01171301	1.368210251	1.089719786	1.717872168	0.006935623
NUGGC	0.023414152	0.648013927	0.46853897	0.896237188	0.008739896
NUF2	0.010101684	1.186024085	1.034536692	1.359693804	0.0144071
NUDT1	0.005450538	1.347834467	1.079062286	1.683552259	0.008525191
NUDCD1	0.002677274	1.346308797	1.074594417	1.686726962	0.009724303
NTSR1	0.001236254	1.399078687	1.207581282	1.620943618	7.77E-06
NT5E	0.028173464	1.123123802	1.019961913	1.236719781	0.018174794
NT5C3A	0.034488091	1.394810062	1.09838969	1.771224845	0.006336527
NT5C1A	0.025605104	0.605725706	0.390448731	0.939697333	0.025249191
NSUN4	0.034906892	0.539717835	0.33426697	0.871445185	0.01163989
NRROS	0.006274844	0.785673773	0.63216004	0.976466778	0.029655278
NRL	0.00512572	0.394150932	0.194297508	0.799572564	0.009887216
NRIP2	0.029778757	0.587311948	0.384187884	0.897829781	0.013984517

NRAS	0.032257921	1.360258335	1.077446518	1.717303556	0.00967537
NR3C2	0.025111525	0.740753816	0.609436625	0.900366327	0.002577076
NR0B2	0.001054285	0.871111696	0.777885815	0.975510251	0.016880592
NPTN	0.005040677	1.481438085	1.115362602	1.967663965	0.006649941
NPRL2	0.034849992	0.560243668	0.391190426	0.802353399	0.001569115
NPHP1	0.005061957	0.569763973	0.358465722	0.905612349	0.01734527
NPC2	0.000685934	0.801342934	0.709049175	0.905650159	0.000389156
NPB	0.008307963	5358.249431	7.470938223	3843002.861	0.010485242
NPAS2	0.000965406	1.515103419	1.23975054	1.851613123	4.91E-05
NPAS1	0.0271439	1.96164149	1.399019509	2.750524428	9.35E-05
NOTUM	0.012750484	0.872439191	0.773681911	0.983802427	0.025987928
NOS1	0.043907391	1.294288881	1.013455803	1.652942045	0.03872779
NOP2	0.027262757	1.373296301	1.089943481	1.73031241	0.007135855
NOP16	0.003013713	1.291354896	1.002117228	1.664074243	0.04811953
NOLC1	0.027358296	1.428777841	1.100471762	1.85502817	0.007391112
NOL11	0.023585491	1.446114437	1.056516527	1.979379319	0.021265712
NOD1	0.01654552	0.696823777	0.550831769	0.88150939	0.002600677
NNT	0.023770817	0.76798607	0.610566882	0.965991805	0.024095553
NMUR1	0.043837766	0.424133461	0.235847379	0.7627356	0.004176565
NMI	0.020111374	1.436550396	1.131396043	1.824009421	0.00294676
NME8	0.02726612	0.397632966	0.164537258	0.960949374	0.040516709
NMB	0.025933109	1.190930438	1.0399165	1.363874223	0.011546041
NLRP3	0.014511268	0.728742325	0.552831764	0.960627466	0.024772058
NLRP1	0.012702655	0.696946124	0.562001947	0.8642922	0.001008142
NLRC4	0.03334131	0.654107262	0.475945981	0.898959813	0.008882605
NLRC3	0.000557231	0.65523574	0.492227877	0.872225844	0.003771768
NLN	0.002906655	1.491339397	1.105740657	2.011405822	0.008831781
NKX2-1-AS1	0.021842023	0.810397444	0.710968592	0.923731407	0.001644697
NKX2-1	0.016711801	0.841760648	0.777578645	0.911240288	2.07E-05
NKIRAS1	0.005439772	0.613930116	0.399210819	0.944138208	0.026301213
NKAPP1	0.015194794	0.344417763	0.138947438	0.853729998	0.021368568
NISCH	0.005137169	0.715543393	0.558227624	0.917192782	0.008233793

NIPAL3	0.030308049	0.762693616	0.593805752	0.979615891	0.03390266
NINJ2	0.016494892	0.804373691	0.691893286	0.935139924	0.004618422
NINJ1	0.018987155	0.796490929	0.635762401	0.997853599	0.04785556
NIFK-AS1	0.021660292	0.488243605	0.311829233	0.764462702	0.001724123
NICN1	0.00812828	0.636567567	0.486490377	0.832942	0.000993262
NHSL2	0.008591445	0.519988074	0.298011288	0.907306563	0.021309601
NHEJ1	0.030431769	4.310598758	1.261412845	14.73051566	0.019786786
NGEF	0.014533973	1.320820831	1.143942381	1.52504855	0.000148687
NFIX	0.015056267	0.833918909	0.727924226	0.955347715	0.008829805
NFATC2	0.013874615	0.784022535	0.624082492	0.984952059	0.036598438
NFATC1	0.013349912	0.715241205	0.540180798	0.947034738	0.019287768
NF1P3	0.001632433	1357087.704	25.95800499	70948712663	0.010851577
NEK8	0.038602106	0.737587856	0.55766354	0.975562873	0.032893976
NEK2	0.000473885	1.300506898	1.1367318	1.487877959	0.000130159
NEIL3	0.000554053	1.297642464	1.124423134	1.497546532	0.000365046
NEDD9	0.046049372	0.82082337	0.709029788	0.950243581	0.008213036
NEDD4	0.019462313	1.567388228	1.207946515	2.033786949	0.000721057
NEDD1	0.005505815	1.666789667	1.223743527	2.270236966	0.00119213
NDUFAF7	0.0492858	0.599515227	0.397054106	0.905212922	0.014947298
NDUFA6-AS1	0.009785984	0.370025796	0.170953184	0.800915702	0.011621093
NDRG2	0.010944236	0.767929429	0.642204634	0.918267445	0.003795399
NDC80	0.00734777	1.321541616	1.141428417	1.530076014	0.000191885
NDC1	0.001372883	1.344225049	1.081106677	1.671380837	0.007776057
NCR3	0.006854333	0.691992318	0.527475784	0.907820573	0.007856461
NCOA4	0.013276599	0.776056577	0.603024235	0.998738982	0.048865024
NCL	0.029880641	1.57125237	1.181410387	2.089734471	0.001897822
NCKAP1L	0.004677281	0.827079917	0.700917578	0.975950968	0.024561502
NCF4	0.010113434	0.853672886	0.733533238	0.993489265	0.040916858
NCCRP1	0.032179653	1.14335749	1.038073892	1.259319169	0.006565726
NCAPH	0.038369871	1.256776401	1.0912102	1.447463487	0.001518933
NCAPG2	0.001650383	1.289859525	1.075381593	1.547113699	0.006083958
NCAPG	0.006722682	1.300531936	1.124044937	1.504729268	0.000413278

NCAPD2	0.000696061	1.336220556	1.117536307	1.597697868	0.001479565
NCALD	0.002699065	0.708007859	0.576234838	0.869914653	0.001015209
NBPF1	0.012323314	0.592294048	0.388423912	0.903168491	0.014970567
NBN	0.000506194	1.473047532	1.155799499	1.877374954	0.001747918
NBEA	0.020059863	0.77404867	0.610087783	0.98207399	0.034952657
NAV2-AS2	0.01102838	0.400393646	0.167633804	0.956340957	0.039355044
NAPSB	0.00204675	0.826024902	0.722769653	0.944031248	0.005026426
NAPSA	0.003436873	0.897586645	0.851129197	0.946579894	6.76E-05
NAPB	0.022169837	0.668869969	0.486265165	0.92004747	0.013427877
NANOS1	0.001508645	0.688748051	0.498356593	0.951876396	0.023900769
NAIP	0.017203787	0.177127573	0.049763089	0.630470848	0.007538118
NAALADL1	0.031031305	0.618685643	0.415845251	0.920467225	0.017845199
NAALAD2	0.035895633	0.33468736	0.115991156	0.9657256	0.042921924
N4BP2L2	0.033202185	0.676704506	0.491103138	0.93244973	0.016960491
N4BP2L1	0.006835727	0.64463428	0.496592856	0.836808967	0.000972757
MZT2A	0.040236081	1.391171059	1.097268942	1.763794493	0.006399624
MZT1	0.007264475	1.321921516	1.070600765	1.632239162	0.009485112
MYOZ3	0.048705713	0.264529647	0.084571653	0.827415947	0.022279191
MYOZ1	8.43E-05	0.712382107	0.566524031	0.895793009	0.00371459
MYO6	0.017253756	0.667195375	0.541553129	0.821987068	0.000143846
MYO1H	0.009192127	0.096032324	0.01486897	0.620231762	0.013822699
MYO1F	0.016429967	0.787456742	0.645201516	0.961076664	0.018748186
MYO1E	0.012441165	1.509729569	1.249933296	1.823524006	1.91E-05
MYO19	0.00043395	1.304746558	1.033374613	1.647382815	0.025357572
MYO16-AS2	0.005221835	42035.302	9.483690649	186316348.8	0.0129529
MYLIP	0.000109621	0.59953702	0.489574388	0.734198209	7.47E-07
MYL5	0.016038205	0.723800053	0.552550943	0.948123468	0.018940234
MYH16	0.009790157	1.627531139	1.111847244	2.382393465	0.012234276
MYEOV	0.008878418	1.132716125	1.036723875	1.237596481	0.005811801
MYBL2	0.018666811	1.142482881	1.039451662	1.25572663	0.005738024
MXD1	0.04840855	1.283117223	1.041238816	1.581183667	0.019327774
MVB12B	0.006687549	0.669139785	0.527858715	0.848234649	0.000899439

MUSK	0.002918594	0.141978041	0.035469862	0.56830681	0.005806693
MTX1P1	0.003582169	1.371603009	1.106727568	1.699871648	0.003898653
MTUS1	0.039535462	0.760147826	0.645628388	0.894980345	0.00099568
MTURN	0.00021555	0.689357816	0.576560199	0.824223037	4.49E-05
MTRR	0.008631059	0.776214059	0.611001043	0.986100225	0.038024254
MTRNR2L12	0.024261504	1.250446858	1.024250193	1.52659707	0.028140873
MTPN	0.023062124	1.395819318	1.063307961	1.832311655	0.016299919
MTND6P11	5.06E-06	1654872003	15506.97591	1.76604E+14	0.000326396
MTND4P23	0.04977706	0.14083085	0.02122465	0.934447855	0.042337819
MTND2P32	0.010614008	6.20E-06	3.49E-10	0.109929739	0.016292853
MTND1P1	0.014014102	6729644.626	2.194096602	2.06409E+13	0.039105576
MTMR9LP	0.006426468	0.732957508	0.586499548	0.915988275	0.00630445
MTMR8	0.013059086	0.375430523	0.180978136	0.778812739	0.008502843
MTMR2	0.011788222	1.613821987	1.19854183	2.172991663	0.001615406
MTMR10	0.030011985	0.587898379	0.405791335	0.851729631	0.004978039
MTL5	0.000132248	1.413577904	1.172183018	1.70468473	0.000291438
MTHFS	0.016987561	1.468558293	1.100233574	1.960186919	0.009098579
MTHFR	0.005372564	0.772942096	0.601976975	0.992462351	0.043457556
MTHFD2L	0.005238701	0.651758405	0.426595528	0.995765286	0.047749812
MTHFD2	0.007938215	1.217044681	1.037684728	1.427406337	0.015747404
MTHFD1L	0.001493834	1.406144661	1.077072044	1.835757244	0.012217829
MTHFD1	0.002284433	1.583633147	1.20743636	2.077040271	0.000893304
MTFR2	0.002148769	1.33310417	1.091874697	1.627628824	0.004757723
MTFR1L	0.020002463	0.51567685	0.344510024	0.771886435	0.001290523
MTCL1	0.022924655	1.338016891	1.071578936	1.67070212	0.010164987
MTBP	0.007666278	1.497238681	1.060595046	2.113647123	0.021768548
MTATP6P9	6.82E-05	6.35515E+11	4365.878907	9.25083E+19	0.004597628
MSTO2P	0.020826714	0.764807375	0.599874016	0.975088611	0.03050145
MSANTD3-TMEFF1	0.020572379	625.8799977	29.07955816	13470.82955	3.92E-05
MSANTD3	0.017084484	1.79200677	1.338506213	2.399158279	8.91E-05
MS4A7	0.045383471	0.823214917	0.709882205	0.954641199	0.010046942
MS4A4E	9.93E-05	0.384611484	0.211117054	0.70068235	0.001794776

MS4A2	0.022220796	0.658943112	0.51442748	0.844056824	0.000959723
MS4A15	0.003196215	0.882684698	0.804337186	0.968663752	0.008505765
MS4A1	0.000646014	0.79028853	0.691254095	0.903511408	0.000570454
MRPS18BP2	0.007010504	20804593849	38255.18737	1.13143E+16	0.000421899
MRPS18AP1	0.028494733	0.558498517	0.349162106	0.893340337	0.015074321
MRPS12	0.012117606	1.278002988	1.041324132	1.568475738	0.01890099
MRPL51	0.022004534	1.396917356	1.063320705	1.835173614	0.016352071
MRPL47	0.0068999	1.301596238	1.033642395	1.639012463	0.025005432
MRPL40P1	0.011210952	1.836031157	1.123794145	2.999668957	0.015268542
MRPL37	0.024726204	1.367390305	1.012665682	1.846370701	0.041141127
MRPL15	0.010193791	1.322321925	1.089414211	1.605023374	0.004708933
MRPL13	0.004113887	1.261044593	1.03096344	1.542473191	0.024029883
MRPL12	0.010702334	1.246943389	1.031512776	1.507366511	0.022573362
MROH8	0.000438525	0.242558531	0.102896406	0.571785189	0.001205372
MPP7	0.018904174	0.764437191	0.63652454	0.918054502	0.004039194
MPL	0.011913919	0.222116472	0.053386173	0.924129304	0.03859875
MPEG1	0.013876934	0.845076572	0.733581035	0.973518095	0.019714027
MPC1	0.000264876	0.704711517	0.550275177	0.902490867	0.005556621
MON1A	0.047043128	0.638884447	0.417886536	0.976756373	0.038588726
MOCS1	0.023899349	0.657175393	0.508367831	0.849541356	0.001351878
MOAP1	0.01345067	0.590600552	0.451988455	0.771721065	0.000113975
MNDA	0.028840634	0.853758961	0.74830969	0.974067785	0.018743736
MND1	0.004238698	1.301294572	1.081864041	1.565231396	0.005188991
MMP24-AS1	0.011774056	0.78397736	0.630780264	0.974381312	0.028241569
MMP14	0.048454864	1.224387413	1.065554376	1.406896326	0.004295192
MMADHC	0.032003764	1.449550484	1.079706421	1.946081423	0.013502384
MLLT6	0.010786439	0.704831012	0.566371996	0.877138627	0.001720054
MKL1	0.019989306	1.50941944	1.0929931	2.08450268	0.012424397
MKI67	0.000273284	1.29577319	1.132516508	1.482563961	0.000162507
MIS18A	0.010389307	1.317012204	1.066460028	1.626428652	0.010539761
MIRLET7D	0.030057117	0.834624722	0.711808899	0.978631243	0.026018271
MIR99AHG	0.007131922	0.328796372	0.166595167	0.648920709	0.001342976

MIR8071-2	0.011090071	0.843094745	0.753796273	0.942971959	0.00280887
MIR7850	0.005650056	0.084290504	0.007916107	0.897523126	0.040408356
MIR762HG	0.00406069	0.540251577	0.315498905	0.92511182	0.024857809
MIR7160	0.02053219	8.419900964	1.180790153	60.04007744	0.033522303
MIR7159	0.010632934	8.400397939	1.189224617	59.33839959	0.03286673
MIR711	0.024410985	5.895838306	1.295867573	26.82443026	0.021718661
MIR6886	0.003005022	116.8772976	4.205852243	3247.927388	0.005003575
MIR6876	0.008531746	0.008589012	0.000176243	0.418575915	0.016432195
MIR6737	0.014616327	5.29065462	1.580410945	17.71123289	0.00688422
MIR6719	0.009366339	0.581298945	0.366149451	0.922870329	0.021430225
MIR646	0.008505931	0.602425044	0.38575142	0.940802586	0.025862809
MIR5692A2	5.50E-10	2304434.359	831.7161111	6384892207	0.000291895
MIR5685	0.023997093	0.706410483	0.547930789	0.910727744	0.007331507
MIR548AK	0.049938557	3.443521316	1.174142924	10.0991445	0.024296931
MIR548AC	0.002043555	0.151987975	0.044220724	0.522387301	0.002782355
MIR526B	1.13E-07	3850.557786	30.97597839	478654.6231	0.000793028
MIR5195	0.030301878	0.777849843	0.622795335	0.971507564	0.026773441
MIR518C	9.22E-06	19017991289	4587.462938	7.88418E+16	0.002331221
MIR5186	0.032763352	0.010323598	0.000140305	0.759607345	0.037038728
MIR516A2	1.11E-16	8441945.086	3950.529848	18039716083	4.56E-05
MIR514B	0.011485322	14.28513435	1.524247157	133.8792481	0.019851321
MIR5100	0.016496263	44481.23852	4.991045448	396426079.6	0.021088107
MIR509-3	0.000493195	58.11525398	2.815003672	1199.779161	0.008538645
MIR508	0.000493195	328.1910099	4.375426839	24616.87579	0.008538645
MIR506	0.000493195	20067750.64	72.51358485	5.55364E+12	0.008538645
MIR502	0.021632058	37.76637418	1.233626556	1156.183783	0.037503768
MIR498	0.005792065	405.3887016	3.148375453	52198.34861	0.015406486
MIR4771-2	5.71E-05	32223.91399	27.31112663	38020424.67	0.00402227
MIR4721	0.023583451	2.446849629	1.010262357	5.926255755	0.047413679
MIR4697HG	0.02800018	0.801157072	0.671739553	0.955508203	0.013653368
MIR4648	0.002447503	0.619197882	0.393535599	0.974260063	0.03819774
MIR455	0.03881273	10.74075547	2.215752003	52.06531592	0.003199928

MIR4532	0.016331446	47.09638832	1.785798687	1242.060378	0.021039502
MIR4524B	0.0013629	0.036697269	0.0064851	0.207659031	0.000185873
MIR4466	0.032874078	9.799788725	1.369338516	70.13302986	0.023025999
MIR4435-1HG	0.007560288	1.346910414	1.062788631	1.706988212	0.013749378
MIR4431	0.008694807	162779.6739	3.29292412	8046715095	0.029549455
MIR4419B	0.027337914	227.3614731	1.185148781	43617.51055	0.043042245
MIR4262	0.027844536	9.380604097	1.245944822	70.62570646	0.029746104
MIR3976	0.012590356	3858.94895	3.608342232	4126960.815	0.020310611
MIR3945	0.01991024	0.205151786	0.048102207	0.874954775	0.032316351
MIR3924	0.003409036	24.10767177	4.952812198	117.343403	8.10E-05
MIR381	5.50E-10	637.5904444	19.36822821	20989.09464	0.000291895
MIR378D1	0.000220256	149.2904712	4.101616398	5433.868656	0.006342246
MIR374B	0.008185011	0.594216195	0.39804744	0.887062323	0.010890761
MIR3671	0.039484858	0.695098588	0.529637382	0.912250652	0.008739448
MIR34A	0.009026496	0.725489127	0.577200268	0.911874964	0.005946473
MIR320D1	0.03293474	0.598652528	0.380064233	0.942958631	0.026874946
MIR3157	0.028543939	0.143821927	0.029993088	0.68965045	0.015328035
MIR3135B	0.003312828	0.542623428	0.371939497	0.791634626	0.001511324
MIR3130-1	0.045934154	2.461995951	1.0821823	5.601111814	0.031691241
MIR2909	0.000300186	0.109239992	0.030563541	0.390444807	0.000656551
MIR26A1	0.017306541	0.214193449	0.056918521	0.80604403	0.022676213
MIR23A	0.003210205	0.770412148	0.626513335	0.947361922	0.013414707
MIR218-2	0.043381748	5.553462289	1.198683071	25.72902225	0.028406155
MIR211	5.50E-10	151299.6506	238.3339092	96048373.23	0.000291895
MIR193B	0.000812793	25.78658653	3.627494597	183.3077975	0.001163744
MIR186	0.002246933	0.815436188	0.697446431	0.953386736	0.010510832
MIR184	0.001483334	322.4398869	4.702852289	22107.3243	0.007413224
MIR155HG	0.006190005	0.753164674	0.585383469	0.969034926	0.027482857
MIR148A	0.009701888	0.668553345	0.495100327	0.902773741	0.008603729
MIR143	0.009400483	0.585076819	0.383428469	0.89277378	0.01291832
MIR1302-8	0.012916767	0.360343815	0.15934959	0.814860365	0.01421618
MIR1302-5	0.049543867	0.012230467	0.000179067	0.835351942	0.041008773

MIR1275	0.013964872	42.51421123	1.580701813	1143.452954	0.025577396
MIR1255B2	0.040676333	0.211229146	0.047448397	0.940342675	0.041280919
MIR1251	0.014144	9.895641932	2.012555116	48.65642111	0.004792614
MIR1193	0.000281489	8227.003202	126.6542255	534396.5543	2.30E-05
MIPEPP3	0.022950799	0.665050016	0.460588348	0.960275106	0.029537843
MIIP	0.033584951	0.754158988	0.586770868	0.969297914	0.027560048
MIF	0.01544595	1.289203066	1.083896599	1.533397694	0.004101487
MIEF2	0.028282599	0.694660337	0.498714552	0.967593549	0.031177279
MID1IP1-AS1	0.003820451	0.752975129	0.584641397	0.969776599	0.02797184
MICU3	0.004094523	0.519541865	0.33736493	0.800094277	0.002955471
MICALCL	0.032655186	0.798125146	0.643584524	0.989774809	0.040017765
MICAL3	0.02740496	1.511870707	1.047688682	2.181710152	0.02718013
MICAL1	0.022124705	0.786524059	0.639744615	0.966979763	0.022692156
MIAT_exon1	0.007682283	97.86001489	1.624792757	5894.033236	0.028372325
MGP	0.002194157	0.830807679	0.7420004	0.930243971	0.001310817
MGMT	0.017649211	0.762053188	0.622496592	0.932896771	0.008463434
MGC32805	0.012637578	0.694424985	0.546299148	0.882714282	0.002890796
MGAT5B	0.018689552	1.590193174	1.287441291	1.964139528	1.67E-05
MFSD5	0.024983653	1.690205418	1.119993059	2.550725055	0.012430556
MFSD2B	0.031443126	2.87331117	1.324610647	6.232712304	0.007551109
MFSD2A	0.022775634	0.811486884	0.71022546	0.927185802	0.002128582
MFNG	0.002274292	0.75914837	0.611307581	0.942743499	0.012649188
MFI2	0.008234437	1.410096925	1.23336193	1.612157219	4.91E-07
MFHAS1	0.02419749	0.774483615	0.628840773	0.953858109	0.016197936
MFF	0.029891987	1.401982267	1.072735366	1.832282536	0.013358477
MFAP4	0.011584424	0.865995943	0.784999594	0.955349506	0.004083028
METTL7A	0.005626276	0.720800449	0.615078135	0.844694774	5.22E-05
METTL5	0.004597998	1.457779646	1.07999221	1.96771928	0.013786229
METTL21EP	0.002793644	0.024019415	0.000728573	0.791866318	0.036544938
MESDC2	0.002272481	2.163525987	1.522025329	3.075405256	1.70E-05
MESDC1	0.049828469	1.789091515	1.304325233	2.454026317	0.000308872
MERTK	0.042078212	0.731328928	0.591750311	0.903830537	0.003782581

MELK	0.005327625	1.240160619	1.088455639	1.413009687	0.001224346
MEIS3	0.028869177	1.367630848	1.05127856	1.779180331	0.019672803
MEI1	0.038590323	0.744614023	0.589234758	0.940966287	0.013529015
MEF2C	0.046333417	0.787901266	0.639556564	0.970654419	0.025102685
MEF2BNB	0.042792377	0.56019761	0.38789198	0.809043183	0.002002241
MED6	0.026916078	1.612829528	1.077268217	2.41464386	0.020263226
MED4-AS1	0.002567675	0.276557183	0.126640801	0.603943394	0.001258106
MECOM	0.006983068	0.852589086	0.739952498	0.98237137	0.027384795
MEAF6P1	0.024272217	0.030703321	0.002014104	0.468046347	0.012204584
MEAF6	0.038281401	0.642690548	0.470160179	0.878532804	0.005572122
MDFI	5.86E-05	1.226806587	1.094340445	1.375307299	0.000454295
MCOLN2	0.002587809	0.721081276	0.570651963	0.911165196	0.006157461
MCM8	0.019350571	1.291962459	1.023630699	1.630633974	0.031037377
MCM6	0.03042768	1.293177112	1.077258401	1.552373173	0.00580864
MCM5	2.84E-05	1.479358496	1.182204508	1.85120387	0.000619265
MCM4	0.007709112	1.283214988	1.103847104	1.491728972	0.001170049
MCM10	0.01959606	1.278414254	1.077065821	1.517403091	0.004969475
MCF2L-AS1	0.032010238	0.781261887	0.651386095	0.9370328	0.007788822
MCCC1-AS1	0.008205733	0.73632065	0.546791407	0.991544659	0.043811783
MC1R	0.04586525	1.348471179	1.021011935	1.780953245	0.035163142
MBTPS2	0.010231043	1.665925811	1.191791548	2.328686432	0.002819708
MBL1P	0.026416287	0.611087398	0.454457786	0.821699659	0.001115287
MBIP	0.008232649	0.774035589	0.671596865	0.8920993	0.000405683
MAU2	0.01797899	0.662050203	0.469112506	0.93433977	0.018958044
MARVELD1	0.036428192	1.351118906	1.111083346	1.643011125	0.002565837
MARK2	0.034178273	1.388210776	1.019248008	1.890736253	0.037441973
4-Mar	0.034569723	1.766568071	1.415428628	2.204818164	4.83E-07
1-Mar	0.009008239	0.761832257	0.583350479	0.994922278	0.045788091
MAPRE3	0.001516951	0.684068459	0.556918035	0.840248702	0.000295843
MAPKAPK3	0.021663838	0.772344011	0.609792969	0.978225892	0.032148929
MAPK6	0.040004195	1.365579852	1.10024275	1.694906267	0.004704386
MAPK1IP1L	0.001558205	1.816627644	1.30487322	2.529085545	0.000405837

MAP4K4	0.0395418	1.417769284	1.157043678	1.737246209	0.000760402
MAP4K1	0.008817633	0.777056314	0.643487362	0.938350232	0.008762023
MAP3K8	0.002891632	0.70962155	0.564198137	0.892528195	0.003371229
MAP3K3	0.031267071	0.652159901	0.50070813	0.849422072	0.001522556
MAP3K12	0.006840198	0.669870495	0.473915724	0.946848685	0.023250419
MAOB	0.016594228	0.813092618	0.707701717	0.934178326	0.003486143
MAOA	0.009019671	0.880687294	0.78991578	0.981889627	0.022063484
MAN2B1	0.027308842	0.758317248	0.579976956	0.991496374	0.043135764
MAN1C1	0.031762223	0.739401507	0.572368446	0.955179471	0.020834899
MALRD1	0.037019742	0.551303934	0.305905666	0.993561289	0.047539884
MAL	0.001086066	0.804940688	0.689481441	0.939734521	0.00601771
MAGEH1	0.001643984	0.737601462	0.606480382	0.897070925	0.00230647
MAGEB18	0.033225079	1384.020581	5.374636956	356398.5781	0.010657449
MAFF	0.028270957	1.273538503	1.010579145	1.60492162	0.040447755
MAD2L1	0.000517454	1.305173287	1.115466397	1.527143547	0.000888771
MACROD2	0.046856013	0.840697632	0.716566537	0.986332003	0.033270739
LZTFL1	0.02058808	0.646713094	0.466182739	0.89715425	0.009059329
LYRM9	0.000474283	0.579944567	0.394933176	0.851626863	0.005448423
LYPD5	0.017408304	1.306575606	1.061902973	1.607623161	0.011480922
LYPD3	0.002988108	1.234718317	1.130907437	1.348058447	2.53E-06
LYL1	0.0417565	0.71135494	0.540895565	0.935533369	0.014820564
LYAR	0.001572185	1.643785837	1.267862956	2.131170302	0.000175896
LY9	0.00132622	0.524956567	0.355332129	0.775554405	0.00121012
LY86-AS1	0.001076592	0.010199234	0.000140914	0.738211256	0.035826005
LY86	0.006917582	0.83721208	0.727176608	0.963897984	0.013457967
LY6K	0.002377548	1.165758441	1.055360493	1.287704773	0.002515599
LY6G5C	0.026527873	0.570245728	0.368353396	0.882794062	0.011767094
LURAP1	0.003953749	0.414574223	0.250575916	0.685907043	0.000608977
LTBR	0.019150033	1.341896039	1.068565815	1.685141855	0.011385606
LTB	0.015423105	0.863861593	0.766179603	0.973997283	0.016834565
LTA4H	0.041966507	0.782866576	0.630233047	0.972465785	0.026946522
LST1	0.01548558	0.837603036	0.725650595	0.966827356	0.015486012

LSINCT5	0.001393744	0.177218806	0.032816987	0.957019768	0.044323295
LRRN3	0.020242432	0.594177769	0.399419276	0.883901309	0.01019999
LRRC66	0.014301935	1.33652308	1.100420833	1.623282556	0.003446178
LRRC59	0.048704017	1.404616944	1.053584302	1.872606451	0.020573072
LRRC56	0.006371302	0.765973132	0.621544988	0.943961982	0.012386577
LRRC48	0.001278235	0.671869277	0.493148847	0.915359182	0.01171996
LRRC42	0.007445629	1.350337574	1.05025508	1.736160671	0.019162489
LRRC3B	0.002558054	0.017272657	0.000672656	0.443532176	0.014249748
LRRC37A14P	0.040360946	0.266195662	0.08435836	0.839989429	0.023985801
LRRC27	0.002775428	0.480158656	0.304695069	0.756665789	0.001569124
LRRC18	0.031353053	0.69208483	0.481391018	0.994994494	0.046916171
LRRC16B	0.020329479	0.590367144	0.373137225	0.934062165	0.024362524
LRRC10B	0.013009313	0.80326359	0.689583597	0.935684081	0.004895751
LRP2BP	0.000553116	0.450778322	0.283331413	0.717185198	0.000770872
LRP12	0.048499615	1.303319676	1.034580624	1.641865448	0.024543515
LRP11	0.045821707	1.275988289	1.028842669	1.582502518	0.026498184
LRP10	0.012605428	1.335839396	1.052758755	1.695038757	0.017167188
LRMP	0.00316809	0.688390736	0.542411832	0.873656837	0.002135433
LRIG1	0.000301234	0.695204524	0.583522754	0.828261327	4.73E-05
LRIF1	0.010456076	1.369695747	1.060547456	1.768960387	0.015936081
LRFN4	0.003396672	1.332934729	1.139913358	1.558640382	0.000317361
LRFN3	0.012945679	1.408275633	1.090248724	1.819071388	0.008751816
LRCH4	0.035128652	0.730809271	0.548645074	0.97345664	0.03204304
LPXN	0.013402942	0.78345751	0.644498506	0.952377179	0.014294163
LPIN1	0.046306396	0.787633861	0.622529131	0.996527019	0.046706717
LPAR6	0.022192508	0.718418567	0.574969089	0.897657366	0.00361409
LPAR3	0.013197792	0.850118893	0.72397292	0.998244702	0.047546265
LPAL2	0.013719759	0.654696277	0.462062357	0.927639331	0.017198808
LOXL2	0.001408512	1.3074432	1.157874298	1.47633273	1.53E-05
LNX2	0.002651284	0.761681623	0.599415096	0.967875014	0.025941027
LNP1	0.014988525	0.655934818	0.446692636	0.963191355	0.031453479
LMOD3	0.013196612	0.049463429	0.006126181	0.399372904	0.004783429

LMO7DN-IT1	0.001791581	0.495297954	0.287052752	0.854616654	0.011588473
LMO7DN	0.000466323	0.241861462	0.093656794	0.624588611	0.003364636
LMO3	0.043391319	0.868876268	0.784722228	0.962055032	0.006846401
LMNB2	0.001677142	1.347114791	1.119452588	1.621076479	0.001607046
LMF1	0.017048591	0.75455893	0.57484631	0.990454611	0.042451338
LLPH	0.023567632	1.436500791	1.044223811	1.976141993	0.02602119
LLNLR-276E7.1	0.002881362	0.035564793	0.001995974	0.633702875	0.023184135
LLNLF-187D8.1	0.027827046	0.153362411	0.03901835	0.60279405	0.007257975
LL22NC03-75H12.2	0.002084602	0.775707351	0.624099613	0.964143994	0.022075766
LL0XNC01-36H8.1	0.040906317	0.563410344	0.321234702	0.988159788	0.045336918
LL0XNC01-220B11.1	0.023462113	9363.436159	1.369652771	64011798.15	0.042378391
LIPT1	0.019075883	0.610910345	0.400407872	0.932078202	0.022237405
LIPK	5.17E-05	1.328763303	1.017694933	1.734912749	0.036720158
LINGO3	0.00223874	0.396612955	0.169360765	0.928797386	0.033163525
LINGO2	0.019115658	2.68968167	1.814043113	3.987990932	8.50E-07
LINC01559	0.00153953	1.182254613	1.013020779	1.379760413	0.03366247
LINC01480	0.003928189	0.629851307	0.444342854	0.892807581	0.009406142
LINC01468	0.045142133	1.354086025	1.141247709	1.606617869	0.00051223
LINC01420	0.005334948	1.352946588	1.052798425	1.738665663	0.018176699
LINC01412	0.030784938	0.268005244	0.092125784	0.779660236	0.015657773
LINC01374	0.031087438	0.258763432	0.106308665	0.629850011	0.00289681
LINC01353	0.02054993	0.401518147	0.171513482	0.939965886	0.035498713
LINC01352	0.025271729	0.191305027	0.076308726	0.479599322	0.000420374
LINC01351	0.011635575	2.545598279	1.512559639	4.284175268	0.00043486
LINC01322	0.007518514	2.327918462	1.698177408	3.191188589	1.52E-07
LINC01315	0.03210432	0.765677306	0.609517804	0.961845138	0.02177665
LINC01312	6.77E-05	7.404668023	3.872762228	14.15762324	1.41E-09
LINC01281	0.03078505	0.353557095	0.157250672	0.794925821	0.011897733
LINC01269	0.012620643	1.260816453	1.037449988	1.532274468	0.019831948
LINC01259	0.005234157	0.006101414	5.92E-05	0.629356599	0.031105267
LINC01215	0.00397066	0.605202369	0.4182667	0.875685076	0.007716715
LINC01177	0.013417438	15598558.28	14.99582538	1.62255E+13	0.019128632

LINC01170	0.038218593	0.093789971	0.022919689	0.383799226	0.000994749
LINC01150	0.008714627	0.519720011	0.352770472	0.765678852	0.000931296
LINC01132	0.004142638	0.630957652	0.398709362	0.99849062	0.049250815
LINC01128	0.001055641	0.551705626	0.305432686	0.996550506	0.048676087
LINC01117	0.002891246	2.252059848	1.640534284	3.091537684	5.11E-07
LINC01116	0.00585834	1.475403942	1.264028587	1.722126235	8.23E-07
LINC01111	0.034454314	134541235.5	2.853923291	6.34262E+15	0.037866741
LINC01066	0.001020762	18194.63701	13.29654626	24897052.92	0.007762204
LINC01031	0.000290393	0.117219579	0.02213052	0.620881455	0.011725142
LINC00996	0.00276891	0.504710368	0.330426642	0.770920147	0.001557493
LINC00987	0.006879681	0.538075929	0.327636292	0.883680204	0.014344546
LINC00982	0.000131184	0.645830195	0.474739153	0.878580664	0.005364031
LINC00930	0.033721997	0.676483684	0.475031935	0.963367178	0.030244677
LINC00926	0.047024542	0.70672534	0.543044741	0.919741356	0.009812116
LINC00921	0.001241656	0.427722345	0.234472839	0.780245613	0.005622324
LINC00908	0.002918059	0.320622191	0.167383769	0.614149092	0.000603474
LINC00892	7.36E-05	0.338832094	0.188686779	0.608453799	0.000290804
LINC00865	0.028006727	0.681639081	0.503848731	0.922165339	0.012938447
LINC00862	0.020066984	1.384395095	1.059361807	1.809155067	0.017203325
LINC00861	0.000301855	0.603700367	0.408357994	0.892486833	0.011399031
LINC00847	0.020572193	0.644302848	0.490345465	0.846599367	0.001603505
LINC00686	0.043321963	20.42149782	1.402261841	297.4034956	0.027289263
LINC00683	0.001479955	0.211927185	0.074995201	0.598880075	0.003419454
LINC00680	0.004257454	0.710425111	0.548889574	0.919499771	0.009387529
LINC00654	0.008408688	0.673567926	0.492426565	0.921342965	0.013415085
LINC00639	0.001230515	0.080685843	0.015817201	0.411590212	0.002463853
LINC00628	0.025496955	4.973956934	1.910606458	12.9488977	0.001015544
LINC00615	0.027757211	54.49865897	1.364465979	2176.751839	0.033574653
LINC00563	0.037880814	0.016237359	0.000539609	0.488598013	0.017676729
LINC00539	0.008983856	0.272534361	0.129064723	0.575486284	0.000652414
LINC00533	0.001481818	9.438419299	3.05341626	29.17511118	9.67E-05
LINC00528	0.00909869	0.456312817	0.232270929	0.896459097	0.022773585

LINC00476	0.048286966	0.488121122	0.258320285	0.922351994	0.027180842
LINC00460	0.038573685	1.17538736	1.052750238	1.312310742	0.004049049
LINC00426	0.002507946	0.541135038	0.342112339	0.855938521	0.008667849
LINC00339	0.025935135	0.749422266	0.581349994	0.966085384	0.02599704
LINC00336	0.022467752	0.338594388	0.120404937	0.952171586	0.040085336
LINC00324	0.001400013	0.523224311	0.375783984	0.728513432	0.000125274
LINC00265-3P	0.001478448	7.00E+49	5.75291E+11	8.52E+87	0.010312846
LINC00264	0.016932136	0.10658665	0.01551036	0.732459706	0.022812334
LINC00240	0.000400276	0.298338361	0.129856803	0.685414826	0.004371743
LINC00211	0.005870078	0.001450846	5.06E-06	0.415900125	0.02358314
LINC00115	0.025323649	0.639585181	0.428452897	0.954758872	0.028782651
LINC00092	0.024703805	0.413200551	0.218368667	0.781864441	0.006603359
LIMD1	0.000657577	0.713551713	0.561253132	0.907177204	0.005864718
LIMCH1	0.000272092	0.819222764	0.697562184	0.96210195	0.015057961
LILRA4	0.000200123	0.558414523	0.375875343	0.829601584	0.003915068
LIFR-AS1	0.000108637	0.382558263	0.22394977	0.653498436	0.000436242
LIFR	0.012090763	0.73152429	0.61707445	0.867201337	0.00031659
LHFPL3-AS2	0.00330049	0.866738661	0.75292403	0.99775791	0.04645722
LGR4	0.0019522	1.285734346	1.095068557	1.509597549	0.002148517
LETM2	0.008855295	1.722329046	1.287938589	2.303228872	0.00024597
LEO1	0.025887208	1.447283412	1.033416976	2.026896521	0.031457168
LDLRAD3	0.000164101	1.696797306	1.394664474	2.064382619	1.26E-07
LDHD	0.045019678	0.80495908	0.697021108	0.929611906	0.003141295
LDHA	0.001493212	1.733831391	1.404333753	2.140638781	3.09E-07
LDB1	0.006471616	0.601178836	0.446922119	0.808677794	0.000769159
LCN10	0.003037468	0.099366828	0.010576172	0.933586035	0.043374157
LCE3E	4.11E-05	1.800850909	1.03900484	3.121317507	0.036053882
LCA5	0.004821472	0.638903897	0.483217617	0.844750222	0.001666796
LBR	0.030348235	1.324488854	1.032176417	1.699584193	0.027182341
LBH	0.019436794	0.841753694	0.716829056	0.988449444	0.035579271
LAX1	0.03142798	0.766745167	0.635112365	0.925660061	0.005713334
LATS2	0.00907702	1.346788828	1.024159517	1.771052378	0.033104042

LARP6	0.026661474	1.291959382	1.042539256	1.601051506	0.019251792
LARP1	0.021114668	1.366187549	1.054552386	1.769915314	0.018173607
LANCL1-AS1	0.003571108	0.576161411	0.357144408	0.929489483	0.023844538
LAMTOR3P2	0.023527916	0.316390691	0.119718657	0.836152624	0.020294776
LAMP3	0.047500605	0.898365366	0.811257945	0.994825796	0.039431519
LAMC2	0.003018706	1.208478654	1.096782472	1.33154996	0.000129739
LAMB3	0.047519934	1.135276893	1.006682195	1.280298421	0.03858876
LAD1	0.00407578	1.215623466	1.029764017	1.435028206	0.021087668
LA16c-390H2.4	0.025929231	0.37623496	0.150519088	0.940430529	0.036495702
LA16c-390E6.3	0.004096975	0.174835183	0.049434403	0.618341473	0.006813275
LA16c-380H5.5	0.01356443	0.768575326	0.612382305	0.964606629	0.023156204
LA16c-380H5.2	0.010732906	0.497421985	0.281431009	0.879180415	0.016257809
LA16c-313D11.12	0.025985847	0.534676024	0.316686183	0.902718419	0.01913166
L3MBTL4-AS1	0.047254648	0.39054064	0.154853265	0.984945275	0.046359668
L3MBTL3	0.007903251	1.391182509	1.045743926	1.850729157	0.023383729
KSR2	0.012925224	0.486626248	0.248136006	0.95433593	0.036084176
KRTAP10-12	0.049391432	10570521.19	3.254672078	3.43309E+13	0.034495905
KRT9	0.000480401	2.284439063	1.173914237	4.445522225	0.015015884
KRT8P8	0.047414647	2.260051931	1.328384707	3.845147195	0.002636137
KRT8P5	0.002033133	19.51402667	4.566645844	83.38663651	6.08E-05
KRT8P47	0.040211089	82268300.27	17.31459993	3.90888E+14	0.020152474
KRT8P36	0.003030496	2.008756563	1.137982557	3.545838998	0.016137967
KRT8P33	0.024326498	2.148740381	1.396409558	3.306397612	0.000504291
KRT8P21	0.041278501	2971.931004	9.617509001	918363.9847	0.006261309
KRT8P20	0.02665962	36.25424188	4.53419011	289.879785	0.000711469
KRT8P2	0.031132687	241.9711614	14.32392645	4087.569366	0.000141484
KRT8P18	0.000261786	130.2095663	9.875344521	1716.85465	0.000215381
KRT8P12	0.02067792	1.302102556	1.011382932	1.676388846	0.040582968
KRT81	0.005103197	1.144631326	1.071788997	1.22242426	5.66E-05
KRT80	0.027911636	1.163559682	1.022873768	1.323595516	0.021226343
KRT8	0.004951911	1.468128943	1.233149279	1.747884567	1.60E-05
KRT6C	3.28E-07	1.45671954	1.126324918	1.88403167	0.004151814

KRT6B	0.046056912	1.146550795	1.03048151	1.275693657	0.012027127
KRT6A	0.00429395	1.155784859	1.094348355	1.220670397	2.05E-07
KRT19P6	0.006167919	9890.327734	5.262837327	18586662.78	0.016769631
KRT18P68	0.036154648	9.007297788	2.082964091	38.94998181	0.003259085
KRT18P54	0.000825295	41.19479891	2.083025075	814.6860436	0.014611149
KRT18P51	0.020683907	75.73097843	7.396104462	775.4326786	0.000266495
KRT18P44	0.046201017	77340.99907	9.719473986	615427351.8	0.014041066
KRT18P40	0.027653025	346.3999205	10.54593536	11378.11876	0.001029888
KRT18P39	0.021035731	3.969982215	1.03333556	15.25231435	0.044673611
KRT18P38	0.025199924	3.485823937	1.297609635	9.36411706	0.013260751
KRT18P29	0.037034094	156.0159545	13.14176558	1852.18477	6.32E-05
KRT18P28	0.000356242	3.177931363	1.703598856	5.928184157	0.000278339
KRT18P24	0.005227788	5724.382708	63.62052067	515062.7037	0.000163939
KRT18P2	0.004925387	1156.944212	18.89288237	70847.84016	0.000780034
KRT18P18	0.035283862	13.07867349	1.763584941	96.99090539	0.01190503
KRT18P17	0.000492363	3.832627591	1.982408681	7.409690237	6.48E-05
KRT18P13	0.011056395	3.444159643	1.711716198	6.930024769	0.000526921
KRT18P11	0.014971982	1.998495578	1.292638262	3.089792942	0.001841828
KRT17	0.044907772	1.097300126	1.024694882	1.17504985	0.007851371
KRT16	0.000692888	1.142319932	1.05136745	1.241140599	0.001670755
KRT14	0.042475854	1.148873682	1.04288143	1.265638356	0.00495175
KREMEN2	0.002561632	1.353917399	1.056146572	1.735641975	0.016801033
KRBOX4	0.005969861	0.626343081	0.416769957	0.941300227	0.024384477
KPNB1	0.00300951	1.356833045	1.026533053	1.793411236	0.032036891
KPNA5	0.038040102	0.596063471	0.409665342	0.867272931	0.006846438
KPNA4	0.017529854	1.705285365	1.218077582	2.387366964	0.001876337
KPNA2	0.001118726	1.353263936	1.159398529	1.579545975	0.000125674
KPNA1	0.044038459	1.430359524	1.011723943	2.022219976	0.042771132
KNSTRN	0.00400801	1.341891133	1.108120227	1.624978743	0.002602276
KNOP1	0.02717821	1.500544576	1.050886442	2.142604504	0.025543923
KNDC1	0.007567532	0.822814939	0.688242448	0.983700477	0.032328963
KMT2C	0.014269866	0.749765591	0.582441201	0.965159127	0.02540453

KLRG2	0.020020824	0.788521552	0.638279065	0.974129142	0.027594939
KLRG1	0.000515363	0.496141622	0.320262495	0.768608604	0.00169893
KLRB1	0.016591323	0.754918691	0.631276391	0.902777671	0.002065098
KLK6	0.032575921	1.123800405	1.0341982	1.221165682	0.005902265
KLK13	0.013394376	0.863156267	0.752435953	0.99016898	0.035639445
KLK11	0.008853588	0.894062081	0.824364004	0.96965297	0.006847966
KLHL9	0.01959455	0.745299216	0.588864814	0.943291073	0.014458943
KLHL8	0.008486671	0.643437935	0.443462416	0.933590674	0.020243227
KLHL35	0.018665478	0.750693648	0.594595201	0.947772454	0.015910715
KLHL32	0.004921897	0.423109388	0.246873246	0.725155751	0.001753508
KLHL24	0.040649471	0.760553923	0.579791757	0.997672463	0.048064958
KLHL11	0.002270801	0.673154103	0.48685915	0.930734171	0.016657073
KLHDC9	0.028427647	0.751134096	0.628144429	0.898204942	0.001708643
KLHDC8B	0.001944975	0.633197745	0.516460543	0.776321425	1.11E-05
KLHDC7A	0.001565613	0.783111217	0.666841451	0.919653655	0.002869613
KLF4	0.045918866	1.155861677	1.012810145	1.319118121	0.031650581
KLF15	0.00299756	0.800802319	0.684262978	0.937189903	0.005633514
KLC4	0.012220903	0.68973749	0.520598473	0.913828661	0.009660473
KL	0.002077915	0.630253097	0.455589898	0.871878346	0.005303435
KIT	0.009100671	0.873658312	0.793201496	0.962276106	0.00614251
KIRREL3	0.015312745	1.564277179	1.026612709	2.38353088	0.037324822
KIFC1	0.019722175	1.216917773	1.068563591	1.38586873	0.00307908
KIF4A	0.004864936	1.318787916	1.146095595	1.517501311	0.000111456
KIF23	0.000396967	1.331130474	1.13718014	1.558159763	0.000370964
KIF20B	0.007636459	1.455175076	1.157838474	1.828868663	0.001296969
KIF20A	0.00414649	1.381915259	1.180291445	1.617981551	5.82E-05
KIF18B	0.000712551	1.197819213	1.039986835	1.379604837	0.012285439
KIF18A	0.000100391	1.629318124	1.288459026	2.060350773	4.57E-05
KIF14	3.57E-05	1.483223959	1.227639286	1.792019316	4.40E-05
KIF11	0.001155491	1.324318256	1.13730774	1.542079404	0.000298648
KIAA1524	0.010722849	1.266190805	1.054438152	1.520467703	0.011482063
KIAA1328	0.002620876	0.312579095	0.161292866	0.60576573	0.000571371

KIAA1324	0.007864933	0.856658775	0.783344011	0.936835218	0.000700598
KIAA1147	0.007020405	0.771594387	0.607593912	0.979861527	0.033434027
KIAA1109	0.013426553	0.711852115	0.547490564	0.925556469	0.011164074
KIAA0408	0.002616381	0.048040211	0.004325932	0.533494647	0.013455062
KIAA0226L	0.00068057	0.558165997	0.395302685	0.78812842	0.000924423
KIAA0125	0.008724874	0.643434186	0.475685476	0.870338854	0.004222371
KIAA0101	0.000806402	1.304912968	1.121265709	1.518639016	0.00058392
KHDRBS2	0.01751808	0.703985026	0.559661853	0.885525632	0.002712539
KHDRBS1	0.034247608	1.723830461	1.12620974	2.638577305	0.0121689
KF459411.1	0.004285649	34750.61205	4.705853091	256617666.2	0.021404652
KDM4C	0.022350061	0.498691184	0.302374654	0.822466083	0.006418165
KDM4A	0.002806009	0.70593589	0.517752676	0.962516476	0.027701554
KCTD7	0.009589193	0.581197261	0.404296388	0.835501543	0.003384094
KCTD3	0.0041575	1.282090323	1.053788749	1.559853053	0.013005899
KCTD12	0.003826822	0.850724286	0.741635782	0.97585881	0.020944308
KCP	0.009191104	1.257735051	1.006038188	1.572402993	0.044135128
KCNV1	0.003617498	2.856536453	1.840800084	4.432746705	2.84E-06
KCNS2	0.048878794	0.065886819	0.006019298	0.721192583	0.025902514
KCNQ1DN	0.006999339	106.2775853	1.691694567	6676.692918	0.027186006
KCNQ1	0.006149154	0.846984604	0.753882836	0.951584099	0.005185656
KCNK3	0.003442541	0.805904992	0.688522978	0.943298737	0.007215242
KCNK17	0.019295519	0.775412408	0.605047198	0.993747934	0.044482865
KCNK16	0.011756131	0.312354581	0.117391902	0.8311083	0.019781385
KCNJ15	0.039618008	0.869630421	0.773104754	0.978207758	0.019964763
KCNJ14	0.001706082	1.628099386	1.083109179	2.447313403	0.019084348
KCNJ11	0.006628471	0.800406843	0.666029401	0.961896146	0.01758409
KCNF1	0.000184198	1.345367533	1.136384213	1.592783302	0.000572477
KCNAB1	0.038093698	0.462986537	0.237880843	0.901108852	0.023424592
KCNA5	0.009099364	0.459954045	0.252298167	0.838522633	0.011252284
KCNA3	0.049491141	0.783848048	0.649269551	0.946321543	0.011275631
KCNA2	0.011725146	0.078560217	0.010886812	0.566897605	0.011640962
KCMF1	0.040614166	1.574844548	1.101263625	2.252081422	0.012828495

KBTBD8	0.007457142	0.540462022	0.36684265	0.796252009	0.001855756
KBTBD3	0.046837167	0.62397107	0.411610036	0.945895051	0.026282308
KB-176G8.1	0.009495124	4.138880032	1.227191754	13.95896596	0.02201957
KB-173C10.2	0.034611504	1.586768238	1.013654743	2.483916204	0.043458119
KB-1732A1.1	0.00163197	2.543106576	1.643703058	3.93464685	2.77E-05
KB-1572G7.3	0.028319063	0.306843261	0.10524064	0.894642859	0.030473837
KB-1562D12.1	0.039024828	0.77436847	0.627658158	0.9553712	0.017034291
KB-1448A5.1	0.045025294	0.023487112	0.001335528	0.413053389	0.010335807
KAT8	0.005717733	0.618660981	0.441465005	0.866980179	0.005287193
KAT2B	9.78E-05	0.72272135	0.566450743	0.922103389	0.008991582
KANK1	0.038035595	0.678331533	0.513781617	0.895582194	0.006182796
KAL1	0.012517728	0.784195904	0.676070214	0.90961442	0.001320567
JPH4	0.018326172	0.303767647	0.129330473	0.713480598	0.006240542
JOSD1	0.022221037	1.608479114	1.206174905	2.144966744	0.00121047
IZUMO4	0.021078379	0.728255278	0.557509375	0.951294765	0.020004801
ITPR2	0.025821871	0.786209542	0.641624196	0.963376145	0.02035095
ITPKB	0.003350518	0.741582943	0.595783969	0.923061529	0.007433736
ITIH4-AS1	0.002397323	0.116182792	0.027330001	0.493905622	0.003553177
ITGB7	0.028107915	0.737230101	0.575265738	0.94479505	0.016012292
ITGB4	0.001665244	1.177477816	1.057213215	1.311423266	0.002957745
ITGB1P1	0.009031052	1.246380327	1.050063608	1.479399826	0.011782347
ITGB1	0.00287234	1.40396976	1.157824756	1.702443376	0.000560601
ITGAL	0.013126341	0.794240678	0.677905051	0.930540721	0.004360357
ITGA9-AS1	0.047775495	0.281092944	0.104234318	0.758034831	0.012166453
ITGA6	0.01696675	1.283225594	1.139489387	1.445092814	3.88E-05
ITGA4	0.004426879	0.749972142	0.605756725	0.928521619	0.00827604
ITFG2	0.027450745	0.688864456	0.485232929	0.977951433	0.037099523
ISCA1P6	0.013408744	0.036118117	0.002454068	0.531573862	0.015497218
IRX6	0.036071768	0.726978234	0.602348748	0.877394291	0.000890066
IRX5	1.90E-05	0.747054228	0.660768367	0.844607652	3.21E-06
IRX3	0.001145858	0.817168233	0.732648622	0.911438171	0.000289332
IRX2	0.001536748	0.854751703	0.780670002	0.935863388	0.000691293

IRF8	0.001153514	0.785353436	0.667913353	0.923443164	0.003458855
IRF4	0.042589762	0.779695075	0.656272375	0.926329422	0.004650054
IQCG	0.009669211	0.728760511	0.545873137	0.972921813	0.031859904
IQCF5-AS1	0.027023114	1.62027E+19	2869.26753	9.15E+34	0.016838885
IQCC	0.010607863	0.643567971	0.472966783	0.875705753	0.00503859
IQCB1	0.002925079	0.737069488	0.567758317	0.956870932	0.021960624
IPO5P1	0.003705157	0.631544845	0.486574047	0.819708521	0.000552031
INTS8	0.047307684	1.35637899	1.028190774	1.789321605	0.031032136
INTS7	0.007020967	1.600683288	1.18945795	2.154079503	0.001901699
INTS10	0.019149224	0.721097387	0.526348625	0.987903104	0.041776798
INPP5J	4.82E-06	0.592329673	0.460038328	0.762663501	4.89E-05
INPP5B	0.000705273	0.586855281	0.440589667	0.781677708	0.000268393
INPP5A	0.03354342	0.629936163	0.462953245	0.857148263	0.00327272
INMT-FAM188B	0.011505138	0.000799179	7.35E-06	0.086938894	0.002874512
INHA	0.019078679	1.115606959	1.030619374	1.207602844	0.006810543
ING4	0.024388715	0.642267824	0.476176553	0.86629204	0.003729819
ING2	0.046951908	1.553947094	1.14883165	2.101919433	0.004232971
INCENP	0.018496389	1.315914973	1.082721017	1.599333706	0.005805423
INAFM2	0.000940094	0.670198266	0.542300224	0.828260245	0.000212204
IMPAD1	0.022645068	1.332338799	1.03567057	1.713987754	0.025569678
ILF3-AS1	0.001129411	0.689436635	0.541709148	0.877450335	0.002506621
IL7R	0.012523275	0.855911177	0.751178096	0.975246677	0.019473474
IL6ST	0.004395105	0.792793347	0.652347799	0.96347576	0.019597084
IL6R	0.011285598	0.843161043	0.712360367	0.997978799	0.047312979
IL33	0.03771703	0.840990499	0.739801353	0.956020175	0.008106942
IL24	0.037623566	0.732022308	0.56375759	0.950508993	0.019239729
IL23R	0.017880985	0.043528001	0.002714031	0.69810811	0.026842858
IL22RA1	0.008179771	1.273008322	1.105931907	1.465325466	0.000772076
IL1R2	0.000238082	1.264615284	1.106459661	1.445377424	0.000573023
IL17F	0.012354847	0.000437152	9.96E-07	0.191879138	0.012710861
IL16	0.000807067	0.656299107	0.518522636	0.830684116	0.000460091
IL11RA	0.002093187	0.653467546	0.492074712	0.867794714	0.003284969

IL11	0.003696695	1.40302866	1.117641906	1.761288127	0.00351688
IL10RB-AS1	0.014581344	0.628410377	0.406322381	0.971887398	0.036785558
IKZF5	0.012804918	0.599143605	0.421852119	0.850945257	0.00421444
IKZF4	0.00030933	0.549302383	0.40028148	0.75380232	0.000207048
IKZF3	0.009466374	0.686468444	0.559862759	0.841704359	0.000298399
IKZF1	0.004409001	0.768369186	0.626427967	0.942472616	0.011453682
IGSF6	0.015628682	0.825124525	0.711559712	0.956814262	0.01094991
IGSF10	0.017488326	0.614613245	0.440180795	0.858168838	0.004262647
IGLV4-69	0.049113468	0.930492806	0.868746907	0.996627273	0.039743897
IGLV4-60	0.031806012	0.912188109	0.846504302	0.982968598	0.015930974
IGLV3-6	0.041874225	0.646940631	0.46185316	0.906201834	0.011316208
IGLC5	0.044660993	0.494096595	0.260292702	0.937911216	0.031084953
IGLC3	0.039976748	0.909893938	0.846314767	0.978249478	0.010619516
IGKV1OR2-6	0.001495049	0.85140579	0.756893549	0.957719643	0.007372148
IGKV1D-12	0.016656136	0.880414646	0.77748759	0.996967616	0.044660132
IGKV1-8	0.037410982	0.88586558	0.807736654	0.971551585	0.010093086
IGKC	0.017686306	0.928904744	0.864660885	0.997921887	0.043710716
IGJ	0.023565584	0.8772829	0.810709867	0.949322709	0.001147786
IGHVIII-5-1	0.009777058	1.64078514	1.024936814	2.626674971	0.039154477
IGHV5-51	0.049098599	0.914096632	0.85373688	0.978723857	0.009966724
IGHV3-13	0.02722105	0.912514808	0.84213393	0.988777729	0.025381576
IGHV2OR16-5	0.014531718	0.575818672	0.340864652	0.972723751	0.03907992
IGHJ3P	0.021158743	0.88306684	0.810619672	0.961988798	0.00440986
IGHD4-4	0.042085463	0.789489195	0.625901183	0.995833219	0.046020027
IGHD2-2	0.003669506	0.793033515	0.680562347	0.924091906	0.00296245
IGHD	0.022902753	0.939603416	0.883940215	0.998771823	0.045564444
IGFBPL1	0.010358425	0.826969578	0.709639812	0.963698303	0.014948869
IGFBP1	0.002888141	1.29367766	1.158317995	1.444855296	4.96E-06
IGFALS	0.000245481	0.771934373	0.615971944	0.967386066	0.024582248
IGF2BP3	0.002333431	1.249261937	1.09630131	1.423564283	0.000838813
IGF2BP2	0.021749038	1.181752212	1.040780644	1.341818085	0.009974947
IGF2BP1	0.018960459	1.328711366	1.182347035	1.493194335	1.82E-06

IFT57	0.019130454	0.849668442	0.740289112	0.975208806	0.020503416
IFNWP19	0.021795527	1.253155859	1.051878013	1.492948411	0.011532573
IFNA13	0.000866354	2.14279E+17	12483.45936	3.68E+30	0.010269975
IFNA11P	0.042365386	15498.92511	1.344368013	178683721.4	0.043178536
IFFO1	0.03118025	0.774306262	0.607130972	0.987513757	0.0392825
IER5L	0.000273736	1.29939396	1.124206134	1.501881738	0.000393429
IER3	0.01522666	1.173169808	1.03406341	1.330989362	0.013133648
ID2	0.011105491	0.815592303	0.68551139	0.970357043	0.02148197
ICOS	0.028300814	0.735413328	0.567109826	0.953664948	0.020461865
ICAM3	0.002295168	0.655897123	0.506335788	0.849635846	0.001403203
HTRA4	0.01312627	0.747613505	0.581574228	0.961056948	0.023210446
HTR5BP	0.020071612	0.008877211	0.000228737	0.344521661	0.01137997
HTR1D	0.006764751	1.329255764	1.137619118	1.553174396	0.000339369
HTATIP2	0.021106499	1.408803894	1.144263827	1.734502451	0.001238191
HSPE1P5	0.016320026	2.226541784	1.15448389	4.294116495	0.016910675
HSPE1P21	0.018085628	3.147255154	1.622449715	6.105098304	0.000695215
HSPD1P2	0.020090757	3629.524734	17.48526971	753402.7219	0.002603311
HSPD1	0.018575585	1.431951431	1.150001958	1.783027312	0.00133092
HSPBP1	0.049924163	1.313899049	1.027756371	1.67970811	0.029373932
HSPBAP1	0.017532533	0.683005677	0.466958267	0.999011662	0.049407221
HSPB7	0.023406505	0.773224881	0.602856245	0.991740107	0.042838726
HSPA4	0.004297058	1.904554024	1.352898698	2.681151247	0.000222384
HSPA2	0.017809389	1.24456776	1.067346533	1.451214635	0.005245633
HSFX1	0.017170816	0.001643845	4.04E-06	0.668486029	0.036496831
HSF2BP	0.013513427	1.844075737	1.267753304	2.682395158	0.001370297
HSDL1	0.000688674	0.621705153	0.468783549	0.824511222	0.000968337
HSD17B7P2	0.003608622	0.716156434	0.548662485	0.934782406	0.01404471
HSD17B6	0.001290348	0.864959715	0.775368309	0.964903131	0.009312407
HSD17B13	0.026427234	0.80088776	0.659523879	0.972551904	0.025035597
HSD11B1L	0.006810819	0.661431317	0.483073463	0.905641523	0.009933249
hsa-mir-4538	0.015482914	0.801198039	0.677428151	0.947581374	0.009629822
HS3ST2	0.002838781	0.787314196	0.672094545	0.922286376	0.003055877

HPSE2	0.010721962	0.400779601	0.219340068	0.732307096	0.002949189
HPS1	0.02848183	0.627792144	0.457881284	0.860753627	0.003837997
HPGDS	0.02942176	0.750858722	0.628241934	0.897407178	0.001633227
HOXD8	0.046134404	1.35637763	1.128043442	1.63093034	0.001190921
HOXD13	0.047539892	1.2285791	1.011381648	1.492420401	0.038080765
HOXD12	0.028695837	3.581189181	1.157425765	11.08055164	0.026852495
HOXC6	0.006591843	1.242585893	1.028938414	1.500594867	0.024050439
HOXB7	0.004475887	1.138576841	1.024944377	1.264807389	0.015552307
HOXA13	0.018338417	1.862538128	1.401992873	2.47436941	1.77E-05
HOXA10-AS	0.013659338	2.026133367	1.222277739	3.358660876	0.006175209
HOXA1	0.043069392	1.280405854	1.064418168	1.540220939	0.008735029
HNRNPL	0.01434392	1.693424865	1.04384603	2.747232535	0.032859668
HNRNPK	0.03670698	2.073752075	1.222919665	3.51654143	0.006793223
HNRNPF	0.041326841	1.482375067	1.079131766	2.036299836	0.015094438
HNRNPC	0.00131025	1.767546609	1.210873261	2.580138744	0.003163053
HNRNPAB	0.037492025	1.512848146	1.129413125	2.026459108	0.005503223
HNRNPA3P1	0.007885062	6.666441572	1.486844592	29.88977024	0.013208291
HNRNPA2B1	0.005543851	1.538996642	1.089994279	2.172956968	0.014302544
HMMR	0.000223709	1.403748593	1.204042256	1.636578867	1.48E-05
HMHA1	0.000694399	0.733156122	0.597729124	0.899266704	0.002892284
HMGN3	0.005599139	0.740364836	0.612908459	0.894326194	0.001817131
HMGN1P36	0.014241815	1.461703537	1.07370986	1.989901845	0.015872766
HMGCLL1	0.001031089	0.37757347	0.19914614	0.715864867	0.002844451
HMGB3P7	0.027058755	0.405508883	0.187181753	0.878490833	0.022113385
HMGA2	0.033318831	1.257960698	1.10848725	1.427589824	0.000376801
HMGA1P3	0.043493612	1.601079081	1.101791544	2.326623614	0.013574795
HMGA1	0.000804859	1.31631808	1.154320989	1.501049799	4.10E-05
HMCN2	0.011876527	0.43371498	0.214159113	0.878359463	0.020330143
HMBOX1	0.022584083	0.687078018	0.479207381	0.985118807	0.041199171
HLFP1	0.049855415	252368950.5	1612.131574	3.95068E+13	0.001523689
HLF	0.00133174	0.791660362	0.685024281	0.914896225	0.00155135
HLA-DRB5	0.006490174	0.876966237	0.806219713	0.953920835	0.00221917

HLA-DRB1	0.000888757	0.868319732	0.785668259	0.959666053	0.005663119
HLA-DRA	0.002537256	0.862318692	0.779105666	0.954419354	0.004222946
HLA-DQB1-AS1	0.003718233	0.787011703	0.687053949	0.901512059	0.00054817
HLA-DQB1	0.004469014	0.877001684	0.798492434	0.963230109	0.00609
HLA-DQA1	0.014508266	0.857780863	0.774776614	0.949677618	0.003133716
HLA-DPB1	0.002171419	0.852625606	0.767077873	0.947713979	0.003122124
HLA-DPA1	0.005173454	0.872078234	0.787406822	0.965854529	0.008622554
HLA-DOB	0.002744526	0.768491613	0.65771498	0.897925967	0.000914273
HLA-DOA	0.012590702	0.849140029	0.758895191	0.950116429	0.00433682
HLA-DMB	0.001045247	0.821047531	0.726065677	0.928454642	0.001669825
HLA-DMA	0.000777719	0.795610019	0.703284703	0.900055554	0.000279993
HK2P1	0.044862647	8.783535348	1.547667981	49.84951176	0.014166164
HK2	0.020319006	1.199131424	1.046073532	1.374584222	0.009147871
HJURP	0.000696022	1.328728707	1.158829645	1.523537118	4.67E-05
HIST1H2BK	0.010103867	1.146659213	1.00713507	1.305512429	0.038699555
HIP1	0.032059997	0.813950046	0.685567963	0.966373451	0.018746922
HINT3	0.030081675	0.679764545	0.504156409	0.916540639	0.011357643
HHIPL2	0.009086	1.144933273	1.037798903	1.263127371	0.006930914
HGF	0.019481699	0.657759129	0.511410725	0.845987482	0.001104316
HERPUD1	8.21E-05	0.655993704	0.522947377	0.822889182	0.000266863
HERC1	0.032945841	0.647698062	0.482725659	0.869050093	0.003782904
HEMK1	0.009690515	0.536357447	0.357772468	0.804084542	0.002565946
HELLS	0.003451646	1.281952479	1.024848526	1.603556151	0.029639016
HEIH	0.027255323	0.714142195	0.557504416	0.9147893	0.007700477
HECTD3	0.035936097	0.625115304	0.433908032	0.90058057	0.011665558
HEATR5B	0.037217778	0.668608398	0.485707002	0.920384487	0.013558403
HDGF	0.002252136	1.277758506	1.001257556	1.630616208	0.048830885
HDC	0.021774888	0.663330489	0.491290684	0.895615063	0.007370008
HCN2	0.002798168	1.397689638	1.091041948	1.790523569	0.008062212
HCLS1	0.012827951	0.827902681	0.714240982	0.959652089	0.01219091
HCG19P	0.039949535	3885303600	2.123225179	7.10974E+18	0.042442246
HCG14	0.00361671	0.487530881	0.283284734	0.839036953	0.009499064

HCFC2	0.033460472	0.56638062	0.390547438	0.82137783	0.002722122
HCCS	0.027683992	1.388841299	1.00092894	1.927090002	0.049354076
HAVCR1	0.028125132	1.247601793	1.079371171	1.442052813	0.002758142
HAGLR	0.018729801	0.900350052	0.813185551	0.996857623	0.043326866
HAGH	0.006095981	0.607219879	0.444114274	0.830227722	0.001773654
H2AFZ	0.009628023	1.390095743	1.139677205	1.695538145	0.001153663
H2AFX	2.70E-05	1.424927758	1.183346937	1.715827416	0.000186923
GVINP1	0.015993648	0.656917648	0.483114769	0.893246955	0.007362619
GUSBP4	0.000170427	0.456170865	0.249607514	0.833676259	0.010733474
GUSBP1	0.01772559	0.626838596	0.421163939	0.932954104	0.021334653
GTSE1	7.36E-06	1.346368413	1.142885387	1.586080218	0.000374391
GTF3C6	0.012411679	1.561271811	1.14522156	2.128469943	0.004839193
GSTM5	0.038373843	0.547116354	0.350408888	0.854248609	0.007979612
GSTM2	0.04387236	0.549468546	0.41328139	0.730532973	3.78E-05
GSK3A	0.007107557	1.799144069	1.312311196	2.466579109	0.000264006
GSG2	0.032214011	1.528738506	1.175192932	1.988644892	0.001561719
GS1-54N10.1	1.08E-06	1.90E+28	5.02064E+13	7.16E+42	0.000143483
GS1-388B5.1	0.009834517	0.081729108	0.016048501	0.416216259	0.002566542
GS1-309P15.3	0.009168194	8.077575295	1.693154954	38.53588386	0.008779792
GS1-124K5.3	0.007380504	0.517449864	0.31680176	0.845179526	0.008490791
GS1-115G20.2	0.032432409	0.002447498	8.44E-06	0.709405127	0.03764892
GS1-115G20.1	0.000116962	0.132667201	0.041179754	0.427408724	0.00071433
GS1-114I9.3	0.005604764	0.19857953	0.047488745	0.830382645	0.026788064
GRPEL1	0.011815642	1.416509146	1.03098737	1.946190825	0.031694474
GRIP1	0.046056932	1.384060334	1.023861395	1.870978841	0.034576071
GRIA1	0.023780049	0.322024315	0.168723458	0.614613169	0.000590449
GREB1L	0.002403486	1.440758806	1.156555027	1.794800843	0.00112455
GRAP2	0.001205562	0.593958302	0.415786304	0.848480242	0.004197173
GRAMD2	0.031645827	0.816864975	0.698658835	0.955070419	0.011200588
GPX8	0.008351759	1.319571649	1.12587051	1.54659823	0.000617784
GPX3	0.013824281	0.832400754	0.73026389	0.948822782	0.006023577
GPSM3	0.006949632	0.823132687	0.698572051	0.96990342	0.020072179

GPRIN2	0.025086223	0.789448347	0.68389722	0.911289993	0.001244405
GPRIN1	0.003996939	1.341814514	1.122470563	1.604020854	0.001244
GPRASP1	0.048528622	0.633924322	0.44430472	0.904469451	0.011948599
GPR98	0.001762131	0.70791051	0.55572013	0.901779984	0.005156415
GPR87	0.033693036	1.123176567	1.034826077	1.219070168	0.005453762
GPR78	0.017652901	2.363843202	1.461021118	3.824554358	0.000457702
GPR75-ASB3	0.003228082	0.394673159	0.192076441	0.810963079	0.011399171
GPR75	0.011364988	0.476962842	0.273965202	0.830373895	0.008869174
GPR65	0.044639405	0.705874852	0.545628682	0.913183862	0.00801996
GPR55	0.014847518	0.280104067	0.11265004	0.696478122	0.006175997
GPR37	0.001608839	1.234464209	1.086415729	1.40268761	0.00123119
GPR34	0.014440581	0.830929928	0.710969639	0.971130844	0.019900966
GPR31	0.001741711	0.013744095	0.000837601	0.225525115	0.00267084
GPR18	0.037110876	0.630993691	0.444531878	0.895668136	0.009980401
GPR174	0.003885359	0.67622311	0.516349556	0.885597149	0.004472602
GPR155	0.037803785	0.639338504	0.433025237	0.943948962	0.024441144
GPR153	0.030037053	1.27728715	1.087021144	1.500856238	0.00294063
GPR133	0.000137622	0.770253845	0.657584157	0.902228223	0.001216085
GPR116	0.003731309	0.841316618	0.763093469	0.927558262	0.000519904
GPR115	0.00323784	1.237392593	1.089754106	1.405032952	0.001016654
GPR108	0.019426832	0.609073559	0.433363928	0.856025562	0.004301663
GPLD1	0.016482286	0.526772756	0.301607617	0.92003491	0.024265789
GPIHBP1	0.008975949	0.79966837	0.666059262	0.960078988	0.016542233
GPI	0.00525302	1.497544564	1.207903986	1.856637404	0.00023109
GPD1L	0.000770625	0.689487019	0.584525642	0.81329597	1.02E-05
GPC6	0.031998403	1.19094652	1.042391402	1.360672786	0.010148463
GPC4	0.042214422	0.832114128	0.735070181	0.941969816	0.003674162
GPC1	0.045925199	1.275144649	1.088820937	1.493352875	0.002563043
GPATCH3	0.026567964	0.582456915	0.381830789	0.888498434	0.01211823
GPAM	0.035643506	0.722572432	0.552976731	0.944182441	0.017275463
GOLM1	0.004606133	1.283927731	1.092758192	1.508540893	0.002378666
GOLGA6D	0.0007515	2.48E+25	3.50997E+11	1.76E+39	0.000325906

GOLGA2P10	0.008632115	0.837254701	0.702536525	0.99780639	0.047200291
GNPNAT1	3.27E-05	1.668820146	1.365270361	2.039860206	5.75E-07
GNMT	0.017984231	0.555619622	0.3961824	0.779219784	0.000660121
GNG7	1.09E-05	0.575059938	0.455897653	0.725368798	3.01E-06
GNAO1	0.013040342	0.652140936	0.443997258	0.957861323	0.029298027
GNAI1	0.042801099	1.195057279	1.006028971	1.419603155	0.04252089
GNA14	0.033793103	0.779762623	0.665571743	0.913545015	0.002075495
GMPPB	0.032421368	0.603149379	0.405291387	0.897599074	0.012682567
GMIP	0.015955169	0.73532995	0.567300482	0.953128283	0.020198401
GMFG	0.049147819	0.798880512	0.679333114	0.939465572	0.006627945
GMFB	0.044418626	1.382669098	1.060958477	1.801930874	0.016490709
GMDS-AS1	0.003946568	0.239166723	0.099480728	0.574992989	0.001391314
GLS2	0.005266624	0.356469987	0.176890046	0.71836067	0.003911814
GLRX2	0.005896063	1.494302782	1.124092595	1.986438497	0.005686861
GLIS2-AS1	0.002889834	0.598610824	0.41216703	0.869392485	0.007038025
GLIPR1L2	0.017119916	0.420965478	0.185235598	0.956684005	0.038857928
GLCCI1	0.002220695	0.776779666	0.65446501	0.921954025	0.003858471
GJB4	0.02944792	1.338827969	1.083682562	1.654045561	0.00683142
GJB3	4.91E-05	1.286595672	1.172188403	1.412169256	1.14E-07
GJB2	9.25E-05	1.174335483	1.077525485	1.279843351	0.000251279
GIP	0.038765442	1.399481039	1.039458467	1.884199554	0.026759549
GIMD1	1.11E-05	14192392.6	1140.825112	1.7656E+11	0.000618703
GIMAP8	0.000153563	0.767178598	0.627514345	0.937927564	0.00973838
GIMAP7	0.000496887	0.808524008	0.689132086	0.948600544	0.009127153
GIMAP6	0.002279356	0.790754814	0.660147067	0.947202838	0.010807264
GIMAP5	0.009745651	0.553746479	0.33443677	0.916870364	0.021601785
GID8	0.047600936	0.726986986	0.528987765	0.999096979	0.049352559
GHRL	0.000141879	0.430631154	0.241470193	0.767975493	0.004312078
GGTA1P	0.001901329	0.752695825	0.593143994	0.955166049	0.01942071
GGT6	0.000765578	0.820910379	0.735699655	0.915990438	0.000416676
GGT3P	0.00100747	0.385828375	0.165041333	0.901977296	0.027943894
GGT2	0.019862823	0.650892367	0.430441051	0.984248302	0.04182933

GGH	0.040149253	1.14357493	1.0226425	1.278808205	0.018643183
GGACT	0.039566388	1.770616671	1.217590506	2.574825757	0.00278576
GFRA3	0.0388013	0.92087143	0.848180711	0.999791883	0.049422896
GFM1	0.011451785	1.4772376	1.05979976	2.059097395	0.02129333
GDF10	0.005455362	0.707451543	0.574417114	0.871296613	0.001129017
GCSHP4	0.005168178	0.105708137	0.012178967	0.917500654	0.041544232
GCSAML	0.025646538	0.363878675	0.17906289	0.739447971	0.005201131
GCSAM	0.032871386	0.575133024	0.367708799	0.89956508	0.015361739
GCDH	0.018130793	0.482847927	0.33139687	0.703513346	0.000149909
GATA2	0.025445469	0.792184269	0.646599238	0.970548493	0.024544718
GAS6-AS1	0.000155265	0.592929905	0.444975122	0.790079838	0.000358714
GARS	0.043408694	1.388092109	1.098573577	1.753910474	0.006001806
GAR1	0.001816969	1.552473255	1.128110646	2.136468807	0.006936225
GAPT	0.000522872	0.631783409	0.46980258	0.849612778	0.002379584
GAPDHP73	0.016272896	7.988223079	2.186794781	29.18047387	0.001668348
GAPDHP72	0.021413612	2.581291696	1.548829715	4.302000895	0.000274015
GAPDHP68	0.033815118	3.243693547	1.070231438	9.831095832	0.037531163
GAPDHP67	0.030095232	54.21137595	6.310834965	465.6869176	0.000273795
GAPDHP65	0.011668255	1.639058899	1.256886763	2.137435253	0.000264388
GAPDHP60	0.007453149	3.169542048	2.014332911	4.987257438	6.11E-07
GAPDHP56	0.036609575	7.02E+19	548286770.4	9.00E+30	0.000461793
GAPDHP50	0.000549871	690101881	466.0225426	1.02193E+15	0.004992127
GAPDH	0.003682452	1.540112059	1.283333384	1.848268878	3.47E-06
GANC	0.029876511	0.603207003	0.431666093	0.842917003	0.003067098
GALT	0.030598149	0.630966271	0.447451159	0.889747243	0.008635629
GALNT3	0.011446065	1.205682107	1.045440964	1.390484391	0.010148633
GALNT2	0.017076871	1.801034456	1.39790199	2.320423846	5.34E-06
GALM	0.02582773	0.775693572	0.633036771	0.950498526	0.014302184
GAL	0.018910632	1.110914349	1.00196008	1.231716427	0.045809532
GADD45G	0.007013711	0.834531168	0.731631109	0.951903578	0.007057579
GAB3	0.000255432	0.598029957	0.446706628	0.800614559	0.000552478
FYN	0.049419978	0.811923559	0.665774867	0.990154327	0.039620574

FYCO1	0.004046732	0.707307087	0.56165761	0.890726498	0.003244034
FXYD6	0.043459978	0.797680574	0.640733211	0.993072137	0.043160403
FXYD1	0.042849616	0.504099876	0.299234491	0.849222576	0.010048788
FUT4	0.000201442	1.823663168	1.419483297	2.342928131	2.60E-06
FUT1	0.000717887	0.614188394	0.465337376	0.810653523	0.000576645
FURIN	0.012487036	1.226733497	1.094012322	1.375555871	0.000468808
FUCA2	0.001053578	1.414776666	1.114651633	1.795711732	0.004341627
FUCA1	0.018101883	0.697457644	0.574214114	0.847152923	0.000281248
FUBP3	0.009530341	1.536100905	1.043240975	2.261803405	0.029674909
FTO-IT1	0.000986355	0.480789388	0.298837874	0.773524562	0.00254116
FTH1P26	0.001469113	58.55121259	4.514527012	759.3806586	0.001853227
FSIP2	0.026909293	2.679473563	1.675970297	4.28383402	3.84E-05
FSCN1P1	0.003334106	103.5447482	11.32079522	947.0637591	3.98E-05
FSCN1	0.002628796	1.272965405	1.136732038	1.425525866	2.93E-05
FRZB	0.011085932	0.780133065	0.667220967	0.912152989	0.001854188
FRS3	0.030777231	0.650660542	0.485314099	0.872340493	0.004066453
FRMPD2	0.016190052	0.388069215	0.164712337	0.914307445	0.030398624
FRMD8	0.009517718	1.398363037	1.007832293	1.940222789	0.044786451
FOXO1B	0.001331295	54276.75712	305.7763055	9634384.057	3.70E-05
FOXM1	0.001110135	1.284586442	1.136168491	1.452392264	6.39E-05
FOSL2	0.004293349	1.300414245	1.085132524	1.558406159	0.004444322
FOSL1	0.000378071	1.245170982	1.131882477	1.369798372	6.63E-06
FOLR2	0.002037688	0.87533	0.77871709	0.983929362	0.025649883
FOLR1	0.019817587	0.917903959	0.856238423	0.984010591	0.015768204
FOCAD-AS1	0.009368269	0.253298233	0.091321666	0.702571448	0.008335685
FNIP2	0.027650755	0.772220333	0.647120483	0.921504199	0.004150071
FNDC3B	0.009602481	1.272886276	1.004294833	1.613310573	0.045998669
FMNL3	0.008532601	0.757748303	0.579754614	0.990388825	0.042289472
FLT3	0.022354791	0.452133471	0.246712821	0.828593644	0.010218795
FLJ27354	0.015588551	0.405548488	0.186927585	0.879857173	0.022379512
FLI1	0.009818162	0.777658772	0.613065627	0.986441156	0.038221262
FKSG48	0.006515677	0.716617716	0.525405218	0.977418826	0.035361365

FKBP9	0.019983142	1.385554466	1.094330939	1.754278446	0.006754077
FKBP5	0.019949142	1.176844259	1.021851562	1.355345983	0.02382306
FKBP4	0.000656198	1.512177196	1.263554203	1.809720443	6.41E-06
FKBP3	0.001997754	1.311830409	1.053608558	1.633338121	0.015228882
FITM1	0.00574951	0.43496618	0.196576775	0.962451325	0.039935921
FIGF	0.040238037	0.752167139	0.644333624	0.878047311	0.000309471
FIBCD1	0.006584831	1.260525232	1.073484467	1.480155426	0.004724397
FHL5	0.029907379	0.737579758	0.575626397	0.945098942	0.016111985
FHL2	0.027247781	1.149322476	1.009566233	1.308425452	0.035387987
FHL1	0.028304554	0.836997518	0.727103824	0.963500427	0.013222234
FGR	0.019909661	0.838694697	0.713627071	0.985681209	0.032760368
FGFBP1	0.004320316	1.103333354	1.019303506	1.194290497	0.014973544
FGF14	0.049884864	0.522076535	0.279634782	0.974713897	0.041313912
FGF12-AS2	0.020875239	1.639022132	1.082264132	2.482197709	0.019633113
FGD3	0.016477476	0.67589268	0.533041899	0.857026278	0.001222558
FGD2	0.002328886	0.691489732	0.53325222	0.896682717	0.005394107
FFAR4	0.023804131	0.698896131	0.509443652	0.958802411	0.026367872
FENDRR	0.0248101	0.627590132	0.447945319	0.87928003	0.00677486
FDCSP	0.002296925	0.879861381	0.804438217	0.96235613	0.005124235
FCRLB	0.010681331	1.525612083	1.07034537	2.174524499	0.019495876
FCRLA	0.002783665	0.753182082	0.623219328	0.910246558	0.003356281
FCRL6	0.013648582	0.592593323	0.4351357	0.807028352	0.000898488
FCRL3	0.001592553	0.661557703	0.485099157	0.902204401	0.009050949
FCRL2	0.001674084	0.747246029	0.578153019	0.965793847	0.026023793
FCRL1	0.000468874	0.555016968	0.385153794	0.799794366	0.001586356
FCHSD2	0.017554359	0.626772888	0.459723916	0.854522115	0.003136116
FCHSD1	0.039665236	0.728375464	0.555524264	0.95500926	0.021847039
FCGRT	0.001430341	0.688742608	0.56535989	0.839052059	0.000213682
FCF1P7	0.007923648	0.346220337	0.154519677	0.775749239	0.009970145
FCER2	0.040935092	0.666460082	0.494843908	0.897594238	0.007559039
FCER1A	0.018100223	0.868218787	0.778653347	0.968086588	0.010964752
FBXO9	0.000124099	0.592808775	0.441385269	0.796180273	0.000511704

FBXO45	0.000480567	1.528633119	1.199389101	1.948257835	0.000605681
FBXO44	0.018403754	0.722320405	0.574218525	0.908620577	0.00546116
FBXO36P1	0.046649731	0.56694456	0.333579361	0.963567211	0.035983737
FBXO33	0.02305977	0.708204286	0.508260764	0.986803125	0.041504711
FBXO31	0.007993331	0.6311958	0.439479433	0.906545581	0.01273332
FBXL8	0.041097829	0.796960451	0.637633725	0.996098443	0.046117552
FBP1	0.000715121	0.778089248	0.681207325	0.888749805	0.00021703
FBLN5	0.006211839	0.842754242	0.720859452	0.98526101	0.031853444
FAT1	0.023828281	1.175743581	1.014667512	1.362390095	0.031266913
FASTKD3	0.046979064	0.720066284	0.538298304	0.963212126	0.026933983
FARSB	0.009960352	1.554019129	1.139123862	2.120028851	0.005402954
FARS2	0.012746652	0.699967035	0.49046564	0.998956524	0.049331303
FANCL	0.013400582	1.376506125	1.0352865	1.830188176	0.027909447
FANCI	0.005103834	1.383204154	1.150918743	1.662370819	0.000543197
FANCD2	0.004867885	1.427629331	1.095219124	1.860929435	0.008475476
FAM90A24P	0.00442228	7.00E-27	6.90E-48	7.09E-06	0.014670388
FAM90A12P	0.008400344	7.10E-19	2.44E-36	0.206278285	0.041659618
FAM83H	0.011873764	1.233418845	1.030848347	1.475796174	0.021912725
FAM83D	0.000101106	1.256191448	1.093359419	1.443273754	0.001281699
FAM83A-AS1	0.000804915	1.391282206	1.223087068	1.582607016	5.08E-07
FAM83A	0.001319557	1.284396429	1.168233573	1.412109895	2.28E-07
FAM78A	0.012727177	0.727287122	0.568214102	0.930893049	0.011451544
FAM76A	0.005980013	0.473880181	0.320320358	0.70105574	0.000185907
FAM72D	0.00012144	1.676398529	1.085976177	2.587821067	0.019684931
FAM72C	0.000145632	1.838119592	1.069792626	3.158260348	0.027506519
FAM72B	7.52E-05	1.80220983	1.257479755	2.582912576	0.001338254
FAM72A	0.010304863	2.479528131	1.521059441	4.041958906	0.000270362
FAM65B	0.021601924	0.738127569	0.597832068	0.911346744	0.004756727
FAM64A	0.003535517	1.26617469	1.086269015	1.475875979	0.002542333
FAM53B	0.033561779	0.762126599	0.589435751	0.985411814	0.038259775
FAM47E	0.004154114	0.372167514	0.171634158	0.806999374	0.012315392
FAM41C	0.014428	0.64339153	0.454122071	0.911544908	0.013101592

FAM32B	0.040411391	0.176013951	0.038287872	0.809157302	0.025611763
FAM25BP	7.81E-05	24.7553785	1.73695353	352.8181693	0.017920131
FAM21FP	0.001391235	0.142982984	0.042664002	0.479189318	0.001620399
FAM21EP	0.001305201	0.072266129	0.013931379	0.374865505	0.001759062
FAM21C	0.003205287	0.563269015	0.390011292	0.813494351	0.002209042
FAM219B	0.022114948	0.589173727	0.397951457	0.872281468	0.008229983
FAM217B	0.036889214	0.717412065	0.524977931	0.980384205	0.037133201
FAM215A	0.001755792	0.092828299	0.015610198	0.552016892	0.008970499
FAM214A	0.003672208	0.663259014	0.499011287	0.881568276	0.004680344
FAM210B	0.014868637	0.639464371	0.501986273	0.814593353	0.000294146
FAM20A	0.035321088	0.855651774	0.739943614	0.989453717	0.035466688
FAM207BP	0.002939789	3.620137194	1.809910439	7.240907075	0.000275488
FAM207A	0.006584288	1.677715611	1.27804707	2.202367766	0.000193677
FAM189B	0.048522987	1.385329006	1.056772577	1.816035441	0.018287444
FAM189A2	0.002644424	0.713419534	0.599696253	0.848708707	0.000138131
FAM184A	0.011425855	0.667996811	0.535572903	0.833163397	0.000344773
FAM183B	0.018391877	0.412289288	0.175266397	0.969851947	0.042346078
FAM179A	0.047842922	0.436678114	0.220920486	0.863151165	0.017159535
FAM177B	0.017136554	1.14522908	1.005950899	1.303790919	0.04039915
FAM174B	0.013370607	0.845355698	0.71477446	0.999792658	0.049717538
FAM159A	0.008803184	0.561586839	0.332803111	0.947646722	0.030664497
FAM13B	0.005851799	0.6001693	0.448499263	0.803129945	0.000592441
FAM131C	0.007007415	1.698042961	1.18532941	2.432530461	0.003889131
FAM129C	0.000818376	0.534101738	0.327807119	0.870221085	0.011799639
FAM129A	0.008783242	0.856453441	0.737727304	0.994286768	0.041828939
FAM122C	0.002108427	0.45450761	0.229409543	0.900473297	0.023790768
FAM117A	8.03E-05	0.504728472	0.389241609	0.654479954	2.50E-07
FAM111B	0.001351116	1.298844763	1.106118614	1.525150826	0.00141935
FAM107A	0.043649108	0.833165926	0.70707126	0.981747525	0.029259044
FAM104B	0.014037314	0.690407246	0.500859586	0.951688214	0.023675681
FAM101A	0.008259795	1.2040027	1.050075926	1.380493035	0.007812136
FAIM2	0.025127639	0.647552433	0.501841381	0.835571098	0.000834302

FADD	0.000134325	1.753076013	1.277772472	2.405182124	0.000503156
FAAH	0.002680337	0.68516452	0.564724029	0.831291738	0.00012645
FA2H	0.021030993	1.14271226	1.021273523	1.278591171	0.0199558
F2	0.006642091	1.314167815	1.120091898	1.541870848	0.000805143
EZH1	0.009758745	0.654833327	0.479621929	0.89405146	0.007701462
EXT1	2.82E-05	1.5536128	1.20855615	1.997187083	0.000585595
EXO1	0.003641936	1.419279618	1.213129347	1.660461549	1.23E-05
EVL	0.021980392	0.79008575	0.627294877	0.995122893	0.045339022
EVI2B	0.005778388	0.845295599	0.730622495	0.977966945	0.023853307
ETV5	0.018175199	0.805168508	0.670347824	0.967104393	0.020465143
ETHE1	0.021922931	1.307590718	1.075372784	1.58995421	0.007179267
ESYT3	0.000296492	0.653731225	0.529161454	0.807625937	8.12E-05
ESX1	0.001581641	1.695228217	1.013477302	2.835582705	0.044328712
ESPL1	0.000353859	1.279716478	1.076166847	1.521766137	0.005263057
ESCO2	0.018928898	1.603460876	1.188900909	2.162574493	0.001976974
ERVFRD-1	0.004842983	0.062618784	0.009875816	0.397041848	0.003280162
ERRFI1	0.034694845	1.203949177	1.057155932	1.371125655	0.005145246
ERO1LB	3.53E-05	0.756666098	0.65120946	0.879200347	0.000271422
ERO1L	0.000462341	1.506572268	1.288528244	1.761513578	2.78E-07
ERMN	0.020863237	0.47624862	0.238679187	0.950282891	0.0353221
ERLIN1	0.023701851	1.623234329	1.244973889	2.116421646	0.000345327
ERG	0.019994702	0.714712263	0.54961757	0.929398271	0.012199505
ERF	0.041890633	1.464028242	1.123748897	1.907346649	0.004736485
EREG	0.00040104	1.088772102	1.007441175	1.17666889	0.031783501
ERCC6L	0.00791159	1.471603285	1.155029572	1.8749444	0.0017711
EPOR	0.016172593	0.786857935	0.626846495	0.987714559	0.038778133
EPN1	0.022134058	1.782826473	1.283411121	2.476579936	0.00056496
EPM2AIP1	0.023753449	0.622877671	0.439762199	0.882241799	0.007690431
EPHX1	0.010269204	0.797880482	0.707424564	0.899902682	0.000235168
EPHB2	0.01393531	1.204124064	1.028560854	1.409653845	0.020877283
EPHA2	0.027839187	1.190558898	1.043043277	1.358937371	0.009755373
EPGN	0.041744282	1.401685586	1.154615524	1.701624863	0.000642234

EPG5	0.048194474	0.669558721	0.491962902	0.911265623	0.010745931
EPC1	0.003375733	0.422697653	0.259272639	0.689132902	0.000554497
EPB41L5	0.018649665	0.724095984	0.580226117	0.903639081	0.004283055
EPB41	0.003332097	0.707277931	0.555594504	0.900372605	0.004922002
ENTPD7	0.042310456	1.249549638	1.001496263	1.559041562	0.04847203
ENTPD1	0.004943527	0.699587486	0.526235746	0.930044479	0.013926205
ENPP7P6	0.015892866	0.000339706	7.91E-07	0.145845353	0.009811611
ENPP7P14	0.003355918	0.001110719	1.45E-06	0.850165232	0.044656562
ENPP5	0.007208311	0.704497391	0.598546838	0.829202565	2.53E-05
ENPP4	0.005137231	0.74898803	0.619599439	0.905396347	0.002816864
ENPP1	0.00052345	1.341826078	1.081479733	1.664846014	0.007546589
ENO1	0.034012905	1.373489353	1.064067397	1.772888643	0.014818491
EMR4P	0.020034169	0.491096307	0.30951687	0.779200121	0.002534185
EMR3	0.00169605	0.373261697	0.207054831	0.672885987	0.001046814
EMP2	0.012640035	0.837992466	0.718353612	0.977556682	0.024527349
EMID1	0.008774337	0.783860309	0.625350032	0.982548898	0.03462665
EMC6	0.048713204	1.5626665	1.197957617	2.038408165	0.00099501
ELOVL6	0.00958145	1.257804149	1.079400773	1.465694038	0.003292844
ELMO2	0.02192698	0.648413604	0.464369851	0.905399437	0.010977875
ELMO1	0.003851411	0.77020752	0.613011565	0.967713592	0.02497775
ELF5	0.010009851	0.785113125	0.677256975	0.910145841	0.001333461
ELF2	0.042899863	0.610151436	0.383251571	0.971384862	0.037312216
ELDR	0.039798403	8.724082597	3.060047388	24.87203873	5.07E-05
EIF6	0.026414342	1.517521655	1.13352869	2.031595667	0.00507896
EIF5A	0.000366319	1.524015067	1.208315579	1.922198112	0.000374051
EIF4H	0.002154257	1.575700265	1.121516068	2.213816988	0.008766695
EIF4G1	0.00316747	1.494420114	1.155221076	1.933215662	0.002224702
EIF4A3	0.035662133	1.339538416	1.037867442	1.728894361	0.024738172
EIF3M	0.004710754	1.672377655	1.159823899	2.411441104	0.005887155
EIF3KP2	0.015229088	0.00384518	4.32E-05	0.342320285	0.015181236
EIF3H	0.020995753	1.319907517	1.012209329	1.721141865	0.040405988
EIF3B	0.006892698	1.546105013	1.196868344	1.997246164	0.000850948

EIF2S1	0.006522829	1.65545382	1.219262314	2.247692987	0.001235908
EHBP1	0.00784237	1.57760741	1.240551406	2.006241038	0.000201031
EGLN3	0.009024267	1.165366238	1.056271121	1.285729054	0.002276209
EGLN1	0.002672033	1.467656816	1.173209669	1.836003049	0.00078461
EFNA5	0.01853873	1.202949266	1.036297798	1.396400667	0.015157852
EFNA2	0.001334319	1.263019711	1.000571555	1.594307554	0.049440654
EFCC1	0.000964861	0.660921226	0.5119136	0.853301939	0.001487963
EFCAB6	0.005572723	0.516295972	0.283938753	0.938799399	0.030236489
EEF2K	0.01433443	0.712686905	0.535580858	0.948358436	0.020140298
EEF1E1-BLOC1S5	0.000283304	76556656.21	6051.073856	9.68575E+11	0.000165298
EDA2R	0.025174629	0.744763608	0.588804046	0.942032983	0.013968999
ECT2L	0.005353679	0.641534641	0.455828298	0.902898519	0.010903398
ECT2	0.002446829	1.415865748	1.214438723	1.650701495	8.94E-06
ECEL1P2	0.003139724	0.767210834	0.601088562	0.979244159	0.0333015
ECE2	0.024829003	1.271083468	1.017293218	1.588188297	0.034785835
EBLN3	0.010205264	0.689329008	0.519794899	0.914157645	0.009790727
EAF2	0.007343178	0.669004474	0.502756601	0.890225977	0.005820734
EAF1	0.033558638	1.506634048	1.040665309	2.181245147	0.029923507
E2F8	0.020649681	1.263560925	1.039861756	1.535383144	0.018615154
E2F7	0.000153309	1.712765464	1.375275653	2.133074579	1.54E-06
E2F6P3	0.007998106	3.15E-10	1.94E-17	0.00512807	0.009811724
DYNLL1	0.020845282	1.449033607	1.055825109	1.988680109	0.021659652
DXO	0.011537875	0.745562171	0.573018879	0.970060448	0.028793476
DUTP8	0.041055673	5.70E-05	4.16E-09	0.779194503	0.04429275
DUT	0.002841514	1.562836146	1.152155884	2.119901356	0.004097954
DUSP26	0.005107525	0.515995776	0.31204654	0.853243368	0.009924376
DUSP22	0.049206945	0.664149846	0.475063147	0.928497656	0.01666893
DUSP2	0.004080004	0.80494374	0.678213584	0.955354537	0.013046137
DTYMK	0.00100326	1.361395764	1.104525531	1.678004152	0.003829857
DTNBP1	0.00559632	0.43292589	0.288071948	0.650618109	5.62E-05
DTL	0.002545945	1.443898101	1.202074674	1.734369561	8.57E-05
DSP	0.043365352	1.12255188	1.012121033	1.245031652	0.028670042

DSG2-AS1	0.011197073	2.238310762	1.068277945	4.689823555	0.032762986
DSG2	0.006119971	1.379516219	1.184831597	1.606190285	3.40E-05
DSCC1	0.011022272	1.280804289	1.078816139	1.520610943	0.004708295
DSC2	0.000742194	1.290858126	1.096385536	1.519825505	0.002180525
DSC1	0.024323646	7.783117466	1.698574088	35.66339434	0.0082387
DRP2	8.42E-05	1.760937249	1.153427059	2.68842314	0.008762946
DRAXINP1	0.000875541	2.395496884	1.53080728	3.748613817	0.000131499
DRAM1	0.001556136	0.818104642	0.716463613	0.934164965	0.003015942
DPYSL2	0.009173965	0.742423181	0.635405919	0.86746466	0.000176654
DPY19L1	0.004610636	1.237815374	1.055906479	1.451063072	0.008519522
DPRXP6	0.034699393	0.008575425	0.000142866	0.514732529	0.022736029
DPPA3P2	0.000195629	0.461160541	0.261579997	0.813017232	0.007461587
DPEP2	0.002566269	0.655349627	0.507199546	0.846773497	0.001229141
DOPEY1	0.013981976	0.524857261	0.370189765	0.74414576	0.000295684
DOK1	0.019368691	0.655897783	0.507583515	0.847549003	0.001261324
DOCK9-AS2	0.006549669	0.704310568	0.538348988	0.921434584	0.010564686
DOCK8	0.018108978	0.78276115	0.640803005	0.956167516	0.016440749
DOCK4	0.009674565	0.699727827	0.538754619	0.908797835	0.007430086
DOCK2	0.024973546	0.790074637	0.64874799	0.962188619	0.019114701
DNTTIP2	0.021374347	1.40616342	1.030997914	1.917846328	0.031337475
DNM1P49	0.002700174	422616400	1769.752176	1.00921E+14	0.001668668
DNM1P46	0.003596053	0.028561142	0.001454131	0.560980161	0.019259719
DNER	0.037995218	1.242285549	1.112054134	1.387768219	0.000123202
DND1P1	0.018195055	0.738158214	0.55841826	0.975751669	0.032975951
DNASE2B	0.005988642	0.612699642	0.421628862	0.890358525	0.010200359
DNASE1L3	0.000135662	0.690511449	0.532763493	0.894967592	0.005133176
DNALI1	0.000103452	0.773222867	0.680623435	0.878420535	7.76E-05
DNAJC9	0.000576938	1.488601851	1.116618692	1.984505082	0.006690707
DNAJC28	0.00208447	0.428953373	0.256699877	0.716794251	0.001233573
DNAJC27	0.007133426	0.431958202	0.263174759	0.70898854	0.000899195
DNAJC22	0.043862284	1.177364124	1.018073581	1.361577696	0.027702522
DNAJC18	0.044169274	0.537027217	0.340427422	0.847165101	0.007515472

DNAJB4	0.005091043	1.393907415	1.162334325	1.671617055	0.000339929
DNAH9	0.027426584	0.680565005	0.493843887	0.937884903	0.018678853
DNAH8	0.024932059	0.003357803	2.75E-05	0.410493356	0.020175066
DNAH1	0.004835304	0.63187506	0.455715905	0.876129377	0.005904808
DMTN	0.027634664	0.825211446	0.699027466	0.974173354	0.023268316
DMAP1	0.023203979	0.686713987	0.524037639	0.899889751	0.006436484
DLGAP5	0.000113508	1.351676794	1.178544379	1.550242985	1.64E-05
DLGAP4-AS1	0.037770155	0.339711722	0.131549592	0.87726653	0.025715303
DLG4	0.001248618	0.727383309	0.548280758	0.964991877	0.02731016
DKK1	4.58E-06	1.239178951	1.156109002	1.328217728	1.38E-09
DKFZP434K028	0.00198792	7.649610401	2.320181451	25.2206737	0.000829731
DIP2B	0.010609264	1.423176446	1.083962208	1.868544107	0.011074443
DIDO1	0.003753103	0.707575471	0.516630603	0.969092896	0.031114418
DIAPH3	0.001043717	1.563352792	1.236716219	1.976259319	0.00018647
DHRS1	0.041877571	0.67873797	0.505796074	0.910812195	0.009807945
DHDH	0.027634643	0.757273606	0.621461687	0.922765353	0.00583271
DFFB	0.026539893	0.636768218	0.416756997	0.97292611	0.036899964
DERL1	0.016737791	1.476644221	1.069223497	2.039309988	0.017966223
DERA	0.000889812	1.438195572	1.137849929	1.817820127	0.002361978
DEPDC1B	0.000364429	1.356199879	1.150425124	1.598781246	0.000284491
DEPDC1	0.004763153	1.324434992	1.124086315	1.560492308	0.000785803
DENR	0.004960234	1.532283792	1.131494959	2.075036747	0.005806632
DENND1C	0.000574151	0.614974746	0.474843797	0.796459679	0.000228842
DEFT1P	0.000781506	38169159767	357.70207	4.0729E+18	0.009784078
DEFB113	0.002795716	826168.8833	10.80015264	63198646039	0.017562591
DEFB110	0.000300178	650160304.7	74.02352757	5.71046E+15	0.012859906
DEFB106B	0.006999339	1532011666	10.83717374	2.16575E+17	0.027186006
DEFB1	0.022701426	1.075498232	1.002734374	1.153542232	0.041713988
DEF6	0.006063693	0.740516226	0.594754241	0.922001463	0.007229422
DEDD2	0.029346858	1.467522992	1.079718281	1.994616348	0.01429217
DDX52	0.032832116	1.651260332	1.153762372	2.363277527	0.006108606
DDX24	0.006518014	0.693048127	0.497646551	0.965174391	0.030027912

DDX23	0.037151451	1.484053386	1.056497933	2.084636782	0.022788959
DDIT4	0.008468696	1.213711587	1.07204438	1.374099657	0.002224228
DDIAS	0.015963877	1.32623935	1.056028004	1.66559107	0.015143259
DCUN1D5	0.038615617	1.293286906	1.019703037	1.640272667	0.033933343
DCUN1D1	0.003764309	1.651331693	1.215723147	2.243024134	0.001326704
DCLK3	0.017507113	4.887816612	1.36203145	17.54052832	0.014936995
DCDC2B	0.045355459	0.702206418	0.503004684	0.980296744	0.037813537
DCBLD2	0.047801949	1.162242023	1.025022058	1.31783166	0.01900105
DCAF11	0.04418631	0.645246791	0.445405802	0.934750781	0.020516248
DBP	0.005207531	0.610246477	0.444103841	0.83854434	0.002319597
DBIP2	0.025327051	0.041195004	0.002036265	0.833402631	0.037641331
DARS2	0.004236744	1.381187789	1.111040534	1.717020802	0.003635279
DARS	0.000605053	1.55791486	1.124199165	2.158957939	0.007739801
DAPK2	0.004992972	0.622021739	0.477456046	0.8103595	0.000434621
DAK	0.048428242	1.334079843	1.002855742	1.774700939	0.047754306
DAAM2	0.00330063	0.741217055	0.63574213	0.86419115	0.000131483
CYTH4	0.012121286	0.798855439	0.658271741	0.969462872	0.022966276
CYP4Z2P	0.010323008	0.676103484	0.499751341	0.914686733	0.011140988
CYP4Z1	0.000500781	0.624412735	0.396018576	0.984527716	0.042654039
CYP4B1	0.000850729	0.860241229	0.80127466	0.923547204	3.25E-05
CYP4A26P	0.000491978	0.231778599	0.070514773	0.76184488	0.016040578
CYP2U1	0.02126616	0.624407547	0.457815198	0.851620449	0.002936219
CYP2B7P	0.007386896	0.882281055	0.820171237	0.949094316	0.000771605
CYP27A1	0.006225966	0.799586553	0.69525974	0.919568067	0.001715844
CYP17A1-AS1	0.006514682	0.089553516	0.009388919	0.854180579	0.03599919
CYP17A1	3.79E-05	0.181104794	0.064257149	0.510432642	0.001229241
CYLD	0.01487111	0.666656154	0.49643933	0.895236135	0.007024076
CYFIP2	0.019350481	0.808511809	0.681252394	0.959543556	0.014991586
CYCS	0.000678607	1.410693187	1.139902451	1.745811904	0.001555754
CYB5RL	0.035948345	0.542657492	0.311334922	0.945853269	0.031057317
CYB5A	0.017896263	0.847791437	0.733712337	0.979607789	0.025130929
CXorf40A	0.034887268	0.592564374	0.391714365	0.896399441	0.013218313

CXorf21	3.74E-05	0.68033845	0.547146333	0.845953594	0.000530453
CXCL17	0.004928741	0.858812937	0.802850352	0.918676386	9.55E-06
CX3CR1	0.019546219	0.712920201	0.579203277	0.87750749	0.00140834
CUTA	0.025780411	0.723425292	0.538285349	0.972242982	0.031824907
CUL9	0.015374723	0.663599147	0.493352199	0.892595247	0.006704871
CTSV	0.000713045	1.272875667	1.119842629	1.446821565	0.000222584
CTSLP7	0.014124688	0.546982802	0.32999586	0.906648303	0.019280531
CTSLP2	0.003554626	0.224123565	0.066159069	0.759251494	0.016288409
CTSL	0.013481884	1.288565709	1.124984513	1.47593284	0.000252028
CTSH	0.014136377	0.797222352	0.709852616	0.895345688	0.000129958
CTSG	0.000848849	0.72722988	0.581021832	0.910229649	0.005414274
CTF1	0.000284405	0.765252197	0.6455813	0.907106393	0.002044776
CTD-3214H19.6	0.015176388	0.593703745	0.438876848	0.803150446	0.000719902
CTD-3214H19.4	0.01936735	8.50E-05	3.09E-08	0.23395778	0.020370311
CTD-3184A7.4	0.034432821	0.756535459	0.633388625	0.903625165	0.002084426
CTD-3179P9.1	0.001780317	0.003630404	0.000147123	0.08958359	0.000592647
CTD-3094K11.1	0.023866068	0.728915769	0.542976131	0.978529567	0.035342318
CTD-3032H12.2	9.97E-05	0.589141205	0.425370088	0.815965601	0.001453363
CTD-3032H12.1	0.000876467	0.64639881	0.433122432	0.964695872	0.032687226
CTD-3001H11.1	0.024437599	0.223319483	0.057684712	0.864554745	0.029953869
CTD-2655K5.1	0.04628969	0.511016588	0.280828309	0.92988472	0.02795189
CTD-2623N2.11	0.035036815	0.135101552	0.032344004	0.56432188	0.006063106
CTD-2589M5.4	0.008555672	0.844094402	0.746124752	0.954927924	0.007088699
CTD-2587H24.5	0.018977732	3.249979371	1.027406532	10.28061004	0.044858348
CTD-2583P5.3	0.013313451	0.047011703	0.006605807	0.334569271	0.002261964
CTD-2576F9.1	0.024316509	2.169770575	1.004947655	4.684725939	0.048548923
CTD-2568A17.1	0.025059248	3.749285254	1.391051068	10.10540895	0.008990607
CTD-2562J17.4	0.035748306	0.02760419	0.001044666	0.729411609	0.031647789
CTD-2561J22.2	0.048051956	0.507910966	0.316017912	0.816325722	0.005138506
CTD-2555C10.3	0.011884476	1.896211726	1.489585139	2.413839139	2.04E-07
CTD-2552B11.3	0.000781506	3.56E+38	2009593612	6.32E+67	0.009784078
CTD-2542C24.3	0.028811502	0.504316207	0.270879981	0.938920756	0.030872795

CTD-2540F13.2	0.016022965	0.788389052	0.626650706	0.991872015	0.042394088
CTD-2537L20.1	0.007738536	1091237.726	102.2650989	11644244106	0.003305284
CTD-2531D15.4	0.038630031	0.439110999	0.244495419	0.788638372	0.005873795
CTD-2524L6.3	0.024751528	0.160654703	0.050732069	0.508749873	0.00187695
CTD-2517O10.6	0.00317105	0.665186836	0.500313229	0.884393017	0.005026496
CTD-2516F10.2	0.008913005	0.447888222	0.230739793	0.869394296	0.017617902
CTD-2515H24.1	0.016365945	0.018613644	0.000504894	0.686218582	0.030421339
CTD-2514K5.4	0.006450302	0.014066652	0.000357985	0.552734255	0.022816208
CTD-2510F5.4	0.010701261	1.280548358	1.117334369	1.467603738	0.000378206
CTD-2385L22.2	0.043230755	468183.1507	3.38068177	64837650378	0.030646986
CTD-2377D24.6	0.019617891	1.347636195	1.024702009	1.772342883	0.032798125
CTD-2376I20.1	0.022343524	0.069783316	0.00615764	0.790840474	0.031601755
CTD-2373N4.3	0.028643217	0.72836415	0.57123045	0.928722086	0.010577145
CTD-2370N5.3	0.039171642	0.101154745	0.015842858	0.645860906	0.015429487
CTD-2357A8.3	0.002017792	1.343095509	1.123186145	1.606061073	0.001223553
CTD-2353F22.1	0.01806704	0.525128182	0.318505839	0.865791372	0.01157417
CTD-2342J14.6	0.046656801	0.550862294	0.305110997	0.994553687	0.04791956
CTD-2340D6.1	0.016294983	0.001504989	1.10E-05	0.205168882	0.009553595
CTD-2318H23.1	0.031921335	118.0180941	1.383352301	10068.49125	0.035464937
CTD-2316B1.2	7.38E-05	312.921763	18.42536676	5314.414147	7.00E-05
CTD-2313J17.6	0.00287869	0.487864898	0.274909381	0.865784054	0.014190013
CTD-2313J17.3	0.027288801	0.087053574	0.008304152	0.912594597	0.041724797
CTD-2310F14.1	0.045745755	0.240159501	0.085589928	0.673871185	0.006732435
CTD-2308B18.2	0	9.9442E+13	391114410.2	2.52834E+19	3.86E-07
CTD-2292P10.4	0.005048212	2.437688316	1.124865693	5.282696734	0.023936031
CTD-2290C23.2	0.009245474	0.004347651	0.000193488	0.09769098	0.000615291
CTD-2280E9.1	0.03164452	0.676575149	0.511619092	0.894716282	0.006140443
CTD-2272G21.3	0.009787828	5.21441E+13	4141.927061	6.56E+23	0.007770025
CTD-2267D19.1	0.049795543	0.398858669	0.190857654	0.833543924	0.014521301
CTD-2240J17.1	0.040064505	0.118673144	0.015073329	0.93432014	0.04291944
CTD-2232E5.2	0.040074754	0.283888344	0.083458082	0.965665512	0.043810617
CTD-2215E18.1	0.008127183	0.002074704	7.14E-05	0.060286851	0.000325905

CTD-2207O23.3	0.025494422	0.000895873	1.71E-06	0.467991279	0.027966354
CTD-2194D22.4	0.002080773	1672453.919	989.36949	2827156223	0.000157664
CTD-2192J16.20	0.027528753	0.07960207	0.014013418	0.452173006	0.004296629
CTD-2186M15.3	0.01284766	0.645587236	0.498100152	0.836745136	0.000943405
CTD-2168K21.1	2.80E-05	367.583678	13.64921096	9899.309251	0.000438965
CTD-2124B20.2	0.000259991	5.24729E+12	62473.10798	4.41E+20	0.001654522
CTD-2066L21.3	0.002239935	1.673527457	1.225358885	2.285611328	0.001204152
CTD-2066L21.2	0.007333993	1.886215046	1.492326535	2.384067507	1.10E-07
CTD-2066L21.1	8.54E-06	4.385905698	2.535657442	7.586264799	1.24E-07
CTD-2062O1.1	0.044583158	667987.6574	3.40095981	1.312E+11	0.031020752
CTD-2026K11.5	0.010952278	0.411617141	0.213053596	0.795239667	0.008245649
CTD-2026K11.4	0.040511078	0.231000379	0.075220618	0.709395594	0.010475244
CTD-2026G6.3	0.000607015	0.443452346	0.218770461	0.898887271	0.024092073
CTD-2017D11.2	0.002562894	0.594264556	0.430355202	0.820602053	0.001573499
CTD-2017D11.1	0.007418306	0.729113996	0.573920865	0.926272681	0.009677674
CTD-2006C1.2	0.001888258	0.299215081	0.12934478	0.692178414	0.004805981
CTD-2003D5.1	0.008527758	3.102299623	1.146955158	8.391141431	0.025744948
CTC-559E9.9	0.000344009	1.26E-05	9.37E-09	0.016893602	0.002137164
CTC-559E9.6	0.000264193	0.37899043	0.234317525	0.612987637	7.66E-05
CTC-559E9.5	0.000103822	0.568877925	0.428658364	0.754965073	9.36E-05
CTC-559E9.4	0.004046622	0.193561242	0.063241597	0.592425807	0.004011644
CTC-559E9.1	0.002525291	0.445910769	0.251271255	0.79132177	0.005785029
CTC-546K23.2	0.021895319	15746.18734	181.871412	1363284.164	2.18E-05
CTC-510F12.3	0.021268793	0.47356191	0.231605395	0.968288679	0.04053366
CTC-508F8.1	0.002350049	0.168473114	0.032114671	0.883807597	0.035202394
CTC-499B15.1	0.033875329	2.50083E+12	21.77396667	2.87E+23	0.028016207
CTC-497E21.3	0.007988692	0.608925378	0.439066923	0.844495673	0.002950297
CTC-471J1.10	0.04213362	0.421038619	0.194207023	0.912806942	0.028448651
CTC-463N11.1	0.01523746	5.61E-13	1.13E-23	0.02779131	0.024759082
CTC-458I2.2	0.009798181	0.367540623	0.16823615	0.802955306	0.012060334
CTC-457L16.2	0.004085159	0.007958737	0.000160313	0.395112294	0.015264441
CTC-457L16.1	0.019651491	0.000756043	4.77E-06	0.119921284	0.00542856

CTC-457E21.4	0.048073739	316471705.2	2.49650189	4.01179E+16	0.039775664
CTC-439O9.1	0.045279111	0.036550054	0.001532514	0.87170927	0.040873923
CTC-436K13.1	0.01628774	0.001088354	3.16E-06	0.374692094	0.0220599
CTC-429P9.5	0.017414189	0.674017157	0.517418759	0.878010548	0.00345184
CTC-429P9.3	0.015421174	0.674911844	0.524614229	0.868268476	0.0022212
CTC-429P9.2	0.004950232	0.666314718	0.522207094	0.850190103	0.001093714
CTC-429P9.1	0.006480417	0.585679943	0.4127183	0.831126209	0.002737502
CTC-428G20.6	0.000806427	0.292165334	0.106579435	0.800910444	0.016781928
CTC-412M14.6	0.0059745	0.239709812	0.089769079	0.640095612	0.004368695
CTC-412M14.5	0.005091003	0.138424811	0.029266428	0.65472386	0.012624494
CTC-394G3.2	0.038824233	22708065852	334399.4797	1.54204E+15	2.66E-05
CTC-360J11.7	0.008728767	2.27486E+13	3.277549117	1.58E+26	0.041484824
CTC-360J11.6	0.005878287	305.7129116	4.656672368	20070.20828	0.007350999
CTC-326K19.6	0.001424346	2.44E+24	26757480917	2.23E+38	0.000617154
CTC-308K20.4	0.012452196	0.31301801	0.124610332	0.786293346	0.013452102
CTC-304I17.5	0.031943391	8.51E-10	1.89E-18	0.382937303	0.039938055
CTC-281M20.4	0.000407254	9.64811E+16	744535.6644	1.25E+28	0.002738958
CTC-273B12.10	0.011899486	0.409780124	0.194543517	0.863147497	0.018917138
CTC-239J10.1	0.014625399	0.051963826	0.004399596	0.613747046	0.018900688
CTC-232P5.1	0.011276765	0.635999001	0.40836214	0.990529459	0.045278268
CTBP2	0.024942239	1.623210691	1.152368197	2.286433238	0.005582934
CTB-66B24.1	0.000484663	0.273552893	0.077741121	0.962568894	0.043446088
CTB-58E17.3	0.004323866	0.533797916	0.355816029	0.800807698	0.002418423
CTB-58E17.1	0.00774806	0.561735882	0.386988485	0.815391706	0.002418133
CTB-51J22.1	0.004005136	0.796349892	0.684705975	0.926197775	0.00312896
CTB-50L17.14	0.027470468	0.512135704	0.273976916	0.957317803	0.036025987
CTB-43E15.1	0.013419873	0.530538083	0.316154157	0.890295611	0.016398416
CTB-31O20.2	0.001692574	0.607783102	0.461039217	0.801234006	0.000412859
CTB-193M12.5	0.021529108	1.219862452	1.003583582	1.482750844	0.045946019
CTB-162E12.1	0.004201514	1522758562	3084.594137	7.51734E+14	0.001571636
CTB-152G17.6	0.039880941	0.236282595	0.092734986	0.602032388	0.002499881
CTB-131B5.5	0.007702095	0.564780197	0.351369803	0.907809004	0.018304626

CTB-113P19.4	0.044552111	0.115459254	0.013946963	0.955823825	0.045299756
CTAG1B	0.031384764	7023.153314	3.373882251	14619562.51	0.023092797
CTA-392C11.1	0.020166908	2.232987433	1.58843911	3.139077124	3.78E-06
CTA-369K23.1	0.000648787	3.77E+45	1.15491E+16	1.23E+75	0.00247277
CTA-313A17.2	0.00255232	0.43879576	0.211779939	0.909159385	0.026678517
CTA-292E10.9	0.007485415	0.638567968	0.427282469	0.954331336	0.028670272
CTA-292E10.8	0.005141287	0.388488909	0.202461841	0.745442361	0.004462506
CTA-246H3.8	0.002263996	0.228326171	0.075542987	0.690108277	0.00886514
CTA-246H3.11	0.008860557	0.464918236	0.257445394	0.839591505	0.011093445
CTA-221G9.12	0.011970769	0.656427335	0.505504586	0.852409371	0.001588749
CT867976.1	0.003581511	114.6851811	3.307791692	3976.275408	0.008762201
CST5	0.034258793	0.707329379	0.538513189	0.929067032	0.012817797
CSNK2B-LY6G5B-562	0.000408524	6.069183351	1.151407355	31.99127259	0.033486536
CSF2RB	0.011139953	0.804292714	0.683097892	0.946989849	0.008959706
CRYBA4	0.033424836	0.196894786	0.051152599	0.757880491	0.01812312
CRY2	0.001035871	0.744700107	0.60345034	0.919012241	0.00601408
CRTC1	0.025484235	0.748750568	0.572864669	0.978638487	0.034173077
CRTAC1	0.003156081	0.834531607	0.75255132	0.925442537	0.000606588
CRP	0.012192295	1.311819078	1.010966072	1.702202816	0.041149612
CROCCP3	0.030047335	0.596194961	0.386579883	0.919469552	0.019294154
CROCCP2	0.018332551	0.729931586	0.591607868	0.900596744	0.00331802
CRNDE	0.000885743	0.700994649	0.607430956	0.808970128	1.17E-06
CREG2	0.003816555	1.571697734	1.275920875	1.936039935	2.13E-05
CREBRF	0.002519236	0.552586987	0.391779567	0.779398426	0.000723933
CRBN	0.013525145	0.5338087	0.360008328	0.791514268	0.001788293
CR2	0.035123918	0.823195681	0.714769129	0.948069946	0.006933875
CR1	0.010714498	0.692397086	0.479661029	0.99948442	0.049679002
CPT2	0.031612022	0.691947285	0.480911031	0.995591732	0.047282141
CPSF2	0.033099283	1.557889465	1.090565089	2.225469721	0.01483349
CPEB3	0.041753184	0.499500467	0.287379247	0.868193229	0.01385171
CPAMD8	0.003961271	0.821759806	0.714064352	0.945697929	0.006163475
COX6CP4	0.009731808	15.89422942	1.975583394	127.8743937	0.009323233

COX6B2	0.046994047	1.450658673	1.052604642	1.999241217	0.023011802
CORO1A	0.014010648	0.80447836	0.690094065	0.937822053	0.005429974
COQ10A	0.02008746	0.599236308	0.431956841	0.831296368	0.00216719
COLGALT1	0.011590495	1.275041921	1.018544438	1.596132521	0.03398025
COLCA1	0.000183215	0.733906149	0.616589058	0.873544913	0.000499041
COL6A6	0.015231579	0.673757635	0.513311084	0.884355248	0.004433302
COL5A2	0.049289226	1.146917333	1.024387835	1.284102878	0.017408938
COL21A1	0.047248062	0.823373826	0.683947551	0.991222874	0.040063218
COL12A1	0.047305774	1.108725378	1.007699634	1.219879339	0.034233348
COL11A1	0.029100079	1.095974112	1.011791443	1.187160913	0.024611753
COA6	0.026184243	1.287053476	1.069041383	1.549525282	0.007698593
CNTN3	0.002737989	0.699941175	0.52031293	0.94158269	0.018384975
CNR2	0.0157537	0.466442079	0.259340858	0.838927637	0.010884163
CNNM1	0.008683271	1.502396819	1.151215163	1.960707498	0.002730069
CNIH1	0.013094021	1.474932968	1.170603892	1.858380342	0.00098095
CNGA3	0.000637821	0.843852822	0.720966107	0.987685244	0.03449182
CMTM5	0.012917867	0.005235327	5.70E-05	0.481063221	0.02277258
CMAS	0.002907337	1.408333598	1.134822577	1.747765302	0.001883798
CMAHP	0.000300256	0.709203538	0.607583927	0.82781923	1.33E-05
CLUL1	0.001518934	0.658398589	0.487449168	0.889300321	0.006433067
CLUHP3	0.038119203	0.724418807	0.586490242	0.894784891	0.002775309
CLSPN	0.010226035	1.322675827	1.096365132	1.595701371	0.003490737
CLIC6	0.0046838	0.855090012	0.791487143	0.923803923	7.20E-05
CLIC5	0.041970678	0.848883532	0.743621528	0.969045709	0.015288618
CLECL1	0.002688067	0.544437873	0.37560995	0.789150015	0.001326099
CLEC9A	0.016030133	0.394451876	0.17835787	0.872360062	0.021609298
CLEC4F	0.014860602	0.553547943	0.348664314	0.878826174	0.012153458
CLEC4A	0.003512227	0.811846566	0.687292491	0.958972863	0.014168938
CLEC3B	0.008340253	0.766642049	0.674811593	0.870969079	4.46E-05
CLEC2D	0.018742859	0.7031981	0.550088977	0.898922882	0.004946868
CLEC17A	0.000202332	0.391298475	0.239810076	0.638482331	0.000172681
CLEC12B	0.000970742	0.108217413	0.015684848	0.746644684	0.024042868

CLEC12A	0.017055375	0.745413139	0.597957699	0.929230861	0.008985109
CLEC10A	0.000511617	0.787243267	0.672657239	0.921348832	0.002876483
CLDN23	0.01517256	0.791784873	0.648901249	0.966130494	0.021487267
CLDN2	0.038900746	0.897618611	0.830163277	0.970555061	0.006732799
CLCN6	0.020140707	0.672431357	0.510089898	0.886439688	0.004877683
CLCN1	0.001733721	2.20231558	1.358844937	3.569350544	0.001352592
CLASP2	0.005558683	0.632218805	0.402428014	0.993222648	0.046649314
CKS1B	0.002766141	1.307176886	1.118166873	1.52813632	0.000774864
CKAP4	0.000856546	1.442179262	1.169376839	1.778623413	0.000620409
CKAP2L	0.017739598	1.403773374	1.178214238	1.672513895	0.000147637
CKAP2	0.049118365	1.242085358	1.013689789	1.521940984	0.036517627
CISH	0.010733074	0.722541889	0.618200039	0.844494902	4.43E-05
CISD2	0.001980874	1.371761697	1.004997595	1.872372795	0.046440238
CISD1P1	0.031040809	0.022493216	0.000660317	0.766214511	0.035040379
CISD1	0.002410242	1.403301444	1.041497232	1.890792296	0.025931782
CIRBP	0.014759864	0.693332845	0.55963965	0.858964216	0.000805396
CIITA	0.013749715	0.760290345	0.642347146	0.899889433	0.00144051
CIDEC	0.004673897	1.450150671	1.218168738	1.726310078	2.93E-05
CIDEB	0.006851401	0.218918312	0.08194036	0.584879382	0.002448059
CHRNA6	0.003735051	0.085905154	0.018890608	0.390654199	0.001491676
CHRDL1	0.000134592	0.806123407	0.712575441	0.911952491	0.000616066
CHIT1	0.026106985	0.888820986	0.810478891	0.974735744	0.012296826
CHIAP2	0.000925186	0.728443049	0.533971756	0.993740342	0.045544293
CHIA	0.01165613	0.872760607	0.770762974	0.988255927	0.031851441
CHEK2P4	0.003968397	615.031681	10.00063546	37823.99328	0.002245823
CHEK1	0.009400996	1.423292762	1.183868351	1.711138139	0.000172602
CHDH	0.046278827	0.772711481	0.616549151	0.968427306	0.025189952
CHD6	0.019251897	0.781281913	0.614514283	0.993307145	0.043927569
CHCHD4P2	0.024641576	93.76728947	1.908347206	4607.287682	0.02230193
CHCHD3	0.01445119	1.403520245	1.023875698	1.923933815	0.035152732
CHCHD2P3	0.031236254	353.1840212	6.973197682	17888.34312	0.003392162
CHAF1B	0.011264425	1.376742704	1.113527028	1.702177337	0.003144638

CHAF1A	0.032801582	1.28768553	1.02674063	1.614949263	0.028643302
CHAD	0.013852361	0.828788194	0.700029393	0.981230042	0.029263304
CH25H	0.003969934	0.851586452	0.76065649	0.953386311	0.005295061
CH17-472G23.4	0.004539907	0.499300374	0.312834965	0.796908566	0.003595299
CH17-38B12.4	0.008904958	2.80581E+14	984317.3974	8.00E+22	0.000810299
CH17-340M24.3	0.001379083	0.686598057	0.564954193	0.834433831	0.000157339
CH17-264B6.4	0.001709498	9.10E+25	167510.7892	4.94E+46	0.014136815
CH17-13I23.3	0.008088288	1.518704569	1.245436225	1.851932296	3.65E-05
CGNL1	0.024302192	0.759076344	0.633751143	0.909184784	0.002752735
CFTR	0.000933279	0.777411779	0.66919316	0.903130979	0.000994129
CFL1	0.002562894	1.908055258	1.347482306	2.701835009	0.00027221
CFAP221	0.009586637	0.814725768	0.680861644	0.974908901	0.025257496
CES5A	0.049136376	7.742937919	1.292503242	46.38525126	0.025034094
CES4A	0.03326364	0.725770088	0.591922378	0.889883944	0.002058796
CERS6-AS1	0.011523025	8.68E-05	1.32E-08	0.569979869	0.037040012
CERS4	0.000678228	0.708313369	0.612468808	0.819156537	3.33E-06
CERKL	0.000782056	0.708787482	0.573908097	0.875366103	0.001393783
CEP70	0.014285932	0.765632068	0.604971814	0.968958304	0.026257426
CEP68	0.044298095	0.634516263	0.431552604	0.932935832	0.020726572
CEP55	0.010304129	1.275095673	1.114001346	1.459485647	0.000420931
CEP104	0.024022426	0.689846105	0.481775665	0.98777851	0.042652158
CENPW	0.010689029	1.167053419	1.021385303	1.333496457	0.023144932
CENPU	0.000527106	1.315138742	1.12646437	1.535414662	0.000526011
CENPN	0.020523086	1.35176219	1.099286869	1.662224001	0.004271864
CENPM	0.005548155	1.313851391	1.114825436	1.548408764	0.001126297
CENPK	0.000800526	1.371431491	1.113405415	1.689253805	0.002976971
CENPH	0.002202769	1.47586986	1.194585194	1.823387613	0.000308539
CENPF	0.004575644	1.263007639	1.101983238	1.447561307	0.000792097
CENPE	0.002387022	1.445794257	1.171206187	1.784759213	0.000602364
CENPC	0.009341301	0.475839306	0.301472181	0.751057842	0.001426048
CENPA	0.020733675	1.227965784	1.070859705	1.408120934	0.003280693
CECR1	0.001478933	0.799333722	0.693260413	0.92163693	0.00204679

CEBPA	0.042085772	0.861070567	0.753582571	0.983890219	0.027899723
CEACAMP11	0.010106108	114.3855866	8.127906071	1609.770377	0.000443033
CEACAM21	0.006989718	0.725195612	0.571789379	0.919759435	0.008055579
CDY2B	3.82E-05	2.51E+108	3.49E+35	1.81E+181	0.003544322
CDT1	0.035667821	1.283511767	1.104826966	1.491095445	0.001101184
CDO1	0.041065338	0.712894116	0.522288296	0.973060327	0.033006219
CDKN3	0.000491463	1.317019834	1.150053539	1.508226517	6.85E-05
CDKL2	0.003026705	0.776570964	0.662132773	0.910787817	0.001878496
CDKAL1	0.023465245	0.642419001	0.449581857	0.917968923	0.015099971
CDK15	0.005987123	0.231069255	0.071316065	0.748681247	0.014584951
CDK1	3.48E-05	1.273785278	1.116573093	1.453132756	0.00031753
CDH26	8.08E-05	0.74048147	0.618443843	0.886600801	0.001076061
CDH24	0.044752092	1.236518176	1.044103613	1.464392212	0.013890709
CDH17	0.032592462	1.174978622	1.075297013	1.283900862	0.000363919
CDCP1	0.004725008	1.344310309	1.16104735	1.556500007	7.59E-05
CDCA5	0.001602594	1.31606255	1.144234411	1.513693889	0.000119347
CDCA4	0.036859171	1.389352375	1.146678614	1.683383643	0.000786846
CDCA3	0.000383509	1.319608841	1.105576582	1.575076319	0.0021295
CDCA2	0.005843959	1.449932475	1.193240868	1.761843931	0.000186122
CDC6	0.001765597	1.265332592	1.096653265	1.459956962	0.001264623
CDC45	0.009352153	1.225039592	1.06487204	1.409297968	0.004523127
CDC42P4	0.030243897	0.195017942	0.041879686	0.908125183	0.037273596
CDC42BPB	0.0056875	1.497114143	1.155160154	1.940294382	0.002286754
CDC37L1	0.027710464	0.651380848	0.452774631	0.937104203	0.020886218
CDC37	0.011625053	1.418581609	1.016740236	1.979240824	0.039622658
CDC25C	0.000250078	1.518514182	1.238554783	1.861754806	5.88E-05
CDC20P1	0.028481963	1.704744888	1.183403006	2.455761156	0.004181321
CDC20	0.038200527	1.211368754	1.081783089	1.356477349	0.00089451
CDADC1	0.001064073	0.421601855	0.235052923	0.756204699	0.003762678
CDA	0.010086685	1.099367019	1.017203296	1.188167446	0.016832471
CD99L2	0.025435473	0.768239989	0.625486307	0.943574104	0.011946803
CD84	0.019568194	0.804723826	0.673383976	0.961680792	0.016859758

CD81	0.017532619	0.784995783	0.631821924	0.975303888	0.028834014
CD80	0.014919144	0.641552901	0.443652372	0.92773115	0.018345848
CD79B	0.006408247	0.803523373	0.695601412	0.928189334	0.002952645
CD79A	0.024630198	0.862739353	0.785373352	0.947726568	0.002070169
CD74	0.001898211	0.862733636	0.776662786	0.958342979	0.005897007
CD69	0.035095621	0.807759119	0.691865346	0.943066157	0.006896418
CD53	0.009425768	0.871391603	0.765193474	0.992328545	0.037884427
CD52	0.004971707	0.842495498	0.747615015	0.949417347	0.004931904
CD5	0.002570751	0.744313664	0.624305788	0.887390189	0.000995656
CD48	0.022706016	0.835903142	0.727110721	0.960973401	0.01175097
CD40LG	0.004450403	0.603035786	0.459661858	0.791129724	0.000260791
CD3EAP	0.001597161	1.632735305	1.225796246	2.174769733	0.000802494
CD37	0.005834908	0.822509537	0.708779689	0.954488325	0.010069267
CD33	0.003965694	0.675510951	0.500332892	0.91202288	0.010430628
CD302	0.000269809	0.691774544	0.573815631	0.833982196	0.0001119
CD300LG	0.035099679	0.44769683	0.221392034	0.905328201	0.025300262
CD300LF	0.000860327	0.790162564	0.670079661	0.931765152	0.005105832
CD28	0.012535004	0.695249235	0.526337095	0.918368671	0.010478446
CD27	0.041925677	0.845703634	0.74016742	0.966287651	0.013730933
CD247	0.047902983	0.770864576	0.622493146	0.95460038	0.017035619
CD226	0.001813901	0.39385875	0.212090858	0.731406892	0.003173788
CD22	0.008611604	0.747988818	0.612582124	0.913326149	0.004375362
CD207	0.032511515	0.90185241	0.821976197	0.989490661	0.029017797
CD200R1	6.46E-05	0.424904267	0.278799087	0.647576137	6.86E-05
CD2	0.005080979	0.836896621	0.730983758	0.958155289	0.009904834
CD1E	0.006654779	0.77776713	0.66018187	0.916295548	0.002653727
CD1D	0.000958449	0.637923665	0.491145459	0.828566355	0.000752791
CD1B	0.004756075	0.708594422	0.559223518	0.897862909	0.004345257
CD19	0.004636023	0.768647604	0.646000391	0.914580157	0.003010029
CD180	0.040061481	0.764082457	0.611122533	0.955327237	0.018227872
CD164L2	0.013526004	0.787180538	0.656355519	0.944081647	0.009866375
CD160	0.023719291	0.207547391	0.067086358	0.64209656	0.006356707

CD109	0.013272006	1.31935911	1.16105418	1.499248262	2.14E-05
CCT8	0.044889646	1.540741249	1.137534052	2.086868162	0.005231614
CCT7	0.026392906	1.534818653	1.109505455	2.12316964	0.009664549
CCT6A	0.008359281	1.458511498	1.221105237	1.742074086	3.13E-05
CCT5	0.026794396	1.279173453	1.042176173	1.570065375	0.018516878
CCT4	0.006365722	1.461740623	1.134868124	1.882761181	0.003285919
CCT2	0.014569142	1.180702395	1.026677411	1.357834632	0.019852626
CCR7	0.037353112	0.815682235	0.69600317	0.955940341	0.011850655
CCR4	0.023879959	0.731383651	0.596640123	0.896557278	0.002603958
CCR2	0.000290542	0.65747613	0.536857988	0.805194059	5.01E-05
CCNT2-AS1	0.027564506	0.519721764	0.31000489	0.871311135	0.013046418
CCNL2	0.047629086	0.836253978	0.719920367	0.971386209	0.019292795
CCNE1	0.009141368	1.19273964	1.047009433	1.358753611	0.00802823
CCNB2	0.002231108	1.284570323	1.119763106	1.473633938	0.00035072
CCNB1IP1P1	0.015015994	0.000458842	1.18E-06	0.178963617	0.011563626
CCNB1	0.000748649	1.33362251	1.163665557	1.528402202	3.49E-05
CCNA2	7.60E-05	1.319065087	1.155949134	1.505198329	3.93E-05
CCM2	0.007281361	1.339873112	1.05532097	1.701150652	0.016304413
CCL3L3	0.04990598	0.784165074	0.637913439	0.963947185	0.020964957
CCL20	0.007767745	1.105926117	1.027193676	1.190693249	0.007539731
CCL14	0.01494295	0.595169752	0.405478203	0.873603147	0.008047538
CCDC90B	0.004602258	1.329754012	1.042509858	1.696142938	0.021718952
CCDC88C	0.014604191	0.739706637	0.595137113	0.919394701	0.00657957
CCDC86	0.000760108	1.388013073	1.082293285	1.780090773	0.009795392
CCDC69	0.007462697	0.770716258	0.651899059	0.911189458	0.002298445
CCDC65	0.031898675	0.754069335	0.581538128	0.977787243	0.033219201
CCDC30	0.008412737	0.499033138	0.258127472	0.964771674	0.038772533
CCDC28A	0.01171666	0.64258189	0.491460809	0.840171746	0.001224826
CCDC181	0.004593791	0.56403051	0.392801694	0.809900825	0.001921228
CCDC173	0.048821233	0.734436932	0.543560627	0.992341205	0.044427757
CCDC153	0.004915043	0.688626411	0.527543548	0.898895146	0.006070229
CCDC152	0.021259286	0.502300508	0.296143047	0.851972731	0.010642078

CCDC149	0.029678239	0.664529579	0.472388558	0.934822729	0.018923518
CCDC146	0.029705193	0.753279035	0.603136669	0.940797224	0.012487884
CCDC137	0.003308313	1.41840682	1.089833741	1.846041127	0.009327442
CCDC13-AS1	0.002869058	0.157324246	0.041555217	0.595615195	0.006472784
CCDC121	0.013319991	0.653138521	0.437943257	0.974075797	0.036729114
CCDC101	0.003554999	0.593824236	0.429300028	0.821400421	0.001640784
CCAT1	0.000315912	1.202027781	1.034212125	1.397073919	0.016465269
CC2D2B	0.019320291	0.002713996	8.24E-05	0.089432663	0.000920243
CBX7	0.008603131	0.678481609	0.539228569	0.853696039	0.000934413
CBX5	0.002517235	1.462174315	1.145001931	1.867205347	0.002324261
CBX3	0.016374991	1.345818677	1.042315564	1.737696311	0.022737391
CBFA2T3	0.009016829	0.613714545	0.46329009	0.812979926	0.000665978
CAT	0.012182949	0.722707933	0.596112237	0.876188617	0.000949163
CASZ1	0.003542084	0.650406395	0.492355849	0.859192552	0.002458509
CASS4	0.00602272	0.641899767	0.49720059	0.828710422	0.000669952
CASP12	0.020157269	0.209100188	0.06806631	0.642357264	0.006277756
CASP10	0.020087325	0.734694982	0.582545603	0.926582766	0.009213841
CASD1	0.017426899	0.730284746	0.562784114	0.947638352	0.018051759
CASC8	0.007004669	1.588719545	1.262563628	1.999130767	7.86E-05
CASC5	8.01E-05	1.588242014	1.255964041	2.00842748	0.000112015
CARHSP1	0.001838769	1.414849832	1.109905984	1.803576226	0.005080424
CARD8-AS1	0.009003841	0.679443004	0.509013466	0.906936314	0.008718479
CAPZA1	0.0085917	1.510513685	1.123181472	2.031418475	0.006363976
CAPS	0.025124811	0.820546317	0.725247585	0.92836746	0.001689688
CAPNS1	0.011756125	1.507779102	1.141059148	1.992357561	0.003876782
CAPN3	0.030020368	0.585594162	0.391426337	0.876079328	0.009223373
CAPN13	0.003357853	0.875069278	0.791451718	0.967521106	0.009205997
CAND1	0.03531622	1.309718001	1.053495161	1.628257352	0.015134418
CAMTA2	0.004867089	0.683015812	0.505621013	0.922648757	0.012967763
CAMTA1	0.028439146	0.540001065	0.363664044	0.801842126	0.002251874
CAMKK1	0.034448116	0.566774757	0.407945057	0.787443358	0.000713625
CAMK1D	0.025627835	0.785676028	0.681362642	0.905959299	0.00090401

CAMK1	0.027478926	0.691586783	0.506662412	0.944005847	0.020182179
CALCOCO1	0.020959456	0.746689031	0.584499431	0.953883749	0.019396077
CAHM	0.033258448	0.602189098	0.380498011	0.953044954	0.030365865
CADM3-AS1	8.61E-05	0.27036744	0.083865251	0.871619071	0.028522307
CADM1	0.016199481	0.860601711	0.760132606	0.974350131	0.017777475
CACNB1	0.005199409	0.719251853	0.586574037	0.881940207	0.001537548
CACNA2D2	0.001013937	0.847532759	0.774438662	0.92752572	0.000324505
CACNA1D	0.041320529	0.766943202	0.608204376	0.967112205	0.024922931
CABLES1	0.000878246	0.782768324	0.657433906	0.931996726	0.005941104
CABIN1	0.040063243	0.782277059	0.614920099	0.995181974	0.045577186
CA5B	0.017864912	0.333294586	0.190698747	0.582517101	0.000114802
CA4	0.003422487	0.741445432	0.596730657	0.92125538	0.006927801
CA3	0.039750861	0.798658694	0.651272259	0.979399477	0.030776677
C9orf72	0.029664704	0.724772846	0.544727144	0.964328075	0.027156344
C9orf40	0.00752876	1.339779275	1.045125021	1.717506011	0.020984619
C9orf173	0.037467959	0.651001714	0.459461773	0.922390624	0.015762715
C6orf3	0.023837928	0.358768101	0.173257807	0.742907652	0.005777065
C6	0.025485524	0.681122145	0.528273771	0.878194986	0.003059264
C5orf56	0.034879519	0.61027404	0.414309596	0.898927777	0.012447764
C5orf38	0.004471092	0.834927493	0.744147862	0.936781456	0.002126612
C4orf48	0.007074652	1.168863853	1.014085398	1.347265929	0.031321758
C4A	0.048442212	0.81942457	0.690686133	0.972158835	0.022385812
C3orf62	0.016087529	0.585313983	0.39685945	0.863258916	0.006899626
C3orf18	0.000666558	0.519345562	0.3779571	0.713625471	5.33E-05
C2orf78	0.031149751	1451567630	1648.190755	1.2784E+15	0.002522859
C2orf42	0.039950378	0.508603495	0.280400474	0.922528806	0.026055396
C2orf40	0.040591703	0.793464404	0.660571994	0.953091816	0.013373122
C2CD2	0.032004368	0.728378852	0.53610185	0.98961746	0.042692641
C20orf24	0.006639461	1.340548261	1.083253583	1.658955638	0.007029619
C20orf197	0.000772179	0.76423627	0.616982004	0.946635514	0.013810652
C20orf194	0.016190672	0.657135283	0.486321926	0.887944297	0.006261123
C1QTNF7	0.025480419	0.611877775	0.446906007	0.837747549	0.002181242

C1QTNF6	1.30E-06	1.592109116	1.340907657	1.890369874	1.11E-07
C1QL1	0.039188745	1.244721203	1.031462847	1.502071429	0.022428322
C1orf210	0.034417769	0.731891097	0.611071326	0.876599105	0.000697038
C1orf168	0.012305795	0.701466695	0.534793535	0.920085028	0.010416429
C1orf132	0.018748331	0.65976795	0.464471134	0.937181485	0.020219346
C1orf112	0.020129011	1.382446006	1.016986898	1.879234593	0.038687235
C1orf105	0.01837677	2.158892753	1.057889428	4.405770393	0.034464811
C1orf101	0.006693047	0.317429312	0.141898989	0.710092223	0.005216082
C19orf48	0.029441964	1.288222241	1.065201077	1.557937349	0.009022697
C19orf33	0.00076844	1.133564953	1.025515295	1.252998866	0.01416972
C18orf8	0.034179785	0.615912885	0.428029238	0.886268154	0.009048374
C18orf54	0.001798814	2.035969124	1.45318008	2.852482174	3.59E-05
C17orf58	0.014380521	1.390193244	1.090896423	1.771604724	0.007738075
C17orf53	0.005576768	1.270705174	1.066109102	1.5145651	0.007480635
C16orf93	0.016463983	0.63075774	0.417008926	0.954069091	0.029060656
C16orf89	0.007519197	0.894230223	0.841914112	0.949797229	0.000278492
C16orf74	0.020301342	1.279885737	1.110873446	1.474612168	0.000637569
C16orf59	0.000973297	1.281957492	1.056340244	1.555762947	0.0119061
C16orf54	0.014931925	0.775933461	0.645992076	0.932012572	0.006669581
C15orf41	0.022833123	2.463260668	1.538286994	3.944422037	0.000174871
C14orf39	0.00346494	0.348188622	0.138497024	0.875364057	0.024898788
C14orf166	0.004562026	1.38404819	1.009090273	1.898333027	0.043789789
C14orf132	0.024333913	0.791336396	0.64334491	0.97337102	0.026728213
C12orf76	0.006336089	0.529862325	0.378120212	0.742499539	0.000224717
C11orf24	0.013085613	1.4911027	1.14751954	1.937559391	0.002792696
C11orf21	0.027612489	0.549089824	0.361728988	0.833495918	0.004874171
C11orf16	0.000522781	0.676908379	0.541478182	0.846211296	0.000612348
C10orf128	0.000267163	0.737791467	0.595458359	0.914146624	0.005422107
C10orf111	0.012330987	0.41829271	0.187331377	0.934006861	0.03345845
BZW2	0.01461112	1.381839053	1.107063251	1.72481488	0.004248025
BZRAP1-AS1	0.001308777	0.291080477	0.137431725	0.616508627	0.001267804
BZRAP1	0.000991835	0.649644892	0.516335717	0.817372249	0.000232402

BUB3	0.000221653	1.601442917	1.173623107	2.185215511	0.002982586
BUB1B	0.000607945	1.301070344	1.119384097	1.512245926	0.00060453
BUB1	0.0154172	1.268347818	1.094360287	1.469996862	0.00158983
BTN2A3P	0.044456514	0.532107531	0.334926752	0.845374167	0.007559438
BTN2A2	0.00051557	0.557877952	0.423921345	0.734164044	3.10E-05
BTN2A1	0.000128686	0.711068928	0.529169627	0.955495168	0.023699741
BTLA	0.014994334	0.551530805	0.364178326	0.835267248	0.004954536
BTK	0.000297861	0.722614395	0.599281982	0.871328656	0.00066777
BTG2	0.002973992	0.790595595	0.696935382	0.896842678	0.000259922
BTBD9-AS1	0.036797076	0.159068614	0.040847925	0.619439644	0.008038401
BTBD9	0.002704654	0.714480643	0.59210495	0.862148829	0.000452541
BTBD18	0.012427806	0.001901303	1.04E-05	0.346742902	0.018338116
BSN-AS1	0.026879368	4.66E-05	1.29E-08	0.16833721	0.017022432
BRIP1	0.040774066	1.430156796	1.093184152	1.871000834	0.009057713
BRD9P2	0.019107851	0.606202723	0.438993138	0.837101333	0.002367224
BRCA2	0.045648368	1.537354883	1.07049776	2.207814088	0.019866191
BRCA1	0.003639305	1.294398812	1.051486381	1.593428422	0.014957799
BPIFB1	0.019264923	0.938568577	0.887222005	0.99288675	0.027198635
BOP1	0.026001784	1.207531866	1.010181299	1.443437142	0.038337932
BNIP3P42	0.006805976	0.159459964	0.03322516	0.765307989	0.021778984
BNIP3P40	0.009644115	0.374735005	0.176148285	0.797205173	0.010821571
BNIP3P26	0.017173421	0.208717744	0.059028026	0.738007009	0.015039577
BNIP3P10	0.010504296	0.370217625	0.172766258	0.793332512	0.010608806
BMP5	0.000391139	0.76912632	0.669119204	0.884080582	0.000221114
BMP1	0.026901962	1.279868029	1.050504152	1.559310517	0.014327428
BLZF1	0.021317	1.414288845	1.020327326	1.960363979	0.037455598
BLOC1S4	0.005879992	1.40498256	1.061962434	1.858800209	0.017269138
BLNK	0.043582874	0.767421615	0.607589695	0.969298756	0.02630606
BLK	0.013524339	0.695676189	0.533420279	0.907287142	0.007405901
BIRC5	0.000512744	1.225187869	1.087336185	1.380516288	0.000853495
BIN2	0.02037405	0.806824291	0.66791385	0.974624852	0.02597534
BEX5	0.007761591	0.748205647	0.639939485	0.87478848	0.000275403

BEX4	7.97E-07	0.724362728	0.624192077	0.840608813	2.17E-05
BEX2	0.047253883	0.882849542	0.789722752	0.986958159	0.028467738
BEND5	0.001240978	0.691413891	0.550989599	0.867626484	0.001443313
BDH2	0.007844753	0.680200836	0.535859591	0.863422406	0.001541851
BCL9L	0.014482117	1.268588304	1.049277674	1.53373728	0.014022725
BCL2L13	0.016982092	1.479897824	1.043049835	2.099705591	0.028083893
BCAR3	0.002091567	1.419849226	1.191896939	1.69139777	8.64E-05
BCAR1P2	0.010055259	16435001.96	151.9284235	1.77787E+12	0.004964123
BCAR1	0.001910683	1.519411531	1.178321185	1.959237794	0.001259749
BCAN	0.021754271	1.471661943	1.186350747	1.825588999	0.000441322
BCAM	0.019135652	0.838071595	0.738135773	0.951537676	0.006396158
BBS5	0.02603949	0.583578425	0.382750806	0.889779388	0.012327903
BBS1	0.047804251	0.398508953	0.189842248	0.836533423	0.01502691
BANK1	0.021815051	0.744175472	0.597738873	0.92648673	0.008219128
BANF1	0.003185696	1.400701318	1.078383653	1.819356383	0.011551848
BANCR	0.000121801	0.807349078	0.655025757	0.99509451	0.044849635
BAIAP2L2	0.021003448	1.217831396	1.075642488	1.378816219	0.001863955
BAI3	0.019659889	0.309436351	0.125031554	0.765813528	0.011178977
BACH1	0.009672025	1.310099845	1.011720763	1.696477593	0.040527513
B4GALT4	0.010819408	1.452028985	1.171202148	1.800191519	0.000671274
B4GALT1	0.000447102	1.617123458	1.282230689	2.039483457	4.91E-05
B3GNT8	0.027082238	0.808999774	0.714143478	0.916455382	0.000865282
B3GNT5	0.027933373	1.386718635	1.144557187	1.680115764	0.000841403
B3GNT3	0.036458049	1.182882275	1.047745661	1.335448601	0.006657565
B3GALT2	0.000836993	0.692456574	0.532886561	0.899808969	0.005960968
B3GALT1	0.021822417	3.195500061	1.466720792	6.961938969	0.003455439
B3GALNT1P1	0.021465454	0.002906156	1.89E-05	0.446324435	0.022963644
AZIN2	0.028076336	0.624549006	0.458836744	0.850109468	0.002769629
AVPR2	0.022139858	0.563959879	0.330232416	0.963111826	0.035939538
AVEN	0.003690553	1.717100863	1.319901739	2.23382945	5.63E-05
AURKB	0.007248848	1.185726243	1.048806553	1.340520536	0.006505763
AURKA	0.019706017	1.267863621	1.102682467	1.457788811	0.000860929

AUNIP	0.005935184	1.318705904	1.067902124	1.628412587	0.010159842
ATRIP	0.02046394	0.004537064	5.63E-05	0.365696835	0.015990584
ATP8B4	0.00057481	0.475101884	0.27340711	0.825588626	0.008295989
ATP8B3	0.000274761	1.368130567	1.129549264	1.657104571	0.001346267
ATP8B2	0.000213967	0.765026481	0.632041809	0.925991775	0.00597476
ATP8A2	0.004160031	0.434802093	0.286655952	0.65951137	8.92E-05
ATP8A1	0.000321557	0.74475665	0.632855645	0.876443897	0.000388814
ATP6V1B2	0.002084614	0.662550427	0.501491561	0.875334906	0.003767804
ATP5HP2	0.000478057	6.043058426	1.559844624	23.41166201	0.009231371
ATP5G1P4	0.002138445	1.666137932	1.282761228	2.16409379	0.000130032
ATP13A4	0.011277215	0.837309857	0.751512282	0.932902646	0.001285637
ATP11B	0.033748572	1.392939434	1.077349553	1.800975607	0.011460054
ATL2	0.002160823	0.777449408	0.608374574	0.993512234	0.044221426
ATIC	0.04499318	1.560894876	1.159870722	2.100572734	0.003294379
ATG2B	0.025004589	0.699233461	0.495828119	0.986082503	0.041362907
ATF7IP2	0.000166663	0.559674675	0.40707389	0.769481288	0.000352667
ATAD3C	0.006092221	0.78255587	0.662588921	0.92424378	0.003879234
ATAD2	0.019922464	1.233364294	1.05073153	1.447741347	0.010312106
ASUN	0.005412315	1.447627804	1.132213728	1.850910484	0.00317459
ASTL	0.02158679	0.284384552	0.083998427	0.962810568	0.043292951
ASTE1	0.034727526	0.622651428	0.396518046	0.977748185	0.039618848
ASPM	0.00047158	1.388911001	1.17456006	1.642379845	0.000122431
ASPH	0.036867855	1.214420333	1.069454833	1.379036029	0.002741753
ASF1B	0.004331103	1.252305804	1.072704949	1.461976874	0.004391788
ASCL4	0.003204072	3.20E-05	1.90E-08	0.053868844	0.006322022
ASB9P1	0.002914298	0.322714893	0.154032072	0.676124789	0.002725474
ASB2	0.005230946	0.523928903	0.359033048	0.764557739	0.000801846
ASB16	0.016168893	0.646265393	0.441701352	0.945568668	0.024563275
ASB14	0.013139883	0.276324183	0.11449505	0.666885197	0.004220002
ASAP3	0.006774094	0.73611332	0.609182517	0.889491745	0.00151051
ASAH2B	0.016066467	1.912401686	1.180776019	3.097353053	0.008403627
ARVCF	0.008013528	0.736323188	0.594794886	0.911527402	0.004945774

ART4	0.033308769	0.494335661	0.273594777	0.893174015	0.01958179
ARRDC5	0.0182316	0.384717981	0.18442179	0.802551181	0.01088763
ARRB1	0.036387033	0.706937814	0.583608369	0.856329517	0.000391688
ARNTL2	0.001496175	1.368158001	1.201317673	1.558169297	2.31E-06
ARNTL	0.011375203	0.676135262	0.491915756	0.929343871	0.015888137
ARMCX6	0.021502363	0.625248012	0.46057657	0.848794971	0.002602699
ARMCX3	0.010501274	0.7525089	0.575545422	0.9838835	0.037640619
ARMC2	0.034729856	0.640753809	0.411594475	0.997499888	0.048718281
ARL14	0.015786095	1.215702817	1.09981319	1.343803977	0.000132754
ARIH2OS	0.022003085	0.390684502	0.22912018	0.666176066	0.000556798
ARIH2	0.046783552	0.629318953	0.421857604	0.938805751	0.023244013
ARID4A	0.022776369	0.660446683	0.470010433	0.928042847	0.016837146
ARHGEF6	0.00245767	0.734230157	0.601403281	0.896393386	0.002411675
ARHGEF4	0.044445746	1.355772448	1.119053541	1.642565672	0.001877984
ARHGEF39	0.008561143	1.339872936	1.03280534	1.738236061	0.027593711
ARHGEF38	0.024182482	0.737599763	0.597313967	0.910833231	0.004688686
ARHGEF3	0.000645388	0.662510597	0.489850373	0.896029309	0.007526728
ARHGEF26-AS1	0.004210021	0.632032925	0.463689412	0.86149394	0.003691406
ARHGAP9	0.000864878	0.745170685	0.612317362	0.906848939	0.003325446
ARHGAP44	0.004157947	0.762125425	0.627573946	0.925524662	0.006127701
ARHGAP30	0.002540058	0.78627922	0.661693138	0.934322839	0.006299242
ARHGAP25	0.002241871	0.722668555	0.575922361	0.906805979	0.005036302
ARHGAP15	0.00016528	0.631537892	0.465975562	0.855924948	0.003047579
ARHGAP11A	0.000741922	1.457823949	1.227626413	1.731186819	1.72E-05
ARGFXP2	0.022839927	0.273342448	0.091429853	0.817195828	0.020273004
ARG2	0.019646648	0.815446321	0.679350395	0.978806678	0.028532027
ARF3	0.013394577	1.459722187	1.047335132	2.03448619	0.025549117
AQP4-AS1	0.033838761	0.163012427	0.036751008	0.723056406	0.017004649
APOBEC3H	0.022586348	0.746771419	0.582940533	0.956645695	0.020849052
APBB1	0.021486776	0.80943225	0.659204619	0.993895597	0.043547482
AP3S1	0.026919132	1.898870448	1.352459164	2.666039073	0.000212351
AP3B1	0.039779622	1.453698147	1.030938666	2.049819619	0.032863362

AP2M1	0.005818089	1.714376365	1.261731827	2.329406501	0.000568282
AP1S3	0.005830037	1.630304261	1.291322195	2.05827174	3.96E-05
AP1AR	0.020735621	1.419629374	1.039214293	1.939299306	0.027689913
AP005530.2	1.45E-06	8.33813E+16	10935039.69	6.36E+26	0.000790826
AP003774.1	0.002419206	0.541142452	0.324301962	0.902970651	0.018739669
AP003558.1	0.001546091	2.136568957	1.073817811	4.251118635	0.030551868
AP003419.11	0.001068556	7.273982197	2.508202415	21.09511445	0.0002595
AP001885.1	0.003220397	71.61246862	1.820901191	2816.377784	0.022615508
AP001525.1	1.15E-08	18060.44038	72.40998559	4504620.518	0.000500074
AP001462.6	0.015653334	0.515368553	0.304932347	0.871028435	0.01329903
AP000889.2	0.04387152	69759.66519	1.071159161	4543125862	0.048595986
AP000704.5	0.031635099	0.513793866	0.320798206	0.822897794	0.005587108
AP000695.6	0.012546711	1.647566884	1.258148242	2.15751733	0.000284447
AP000695.4	0.000619271	1.786227944	1.340793006	2.379644178	7.38E-05
AP000640.10	0.023930047	0.272288809	0.094463977	0.784862107	0.016020068
AP000568.2	0.016475141	11.94422213	1.605057137	88.88433883	0.015434753
AP000473.8	0.040302928	0.264918583	0.075542006	0.929044108	0.037993039
AP000472.3	0.005059729	1244432.989	49.6748138	31175023049	0.006613547
AP000439.3	0.021465629	1.576459224	1.256436203	1.977994329	8.43E-05
AP000438.4	0.01791674	8.40E-06	1.15E-10	0.612229622	0.040771189
AP000351.3	0.008934236	0.786789056	0.638788754	0.969079395	0.024109691
AP000295.9	0.026248635	0.167427418	0.036584379	0.766227035	0.02127294
AOC4P	0.02028962	0.150564505	0.033458959	0.677536611	0.01361545
AOAH-IT1	0.012135573	0.045891921	0.004094438	0.514373063	0.012449631
ANXA2P3	0.009267863	3.852158623	1.169152049	12.69221234	0.026633263
ANXA2P2	0.00653376	1.219723814	1.047172123	1.420708353	0.010703135
ANXA2	0.009018572	1.329102231	1.092710514	1.616633791	0.004409533
ANTXRLP1	0.019233829	0.107278041	0.01947041	0.591080428	0.010351708
ANP32E	0.020518511	1.238748479	1.058497249	1.449694644	0.007618196
ANP32D	0.00593279	0.003927547	4.08E-05	0.378193027	0.017443668
ANP32B	0.034523615	1.819440828	1.312670983	2.521854273	0.000326485
ANO9	0.028727405	0.847403434	0.72359036	0.992402082	0.039917283

ANO2	0.027766749	0.184611969	0.051127591	0.666598577	0.009906509
ANLN	5.56E-05	1.382016504	1.221794565	1.563249398	2.66E-07
ANKRD65	3.91E-05	0.767911075	0.6859105	0.859714816	4.57E-06
ANKRD6	0.038458858	0.705028489	0.51004211	0.974557121	0.034345698
ANKRD55	0.000865767	0.261085832	0.090084436	0.756688003	0.013379972
ANKRD44	0.001997988	0.630535851	0.486612216	0.817027289	0.000485546
ANKRD36BP2	0.005003699	0.69733537	0.540427426	0.899800038	0.005574971
ANKRD32	0.032280174	1.391990253	1.117982219	1.73315535	0.003105261
ANKRD29	0.002736061	0.800522886	0.68700888	0.932792732	0.004348906
ANKRD2	0.009747823	1.551726294	1.028906452	2.340207399	0.036091072
ANKRD13A	0.012559965	0.662742531	0.504028288	0.871434546	0.003227192
ANKDD1B	0.01100268	0.733995739	0.570364847	0.944570388	0.016257407
ANKDD1A	0.000813539	0.597872412	0.397667032	0.898871146	0.013419949
ANK2	0.026985673	0.583686996	0.408767776	0.833457355	0.003053439
ANGPTL6	0.000179457	0.408290655	0.196926054	0.846517035	0.01604663
ANGPTL4	0.005902198	1.180763829	1.081435005	1.289215916	0.000210421
ANGEL1	0.000723896	0.706290839	0.521607938	0.956363416	0.02454645
AMY2B	0.039867286	0.737650036	0.557765709	0.975548635	0.032880561
AMT	0.000688087	0.706229679	0.584920058	0.852698334	0.000297883
AMIGO1	0.006594874	0.668792055	0.517953749	0.863557438	0.002036276
AMICA1	0.000361787	0.743396727	0.614173761	0.899808376	0.002337749
ALKBH3-AS1	0.03831755	0.10226288	0.01704742	0.613447464	0.012611282
ALK	0.038227584	0.391365002	0.160183294	0.956195624	0.039567092
ALG8	0.003077899	1.448349936	1.107156641	1.894689026	0.006878357
ALG3	0.000125042	1.494186849	1.176906192	1.897002797	0.000975551
ALDOA	0.00197006	1.446680773	1.172732992	1.784622138	0.000565712
ALDH6A1	0.021194128	0.80193885	0.651319114	0.987389907	0.037569172
ALDH2	0.004670202	0.775984656	0.663398395	0.907678089	0.001518669
ALAD	0.003750228	0.699757369	0.5077548	0.964363853	0.029131231
AL662800.1	0.030274102	0.776451206	0.627912206	0.960128613	0.019515296
AL592205.2	5.50E-10	2.43755E+17	95368236.23	6.23E+26	0.000291895
AL590708.2	0.039305513	125.5112872	2.617311307	6018.803791	0.014396573

AL590684.1	0.000781506	53278941.25	73.20326619	3.87776E+13	0.009784078
AL450327.1	0.026497352	0.484899652	0.245190423	0.958959447	0.03748764
AL389915.1	0.000162611	1710.215667	8.728347505	335096.3773	0.005700243
AL359955.1	0.035405856	123.1112554	2.235927081	6778.566855	0.018602174
AL359709.1	0.001431604	48.89896583	2.753160053	868.4961327	0.008051547
AL359538.1	0.015171426	26.54980018	1.613472007	436.8789087	0.021747231
AL359262.1	5.50E-10	673258.6355	472.8579602	958590588.5	0.000291895
AL356213.1	0.023147294	25.10413338	1.98958889	316.7576557	0.012708992
AL356154.1	0.015327183	36.88616023	2.319489435	586.5897884	0.010587402
AL354932.1	0.001427391	27153504.67	36.46369537	2.02205E+13	0.013090892
AL162381.1	0.006854421	0.10718972	0.016626432	0.691046413	0.018843142
AL161452.1	0.046367484	19.57128121	1.199747759	319.2629828	0.036815245
AL158077.1	0.000841752	3137.65282	68.62351553	143461.9772	3.66E-05
AL157702.1	5.50E-10	28821101.91	2651.484145	3.1328E+11	0.000291895
AL139319.1	0.049970541	19.19649962	1.425185533	258.5667544	0.025946971
AL133260.1	0.048987247	0.24790804	0.068934435	0.891548562	0.03269942
AL133245.2	0.000205135	0.031120309	0.001852239	0.522866441	0.015934911
AL122127.2	0.005180624	0.804646246	0.712540922	0.9086574	0.000457787
AL121578.5	0.003423297	1.46775E+11	850628.1429	2.5326E+16	2.93E-05
AL121578.2	0.005405987	4.787610836	1.941775905	11.80425479	0.000670838
AL118520.1	0.034378801	0.123012266	0.015375048	0.984193185	0.048270315
AL096864.1	0.040998732	53430.56884	2.010746416	1419784048	0.036229064
AL050303.10	0.02917975	1.53E+27	3956260.925	5.94E+47	0.009653066
AL035419.1	0.031991349	17.02934974	1.8260739	158.8099761	0.012826244
AL034375.1	0.003709568	0.143815677	0.032911552	0.628440413	0.009956767
AL020997.1	0.006365271	0.421619098	0.219638035	0.809343714	0.009439119
AKR1A1	0.017939743	0.630337113	0.46574884	0.853088277	0.002798065
AKNA	0.015521371	0.718823542	0.576714633	0.895949667	0.003307585
AK9	0.032003053	0.538498012	0.32898794	0.881430817	0.01381803
AK4P4	0.003922443	0.028556313	0.001987383	0.410319954	0.008920182
AK4P1	0.000198367	1.740497192	1.214124361	2.495074287	0.002562519
AK4	0.015993622	1.210750333	1.050869066	1.394956247	0.00812966

AK2P2	0.029690533	0.184948111	0.039270154	0.871038191	0.032794048
AK2	0.031910222	0.659700846	0.492449183	0.88375658	0.005298637
AJ003147.9	0.00160011	4.918056499	1.824919931	13.25388545	0.001637139
AIM1L	0.011700914	1.305586289	1.117086372	1.525894149	0.000803103
AHSG	0.014182474	3.29059049	2.019664815	5.36127861	1.73E-06
AHSA1	0.000210856	1.905872893	1.372035602	2.647417806	0.000119925
AHNAK2	0.006148328	1.30786749	1.159622281	1.475064251	1.23E-05
AHCTF1	0.048337115	1.376164038	1.007480147	1.879766531	0.044770773
AGPS	0.000180361	1.510799563	1.099214337	2.076497043	0.010993152
AGO4	0.018997718	0.661937098	0.493428348	0.8879926	0.005915054
AGMAT	0.019082581	1.312248102	1.056560549	1.629812019	0.013989425
AGFG1	0.005969821	1.557284476	1.187009783	2.043062302	0.001386026
AGER	0.002431755	0.891332226	0.824400556	0.963697965	0.003872087
AGAP3	0.013038256	0.706156878	0.532051434	0.937235584	0.016007884
AGAP2-AS1	0.012441706	1.172040019	1.002075563	1.370832558	0.047044178
AF246928.1	0.002725875	3487.940039	34.35444336	354123.7909	0.000539656
AF186192.5	0.017013487	0.688451171	0.505983629	0.936720059	0.017499883
AF186192.1	0.000178476	0.569587042	0.391703629	0.828252215	0.003214875
AF131215.9	0.0428619	0.615038291	0.42783206	0.884160246	0.008669984
AF131215.4	0.033677066	0.003535311	1.60E-05	0.781171623	0.040400245
AF131215.2	0.018624433	0.635374136	0.460550468	0.876560378	0.005737404
AF111168.1	0.018698819	0.081478316	0.010108111	0.656771158	0.018533034
ADRBK2	0.013189868	0.763333995	0.616600617	0.944985735	0.013157032
ADRB2	0.006821284	0.696115979	0.557993829	0.868427986	0.00132683
ADPGK-AS1	0.014243526	0.014532725	0.001267837	0.166583056	0.000673466
ADM	0.001595485	1.275823688	1.13602268	1.432828861	3.89E-05
ADIPOR2	0.002979663	1.605936613	1.244589285	2.072195573	0.000270126
ADHFE1	0.003423393	0.705956136	0.558465094	0.892399671	0.003591005
ADH1C	0.021762177	0.910409721	0.832432504	0.995691369	0.039929477
ADCK3	0.004792046	0.751973011	0.598555722	0.944713062	0.014345851
ADAMTSL5	0.005438871	1.301127941	1.044259152	1.621181789	0.018980047
ADAMTS9-AS2	0.003020152	0.203777922	0.06446245	0.644180317	0.006751376

ADAMTS8	0.045266104	0.679994748	0.544001423	0.849984647	0.000704913
ADAMTS7P3	0.021086885	0.454848934	0.260892618	0.792998875	0.00547338
ADAMTS4	0.000594207	1.178222846	1.010744954	1.373451404	0.036029922
ADAMTS17	0.001106178	0.512480656	0.285761235	0.919076453	0.024889051
ADAM12	0.030608496	1.206722793	1.049018473	1.388135612	0.008546884
ADAM10	0.004351043	1.497311589	1.129960052	1.984089606	0.004943381
ADA	0.003863187	1.292166994	1.071392336	1.558435209	0.007332903
ACVR2B-AS1	0.000225139	0.54972436	0.331815281	0.910738259	0.020181117
ACTR2	0.003931769	1.567073556	1.116591922	2.19929903	0.009385051
ACTR1B	0.024299793	0.647068862	0.45775893	0.914669459	0.013699546
ACTN4	0.025778315	1.386408464	1.106906448	1.736486793	0.004453029
ACTG1P11	0.010914456	201.1740049	5.657195249	7153.894904	0.003602426
ACTBP2	0.034833344	1.476219208	1.042287588	2.090807925	0.028293802
ACTB	0.016404606	1.471657943	1.093357169	1.980850505	0.010812791
ACSS3	0.028013698	0.663357974	0.485102999	0.907114166	0.010155039
ACSS1	0.000993622	0.743217889	0.632963942	0.872676616	0.000292016
ACSM5	0.002182681	0.329522444	0.17800701	0.6100043	0.000410671
ACSL3	0.018243254	1.435108616	1.157210163	1.779743045	0.001003251
ACSBG1	0.04178067	0.192972117	0.049550063	0.751527553	0.017703715
ACOT7	0.01179477	1.332651206	1.071367808	1.657655964	0.009906371
ACKR1	0.021062026	0.86713609	0.77490355	0.970346567	0.012969829
ACER1	0.003698519	0.018206698	0.000598343	0.55400329	0.021511799
ACCSL	0.000196654	4.24E-09	5.43E-15	0.003317954	0.005361102
ACCS	0.039641221	0.778836593	0.635458852	0.954564464	0.016043671
ACAT2	0.044878665	1.336566921	1.069170488	1.670838426	0.010858431
ACAT1	0.008087883	0.740369371	0.59294414	0.924449316	0.007969366
ACAP1	0.006225947	0.733745668	0.594380183	0.905788452	0.003968495
ACADSB	0.028072843	0.746170359	0.61309057	0.908136956	0.003484816
ACAD8	0.002848516	0.739681585	0.611600247	0.894585721	0.001882218
AC253578.1	0.03746864	9.557549331	1.416742437	64.47660974	0.020469459
AC246787.3	0.023596927	0.833793734	0.732323931	0.949323054	0.006042369
AC246787.1	0.023670634	0.832268287	0.720706591	0.961099163	0.01240877

AC244205.1	0.005938875	0.538436385	0.316181701	0.916921313	0.022649739
AC243980.1	0.019633497	11.01094774	1.041926729	116.3622804	0.046140554
AC233702.1	9.22E-06	60389097.05	591.1244955	6.16933E+12	0.002331221
AC145124.2	0.028589338	0.398328332	0.182246151	0.870610759	0.021039187
AC143336.1	0.03792759	0.072181069	0.005659848	0.920538331	0.043000199
AC139712.4	0.00849443	4377525672	1.835628965	1.04393E+19	0.043894462
AC138811.1	0.000493195	29497808.63	79.99065318	1.08778E+13	0.008538645
AC138783.10	0.007850832	395635616.6	38.51286732	4.06429E+15	0.016252949
AC124861.2	0.002495286	442132.5815	110.2388551	1773251540	0.002134229
AC118550.1	0.025604561	64.69177329	1.00161612	4178.272942	0.049911306
AC117945.1	0.013269655	14.9296091	1.232864417	180.7929768	0.033630181
AC116345.1	0.014561156	28.591763	1.604545921	509.4830261	0.02250579
AC115286.1	0.043398101	7.259302448	1.658386323	31.77635469	0.008501627
AC114973.1	0.00388565	1.52981E+17	25811.26598	9.07E+29	0.008365593
AC113133.1	0.032924316	2.474095786	1.213768658	5.043094431	0.012661617
AC112504.1	0.031189782	0.00682213	6.11E-05	0.761231731	0.038137276
AC109826.1	0.002845402	0.407241742	0.210273314	0.788715571	0.007727678
AC109642.1	0.009614823	0.64316946	0.486405521	0.850456946	0.001958935
AC108057.1	0.002030643	465.9491875	3.260539587	66586.72271	0.01523315
AC108004.3	0.029330562	0.017206042	0.000433498	0.682927802	0.03053988
AC107072.2	0.003847637	0.582080446	0.384410281	0.881395898	0.010577463
AC104837.1	5.50E-10	52.73487237	6.170542184	450.6843453	0.000291895
AC104113.3	0.030822359	0.230659397	0.064812655	0.820885319	0.023531227
AC103863.1	0.028472254	122.9848425	2.373845026	6371.633919	0.016885343
AC098820.2	0.045706873	5.824589856	1.58376833	21.42096564	0.008002028
AC098680.1	0.004352929	388.9655557	4.130606161	36627.60322	0.010122117
AC097711.1	0.003824246	36.78885168	6.172092363	219.2805176	7.55E-05
AC097452.1	0.018005857	210.4704609	1.128489779	39254.06836	0.044933372
AC096559.2	0.034130924	2608953.275	5.82437314	1.16865E+12	0.026056483
AC093911.1	0.028728108	0.078409915	0.010819826	0.568226742	0.011758243
AC093865.1	9.76E-05	311.6063292	5.46921598	17753.64234	0.005373434
AC093724.2	0.006298423	1.473284955	1.047182776	2.072769538	0.026104796

AC093484.4	0.026836719	0.15299774	0.028937869	0.80891611	0.027136347
AC092765.1	0.014773593	0.630547028	0.425049511	0.935395863	0.021913248
AC092168.3	0.000254769	2129.542034	8.30405969	546112.3168	0.006771029
AC092159.3	0.002625489	0.003130803	1.59E-05	0.614662779	0.032303878
AC092159.2	0.018350989	0.023972727	0.001469089	0.391189134	0.008824884
AC092071.1	0.018827441	0.816642246	0.705747604	0.944961846	0.006523768
AC092066.1	0.00300005	1.315117122	1.060895519	1.63025766	0.012443228
AC092048.1	8.86E-05	36319.07632	160.2093488	8233447.764	0.000147952
AC092022.1	0.002030643	437.7636077	3.221637356	59484.34137	0.01523315
AC091132.1	0.000476075	0.263487007	0.108659837	0.638924233	0.003165592
AC090286.4	0.024347792	8044671.048	48.99349473	1.32093E+12	0.009455595
AC090133.1	0.018073386	11.87676688	1.468094995	96.0820601	0.020344478
AC090095.1	0.019006757	69.05374939	2.27785161	2093.384961	0.014978277
AC087350.1	0.026174519	0.823730059	0.687728749	0.986626211	0.035186526
AC083949.1	0.016763982	0.600412541	0.36630124	0.984149602	0.043038711
AC083899.3	0.017301757	2.401094801	1.159676779	4.971433719	0.018328337
AC079779.7	0.035459012	1.18E-54	1.24E-103	1.12E-05	0.030924679
AC079767.4	0.002854414	0.766059875	0.632755828	0.927447376	0.006292274
AC079756.1	7.92E-11	3858312.004	1253.17935	11879043114	0.000215093
AC079298.1	0.02418531	5.537007641	1.466471755	20.90626942	0.011577598
AC079163.1	1.13E-07	4.61354E+15	3264549.023	6.52E+24	0.000793028
AC074289.1	0.006056092	0.663946247	0.469286915	0.939349905	0.020701944
AC074051.2	5.50E-10	35107.81015	121.9058681	10110738.34	0.000291895
AC073316.1	0.000387893	0.208093764	0.082503555	0.524862411	0.000882231
AC073255.1	0.000781506	37727072.94	67.35297872	2.11324E+13	0.009784078
AC069303.1	0.001422775	669835.6439	210.0344923	2136219556	0.001117829
AC069280.1	1.11E-16	3206977.718	2389.925223	4303358943	4.56E-05
AC069157.1	3.01E-07	43229928.94	4528.17558	4.12711E+11	0.000169643
AC069155.1	0.016736763	29.39416091	9.166717694	94.25584211	1.30E-08
AC068057.1	3.51E-05	106.4586416	1.401357083	8087.476424	0.034626902
AC067961.1	0.014285798	0.036135136	0.002457076	0.531423476	0.015482924
AC067735.1	0.015007969	519.0502521	6.484511345	41547.18063	0.005173974

AC061992.1	0.007561668	0.194231356	0.05741313	0.657093932	0.008407101
AC035144.1	0.041209796	27.98550299	1.372934328	570.4485363	0.030309491
AC027612.6	0.003310799	1.854169188	1.384171478	2.483755396	3.48E-05
AC026471.6	0.016729007	0.83578499	0.712527886	0.980363805	0.027554067
AC026393.1	0.031715793	44.94596177	1.906364129	1059.681856	0.018269551
AC024132.1	0.022937401	1.5065E+17	4165181499	5.45E+24	8.41E-06
AC023158.1	0.000497529	46.25117362	4.311904988	496.1081162	0.001539514
AC023157.1	0.046739174	0.142270011	0.021633605	0.935616423	0.042436279
AC021192.1	0.024973357	32.42030039	1.917591987	548.1227938	0.015898469
AC021188.4	0.021172037	0.36097584	0.185844065	0.701144569	0.002628772
AC021016.7	0.01324378	1.924852178	1.1084084	3.342681188	0.020047041
AC019181.2	0.01132792	0.615790962	0.39886287	0.950698942	0.028659908
AC018892.9	0.049793158	0.360687418	0.141280489	0.920830712	0.032970661
AC018634.9	0.004811315	0.006752269	8.70E-05	0.524246868	0.024398391
AC018462.2	0.010437517	0.12242697	0.016995448	0.881904585	0.03709601
AC018359.1	0.012522834	0.056771803	0.004338249	0.742935114	0.02878349
AC016993.1	0.000422158	1.61642174	1.060995619	2.462610773	0.025377941
AC016970.1	0.030893848	4.358548112	2.109990723	9.00332946	6.97E-05
AC016909.1	0.038291177	3.41E-06	1.18E-11	0.983259154	0.049693163
AC016907.2	0.001166953	6.78878E+16	279989.9515	1.65E+28	0.003758671
AC016722.4	0.015542623	0.341116413	0.135326811	0.859847403	0.022603071
AC015977.6	0.025979209	0.053205712	0.003979886	0.711288599	0.026590355
AC015820.1	4.44E-05	97172.86054	174.6033544	54080088.29	0.000370107
AC013472.3	0.00528614	0.000169911	7.72E-07	0.037384237	0.001609342
AC013429.5	0.000125669	0.000216649	1.21E-06	0.038694539	0.001426564
AC012668.2	0.020776705	0.080723636	0.006600184	0.987291415	0.048841187
AC012513.6	0.005513455	113.3804061	18.45526879	696.5553648	3.26E-07
AC012487.2	0.001355018	2.360588483	1.186436042	4.696736939	0.014404874
AC012476.1	0.039999717	0.535140156	0.304458365	0.940604758	0.029798751
AC012065.4	0.041543105	2.053295835	1.242631163	3.392819939	0.004989079
AC011899.9	0.003710959	0.589554607	0.386623156	0.899001079	0.014105619
AC011899.10	0.00313932	0.126830565	0.019173228	0.83898193	0.032185993

AC011284.3	0.002300149	0.119100375	0.027112846	0.523180026	0.004833757
AC011239.2	0.029094691	0.186630686	0.034943969	0.99676752	0.049558899
AC010980.2	0.009123577	1.596694927	1.051062469	2.425578654	0.028278297
AC010970.1	9.22E-06	7292518015	3260.510927	1.63106E+16	0.002331221
AC010967.1	0.00010915	77370.4633	14.87552841	402418551.1	0.009927202
AC010931.1	1.05E-07	759.8098151	50.94787721	11331.40352	1.50E-06
AC010746.3	0.004302749	0.02337051	0.000597087	0.914741717	0.044686682
AC010633.1	0.012843703	0.132925257	0.025450956	0.69424205	0.0167268
AC009970.1	0.012829979	0.212198935	0.059289428	0.75946741	0.017177684
AC009961.5	0.002434809	0.239648782	0.082640864	0.694953273	0.008541213
AC009473.1	0.03947706	0.509878688	0.28086211	0.925636701	0.026832329
AC009299.3	0.009787459	0.691857082	0.556131583	0.860706775	0.000945493
AC009299.2	0.01108292	0.163991577	0.045803367	0.587145424	0.005465809
AC009237.4	0.000798455	1.74154E+11	285378.3551	1.06279E+17	0.000140037
AC009237.2	0.043007564	3.27475E+13	7699900.363	1.39E+20	6.44E-05
AC009166.7	0.007078917	218.4329012	2.945757005	16197.17182	0.014218248
AC009133.17	0.003932914	0.265105062	0.103973236	0.675949857	0.005435076
AC009127.1	0.003134881	610.2514626	1.34813838	276237.85	0.039810656
AC009120.3	0.000906249	0.736839346	0.576148977	0.942346934	0.014971347
AC008753.1	0.013480852	10.73312193	2.189986147	52.60302971	0.003426913
AC008622.1	0.028907202	2.572969216	1.11111293	5.958143779	0.027391508
AC008278.2	0.000306358	0.277237287	0.10078413	0.762625166	0.01296062
AC007880.1	0.01334229	0.020091949	0.001287869	0.313452986	0.005310147
AC007879.5	0.018965714	1.962484021	1.049106201	3.671071172	0.034859121
AC007392.3	0.023303044	0.02009947	0.001255382	0.321805403	0.005757715
AC007386.4	0.010769934	0.60008886	0.410778525	0.876644271	0.008272071
AC007318.5	0.005535433	1.319082076	1.09500021	1.589020266	0.003552873
AC007255.8	0.002619918	1.380988895	1.053588452	1.81012835	0.019383777
AC007163.6	0.040413739	4.189529072	1.881310704	9.329747501	0.000453086
AC007163.3	0.048714366	4.976285144	1.411797917	17.54033884	0.01254306
AC007128.1	0.014558702	1.329766397	1.019369465	1.734678867	0.03560452
AC007050.1	0.006237432	659.3119176	2.201254928	197474.7219	0.025669834

AC006942.4	0.035918466	0.626659705	0.427710253	0.918150508	0.01647805
AC006547.13	0.034821327	0.639593727	0.419592058	0.974947279	0.037715943
AC006539.3	0.000514773	0.363878905	0.188152389	0.703726685	0.002663896
AC006534.3	0.024408078	0.439932793	0.194652876	0.994287197	0.048411876
AC006380.1	0.000493195	29497808.63	79.99065318	1.08778E+13	0.008538645
AC006369.2	7.67E-05	0.312351451	0.176924524	0.551440957	6.01E-05
AC006129.2	9.28E-05	0.636059373	0.487555467	0.829795898	0.000852119
AC006129.1	0.004559069	0.350971711	0.178027063	0.691923688	0.002499684
AC006116.1	0.007786746	0.698119402	0.49618369	0.982238453	0.039128259
AC006042.7	0.034598067	0.309939527	0.118341768	0.811738003	0.017099393
AC006028.11	0.030730979	0.082193973	0.007539366	0.896076601	0.040365467
AC006014.8	0.001362444	0.02037483	0.000740093	0.560921239	0.021347822
AC005355.2	0.000868071	1.43265969	1.199134002	1.711663405	7.49E-05
AC005300.5	0.008853492	0.163516081	0.041041053	0.651482045	0.010242735
AC005288.1	0.03716603	0.318669232	0.113145218	0.897519854	0.030417254
AC005255.3	0.044473742	1.290434573	1.001946065	1.661987052	0.048265513
AC005150.2	0.014202091	41.80179487	7.56557743	230.9658543	1.87E-05
AC005077.14	0.001737342	1.281850286	1.121469212	1.465167423	0.000271629
AC005077.12	0.025321765	1.428027999	1.107722183	1.840952539	0.005969879
AC005076.5	0.008194951	0.589302238	0.370078905	0.938386713	0.025888578
AC005037.3	0.010405696	2.560306307	1.261764729	5.195238254	0.009214888
AC005037.1	0.002327638	17.09265713	2.345402255	124.566661	0.005091863
AC004866.3	0.03519	15099281808	1582.465168	1.44072E+17	0.004258149
AC004485.3	0.017751212	1.09E-07	2.87E-13	0.041601725	0.014488655
AC004066.3	0.017314085	0.430947664	0.234862448	0.790743225	0.006566263
AC002519.8	0.030644215	0.049489064	0.003282997	0.74601572	0.029882539
AC002480.2	0.013316156	2.38E-06	9.00E-11	0.062966929	0.012696639
AC002398.9	0.018992862	12.4502423	1.600617062	96.84298447	0.015978882
ABI3BP	0.002969103	0.791373149	0.661342638	0.946969734	0.010620937
ABHD14A	0.029219454	0.745724974	0.59444765	0.935499934	0.011201701
ABCD2	0.035440959	0.450206761	0.237051094	0.855031394	0.014747347
ABCC8	0.004994912	0.542970223	0.298362912	0.988114309	0.045597061

ABCC6P2	0.000975873	0.631699592	0.477852973	0.835077726	0.001257207
ABCC6P1	0.011737685	0.677069522	0.481223382	0.952620249	0.025182638
ABCC6	0.007214289	0.766344633	0.635622512	0.923951065	0.005289427
ABCC2	0.01641664	1.190954913	1.100806709	1.288485611	1.35E-05
ABCC12	0.007060449	0.044714375	0.003210256	0.622808671	0.020760306
ABCB10P4	0.033559176	4.059150476	1.427385896	11.54327125	0.008606768
ABCA8	0.00579955	0.654925263	0.494989523	0.866537736	0.003049058
ABCA6	0.035284678	0.511363937	0.304576684	0.858545941	0.011185142
ABCA5	0.027157353	0.701858227	0.510869542	0.96424807	0.028916448
ABCA3	0.01296952	0.851234843	0.777788072	0.931617216	0.000467836
ABAT	8.92E-06	0.627524481	0.504594456	0.780402895	2.80E-05
A2MP1	0.000789214	0.015229958	0.000620613	0.373745925	0.01038606
A2M-AS1	0.025889146	0.43066873	0.216846284	0.855331949	0.016113651
A2M	0.03635591	0.865952386	0.767586037	0.976924407	0.019312523

Table S5. The correlations of 222 autophagy-related genes with the fifteen-gene signature risk score examined by the Pearson correlation coefficient.

Gene symbol	r	P-value
GAPDH	0.636398223	4.00E-58
BIRC5	0.586764309	1.39E-47
ERO1L	0.56158587	6.74E-43
DAPK2	-0.485664782	5.92E-31
DLC1	-0.469843388	8.04E-29
EIF2S1	0.467299031	1.73E-28
DRAM1	-0.466205256	2.40E-28
SPHK1	0.445133293	1.05E-25
ATIC	0.434213554	2.08E-24
GNAI3	0.430734305	5.28E-24
NAMPT	0.424744472	2.55E-23
DAPK1	-0.383092611	6.33E-19
EIF4EBP1	0.38242139	7.37E-19
FADD	0.381942606	8.21E-19
PRKCD	-0.380598426	1.11E-18
ITGB1	0.377831979	2.06E-18
HSPB8	-0.371602696	8.09E-18
IKBKB	-0.361099882	7.61E-17
RAC1	0.360441618	8.73E-17
CCR2	-0.358494785	1.31E-16
HIF1A	0.35369732	3.52E-16
ATG16L2	-0.347517697	1.22E-15
ERN1	-0.341266829	4.20E-15
EIF4G1	0.338630547	7.01E-15
PRKAB1	-0.33477402	1.47E-14
NCKAP1	0.331931736	2.52E-14
ATG16L1	0.331363204	2.81E-14
BCL2	-0.329293226	4.14E-14
IL24	-0.327841818	5.42E-14
RELA	0.292994872	2.35E-11
SIRT2	-0.292964983	2.36E-11
MAP1LC3A	-0.28262637	1.23E-10
ULK2	-0.280789925	1.64E-10
NLRC4	-0.277111566	2.89E-10
ATG2B	-0.273591138	4.93E-10
GRID1	-0.269118125	9.61E-10
PRKCQ	-0.265834589	1.56E-09
ITGB4	0.263702688	2.12E-09
CX3CL1	-0.258246686	4.63E-09
BID	0.254381821	7.97E-09
HGS	0.25228027	1.07E-08
TSC1	-0.249368251	1.59E-08
FOS	-0.248188689	1.87E-08
ATG9A	0.243413037	3.54E-08
KLHL24	-0.24157891	4.51E-08

GABARAPL2	-0.240095859	5.48E-08
EEF2K	-0.238869231	6.43E-08
CDKN2A	0.235428671	1.00E-07
RAB1A	0.234369315	1.15E-07
ATG5	0.23305764	1.36E-07
MYC	0.230230739	1.94E-07
ITPR1	-0.229758979	2.05E-07
CALCOCO2	-0.225800922	3.35E-07
ARSA	-0.2250688	3.67E-07
CHMP2B	0.224996569	3.70E-07
CFLAR	-0.223889286	4.23E-07
RPS6KB1	0.222455108	5.04E-07
P4HB	0.216944577	9.71E-07
CASP1	-0.215390316	1.17E-06
CXCR4	-0.214182626	1.34E-06
EDEM1	-0.209700099	2.25E-06
MBTPS2	0.205757393	3.50E-06
DNAJB1	0.205421315	3.63E-06
CTSD	-0.205400161	3.64E-06
BCL2L1	0.202594004	4.97E-06
CASP3	0.191224319	1.67E-05
AMBRA1	-0.190989969	1.71E-05
NKX2-3	0.189831736	1.93E-05
HSPA8	0.189645856	1.97E-05
RPTOR	0.189475208	2.00E-05
HSP90AB1	0.189215687	2.05E-05
GABARAP	-0.188387198	2.24E-05
ULK3	-0.188197417	2.28E-05
TSC2	-0.18623744	2.78E-05
MAP1LC3C	-0.186214977	2.79E-05
PELP1	0.184847917	3.20E-05
KIF5B	0.182217207	4.16E-05
APOL1	0.182179895	4.17E-05
WDR45	-0.181108944	4.63E-05
ITGA6	0.17532838	8.11E-05
CHMP4B	0.174848576	8.49E-05
FOXO1	-0.17405425	9.15E-05
CLN3	-0.17307049	0.0001
DNAJB9	-0.172338998	0.000108
SESN2	-0.17171433	0.000114
BAG3	0.170371063	0.000129
RAB7A	0.170062402	0.000133
HDAC1	0.169067997	0.000146
RAF1	-0.168497584	0.000153
EIF2AK2	0.165906011	0.000194
FKBP1A	0.165396397	0.000203
WIPI2	0.164335977	0.000224
CAPN1	0.163988069	0.000231

RAB24	-0.163693327	0.000237
RHEB	0.162957765	0.000253
USP10	0.162192797	0.000271
BNIP3	0.159351565	0.000347
TM9SF1	0.157641415	0.000403
STK11	-0.154228724	0.000539
MAP1LC3B	-0.153441458	0.000576
HSPA5	0.152245521	0.000636
TNFSF10	-0.150724431	0.000722
MTMR14	-0.149301004	0.000811
WDFY3	-0.146870869	0.000988
NRG3	-0.146809216	0.000993
SIRT1	-0.145883318	0.00107
FOXO3	-0.144811984	0.001166
CAPNS1	0.142008595	0.001455
SERPINA1	-0.14028564	0.001663
MAPK8IP1	-0.136974847	0.002143
HDAC6	-0.136852434	0.002163
SH3GLB1	0.136165312	0.002278
CDKN1A	0.135842268	0.002334
RAB11A	0.135613755	0.002375
MLST8	0.13442554	0.002595
ULK1	0.131370704	0.003251
TBK1	0.131365421	0.003252
VEGFA	0.131155633	0.003302
RAB33B	-0.129387392	0.003754
DIRAS3	-0.128648832	0.003958
ARNT	-0.127880997	0.004182
ITGA3	0.12780367	0.004205
UVRAG	-0.12562113	0.004907
PARK2	-0.124255094	0.005398
PTEN	-0.123802173	0.005571
ATG3	0.121193103	0.006664
CD46	-0.1211851	0.006668
PRKAR1A	-0.120509359	0.006981
PARP1	0.116094338	0.00937
PINK1	-0.115491589	0.009747
CDKN1B	-0.113352325	0.011198
MAPK9	-0.113134044	0.011356
NFE2L2	-0.112204698	0.012052
EIF2AK3	-0.108940984	0.014803
GOPC	0.107895258	0.015795
NFKB1	-0.106526018	0.017181
SPNS1	-0.10624573	0.017477
MTOR	0.103576362	0.020532
VAMP7	0.102583382	0.021782
TP53INP2	0.101436612	0.023307
ATG10	0.100566288	0.024525

IFNG	0.098036196	0.028383
BNIP3L	-0.095426017	0.032898
CAPN10	-0.092024804	0.039691
IKBKE	-0.090983276	0.041994
NAF1	0.090808215	0.042393
PIK3R4	0.09023265	0.043724
TMEM74	0.089726991	0.044922
PEA15	-0.08968028	0.045034
ATG2A	-0.088955997	0.046803
ATG12	0.088089761	0.048994
SAR1A	0.087543	0.050421
MAP2K7	-0.087170376	0.051413
GRID2	0.085777549	0.055266
BAK1	0.085274207	0.056717
NRG2	-0.0842393	0.059798
TP53	-0.083574779	0.061849
CAMKK2	0.081384391	0.069022
GNB2L1	-0.076659175	0.086826
TUSC1	-0.075199807	0.093018
ATG9B	0.073468953	0.100814
ATG4A	-0.073000209	0.103012
PEX14	0.072860536	0.103674
CTSB	0.072741936	0.104239
IRGM	-0.072437372	0.105701
FAS	-0.070267988	0.11659
BAX	0.067966445	0.129083
RB1CC1	0.066908746	0.135161
ATF4	0.066573853	0.137131
PTK6	0.06334259	0.157286
CASP4	0.060971496	0.173445
LAMP1	-0.060902945	0.17393
ZFYVE1	-0.059169109	0.186532
PPP1R15A	-0.054709753	0.22201
EEF2	-0.054528719	0.223546
BNIP1	0.052864537	0.238019
PIK3C3	-0.052599111	0.240387
DDIT3	0.051698071	0.248548
TP73	-0.049222923	0.271956
RAB5A	0.048940045	0.274724
ARSB	-0.047696287	0.287121
ST13	0.047171499	0.292463
NBR1	-0.046108013	0.303492
SQSTM1	-0.045869078	0.306007
EGFR	0.044380832	0.321984
ATG7	0.042170933	0.34669
NRG1	0.041485183	0.354595
RGS19	0.036279957	0.418239
GAA	-0.034953471	0.43547

BECN1	0.032854237	0.463557
LAMP2	-0.032278179	0.471437
KIAA0226	0.030702276	0.493365
BIRC6	-0.03066916	0.493831
BAG1	-0.029227033	0.514375
ATG4D	0.028376951	0.52669
CAPN2	-0.026922432	0.548102
ATG4B	-0.025959446	0.562511
TP63	0.022229123	0.619982
CANX	-0.021018317	0.63917
CASP8	-0.018381813	0.68178
VAMP3	0.013619949	0.761278
RB1	0.011753105	0.793199
ERBB2	-0.010326485	0.817831
PEX3	-0.009967499	0.824059
ATG4C	-0.009676747	0.829111
WIPI1	0.00577083	0.897581
CCL2	-0.005439965	0.903424
MAPK3	-0.005089103	0.909625
GABARAPL1	-0.00490648	0.912855
FKBP1B	-0.004688045	0.91672
ATF6	0.002936275	0.947782
MAPK8	-0.002068491	0.963201
NPC1	-0.000856227	0.984763
MAPK1	-0.000304761	0.994576
C12orf44	NA	NA
C17orf88	NA	NA
CTSL1	NA	NA
FAM48A	NA	NA
KIAA0652	NA	NA
KIAA0831	NA	NA
TMEM49	NA	NA
WDR45L	NA	NA

Table S6. Enriched gene sets in HALLMARK collection in high and low risk groups (| NES |> 1, NOM p-val <0.05, and FDR q-val <0.25).

NAME	SIZE	ES	NES	NOM p-val	FDR q-val
**High risk group**					
HALLMARK_GLYCOLYSIS	198	0.626829	2.5584967	0	0
HALLMARK_UNFOLDED_PROTEIN_RESPONSE	107	0.62021124	2.5225718	0	0
HALLMARK_MTORC1_SIGNALING	195	0.685844	2.4932897	0	0
HALLMARK_MYC_TARGETS_V1	194	0.77574927	2.4883788	0	0
HALLMARK_MYC_TARGETS_V2	58	0.8362678	2.4551775	0	0
HALLMARK_G2M_CHECKPOINT	190	0.8219814	2.1874313	0	0.00109753
HALLMARK_E2F_TARGETS	195	0.86343986	2.1884105	0	0.001166416
HALLMARK_DNA_REPAIR	148	0.5083392	2.164359	0	0.001344019
HALLMARK_MITOTIC_SPINDLE	198	0.59759057	1.8531862	0.006289308	0.01953797
HALLMARK_UV_RESPONSE_UP	154	0.36208987	1.6796376	0.009861933	0.060963713
HALLMARK_HYPOXIA	191	0.47134387	1.813675	0.011472276	0.024610365
HALLMARK_CHOLESTEROL_HOMEOSTASIS	73	0.4231527	1.5738246	0.037113402	0.10075337
HALLMARK_REACTIVE_OXYGEN_SPECIES_PATHWAY	47	0.47216973	1.6185147	0.04842105	0.081922725
**Low risk group**					
HALLMARK_BILE_ACID_METABOLISM	112	-0.4856536	-1.8567673	0.00193424	0.14327507

Table S7. Correlations of risk score with 22 kinds of TICs tested by Spearman coefficient.

TIC	r	p-value
B.cells.naive	0.03557457	4.83E-01
B.cells.memory	-0.15056065	2.84E-03
Plasma.cells	-0.05609216	2.69E-01
T.cells.CD8	0.112767448	2.58E-02
T.cells.CD4.naive	0.010518615	8.36E-01
T.cells.CD4.memory.resting	-0.21218584	2.33E-05
T.cells.CD4.memory.activated	0.241094225	1.41E-06
T.cells.follicular.helper	0.112106331	2.66E-02
T.cells.regulatory..Tregs.	-0.12757481	1.16E-02
T.cells.gamma.delta	0.010743436	8.32E-01
NK.cells.resting	0.032627182	5.20E-01
NK.cells.activated	0.055741451	2.72E-01
Monocytes	-0.14365477	4.42E-03
Macrophages.M0	0.24114012	1.40E-06
Macrophages.M1	0.100311328	4.75E-02
Macrophages.M2	0.01962765	6.99E-01
Dendritic.cells.resting	-0.22693763	5.83E-06
Dendritic.cells.activated	0.092644694	6.72E-02
Mast.cells.resting	-0.41224864	1.79E-17
Mast.cells.activated	0.245482711	8.93E-07
Eosinophils	-0.02109836	6.77E-01
Neutrophils	0.19469946	1.07E-04

Table S8. Prognostic capacity of 22 TICs examined by Kaplan–Meier analysis.

TIC	P-value
Mast cells resting	0.00061317
Dendritic cells resting	0.02270994
B cells memory	0.05764483
Mast cells activated	0.09517642
Monocytes	0.15410061
Macrophages M1	0.17839695
Macrophages M0	0.18405602
Neutrophils	0.19812556
NK cells resting	0.21829716
T cells CD4 memory activated	0.27658392
Plasma cells	0.43978489
Macrophages M2	0.5779231
Dendritic cells activated	0.58829321
T cells follicular helper	0.61044847
T cells regulatory (Tregs)	0.75745083
B cells naive	0.83463272
T cells gamma delta	0.83631404
Eosinophils	0.87117524
T cells CD4 memory resting	0.88734002
T cells CD8	0.92482278
NK cells activated	0.99175363
T cells CD4 naive	0.99286373
